# Electronic cigarettes for smoking cessation

**DOI:** 10.1002/14651858.CD010216.pub9

**Published:** 2025-01-29

**Authors:** Nicola Lindson, Ailsa R Butler, Hayden McRobbie, Chris Bullen, Peter Hajek, Angela Difeng Wu, Rachna Begh, Annika Theodoulou, Caitlin Notley, Nancy A Rigotti, Tari Turner, Jonathan Livingstone-Banks, Tom Morris, Jamie Hartmann-Boyce

**Affiliations:** Nuffield Department of Primary Care Health SciencesUniversity of OxfordOxfordUK; National Drug and Alcohol Research CentreUniversity of New South WalesSydneyAustralia; National Institute for Health InnovationUniversity of AucklandAucklandNew Zealand; Wolfson Institute of Population HealthBarts & The London School of Medicine and Dentistry, Queen Mary University of LondonLondonUK; Norwich Medical SchoolUniversity of East AngliaNorwichUK; Tobacco Research and Treatment Center, Department of MedicineMassachusetts General Hospital and Harvard Medical SchoolBostonMassachusettsUSA; Cochrane AustraliaSchool of Public Health & Preventive Medicine, Monash UniversityMelbourneAustralia; Department of Population Health SciencesUniversity of LeicesterLeicesterUK; Department of Health Promotion and PolicyUniversity of MassachusettsAmherstMAUSA

**Keywords:** Humans, Bias, Electronic Nicotine Delivery Systems, Nicotine, Nicotine/administration & dosage, Randomized Controlled Trials as Topic, Smoking Cessation, Smoking Cessation/methods, Vaping

## Abstract

**Background:**

Electronic cigarettes (ECs) are handheld electronic vaping devices that produce an aerosol by heating an e‐liquid. People who smoke, healthcare providers, and regulators want to know if ECs can help people quit smoking, and if they are safe to use for this purpose. This is a review update conducted as part of a living systematic review.

**Objectives:**

To examine the safety, tolerability, and effectiveness of using EC to help people who smoke tobacco achieve long‐term smoking abstinence, in comparison to non‐nicotine EC, other smoking cessation treatments, and no treatment.

**Search methods:**

We searched the Cochrane Central Register of Controlled Trials (CENTRAL), MEDLINE, Embase, and PsycINFO to 1 February 2024 and the Cochrane Tobacco Addiction Group's Specialized Register to 1 February 2023, reference‐checked, and contacted study authors.

**Selection criteria:**

We included trials randomizing people who smoke to an EC or control condition. We included uncontrolled intervention studies in which all participants received an EC intervention. Studies had to report an eligible outcome.

**Data collection and analysis:**

We followed standard Cochrane methods for screening and data extraction. We used the risk of bias tool (RoB 1) and GRADE to assess the certainty of evidence. Critical outcomes were abstinence from smoking after at least six months, adverse events (AEs), and serious adverse events (SAEs). Important outcomes were biomarkers, toxicants/carcinogens, and longer‐term EC use. We used a fixed‐effect Mantel‐Haenszel model to calculate risk ratios (RRs) with a 95% confidence interval (CI) for dichotomous outcomes. For continuous outcomes, we calculated mean differences. Where appropriate, we pooled data in pairwise and network meta‐analyses (NMA).

**Main results:**

We included 90 completed studies (two new to this update), representing 29,044 participants, of which 49 were randomized controlled trials (RCTs). Of the included studies, we rated 10 (all but one contributing to our main comparisons) at low risk of bias overall, 61 at high risk overall (including all non‐randomized studies), and the remainder at unclear risk.

Nicotine EC results in increased quit rates compared to nicotine replacement therapy (NRT) (high‐certainty evidence) (RR 1.59, 95% CI 1.30 to 1.93; I^2^ = 0%; 7 studies, 2544 participants). In absolute terms, this might translate to an additional four quitters per 100 (95% CI 2 to 6 more). The rate of occurrence of AEs is probably similar between groups (moderate‐certainty evidence (limited by imprecision)) (RR 1.03, 95% CI 0.91 to 1.17; I^2^ = 0%; 5 studies, 2052 participants). SAEs were rare, and there is insufficient evidence to determine whether rates differ between groups due to very serious imprecision (RR 1.20, 95% CI 0.90 to 1.60; I^2^ = 32%; 6 studies, 2761 participants; low‐certainty evidence).

Nicotine EC probably results in increased quit rates compared to non‐nicotine EC (moderate‐certainty evidence, limited by imprecision) (RR 1.46, 95% CI 1.09 to 1.96; I^2^ = 4%; 6 studies, 1613 participants). In absolute terms, this might lead to an additional three quitters per 100 (95% CI 1 to 7 more). There is probably little to no difference in the rate of AEs between these groups (moderate‐certainty evidence) (RR 1.01, 95% CI 0.91 to 1.11; I^2^ = 0%; 5 studies, 840 participants). There is insufficient evidence to determine whether rates of SAEs differ between groups, due to very serious imprecision (RR 1.00, 95% CI 0.56 to 1.79; I^2^ = 0%; 9 studies, 1412 participants; low‐certainty evidence).

Compared to behavioural support only/no support, quit rates may be higher for participants randomized to nicotine EC (low‐certainty evidence due to issues with risk of bias) (RR 1.96, 95% CI 1.66 to 2.32; I^2^ = 0%; 11 studies, 6819 participants). In absolute terms, this represents an additional four quitters per 100 (95% CI 3 to 5 more). There was some evidence that (non‐serious) AEs may be more common in people randomized to nicotine EC (RR 1.18, 95% CI 1.10 to 1.27; I^2^ = 6%; low‐certainty evidence; 6 studies, 2351 participants) and, again, insufficient evidence to determine whether rates of SAEs differed between groups (RR 0.93, 95% CI 0.68 to 1.28; I^2^ = 0%; 12 studies, 4561 participants; very low‐certainty evidence).

Results from the NMA were consistent with those from pairwise meta‐analyses for all critical outcomes. There was inconsistency in the AE network, which was explained by a single outlying study contributing the only direct evidence for one of the nodes.

Data from non‐randomized studies were consistent with RCT data. The most commonly reported AEs were throat/mouth irritation, headache, cough, and nausea, which tended to dissipate with continued EC use. Very few studies reported data on other outcomes or comparisons; hence, evidence for these is limited, with CIs often encompassing both clinically significant harm and benefit.

**Authors' conclusions:**

There is high‐certainty evidence that ECs with nicotine increase quit rates compared to NRT and moderate‐certainty evidence that they increase quit rates compared to ECs without nicotine. Evidence comparing nicotine EC with usual care or no treatment also suggests benefit, but is less certain due to risk of bias inherent in the study design. Confidence intervals were, for the most part, wide for data on AEs, SAEs, and other safety markers, with no evidence for a difference in AEs between nicotine and non‐nicotine ECs nor between nicotine ECs and NRT, but low‐certainty evidence for increased AEs compared with behavioural support/no support. Overall incidence of SAEs was low across all study arms. We did not detect evidence of serious harm from nicotine EC, but longer, larger studies are needed to fully evaluate EC safety. Our included studies tested regulated nicotine‐containing EC; illicit products and/or products containing other active substances (e.g. tetrahydrocannabinol (THC)) may have different harm profiles.

The main limitation of the evidence base remains imprecision due to the small number of RCTs, often with low event rates. Further RCTs are underway. To ensure the review continues to provide up‐to‐date information to decision‐makers, this is a living systematic review. We run searches monthly, with the review updated when relevant new evidence becomes available. Please refer to the *Cochrane Database of Systematic Reviews* for the review's current status.

## Plain language summary

**Can electronic cigarettes help people stop smoking, and do they have any unwanted effects when used for this purpose?**

**What are electronic cigarettes?**

Electronic cigarettes (e‐cigarettes) are handheld devices that work by heating a liquid that usually contains nicotine and flavourings. E‐cigarettes allow users to inhale nicotine in a vapour rather than smoke. Because they do not burn tobacco, e‐cigarettes, in general, do not expose users to the same levels of chemicals that can cause diseases in people who use conventional cigarettes.

Using an e‐cigarette is commonly known as 'vaping'. Many people use e‐cigarettes to help them to stop smoking tobacco. In this review we focus primarily on e‐cigarettes containing nicotine.

**Why did we do this Cochrane review?**

Stopping smoking lowers the risk of many diseases. Many people find it difficult to stop smoking. We wanted to find out if using e‐cigarettes could help people to stop smoking, and if people using them for this purpose experience any unwanted effects.

**What did we do?**

We searched for studies that looked at the use of e‐cigarettes for stopping smoking.

We looked for randomized controlled trials, in which the treatments people received were decided at random. This type of study usually gives the most reliable evidence about treatment effects. We also looked for studies in which everyone received e‐cigarette treatment. We also included studies that gave e‐cigarettes to people who smoked and monitored their health even if there was no randomized group, because such studies can contribute to our understanding of health effects.

We were interested in:

· how many people stopped smoking for at least six months; and

· how many people had unwanted effects, reported on after at least one week of use.

**Search date**

We included evidence published up to 1 February 2024.

**What we found**

We found 90 studies, which included 29,044 adults who smoked. The studies compared nicotine e‐cigarettes with:

· nicotine replacement therapy, such as patches or gum;

· varenicline (a medicine to help people stop smoking);

· e‐cigarettes without nicotine;

· heated tobacco (products designed to heat tobacco to a high enough temperature to release vapour, without burning it or producing smoke; these differ from e‐cigarettes because they heat tobacco leaf/sheet rather than a liquid);

· other types of nicotine‐containing e‐cigarettes (e.g. pod devices, newer devices);

· behavioural support, such as advice or counselling; or

· no support for stopping smoking.

Most studies took place in the USA (39 studies), the UK (20), and Italy (9).

**What are the results of our review?**

People are more likely to stop smoking for at least six months using nicotine e‐cigarettes than using nicotine replacement therapy (7 studies, 2544 people), or e‐cigarettes without nicotine (6 studies, 1613 people).

Nicotine e‐cigarettes may help more people to stop smoking than no support or behavioural support only (11 studies, 6819 people).

For every 100 people using nicotine e‐cigarettes to stop smoking, 8 to 10 might successfully stop, compared with only 6 of 100 people using nicotine‐replacement therapy, 7 of 100 using e‐cigarettes without nicotine, or 4 of 100 people having no support or behavioural support only.

We are uncertain if there is a difference between how many unwanted effects occur using nicotine e‐cigarettes compared with nicotine replacement therapy, no support or behavioural support only. There was some evidence that non‐serious unwanted effects were more common in groups receiving nicotine e‐cigarettes compared to no support or behavioural support only. Low numbers of unwanted effects, including serious unwanted effects, were reported in studies comparing nicotine e‐cigarettes to nicotine replacement therapy. There is probably no difference in how many non‐serious unwanted effects occur in people using nicotine e‐cigarettes compared to e‐cigarettes without nicotine.

The unwanted effects reported most often with nicotine e‐cigarettes were throat or mouth irritation, headache, cough, and feeling sick. These appeared similar to those people experience when using NRT. These effects were reduced over time as people continued using nicotine e‐cigarettes.

**How reliable are these results?**

Our results are based on few studies for most outcomes and, for some outcomes, the data varied widely.

We found evidence that nicotine e‐cigarettes help more people to stop smoking than nicotine replacement therapy. Nicotine e‐cigarettes probably help more people to stop smoking than e‐cigarettes without nicotine, but more studies are still needed to confirm this.

Studies comparing nicotine e‐cigarettes with behavioural or no support also showed higher quit rates in people using nicotine e‐cigarettes, but provide less certain data because of issues with study design.

Most of our results for the unwanted effects could change when more evidence becomes available.

**Key messages**

Nicotine e‐cigarettes can help people to stop smoking for at least six months. Evidence shows they work better than nicotine replacement therapy, and probably better than e‐cigarettes without nicotine.

They may work better than no support, or behavioural support alone, and they may not be associated with serious unwanted effects.

However, we still need more evidence, particularly about the effects of newer types of e‐cigarettes that have better nicotine delivery than older types of e‐cigarettes, as better nicotine delivery might help more people quit smoking.

## Summary of findings

**Summary of findings 1:** Nicotine EC compared to NRT for smoking cessation

**Nicotine EC compared to NRT for smoking cessation**						
**Patient or population:** people who smoke cigarettes, aged 18 or older **Setting:** various settings in New Zealand, UK, USA **Intervention:** nicotine EC **Comparison:** NRT						
**Outcomes**	Anticipated absolute effects^*^ (95% CI)		**Relative effect (95% CI)**	**№ of participants (studies)**	**Certainty of the evidence (GRADE)**	**Comments**
	**Events with NRT**	**Events with Nicotine EC**				
Smoking cessation at 6 months to 1 year Assessed with biochemical validation	Study population		RR 1.59 (1.30 to 1.93)	2544 (7 RCTs)	⊕⊕⊕⊕ HIGH	‐
	6 per 100	10 per 100 (8 to 12)				
Adverse events at 4 weeks to 6 to 9 months Assessed by self‐report	Study population		RR 1.03 (0.91 to 1.17)	2052 (5 RCTs)	⊕⊕⊕⊝ MODERATE^a^	‐
	23 per 100	24 per 100 (21 to 27)				
Serious adverse events at 4 weeks to 1 year Assessed via self‐report and medical records	Study population		RR 1.20 (0.90 to 1.60)	2761 (6 RCTs)	⊕⊕⊝⊝ LOW^b^	2 studies reported no events; effect estimate based on the 4 studies in which events were reported
	4 per 100	5 per 100 (4 to 6)				
*The estimated number of events in the intervention group (and its 95% confidence interval) is based on the assumed number of events in the comparison group and the **relative effect** of the intervention (and its 95% CI). For cessation, the assumed number of events in the control group is based on assumed quit rates for NRT assuming receipt of limited behavioural stop‐smoking support (as per Hartmann‐Boyce 2018a). The assumed risk for adverse events and serious adverse events is a weighted mean average of quit rates across control groups in contributing studies. **CI:** confidence interval; **EC**: electronic cigarette; **NRT**: nicotine replacement therapy; **RCT:** randomized controlled trial; **RR:** risk ratio						
**GRADE Working Group grades of evidence** **High certainty:** We are very confident that the true effect lies close to that of the estimate of the effect **Moderate certainty:** We are moderately confident in the effect estimate: The true effect is likely to be close to the estimate of the effect, but there is a possibility that it is substantially different **Low certainty:** Our confidence in the effect estimate is limited: The true effect may be substantially different from the estimate of the effect **Very low certainty:** We have very little confidence in the effect estimate: The true effect is likely to be substantially different from the estimate of effect						

^a^Downgraded one level due to imprecision; CIs consistent with benefit and harm. ^b^Downgraded two levels due to imprecision; fewer than 300 events and CIs encompass clinically important harm and clinically important benefit.

**Summary of findings 2:** Nicotine EC compared to non‐nicotine EC for smoking cessation

**Nicotine EC compared to non‐nicotine EC for smoking cessation**						
**Patient or population:** people who smoke cigarettes, aged 18 or older **Setting:** various settings in Canada, Italy, New Zealand, UK, USA **Intervention:** nicotine EC **Comparison:** non‐nicotine EC						
**Outcomes**	Anticipated absolute effects^*^ (95% CI)		**Relative effect (95% CI)**	**№ of participants (studies)**	**Certainty of the evidence (GRADE)**	**Comments**
	**Events with non‐nicotine EC**	**Events with nicotine EC**				
Smoking cessation at 6 to 12 months Assessed with biochemical validation	Study population		RR 1.46 (1.09 to 1.96)	1613 (6 RCTs)	⊕⊕⊕⊝ MODERATE^a,b^	‐
	7 per 100	10 per 100 (8 to 14)				
Adverse events at 1 week to 6 months Assessed via self‐report	Study population		RR 1.01 (0.91 to 1.11)	840 (5 RCTs)	⊕⊕⊕⊝ MODERATE^b^	‐
	9 per 100	9 per 100 (8 to 10)				
Serious adverse events at 1 week to 1 year Assessed via self‐report and medical records	Study population		RR 1.00 (0.56 to 1.79)	1412 (9 RCTs)	⊕⊕⊝⊝ LOW^c^	5 studies reported no events; effect estimate based on the 4 studies in which events were reported
	3 per 100	3 per 100 (2 to 6)				
*The estimated number of events in the intervention group (and its 95% confidence interval) is based on the assumed number of events in the comparison group and the **relative effect** of the intervention (and its 95% CI). For cessation, the assumed number of events in the control group is based on assumed quit rates for NRT assuming receipt of limited behavioural stop‐smoking support (as per Hartmann‐Boyce 2018a). The assumed risk for adverse events and serious adverse events is a weighted mean average of quit rates across control groups in contributing studies. **CI:** confidence interval; **EC**: electronic cigarette; **RCT:** randomized controlled trial; **RR:** risk ratio						
**GRADE Working Group grades of evidence** **High certainty:** We are very confident that the true effect lies close to that of the estimate of the effect **Moderate certainty:** We are moderately confident in the effect estimate: The true effect is likely to be close to the estimate of the effect, but there is a possibility that it is substantially different **Low certainty:** Our confidence in the effect estimate is limited: The true effect may be substantially different from the estimate of the effect **Very low certainty:** We have very little confidence in the effect estimate: The true effect is likely to be substantially different from the estimate of effect						

^a^Not downgraded for risk of bias. One of four studies considered high risk of bias; removing this study increased the direction of the effect in favour of the intervention. ^b^Downgraded one level due to imprecision; < 300 events overall. ^c^Downgraded two levels due to imprecision: confidence intervals encompass clinically significant harm as well as clinically significant benefit; < 300 events overall.

**Summary of findings 3:** Nicotine EC compared to behavioural support only/no support for smoking cessation

**Nicotine EC compared to behavioural support only/no support for smoking cessation**						
**Patient or population:** people who smoke cigarettes, aged 18 or older **Setting:** various settings in Canada, Italy, Switzerland, UK, USA **Intervention:** nicotine EC **Comparison:** behavioural support only/no support						
**Outcomes**	Anticipated absolute effects^*^ (95% CI)		**Relative effect (95% CI)**	**№ of participants (studies)**	**Certainty of the evidence (GRADE)**	**Comments**
	**Events with behavioural support only/no support**	**Events with nicotine EC**				
Smoking cessation at 6 to 12 months Assessed using biochemical validation	Study population		RR 1.96 (1.66 to 2.32)	6819 (11 RCTs)	⊕⊕⊝⊝ LOW^a^	‐
	4 per 100	8 per 100 (7 to 9)				
Adverse events at 12 weeks to 6 months Assessed via self‐report	Study population		RR 1.18 (1.10 to 1.27)	2351 (6 RCTs)	⊕⊕⊝⊝ LOW^a^	‐
	49 per 100	58 per 100 (54 to 62)				
Serious adverse events at 4 weeks to 8 months Assessed via self‐report and medical records	Study population		RR 0.93 (0.68 to 1.28)	4561 (12 RCTs)	⊕⊝⊝⊝ VERY LOW^a,b^	5 of the 12 studies reported no SAEs; MA is based on pooled results from 7 studies.
	4 per 100	4 per 100 (3 to 5)				
*The estimated number of events in the intervention group (and its 95% confidence interval) is based on the assumed number of events in the comparison group and the **relative effect** of the intervention (and its 95% CI). For cessation, the assumed number of events in the control group is based on assumed quit rates assuming receipt of limited behavioural stop‐smoking support (as per Hartmann‐Boyce 2018a). The assumed risk for adverse events and serious adverse events is a weighted mean average of quit rates across control groups in contributing studies. **CI:** confidence interval; **EC**: electronic cigarette; **MA:** meta‐analysis; **RCT:** randomized controlled trial; **RR:** risk ratio						
**GRADE Working Group grades of evidence** **High certainty:** We are very confident that the true effect lies close to that of the estimate of the effect **Moderate certainty:** We are moderately confident in the effect estimate: The true effect is likely to be close to the estimate of the effect, but there is a possibility that it is substantially different **Low certainty:** Our confidence in the effect estimate is limited: The true effect may be substantially different from the estimate of the effect **Very low certainty:** We have very little confidence in the effect estimate: The true effect is likely to be substantially different from the estimate of effect						

^a^Downgraded two levels due to risk of bias. Due to lack of blinding and differential support between arms, judged to be at high risk of bias. ^b^Downgraded two levels due to imprecision. Confidence intervals incorporate clinically significant benefit and clinically significant harm; < 300 events overall.

## Background

Throughout this review, we discuss (1) conventional cigarettes and (2) electronic cigarettes, defined as hand‐held and producing for inhalation an aerosol formed by heating an e‐liquid using a battery‐powered heating coil. In this review, all mention of smoking, smoking cessation, cigarette use, smoke intake, etc. concerns combustible tobacco cigarettes. When the text concerns electronic cigarettes, we use the abbreviation 'ECs'. EC users are sometimes described as 'vapers', and EC use as 'vaping'. We refer to ECs that do not contain nicotine as non‐nicotine ECs; these can also be conceptualized as placebo ECs, but we are using the term non‐nicotine EC, as they can be conceptualized as an intervention in themselves. This review does not address the use of vaping devices to inhale substances other than nicotine, such as cannabis.

### Description of the condition

Stopping smoking tobacco is associated with large health benefits. Despite most people who smoke wanting to quit, many find it difficult to succeed in the long term. Almost half who try to quit without support will not manage to stop for even a week, and fewer than five per cent remain abstinent one year after quitting (Hughes 2004).

Behavioural support and medications such as nicotine patches or gum increase the chances of quitting through providing nicotine to help alleviate withdrawal symptoms, but even with such support, long‐term quit rates remain low (Hartmann‐Boyce 2019; Hartmann‐Boyce 2021b; Livingstone‐Banks 2023). One of the limitations of current treatments is that, apart from providing nicotine more slowly and at lower levels than smoking, none adequately addresses the sensory, behavioural, and/or social aspects of smoking that ex‐smokers miss when they stop smoking (e.g. holding a cigarette in their hands, taking a puff, enjoyment of smoking, feeling part of a group). ECs may offer a way to overcome these limitations (Notley 2018b), and have become a popular consumer choice for smoking cessation support where regulations allow (Buss 2023).

There is no doubt that people become dependent on tobacco, and many find it difficult to stop smoking, primarily because of nicotine and its actions on the brain's reward system (Balfour 2004). However, developing dependence on tobacco smoking is a complex biopsychosocial process (Benowitz 2010; Rose 2006). Other tobacco chemicals, such as acetaldehyde and monoamine oxidase (MAO) inhibitors, seem to potentiate the effects of nicotine (Rose 2006). In addition, sensory and behavioural cues provide additional reinforcement of smoking behaviour (Rose 1993; Rose 2000) and may over time become almost as rewarding as nicotine. There are several lines of evidence to support this. Firstly, people who smoke appear to have a preference for cigarette smoke compared to other forms of nicotine delivery. This is partly related to the speed of nicotine delivery through smoke inhalation. However, even when nicotine is administered intravenously, it does not provide the same level of satisfaction or reward as smoking (Rose 2000; Westman 1996). Secondly, the local sensory effects of smoking (e.g. the ‘scratch’ in the back of the throat) may be important for enjoyment and reward. Numbing the sensations of cigarette smoke by anaesthetizing the upper and lower respiratory tract leads to less enjoyment of smoking (Rose 1985). Conversely, products that mimic the sensory effects of smoking on the mouth and throat (such as citric acid, black pepper, and ascorbic acid) reduce craving and some withdrawal symptoms, at least in the short term (Levin 1993; Rose 1994; Westman 1995). Thirdly, very low nicotine content cigarettes (VLNCs), which have e.g. 0.08 mg instead of the normal 1 mg, and so have negligible or no central effects, have also been investigated for their role in aiding smoking cessation (Przulj 2013). Despite delivering low levels of nicotine, VLNCs are satisfying over the initial few days of abstinence from nicotine (Donny 2007; Donny 2015; Pickworth 1999; Rose 2000). They also reduce tobacco withdrawal symptoms, including urges to smoke and low mood (Barrett 2010; Donny 2009; McRobbie 2016; Perkins 2010; Rose 2000), and have been shown to improve long‐term continuous abstinence rates in one study (Walker 2012). Social aspects of smoking, such as feeling part of a like‐minded group, or including smoking behaviour as part of one's social identity, are also elements of cigarette smoking that some people who smoke report to be drivers of cigarette use (Notley 2018a).

Considering the other factors that contribute to tobacco dependence, there is interest in developing smoking‐cessation products that not only help relieve the unpleasant effects of nicotine withdrawal, but that also act as effective substitutes for smoking behaviour and the rituals and sensations that accompany smoking, without the health risks associated with the inhalation of tobacco smoke. The only pharmaceutical treatment with some of these characteristics is the nicotine inhalator. However, these do not have greater cessation efficacy than other nicotine replacement therapy (NRT) products (Hajek 1999; Hartmann‐Boyce 2018a). This may in part be due to the considerable effort (e.g. 20 minutes of continuous puffing) needed to provide nicotine blood concentrations consistent with other NRT products (Schneider 2001). Adherence to correct use of the inhalator is low compared to other types of NRT (Hajek 1999). It is therefore possible that any advantage of sensorimotor replacement is diminished by low nicotine delivery and limited similarities between inhalator use and sensations of smoking (Bullen 2010).

### Description of the intervention

ECs are devices that produce, for inhalation, an aerosol formed by heating a solution (an e‐liquid) using a battery‐powered heating coil (E‐cigarette ontology 2021). The e‐liquid, usually comprising propylene glycol and glycerol, with or without nicotine and flavours, is stored in disposable or refillable cartridges or a reservoir or 'pod'. The commonly used term for this aerosol is vapour, which we use throughout the review. ECs are marketed as consumer products. Although routes are in place for licencing them as medicine or medical devices in some areas, no country yet has a licenced medicinal EC.

ECs provide sensations similar to smoking a cigarette. They provide taste and throat sensations that are closer to smoking than those provided by the nicotine inhalator (Barbeau 2013). The aerosol, typically referred to as vapour, looks like tobacco smoke, but is only visible when the user exhales after drawing on the mouthpiece, not when the device is being held. In qualitative studies, users report a sense of shared identity with other users, similar to tobacco‐smoking identity, and also report pleasure and enjoyment of use, suggesting that ECs may be viewed less as medical cessation aids but rather as acceptable alternatives to tobacco smoking (Cox 2017; Notley 2018a).

There are many different brands and models of EC available. Variation exists both in the device ('product') and consumable (e‐liquid used). There is a wide variation in the composition of e‐liquids (nicotine content, flavours, and other components) (Goniewicz 2012; Goniewicz 2014), with some users choosing to mix their own e‐liquids (Cox 2019b). Initial studies showed that early models of EC delivered very low amounts of nicotine to naïve users (Bullen 2010; Eissenberg 2010; Vansickel 2010). Later studies that have measured nicotine pharmacokinetics in both experienced and naïve EC users have found that some EC users can achieve blood nicotine levels similar to those achieved with smoking, albeit more slowly, and that their ability to do so often improves over time (Hajek 2015b; Vansickel 2012; Vansickel 2013; Yingst 2019a; Yingst 2019b).

Early in their development, ECs were designed to look like cigarettes and used disposable cartridges. These models were often called 'cig‐a‐likes'. The nicotine delivery from these products was low (Hajek 2017). The later refillable, or 'tank', products had a larger battery and a transparent container that users fill with an e‐liquid of their choice, and usually provide faster and more efficient nicotine delivery, allowing a wider choice of flavours and nicotine concentrations, and were typically used by experienced vapers who reportedly managed to switch to vaping completely (ASH 2019; Chen 2016; Dawkins 2013b; Farsalinos 2014). More recently, smaller 'pod' devices that use nicotine salt solutions have become available. This nicotine formulation reduces irritant effects and allows the delivery of higher nicotine levels that closely mimic the pharmacokinetic profile of nicotine delivery from cigarettes, despite the low battery power of the devices (Hajek 2020). In qualitative studies, pod devices have been highly rated by users in terms of satisfaction, usability (simple to use), affordability, and availability (Belderson 2024). The nicotine salts used in pods allow for high nicotine delivery; this may increase the likelihood that adult smokers are able to transition completely from conventional cigarettes (Prochaska 2022). Average nicotine concentrations in EC sold in the United States increased overall during 2013‐2018, for all flavour categories, and for rechargeable ECs (Romberg 2019). The EU Tobacco Products Directive (European Parliament 2014) does not allow sales of e‐liquids with nicotine content higher than 20 mg/mL, and so the US version of the Juul pod device (59 mg/nl nicotine) is not legally available within the EU (Huang 2019; Talih 2020). Most recently, there has been rapid growth in the use of disposable and single‐use devices (Tattan‐Birch 2022; Tattan‐Birch 2024). These are available in a range of attractive flavours, generally have a high nicotine content, are low cost, and have a closed system that is designed to be disposed of following use. According to ASH 2024, for adults in Great Britain, tank‐style devices are the most popular. For young people, the ASH 2024 report shows that disposables are now the most popular type of device. The different device types may differ significantly in their efficacy in helping people who smoke to quit, as they differ in delivery of nicotine. Nicotine itself, when delivered through mechanisms and doses similar to that delivered in traditional NRT, is not considered harmful (Hartmann‐Boyce 2018a). The safety profile of the different types of nicotine EC may be similar as they use the same constituents, although within the generic range of EC types there is some evidence to suggest EC providing less nicotine may pose higher risks. This is because low‐nicotine delivery devices need to be puffed with higher intensity to provide users with the nicotine levels that they seek, and more intensive puffing is accompanied by increased inhalation of potential toxicants (Dawkins 2016; Dawkins 2018; Smets 2019). Throughout this review, we refer to a nicotine‐containing EC as ‘nicotine EC’ and to nicotine‐free EC as 'non‐nicotine EC', which can also be considered 'placebo EC'. The 'placebo' comparison is a test just of the nicotine effect and not of the potential sensorimotor or behavioural and social replacement that the EC may provide.

There is no one agreed classification system for EC devices, and product development has moved so quickly that the definitions used within trials of the devices tested may no longer necessarily be fit for purpose. In this review, the definitions used are based on those drawn from the included trials. We currently label three different types of EC as 'cartridges' for devices with disposable cartridges and ‐ typically, but not always ‐ low nicotine delivery (e.g. cig‐a‐likes); refillable ECs for devices that people fill with their own choice of e‐liquids; and pods for the small devices with disposable pods that commonly use nicotine salts. To date, there are no trials of disposable devices, so we do not include this category in the current review. We may review this categorization system in future versions of the review as new trials and devices emerge.

### Why it is important to do this review

Since ECs appeared on the market in 2006, there has been a steady increase in their use. In Great Britain, only 27% of people who smoke have never tried vaping according to a recent survey (ASH 2023). EC use is most prevalent in people who currently (27%) and have previously (15%) smoked (ASH 2023). Only 1.1% of adults who have never smoked report currently using ECs (ASH 2023). Prevalence data from the USA in 2019 showed that 4.4% of adults were current EC users (Du 2020). Data from lower‐income countries suggest similar levels of EC use and awareness (Besaratinia 2019; Jiang 2016; Palipudi 2016).

Regulatory approaches being used for ECs currently vary widely, from no regulation to partial and complete bans (McNeill 2022). Within the USA, for example, the Food and Drug Administration (FDA) has classified EC as tobacco products and laws include prohibition of EC use indoors, a requirement for retailers to have a license to sell, and prohibition of sales to minors. Laws prohibiting sales to minors apply nationwide, but other laws vary by state (Du 2020). The European Union includes ECs in their Tobacco Products Directive, except where therapeutic claims are made or in instances where they contain over 20 mg/nl of nicotine (European Parliament 2014).

Categorical statements about the toxicity of ECs are not possible because of the large number of devices and liquids available and the frequent addition of new products to the market. In 2019, cases of severe lung injury associated with EC use were reported in the USA and, by February 2020, there were around 2800 hospitalized cases and 68 deaths (CDC 2020). This illness, which was termed E‐cigarette or Vaping‐Associated Lung Injury (EVALI), caused concern throughout the world (Hall 2020) and a negative change in people's perception of the risks of EC use compared to smoking (Tattan‐Birch 2020). These cases were somewhat at odds with data from trials and cohort studies, and it was later found that these injuries were related to use of tetrahydrocannabinol (THC)‐containing products adulterated with vitamin E acetate (Blount 2020; Hartnett 2020).

Amongst those brands of nicotine EC that have been tested, levels of toxins have been found to be substantially lower than in cigarettes (Hajek 2014; McNeill 2022). Long‐term effects beyond 12 months are unclear, although based on what is known about liquid and vapour constituents and patterns of use, a report from the UK's Royal College of Physicians has concluded that using an EC is likely to be considerably safer than smoking (RCP 2016). The US National Academies of Sciences, Engineering, and Medicine (NASEM) concluded that ECs are likely to be far less harmful than continuing to smoke cigarettes, with the caveat that the long‐term health effects of e‐cigarette use are not yet known (NASEM 2018).

Despite general acknowledgement that EC use exposes the user to fewer toxicants and at lower levels than smoking cigarettes (McNeill 2021; McNeill 2022; NASEM 2018; RCP 2024), in some countries and settings there remains hesitancy in making these products available to people who smoke as a harm‐reduction tool or smoking‐cessation aid (e.g. McDonald 2020). Concerns include that the long‐term effects of EC use on health are not yet known, the possible harms of second‐hand EC vapour inhalation, the lack of quality control measures, and that ECs may undermine smoke‐free legislation if used in smoke‐free spaces (McNeill 2022). Of concern is also the involvement of the tobacco industry and that ECs may be a gateway to smoking initiation or nicotine dependence amongst nicotine‐naïve users or may prolong continued dual use of tobacco amongst cigarette smokers (McNeill 2022). A report from the US Preventive Services Taskforce concluded "that the current evidence is insufficient to assess the balance of benefits and harms of electronic cigarettes (e‐cigarettes) for tobacco cessation in adults" (USPFTS 2021). However, others suggest that potential benefits outweigh potential disadvantages (Farsalinos 2014; Hajek 2014; McNeill 2021; McNeill 2022; NASEM 2018; RCP 2024).

People who smoke, healthcare providers, and regulators are interested in knowing if ECs can help people to quit and if it is safe to use them to do so. In particular, healthcare providers have an urgent need to know what they should recommend to people to help them to stop smoking. The largest health gains are achieved from stopping smoking completely, as opposed to reducing cigarette consumption and, as such, this review focuses on the effectiveness of ECs in aiding complete smoking cessation.

This review was first published in 2014, and updated in 2016, 2020, 2021, 2022, and 2024. We published an update to the protocol in 2023 (see Lindson 2023b).

Following publication of the 2020 update of this review, we are maintaining it as a living systematic review (Brooker 2019). This means we are continually running searches and incorporating new evidence into the review. For more information about the living systematic review methods being used, see Appendix 1. A living systematic review approach is appropriate for this review for three reasons. First, the review addresses an important public health issue: the role of ECs in enabling people who smoke to stop smoking, with the potential for substantial ongoing individual and societal benefits, depending on the extent of effectiveness. Secondly, there remains uncertainty in the existing evidence; more studies are needed to confirm the degree of benefit for different comparisons and product types, and there is considerable uncertainty about adverse events and other markers of safety. Thirdly, we are aware of multiple ongoing trials that are likely to have an important impact on the conclusions of the review.

## Objectives

To examine the safety, tolerability, and effectiveness of using electronic cigarettes (ECs) to help people who smoke tobacco achieve long‐term smoking abstinence, in comparison to non‐nicotine EC, other smoking cessation treatments, and no treatment.

## Methods

### Criteria for considering studies for this review

#### Types of studies

We include randomized controlled trials (RCTs) and randomized cross‐over trials in which people who smoke are randomized to ECs or to a control condition. RCTs are the best available primary evidence; we also include uncontrolled intervention studies in which all participants are given an EC intervention. These studies have the potential to provide information on harms and longer‐term use.

We include studies regardless of their publication status or language of publication.

#### Types of participants

Participants are people defined as currently smoking cigarettes at enrolment into the studies. Participants could be motivated or unmotivated to quit. We include studies that recruited pregnant people and include their data in our pairwise meta‐analyses and narrative syntheses; however, we will not include their data in our network meta‐analyses (NMA) (described in our Data synthesis section) as some of the comparisons eligible for inclusion are unlikely to be offered to this population (e.g. varenicline).

Should a study meet all other criteria but include only a subset of eligible participants (e.g. a study on people who currently smoke and people who formerly smoked), we would only include data on the subgroup of participants who met our inclusion criteria. If these data were not available, we would include the study if at least 80% of participants met our inclusion criteria and would test the exclusion of the study in a sensitivity analysis.

We include participants regardless of age.

#### Types of interventions

Any type of EC or intervention intended to promote EC use for smoking cessation, including studies that did not measure smoking cessation but provided ECs with the instruction that they be used as a complete substitute for cigarette use. ECs may or may not contain nicotine. For the purpose of the NMA, we assume that the interventions were jointly randomizable for all participants except those who were pregnant.

##### Types of comparators

We compare nicotine ECs with alternative smoking cessation aids, including NRT or no intervention, with ECs without nicotine, and ECs added to standard smoking cessation treatment (behavioural or pharmacological or both) with standard treatment alone. We compare different types of EC (refillable, cartridge, nicotine salt, free‐base), different nicotine doses, and different flavours.

#### Types of outcome measures

##### Primary outcomes

- Cessation at the longest follow‐up point, at least six months from the start of the intervention, measured on an intention‐to‐treat basis using the strictest definition of abstinence, preferring biochemically validated results where reported
- Number of participants reporting any type of adverse event(s) at one week or longer (as defined by study authors)
- Number of participants reporting any type of serious adverse event(s) at one week or longer (as defined by study authors)

##### Secondary outcomes

Number of people still using study product (EC or pharmacotherapy) at longest follow‐up (at least six months). The product could be that provided by the study, or could be the same product type but bought independently by the participant.

Changes in the following measures at longest follow‐up (one week or longer):

- Carbon monoxide (CO), as measured through breath or blood
- Blood pressure
- Heart rate
- Blood oxygen saturation
- Lung function measures
- Known toxins/carcinogens, as measured through blood, urine or saliva (toxicant names and abbreviations are listed in Appendix 2)

Studies had to set out to measure one of the primary or secondary outcomes above to be eligible for inclusion. If a study set out to measure an eligible outcome but did not measure and/or report results on this outcome, we would still include this study and flag its missing data in the results section.

In addition, we set out to include any measure of an association between withdrawal symptoms and smoking cessation at six months or longer, as long as withdrawal was measured using a validated scale designed explicitly to investigate smoking withdrawal or craving. We added this because British guidelines now specify that efforts should be made to provide EC in a way that will reduce symptoms of withdrawal in people who smoke (NICE 2021). However, no studies provided data on this.

### Search methods for identification of studies

#### Electronic searches

Searches are conducted monthly. This update includes results from searches conducted up to 1 February 2024:

- Cochrane Tobacco Addiction Group Specialized Register (CRS‐Web up to 1 February 2023);
- Cochrane Central Register of Controlled Trials (CENTRAL; 2024, Issue 1) via CRS‐Web;
- MEDLINE (OVID SP; 1 January 2004 to 1 February 2024);
- Embase (OVID SP; 1 January 2004 to 1 February 2024);
- PsycINFO (OVID SP; 1 January 2004 to 1 February 2024);
- ClinicalTrials.gov (via CENTRAL; 2024, Issue 1);
- WHO International Clinical Trials Registry Platform (ICTRP: www.who.int/ictrp/en/, via CENTRAL; 2024, Issue 1).

We did not search the Cochrane Tobacco Addiction Group Specialized Register beyond 1 February 2023 as it ceased to be maintained. At the time of the last search, the Register included the results of searches of MEDLINE (via OVID) to update 20221222; Embase (via OVID) to week 202251; and PsycINFO (via OVID) to update 20221219. See the Tobacco Addiction Group website for full search strategies and a list of other resources searched.

For the first version of the review, we also searched CINAHL (EBSCO Host) (2004 to July 2014). We did not search this database from 2016 onwards, as it did not contribute additional search results to the first version of the review. The search terms were broad and included 'e‐cig$' OR 'elect$ cigar$' OR 'electronic nicotine'. The search for the 2016 update added the terms 'vape' or 'vaper' or 'vapers' or 'vaping'. The 2020 searches added further terms, including the MESH heading 'Electronic Nicotine Delivery Systems' and terms to limit by study design. All current search strategies are listed in Appendix 3. The previously used search strategy is shown in Appendix 4. The search date parameters of the original searches were limited to 2004 to the present, due to the fact that ECs were not available before 2004.

#### Searching other resources

We searched the reference lists of eligible studies found in the literature search and contacted authors of known trials and other published EC studies. We also searched abstracts from the Society for Research on Nicotine and Tobacco (SRNT) Annual Meetings up to 1 February 2024.

### Data collection and analysis

#### Selection of studies

Two review authors (for this update from: ARB, JHB, NL, AT) independently pre‐screened all titles and abstracts obtained from the search, using a screening checklist, and then independently screened full‐text versions of the potentially relevant papers for inclusion. We resolved any disagreements by discussion or with a third review author.

#### Data extraction and management

One review author extracted data on study characteristics (ARB), whereas two review authors (for this update: ARB and ADW) independently extracted outcome data, effect modifiers, and the information needed to make risk of bias judgements. We used a pre‐piloted data extraction form, and checked the forms for inconsistencies. We resolved any disagreements by discussion or with a third review author (NL). We extracted data on the following:

- Author
- Date and place of publication
- Study dates
- Study design
- Inclusion and exclusion criteria
- Setting
- Summary of study participant characteristics
- Summary of intervention and control conditions
- Number of participants in each arm
- Smoking cessation outcomes
- Type of biochemical validation (if any)
- Adverse events (AEs), serious adverse events (SAEs), number of people still using study product, and relevant biomarkers
- Continued EC use or pharmaceutical intervention (PI) use at longest follow‐up
- Data investigating the association between withdrawal and smoking cessation
- Assessment time points
- Study funding source
- Author declarations of interest
- Risk of bias in the domains specified below
- Additional comments

We adopted a broad focus to detect a variety of adverse events.

There were no papers that required translation*;* should there be in the future, we would seek a translator to assist us.

For studies that received tobacco or EC industry funding the study name is followed by an asterisk (*).

One review author (NL for this update) then entered the data contributing to pairwise network meta‐analysis (NMA) into Review Manager 2022 software for analyses, and another checked them (JHB for this update). Data for the NMA were collated by one author (ARB) and checked by NL and JHB.

#### Assessment of risk of bias in included studies

Two review authors (for this update: ARB and ADW) independently assessed the risks of bias for each included study, using the Cochrane risk of bias tool (RoB 1) (Higgins 2011). We resolved any disagreements by discussion or with a third review author (NL). This approach uses a domain‐based evaluation that addresses seven different areas: random sequence generation; allocation concealment; blinding of participants and providers; blinding of outcome assessment; incomplete outcome data; selective outcome reporting; and other potential sources of bias. We assigned a grade (low, high, or unclear) for risk of bias for each domain. We resolved disagreements by discussion or by consulting a third review author.

Specific considerations about judgements for individual domains in this review are outlined below:

- Random sequence generation/allocation concealment: We rated all non‐randomized studies at high risk in these domains.
- Blinding of participants and personnel: We did not evaluate this domain for non‐randomized studies, as we considered it not to be applicable. For randomized studies that did not use blinding, we considered studies to be at low risk in this domain if the intervention was compared to an active control of similar intensity, as we judged performance bias to be unlikely in this circumstance. If studies were unblinded and the comparator group was a minimal‐intervention control or of lower intensity than the intervention group, we considered the study to be at high risk of bias in this domain.
- Following the standard methods of the Cochrane Tobacco Addiction Group, we considered studies to be at low risk of detection bias (blinding of outcome assessment) if our primary outcome was objectively measured or if the intensity of the intervention was similar between groups, or both. For studies where cessation was measured, our judgement was based on whether cessation was biochemically verified. Where cessation was not measured, we judged this domain based on adverse or serious adverse events.
- Again, following the standard methods of the Cochrane Tobacco Addiction Group, we rated studies at high risk of attrition bias if loss to follow‐up was greater than 50% overall or if there was a difference in follow‐up rates of more than 20% between study arms.

We judged studies to be at high risk of bias overall if they were rated at high risk in at least one domain, and at low risk of bias overall if they were judged to be at low risk across all domains evaluated. We judged the remaining studies to be at unclear risk of bias overall.

#### Measures of treatment effect

We analyzed dichotomous data by calculating the risk ratio (RR). For cessation, we calculated the RR as (number of events in intervention condition/intervention denominator)/(number of events in control condition/control denominator) with a 95% confidence interval (CI), using data at the longest follow‐up period reported.

We analyzed continuous data (other measures of tobacco exposure) by comparing the difference between the mean change from baseline to follow‐up in the intervention and comparator groups, or by comparing absolute data at follow‐up where insufficient data were available on mean change. For outcomes other than cessation where data were reported at multiple time points, we used data at the longest follow‐up point at which ECs were still being provided, or their use was encouraged.

#### Unit of analysis issues

In the case of trials with multiple arms, we do not combine data between arms unless this is the way it has been presented by study authors, or there is no evidence of difference between similar trial arms for the outcome of interest. We note in our analyses where this is the case.

For all but one study, the unit of assignment was the individual. Dawkins 2020 assigned the condition based on homeless support service; this was a small pilot study with very few events and hence we judged clustering to have very little impact on our overall result. If larger cluster‐randomized trials are eligible in the future, we will assess whether the study authors have adjusted for this clustering, and whether this had an impact on the overall result. When clustering appears to have had little impact on the results, we will use unadjusted quit‐rate data; however, when clustering does appear to have an impact on results, we will adjust for this using the intraclass correlation (ICC).

For randomized cross‐over trials, we report results at the end of the first assignment period where available and where sufficiently long to meet our inclusion criteria for outcomes. All other outcomes from randomized cross‐over trials are reported narratively. We offer a narrative synthesis of data from non‐randomized studies and outcomes from comparative trials that are not reported with sufficient data for meta‐analysis, using effect direction plots as described in the *Cochrane Handbook for Systematic Reviews of Interventions,* where possible (Higgins 2021).

#### Dealing with missing data

For smoking cessation, we use a conservative approach, as is standard for the Cochrane Tobacco Addiction Group, treating participants with missing data as still smoking. We base the proportion of people affected by adverse events on the number of people available for follow‐up, and not the number randomized. For other outcomes, we use complete‐case data and do not attempt to impute missing values.

#### Assessment of heterogeneity

We assessed the clinical and methodological diversity between studies to guide our decision about whether data should be pooled. We were also guided by the degree of statistical heterogeneity, assessed by calculating the I^2^ statistic (Higgins 2003), and considering a value greater than 50% as evidence of substantial heterogeneity. We did not present pooled results where I^2^ values exceeded 75%.

For our NMA, we assessed global inconsistency using scatterplots of residual deviance in the NMA model against residual deviance in the Unrelated Mean Effects (UME) model. The UME model does not assume transitivity of treatment effects, so a smaller deviance in this model in comparison to the main NMA indicates inconsistency within the network. We also used scatterplots of leverage versus deviance to check the general model fit. We assessed local inconsistency using node‐split models for the main analyses of each outcome. We did not formally assess potential participant‐level effect moderators as a way to evaluate transitivity in our NMA, as previous work has shown these to be inconsistently reported in this body of studies (Hartmann‐Boyce 2021b; Hartmann‐Boyce 2022c; Lindson 2023a).

#### Assessment of reporting biases

Reporting bias can be assessed using funnel plots, where 10 or more RCTs contribute to an outcome. Where studies were included in an analysis but did not contribute data to the pooled effect (as zero events were reported), these were not included in the count of included studies when deciding whether to generate funnel plots. Therefore, there were only two analyses with sufficient studies to support this approach.

Had it been appropriate for our NMA, we would have adapted existing methods for assessing publication bias in standard systematic reviews by generating a funnel plot for each of the comparisons of interest and overlaying these plots on top of one another, while aligning the reference lines (representing the overall component effect). This is called a 'comparison adjusted funnel plot'. We would have considered studies distributed asymmetrically as potential evidence of publication bias. However, there were insufficient studies for each comparison to make this approach appropriate.

#### Data synthesis

We provide a narrative summary of the included studies.

##### Pairwise meta‐analyses

Where appropriate, we have pooled data from RCTs in pairwise meta‐analyses, grouping studies by comparison type and outcome. Data from single‐armed intervention studies are summarized narratively. Where presented per type of AE, rather than for all types together, these data were summarized narratively rather than meta‐analyzed.

For dichotomous data, we used a fixed‐effect Mantel‐Haenszel model to calculate the RR with a 95% CI, in accord with the standard methods of the Cochrane Tobacco Addiction Group for cessation studies.

For continuous outcomes, we pooled mean differences (or standardized mean differences for studies using different measures for the same construct), using the inverse variance approach (also with a 95% CI).

##### Network meta‐analyses

We also carried out three network meta‐analyses (NMA), as these can give further insight into comparative effects by drawing on data from both direct and indirect comparisons. NMA produces estimates of the relative effects between any pair of interventions in the network, usually providing more precise estimates than a single direct estimate (Chaimani A). Our three NMAs are as follows: one for each of our three primary outcomes: smoking cessation at six months follow‐up or longer; AEs at one‐week follow‐up or longer; and SAEs at one‐week follow‐up or longer. All NMAs were run using MetaInsight (Owen 2019). The models were Bayesian generalized linear models with non‐informative prior distributions on all parameters. Fixed effects were assumed in each case. MCMC simulations were run for 25,000 iterations, with the first 5,000 discarded, on four chains. Convergence was assessed through Gelman plots. Bayesian models were chosen because they are more flexible and have become the most common choice for NMA in recent years.

Our NMAs only included RCTs and excluded studies carried out on pregnant people, as specified in the Types of participants section. Interventions were classified as nodes as per our primary comparisons, but only including nodes where two or more studies contributed data. In addition, NRT was split into combined and single forms because evidence indicates there is a clinically significant difference in smoking cessation outcomes between them (Theodoulou 2023). In this update, the following interventions were eligible nodes (further nodes may be added as additional comparisons are added):

- Nicotine EC
- Non‐nicotine EC
- NRT (single form)
- NRT (combined fast‐acting form and patch)
- Nicotine EC plus single‐form NRT
- Non‐nicotine EC plus single‐form NRT
- Advice to use nicotine e‐cigarettes in a quit attempt (but no e‐cigarettes provided)
- No e‐cigarettes or pharmacotherapy

Studies that provided an additional intervention component, such as behavioural support, were included only if the additional component was matched between intervention arms and comparator arms, as is the case for this review overall.

Studies with no outcome events in any study arm were excluded. In contrast, studies with no outcome events in some but not all arms were included.

We did not conduct any ranking of interventions in the NMA.

#### Subgroup analysis and investigation of heterogeneity

For our pairwise analyses, we had planned to undertake subgroup analyses to investigate differences between studies, such as the following:

- Intensity of behavioural support used (as this could potentially influence cessation).
- Type of EC, e.g. cartridge; refillable; pod (as this could potentially influence all outcomes due to different delivery mechanisms).
- Instructions for EC use, e.g. study provision, length of provision, whether participants had a role in product choice (as this could potentially influence all outcomes, given variation in available devices and e‐liquids).
- Type of participants (this could potentially influence all outcomes, depending on, e.g. pre‐existing conditions, previous experience with EC).

However, there were too few studies to conduct such analyses. Should further studies become available in future, we will follow this approach. For continuous outcomes, we subgroup data based on whether absolute values or change scores were available. For adverse events, we subgroup data by length of follow‐up for descriptive purposes.

In the absence of sufficient data for subgroup analyses on EC type, in the text we specify the type of nicotine EC when reporting pooled results for cessation.

#### Sensitivity analysis

For both pairwise and network meta‐analyses, we conducted sensitivity analyses to detect whether pooled results were sensitive to the removal of studies judged to be at high risk of bias overall, and to the removal of studies reporting funding by the tobacco and/or vaping industry. We did this for all outcomes.

#### Summary of findings and assessment of the certainty of the evidence

Following standard Cochrane methodology, we created summary of findings tables for our three main comparisons using GRADEpro GDT: nicotine EC versus non‐nicotine EC; nicotine EC versus NRT; and nicotine EC versus behavioural support only/no support. We selected these comparisons a priori as being the most clinically relevant.

In the summary of findings tables, we present data on our primary outcomes (cessation at longest follow‐up, at least six months from baseline, and adverse events and serious adverse events at one week or longer, at the longest follow‐up at which participants were still being provided or encouraged to use EC) for these main comparisons, from our pairwise meta‐analyses only.

Following standard Cochrane methodology, we used the five GRADE considerations (study limitations, consistency of effect, imprecision, indirectness, and publication bias) to assess the certainty of the body of evidence for each outcome (also based on our pairwise meta‐analyses only), and to draw conclusions about the certainty of evidence within the text of the review. GRADE assessments were carried out by JHB and NL. If, in the future, results from NMA are inconsistent with those from our pairwise analyses, we will consider this as a possible justification for downgrading based on indirectness (as it would signal that direct and indirect evidence are incongruent).

We did not conduct certainty assessments for the NMA outcomes, as our primary analyses of interest were the pair‐wise comparisons.

## Results

### Description of studies

#### Results of the search

For this update, our bibliographic database searches identified 624 non‐duplicate records (See Figure 1 for PRISMA flow diagram). We screened all records and retrieved the full‐text papers of 34 potentially relevant articles. After screening and checking the full texts, we included 26 records, representing two new studies for this update (Pope 2024; Xu 2023*), 11 new articles linked to studies already identified, 11 new ongoing studies, and two records awaiting classification (see Characteristics of ongoing studies). Secondary study reports, commentaries, and correspondence relating to included studies are linked to studies in the reference section.

**1:**
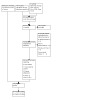
PRISMA diagram for 2024 update

#### Included studies

In total, we include 90 studies, with two new included studies and 88 studies included in the previous version of this review. Key features of these included studies are summarized below. Further details on each included study can be found in the Characteristics of included studies table.

#### Participants

The 90 included studies represented 29,044 participants. Thirty‐nine studies were conducted in the USA, 20 were conducted in the UK, nine in Italy, five in Australia, four in Greece, two each in New Zealand, Switzerland, and Canada, and one each in Belgium, Ireland, the Netherlands, Poland, the Republic of Korea, South Africa, and Turkey. All studies were conducted in adults who smoked. Twenty‐six studies exclusively recruited participants who were not motivated to quit smoking, and 45 studies exclusively recruited participants motivated to quit; motivation was not specified for the other studies. Thirty‐two studies recruited from specific population groups; these included nine studies that recruited participants based on physical health condition (heart attack, cancer, HIV, periodontitis, awaiting surgery, smoking‐related chronic disease), five studies that recruited participants with serious mental illness, four studies that recruited participants on treatment or having recently completed treatment for alcohol or other drug use, and three studies in dual users of EC and conventional cigarettes. Two studies recruited people accessing homeless centres or using supported temporary accommodation, and a further two recruited specifically within socially deprived areas. One study each recruited: people aged 55 or older, young adults, people who self‐identified as African‐American, pregnant women, people who had recently made a failed attempt to quit smoking, black and Latino participants, and people attending the emergency department.

#### Interventions and comparators

Three studies recruited dual users of combustible cigarettes and EC at baseline, and instructed them to continue using their own EC devices (Czoli 2019; Martinez 2021; Vickerman 2022). One study recruited users of combustible cigarettes only and provided information on using EC, but did not provide them with EC (Elling 2023). The remaining studies all provided some form of nicotine EC.

In two studies where nicotine ECs were provided on their own, nicotine levels were judged to be so low as to be clinically comparable to non‐nicotine EC (Lee 2019; Van Staden 2013*); we include these studies in non‐nicotine EC comparisons. Twelve studies compared nicotine EC with non‐nicotine EC, 26 studies compared nicotine EC to behavioural support only or to no support, and 20 studies compared nicotine EC to NRT. Six studies compared high‐ versus low‐nicotine EC devices (Caponnetto 2013a*; Cobb 2021; Kanobe 2022*Kimber 2021; Morris 2022*; White 2021), four studies included comparisons based on flavours (Edmiston 2022*; Morris 2022*; White 2021; Xu 2023*), two studies directly compared device types (Kimber 2021; Yingst 2020), and two studies directly compared a freebase nicotine to a salt‐based nicotine device (Morris 2022*; Russell 2021). Results from these studies are reported by comparison in Effects of interventions. Further details on the intervention and comparator groups (where applicable) for each study can be found in the Characteristics of included studies table.

Where reported in the primary research publications, details of the devices tested can also be found in the Characteristics of included studies table. Of the studies with sufficient data with which to judge, 31 used cartridge devices, 34 used refillable devices, four used both types, 10 used a pod device, and the remainder did not report device type.

#### Outcomes

Of the 90 included studies:

- 40 reported data on abstinence at six months or longer;
- 64 reported data on adverse events;
- 46 reported data on serious adverse events;
- 49 reported data on carbon monoxide;
- 12 reported data on heart rate;
- 14 reported data on blood pressure;
- 4 reported data on blood oxygen saturation;
- 16 reported data on at least one known toxin/carcinogen;
- 8 reported data on at least one measure of lung function;
- 20 reported data on study product use at six months or longer.

One study measured safety outcomes but did not report them in the text available at the time of writing (they may be forthcoming); hence, this study currently does not contribute any data to this review (Skelton 2022).

#### Study types

Forty‐nine studies were RCTs, 28 of which contributed to cessation analyses. Eight studies used randomized cross‐over designs, and the remainder were uncontrolled cohort studies.

#### Funding

Of the 83 studies that reported funding information, 68 had no tobacco or EC industry funding or support. Below, we detail the industry funding from the 15 studies that report tobacco or EC industry funding or support. An asterisk (*) has been added after the study name for all studies that received tobacco or EC industry funding. Where these studies contributed to meta‐analyses, we tested whether results were sensitive to their inclusion, and took account of this in our results and conclusions.

Fifteen of the included studies reported support from the tobacco or vaping industry, or that authors had received tobacco or vaping industry support outside of the study being conducted. Six of these studies received funding from the Lega Italiana AntiFumo (Caponnetto 2013a*; Caponnetto 2013b*; Caponnetto 2021*; Polosa 2011*; Polosa 2014b*; Polosa 2015*). Caponnetto 2013b* and Polosa 2011* also received free “Categoria” EC kits from the Arbi Group Srl (Milano, Italy). Caponnetto 2021* received free JUUL EC from the manufacturer, PAX Labs (became JUUL Labs in 2017). Altria Group (formerly, Philip Morris Companies) acquired a 35% stake in JUUL Labs on 20 December 2018; the study was completed before Altria invested in JUUL. Polosa 2014b* thank FlavourArt (Oleggio, NO, Italy; www.flavourart.it), an EC flavour company.

Caponnetto 2023* was funded by Philip Morris Product Société Anonyme.

Edmiston 2022* was funded by Altria Client Services LLC. Altria is the parent company of Philip Morris USA (producer of Marlboro cigarettes), John Middleton, Inc., U.S. Smokeless Tobacco Company, Inc., and Philip Morris Capital Corporation.

Kanobe 2022* was funded by RAI Services Company. The parent company is Reynolds American. Reynolds American manufacture and market a variety of tobacco products, including cigarettes (Newport, Camel, Pall Mall, Kent, Doral, Misty, Capri, and Natural American Spirit brands), EC (Vuse brand), and moist snuff (Grizzly and Kodiak brands).

Morris 2022* was funded entirely by Fontem US LLC, a subsidiary of Imperial Brands PLC.

Nides 2014* was funded by NJOY, Inc., Scottsdale, AZ. Funded by the EC/alternative nicotine products industry.

Rose 2023* was funded by the National Institute on Drug Abuse (NIDA). However, the lead author declared research support from Foundation for a Smoke‐Free World (which has links to the tobacco industry), Philip Morris International, Altria, Embera Neuro Therapeutics, Inc., Otsuka Pharmaceutical, JUUL Labs, consulting with Revive pharmaceuticals, and consulting and patent purchase agreements with Philip Morris International.

Van Staden 2013* was funded by eGo e‐cigarette packs by Twisp.

Walele 2018* was funded and supported by Fontem Ventures B.V. Imperial Brands plc (Imperial Tobacco plc) is the parent company of Fontem Ventures B.V., the manufacturer of the EC prototype used in their study.

Xu 2023* was funded by Juul Labs, Inc.

#### Excluded studies

We list 32 studies excluded at full‐text stage, along with reasons for exclusion, in the Characteristics of excluded studies table. After the reference being a duplicate, the most common reason for exclusion was that studies did not include outcomes relevant to this review.

### Risk of bias in included studies

Overall, we judged 10 studies to be at low risk of bias (Bullen 2013; Cobb 2021; Eisenberg 2020; Hajek 2019; Hajek 2022; Kerr 2020; Lee 2018; Lee 2019; Martinez 2021; Myers‐Smith 2022), 19 to be at unclear risk, and the remaining 61 at high risk of bias (this includes the non‐randomized studies, which we deemed to be at high risk due to lack of randomization).

Details of the risk of bias judgements for each domain of each included study can be found in the Characteristics of included studies table. Figure 2 and Figure 3 illustrate our judgements across the included studies.

**2:**
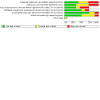
Risk of bias graph

**3:**
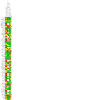
Risk of bias summary

#### Allocation

We judged 28 studies to be at high risk of selection bias; for the majority of cases, this is because the study was not randomized. We rated a pilot cluster‐randomized trial to be at high risk, as randomization was not carried out as intended for pragmatic reasons (Dawkins 2020). We judged 31 studies to be at low risk of selection bias, and the remainder to be at unclear risk as there was insufficient information with which to judge.

#### Blinding

In all, we assessed 65 studies for performance bias and detection bias. For performance bias, we rated 31 to be at low risk, 24 at high risk, and 10 at unclear risk. For detection bias, we rated 46 as low risk, 11 as high risk, and eight at unclear risk.

In these studies, blinding was not used and different levels of support were provided; this alone, or in conjunction with the outcome measures being used (subjective rather than objective measures), meant that we thought there was a high risk of bias being introduced. We judged the rest to be at unclear risk, or ineligible for this domain due to single‐arm design.

#### Incomplete outcome data

We judged most studies (66 out of 90) to be at low risk of attrition bias. We rated 10 studies with substantial loss to follow‐up as being at high risk of attrition bias. The remainder did not provide sufficient data on which to judge, and hence we judged them to be at unclear risk.

#### Selective reporting

Of the 90 studies, we considered 46 at low risk of reporting bias, as all prespecified or expected outcomes were reported. We rated 10 as being at high risk, as data were not available as specified in the original protocols (note in some cases these are recent studies, and judgement on these may change as more publications emerge). We judged the rest to be at unclear risk, due to insufficient information with which to make a judgement.

#### Other potential sources of bias

We considered Ioakeimidis 2018 to be at high risk of other bias; data were from a conference poster and the associated abstract, and quit rates in the intervention arm differed between the two sources. We considered four further studies to be at unclear risk in this domain.

### Effects of interventions

See: Table 1; Table 2; Table 3

Data on our outcomes of interest are summarized below and in our Table 1, Table 2, and Table 3. Due to the volume of data available, some relevant information is hosted on a companion repository; these data are open‐access and can be found at http://dx.doi.org/10.5287/ora-jnvxbp7qr. They are referred to below as supplemental tables. Forest plots are available through 'analysis' links; for some outcomes, benefit is plotted on the right; for others it is plotted on the left. This is due to direction of effect, e.g. an increase in cessation is a benefit, whereas an increase in a carcinogen is not.

#### Direct comparisons between nicotine EC and other pharmacotherapies

Comparisons reported here include nicotine ECs versus NRT, and nicotine ECs versus varenicline. Only randomized controlled trials contributed data.

##### Cessation

Pooled data from seven studies (two cartridges, four refillable, one pod), five of which were rated at low risk of bias (Bullen 2013; Hajek 2019; Hajek 2022; Lee 2018; Myers‐Smith 2022) and two as unclear (Klonizakis 2022; Russell 2021), showed increased quit rates in people randomized to nicotine EC when compared to NRT (risk ratio (RR) 1.59, 95% confidence interval (CI) 1.30 to 1.93; I^2^ = 0%; 2544 participants; Analysis 1.1). The certainty of evidence is high and has not been downgraded. One study included in this analysis, Hajek 2022, was conducted in pregnant women. There was no evidence of a subgroup difference between this study and studies in participants not selected on the basis of pregnancy (P = 0.87, I^2^ for subgroup differences = 0%). Follow‐up time was based on end of pregnancy, and our analysis included only those participants with follow‐up of at least six months. Results were not sensitive to the exclusion of the one study that received industry funding (Russell 2021); when this study was removed, the point estimate increased to 1.72, but the CIs remained consistent with those from the main analysis.

One study, Ioakeimidis 2018, available as a conference presentation only and considered at high risk of bias due to inconsistencies in the data reported and an unclear definition of abstinence, favoured varenicline for quitting compared to nicotine EC (cartridge) (RR 0.31, 95% CI 0.11 to 0.82; 54 participants; Analysis 2.1).

##### Adverse events

Pooled data from five studies (four considered at low risk of bias (Bullen 2013; Hajek 2022; Lee 2018; Myers‐Smith 2022) and one at unclear risk (Wagener 2023)) showed that there is probably no difference in the number of participants reporting adverse events (AEs) between nicotine EC and NRT arms (RR 1.03, 95% CI 0.91 to 1.17; I^2^ = 0%; 2052 participants; Analysis 1.2). The certainty of evidence is moderate, downgraded one level due to imprecision; the CIs were consistent with benefit and harm. None of the studies contributing data to this analysis received funding from the vaping or tobacco industries.

Hajek 2019 and Bonafont Reyes 2022 did not contribute data to this analysis due to the way in which events were recorded. In Hajek 2019's prespecified adverse reactions of interest, nausea was more frequent in the NRT group, throat/mouth irritation was more frequent in the nicotine EC group, and there was little difference in other reactions (see Supplemental Table 1 for more detail). Bonafont Reyes 2022 recruited participants with chronic obstructive pulmonary disease (COPD) and reported "a trend towards decreased dyspnoea and COPD symptoms ... in the EC arm compared to the NRT arm", but did not provide further detail.

In Ioakeimidis 2018, reports of sleep disorders were evenly distributed between groups, and nausea was more common in the varenicline arm than in the nicotine EC arm (see Supplemental Table 1 for more detail).

##### Serious adverse events

Six studies (five at low risk of bias (Bullen 2013; Hajek 2019; Hajek 2022; Lee 2018; Myers‐Smith 2022) and one at unclear risk (Wagener 2023)) comparing nicotine ECs with NRT provided data on serious adverse events. In some studies, no events occurred. Pooled results showed that there may be a slight increase in serious adverse events in the nicotine EC arms compared to NRT. There is low certainty of evidence for this outcome, downgraded two levels due to imprecision; there were fewer than 300 events and wide CIs incorporating no difference, as well as clinically significant harm and clinically significant benefit (RR 1.20, 95% CI 0.90 to 1.60; I^2^ = 32%; 2761 participants; Analysis 1.3). None of the studies contributing data to this analysis received funding from the vaping or tobacco industries. In Hajek 2022 (conducted in pregnant women), the authors reported no evidence of a difference in birth outcomes overall. However, low birthweight (< 2500 g) was less frequent in the EC than the NRT arm (14.8% versus 9.6%; RR 0.65, 95% CI 0.47 to 0.90).

No SAEs occurred in Ioakeimidis 2018 (Analysis 2.2).

##### Carbon monoxide (CO)

Pooled data from four studies (Hatsukami 2020; Kerr 2020; Klonizakis 2022; Lee 2018), none of which received tobacco/vaping industry funding and none of which were considered at high risk of bias, comparing nicotine EC with NRT, found some evidence that CO levels decreased more in those randomized to nicotine EC; however, the CIs incorporated the possibility of no between‐group difference (mean difference (MD) ‐1.81 ppm, 95% CI ‐3.64 to 0.01; I^2^ = 0%; 357 participants; Analysis 1.4). A fourth, small study (Eisenhofer 2015; n = 11) was reported as a conference abstract and hence had limited data available. At three weeks, this study showed that both EC and NRT groups had "significantly reduced" CO, but between‐group differences were not reported.

##### Heart rate, blood pressure, and oxygen saturation

Pooled data from two studies (166 participants; one study judged to be at unclear risk of bias (Hatsukami 2020) and one at low risk (Kerr 2020), neither in receipt of vaping/tobacco industry funding) showed no clear evidence of a clinically meaningful difference in heart rate (MD 0.53 bpm, 95% CI ‐1.76 to 2.83; I^2^ = 0%; Analysis 1.5), systolic blood pressure (MD ‐1.62, 95% CI ‐3.59 to 0.36; I^2^ = 0%; Analysis 1.6), or blood oxygen saturation (MD ‐0.14, 95% CI ‐0.59 to 0.30; I^2^ = 0%; Analysis 1.7), although confidence intervals were wide.

##### Toxicants

Only Hatsukami 2020 (unclear risk of bias, no tobacco/vaping industry funding, n = 111) contributed data for these outcomes. For 3‐HPMA, 2‐HPMA, and HMPMA, point estimates favoured EC but CIs included no difference (Analysis 1.8; Analysis 1.10; Analysis 1.11). There was no evidence of a difference for NNAL (nitrosamine 4‐(methylnitrosamino)‐1‐(3‐pyridyl)‐1‐ butanol) but CIs were again wide (Analysis 1.9). For PheT, CEMA, and AAMA (Analysis 1.12; Analysis 1.13; Analysis 1.14), point estimates favoured NRT but CIs included no difference.

##### Lung function

Lee 2018 and Kerr 2020 (no tobacco/vaping industry funding) measured change in FEV1 (forced expiratory volume) and FEV1/FVC (forced vital capacity) (both low risk of bias; n = 81). High statistical heterogeneity (I^2^ = 89%) precluded pooling for FEV1 (Analysis 1.15). The point estimate for Lee 2018 favoured EC and for Kerr 2020 favoured NRT; for Kerr 2020, the CIs included no difference. There was no evidence of a difference for FEV1/FVC, but there was moderate unexplained statistical heterogeneity and, again, CIs were wide (MD ‐0.16%, 95% CI ‐1.83 to 1.50; I^2^ = 51%; Analysis 1.16). For PEF (peak expiratory flow) only one study contributed to this analysis (Kerr 2020, n = 55). The point estimate favoured NRT but CIs were wide and included no difference (MD ‐3.00, 95% CI ‐27.09 to 21.09) (Analysis 1.17).

##### Study product use

Five studies (one, Russell 2021, with vaping industry funding) reported study product use at six months or longer, but statistical heterogeneity precluded pooling (I^2^ = 95%). Whereas Russell 2021 and Lee 2018 found no difference between the EC and NRT arms, in the other three studies, people in the EC arm were more likely to continue to use the study product (EC) than those in the NRT arm (Analysis 1.18). A companion publication explored long‐term rates in more detail (Butler 2022).

#### Nicotine EC versus other tobacco/nicotine products used for stopping combustible tobacco use

Currently, the only comparison reported here is nicotine EC compared to heated tobacco. One study (Caponnetto 2023*; n = 220, tobacco/vaping industry‐funded), considered at high risk of bias due to a lack of blinding alongside strong participant product preferences, reported on AEs, SAEs, expired carbon monoxide (eCO), and VO_2_ max as a measure of lung function, heart rate, and blood pressure at 12 weeks follow‐up. The effect estimate demonstrated no clear evidence of a difference in AEs between the nicotine EC and heated tobacco group (RR 0.86, 95% CI 0.68 to 1.10; 220 participants; Analysis 3.1). There were no SAEs reported in either arm, so an effect estimate could not be calculated for this outcome (Analysis 3.2). There was no clear evidence of a between‐group difference in eCO levels (MD 1.90, 95% CI ‐0.71 to 4.51; 217 participants; Analysis 3.3), or VO_2_ max (MD 6.20, 95% CI ‐2.01 to 14.41; 211 participants; Analysis 3.4).

The following was reported on heart rate and blood pressure and is reported in Supplemental Tables 4 & 5: “No significant changes in the mean resting heart rate, blood pressure, and BMI during product use were observed between and within study groups.”

#### Nicotine EC alone or versus control

Comparisons reported here include nicotine EC versus non‐nicotine EC, and nicotine EC compared to behavioural support only or to no support. In this section, we also reported results from studies in which all participants received nicotine EC (cohort studies and randomized studies that did not differ across arms in EC provision, device generation, or nicotine content).

##### Cessation

###### Randomized controlled trials

At six months or longer, quit rates were higher in nicotine EC groups than in comparator groups. Compared to EC without nicotine (placebo EC), pooled results showed nicotine EC probably produced higher quit rates (RR 1.46, 95% CI 1.09 to 1.96; I^2^ = 4%; 5 studies of cartridge and 1 study of refillable devices, 1613 participants; Analysis 4.1). There is moderate‐certainty evidence that nicotine EC probably increases quit rates compared to non‐nicotine EC. The certainty has been downgraded one level due to imprecision; there are fewer than 300 events overall. This has not been downgraded for risk of bias: removing the one study considered at high risk of bias increased the direction of the effect in favour of nicotine EC. The interpretation of the effect remained the same when we removed the one study at high risk of bias (Lucchiari 2022) and when we removed the one study with tobacco/vaping industry funding (Caponnetto 2013a*). The effect may be more pronounced when comparing nicotine EC to behavioural support only or to no support (RR 1.96, 95% CI 1.66 to 2.32; I^2^ = 0%; 11 studies (5 refillable, 3 cartridge, 3 pod), 6819 participants; Analysis 5.1). As this involved unblinded comparisons with unequal levels of support, we judged all data contributing to this outcome to be at high risk of bias (the certainty of the evidence was low, downgraded two levels). One of the studies contributing data to this comparison reported tobacco/vaping industry funding (Xu 2023*). The removal of this study in a sensitivity analysis did not change the interpretation of the effect (Table 4).

**1:** Sensitivity analysis for all studies

**Analysis number**	**Sensitivity analysis removing studies at high risk of bias**	**Sensitivity analysis removing industry‐funded studies**
1.1‐1.18	N/A 1	N/A 1
2.1‐2.2	N/A 2	N/A 1
3.1‐3.4	N/A 1	N/A 2
4.1	RR 1.61 (1.15, 2.26) I^2^ = 9%	RR 1.34 (0.98, 1.82) I^2^ = 0%
4.2	N/A 1	RR 1.01 (0.91, 1.11) I^2^ = 0%
4.3	RR 1.00 (0.56, 1.79) I^2^ = 0%	RR 0.95 (0.52, 1.72) I^2^ = 0%
4.4	N/A 3	NA 3
4.5‐4.9	N/A 1	N/A 2
4.10	N/A 1	MD ‐0.03 (‐0.46, 0.40)
4.11	N/A 1	N/A 1
5.1	RR 1.83 (1.53, 2.18), I^2^ = 0%	RR 4.73 (0.56, 39.88) I^2^ not estimable
5.2	RR 1.29 (1.15, 1.45) I^2^ not estimable	RR 1.19 (1.05, 1.30) I^2^ = 8%
5.3	N/A 2	RR 0.90 (0.65, 4.70) I^2^ = 0%
5.4	N/A 3	N/A 1
5.5	N/A 1	N/A 1
5.6	MD 1.11 (‐3.95, 6.18) I^2^ = 0%	N/A 1
5.7	N/A 1	N/A 1
5.8	ST MD ‐0.30 (‐0.74, 0.13)	ST MD ‐0.30 (‐0.74, 0.13)
5.9	ST MD 0.00 (‐0.44, 0.44)/N/A no overall value	N/A 3
5.10‐5.14	N/A 1	N/A 1
5.15	N/A 2	N/A 2
5.16	N/A 2	N/A 2
5.17	N/A 2	MD ‐0.14 (‐0.28, 0.00) I^2^ not estimable
5.18‐5.19	N/A 2	N/A 2
6.1	N/A 1	N/A 1
6.2	N/A 2	N/A 2
6.3	N/A 1	N/A the 1 study that is industry‐funded does not contribute to the RR
6.4	MD ‐0.90 (‐1.70, ‐0.10) I^2^ = 0%	MD ‐1.15 (‐2.05, ‐0.24) I^2^ = 0%
6.5‐6.10	N/A 1	N/A 2
6.11‐6.12	N/A 1	N/A 1
7.1‐7.2	N/A 2	N/A 2
8.1‐8.4	N/A 2	N/A 2
9.1	N/A 2	N/A 1
10.1‐10.2	N/A 1	N/A 1
11.1	RR 2.86 (0.30, 27.10) I^2^ not estimable	N/A 1
11.2	N/A 1	N/A 1
11.3	1.19 (0.33, 4.33) No change as the study at high risk of bias was not estimable	N/A 1
12.1‐12.3	N/A 2	N/A 1
13.1‐13.4	N/A 1	N/A 1
14.1	RR 1.04 (0.89, 1.22) I^2^ not estimable	N/A 1
14.2‐14.3	N/A 1	N/A 1
14.4	N/A 2	N/A 1
15.1	N/A 2	N/A 1
15.2	RR 1.25 (0.78, 1.99) I^2^ not estimable	RR 1.09 (0.90, 1.31) I^2^ not estimable
15.3	RR 0.59 (0.11, 3.34) I^2^ not estimable	RR 0.67 (0.37, 1.19) I^2^ not estimable
15.4	MD ‐9.10 (‐15.83, ‐2.37) I^2^ not estimable	MD ‐1.40 (‐4.26, 1.46) I^2^ not estimable
15.5‐15.8	N/A 2	N/A 1
16.1	RR 3.85 (1.91, 7.74) I^2^ not estimable	N/A 1
16.2‐16.3	N/A 2	N/A 1
17.1‐2	N/A 2	N/A 1

N/A 1 = no studies at high risk of bias/no industry‐funded studies N/A 2 = all studies at high risk of bias/industry‐funded N/A 3 = results not pooled MD: mean difference; RR: risk ratio; ST MD: standardized mean difference

Pulvers 2020 (pod device) measured cessation at six months in the intervention group only, using self‐report. As they did not measure cessation at six months in the comparator group, we could not include these data in our meta‐analysis. At six months, 23 (24%) intervention participants were exclusively using EC and 10 (10.4%) reported using neither EC nor combustible cigarettes (making a combined quit rate of 34.4% in the intervention arm at six months).

###### Data from other studies

Ten studies provided all participants with nicotine EC and assessed abstinence at six months or longer (Table 5; 2 refillable, 6 cartridges, 1 pod, 1 not specified). The highest proportion of quitters at six months was observed in Ely 2013 (cartridge), in which all participants (n = 48) used EC and 18 used additional pharmacotherapy; 44% of participants were abstinent at six months. The lowest quit rates were seen in Caponnetto 2013b*, where 14% of participants were abstinent at 12 months, and Price 2022, where 5% of participants were abstinent at 12 months. In the former, participants were unmotivated to quit smoking and, in the latter, motivation was unclear and participants were recruited from a socially deprived area on the basis of receiving a free nicotine EC.

**2:** Summary of proportion of participants abstinent from smoking at 6+ months follow‐up: cohort studies of nicotine EC

**Study**	**Motivated or unmotivated to quit smoking?**	**% abstinent**				
**Cohort studies**		**6‐month**	**12‐month**	**18‐month**	**24‐month**	**Notes**
**Adriaens 2014****^a^**	Unmotivated to quit	19.6% (10/51)	‐	‐	‐	Data from 8‐month follow‐up
**Edwards 2023**	"Willing to attempt to quit"	26.6% (8/30)	‐	‐	‐	‐
Caponnetto 2013b*	Unmotivated to quit	‐	14% (2/14)	‐	‐	‐
Caponnetto 2021*	Unmotivated to quit	35% (14/40)	‐	‐	‐	‐
**Ely 2013****^b^**	Motivated to quit	44% (21/48)	‐	‐	‐	‐
**Pacifici 2015**	Unmotivated to quit	‐	53% (18/34)	‐	‐	‐
Polosa 2011*	Unmotivated to quit	23% (9/40)	‐	15% (6/40)	13% (5/40)	‐
Polosa 2014b*	Unmotivated to quit	36% (18/50)	‐	‐	‐	‐
Polosa 2015*	Not defined	42% (30/71)	41% (29/71)	‐	‐	‐
**Price 2022**	Not defined	‐	5% (42/871)	‐	‐	‐

^a^Technically an RCT but observational for purposes of EC analysis. ^b^All participants (N = 48) used an EC, but 16 also used bupropion and 2 used varenicline.

##### Adverse events

###### Randomized controlled trials

Pooled data from five studies (none at high risk of bias, one reporting tobacco/vaping industry funding) showed that there is probably no difference in the number of participants experiencing adverse events when comparing nicotine EC to non‐nicotine EC (RR 1.01, 95% CI 0.91 to 1.11; I^2^ = 0%; 840 participants; Analysis 4.2); this is moderate‐certainty evidence, downgraded one level due to imprecision (fewer than 300 events overall). Removing the one study linked to industry funding had no effect on the interpretation of the result (Table 4). When comparing nicotine EC to behavioural support only or to no support, evidence suggests more people in the groups randomized to nicotine EC experience adverse events (RR 1.18, 95% CI 1.10 to 1.27; I^2^ = 6%; 6 studies, 2351 participants; Analysis 5.2). As this involved unblinded comparisons with unequal levels of support, we judged all data contributing to this outcome to be at high risk of bias (low‐certainty evidence downgraded two levels due to risk of bias). Interpretation of the outcome was not sensitive to the inclusion of the one study with tobacco/vaping industry support (Walele 2018*).

A further 11 randomized controlled trials provided adverse event or related data for this comparison, but could not be included in the meta‐analysis due to the way in which data were presented (see Supplemental Table 1). In the studies comparing nicotine EC to non‐nicotine EC, one found similar event rates across arms (Caponnetto 2013a*), and two reported more events in the nicotine EC arms (Felicione 2019; Tseng 2016). In a further study comparing nicotine to non‐nicotine EC, events were reported by type, with an increase in some seen in the nicotine group and an increase in others seen in the non‐nicotine group (Lucchiari 2022). In the seven studies comparing nicotine EC to behavioural support only or traditional cigarettes, Kumral 2016 found an increase in sinonasal symptoms in the group receiving nicotine EC compared to behavioural support only, and Ozga‐Hess 2019 found that throat irritation, cough, and dry mouth increased in the e‐cigarette group relative to the traditional cigarette group. By contrast, Pulvers 2020 found a reduction in respiratory symptoms in the EC compared to the traditional cigarette group, and Pope 2024 found no clear difference in the number of participants reporting dry cough and throat and mouth irritation between the EC arm and the referral information arm. Begh 2021 found an increase in throat irritation, palpitations, and dizziness in the EC group, but decreases in cough, headache, nausea, dry mouth, shortness of breath, and stomach pain. Edmiston 2022* did not break down AEs by group but reported that three participants experienced a non‐serious adverse event definitely related to the study product. Pratt 2022 reported no statistically significant between‐group difference in AEs.

###### Data from other studies

Eighteen studies provided all participants with nicotine EC and assessed adverse events at one week or longer and one RCT reported AEs reported in the nicotine EC group only (see Supplemental Table 1). In the eight studies that tracked event rates over time, six showed adverse events reducing over time (Caponnetto 2013b*; Edwards 2023; Goniewicz 2017; Polosa 2011*; Polosa 2014b*; Pratt 2016). Hickling 2019 showed no change. The most commonly reported adverse events were throat/mouth irritation, headache, cough, and nausea.

##### Serious adverse events

###### Randomized controlled trials

Nine studies compared nicotine EC with non‐nicotine EC and reported data on SAEs; in five of these (including one tobacco/vaping industry study, Caponnetto 2013a*), no events occurred, so results could not contribute to the meta‐analysis, although they are included in the forest plots for descriptive purposes. In the four studies (three low risk of bias, one unclear) where events occurred, there may be little to no difference between groups, but CIs were wide (RR 1.00, 95% CI 0.56 to 1.79; I^2^ = 0%; 1412 participants; Analysis 4.3). The evidence was of low certainty; this was downgraded two levels due to imprecision: the confidence intervals encompassed clinically significant harm as well as clinically significant benefit, and there were fewer than 300 events overall. One of these studies had links to industry funding (Rose 2023*); removing it from the analysis changed the effect estimate to 0.95 but the 95% CI remained wide (0.52 to 1.72) and so the interpretation of the result remained the same.

Twelve studies compared nicotine EC with behavioural support only or no support and reported data on SAEs; in five of these, no events occurred. Pooled results from the seven studies in which events occurred showed very uncertain evidence about the difference between arms, but CIs were wide (RR 0.93, 95% CI 0.68 to 1.28; I^2^ = 0%; 4561 participants; Analysis 5.3). Here the certainty of evidence was very low; this was downgraded two levels due to risk of bias (lack of blinding and differential support between arms, judged to be at high risk of bias). Removing the one study with tobacco/vaping industry support did not affect the interpretation of the results (Walele 2018*).

In a study in people experiencing homelessness (Dawkins 2020), SAEs were not reported, but authors reported that four to seven participants in the usual care arm and five to seven participants in the nicotine EC arm visited Accident & Emergency services at a hospital. The authors reported that these visits were unrelated to study treatment and were assessed to gather data for future economic evaluation. Further detail can be found in Supplemental Table 2.

###### Data from other studies

Nine studies provided all participants with nicotine EC and reported SAEs at a week or longer (Supplemental Table 2). In six of these, authors reported that no SAEs occurred (Caponnetto 2013b*; Caponnetto 2021*; Edwards 2023; Humair 2014; Kanobe 2022*; Polosa 2011*; Valentine 2018). In NCT02648178 (19 participants), one death occurred (no further detail provided). Hickling 2019 (50 participants) recruited participants from mental health settings; five SAEs were recorded during the study, all of which were psychiatric hospitalizations. None were considered related to study treatment.

##### Carbon monoxide

###### Randomized controlled trials

High statistical heterogeneity (I^2^ = 80%) precluded pooling CO data from the six trials (n = 677, none considered at high risk of bias) comparing nicotine EC with non‐nicotine EC (Analysis 4.4). Point estimates from four studies (one reporting links to industry funding; Rose 2023*) favoured nicotine EC and from two (one reporting industry funding; Caponnetto 2013a*) favoured non‐nicotine EC, but in all cases CIs were consistent with no clinically meaningful difference. Three further randomized studies measured CO levels in those assigned to nicotine EC and those assigned to non‐nicotine EC, but did not present data in a way that could be pooled: George 2019 did not compare data by group; Tseng 2016 reported no between‐group differences; and Meier 2017 found a slightly higher CO reading in those using nicotine EC, but the clinical and statistical significance of this difference was not clear (see Supplemental Table 3 for more detail). These data were from all study participants based on group randomized, not on subsequent EC or cigarette use.

Pooled data from 11 studies comparing nicotine EC to behavioural support alone or to no support resulted in a high I^2^ value (89%); thus, pooled results were not presented here (see Analysis 5.4 for individual study data). None of these studies reported tobacco/vaping industry funding. A funnel plot did not show asymmetry (Figure 4). Heterogeneity was primarily driven by magnitude rather than direction of effect, with results in 10 of 11 studies favouring nicotine EC. Three further trials reported data that could not be included in a meta‐analysis. Walele 2018* compared nicotine EC to cigarettes and found that CO levels declined in the EC group and remained similar to baseline in the cigarette group. Czoli 2019 instructed baseline dual users to spend periods only using EC or only using traditional cigarettes; CO measured during sole EC use was lower than baseline and lower than during cigarette‐only periods. Further details can be found in Supplemental Table 3.

**4:**
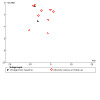
Funnel plot. Comparison: Nicotine EC vs behavioural/no support. Outcome: Carbon monoxide (ppm)

###### Data from other studies

Twenty studies provided all participants with nicotine EC and reported data on CO at one week or longer. In the 19 studies that presented change over time, CO declined from baseline, although in Ikonomidis 2018 CO levels were equivalent to baseline again at 24 weeks, and in Polosa 2014b* a decline was observed in people who quit smoking or reduced cigarette consumption by at least half, but not in those who continued smoking at least half as many cigarettes as they had from baseline.

##### Heart rate

###### Randomized controlled trials

One RCT (Caponnetto 2013a*, unclear risk of bias, n = 141) provided data on heart rate and compared nicotine EC with non‐nicotine EC; there was no evidence of a clinically significant between‐group difference (MD ‐2.3, 95% CI ‐6.10 to 1.50) (Analysis 4.5). This was comparable with findings from the one RCT (Hatsukami 2020, unclear risk of bias, n = 90) comparing nicotine EC with no pharmacotherapy, which also found no evidence of a clinically significant difference (MD 1.17, 95% CI ‐4.17 to 6.61) (Analysis 5.5).

A further three RCTs provided data on heart rate that could not be used to calculate effect estimates. George 2019 compared nicotine to non‐nicotine EC and found no difference in heart rate between arms; Walele 2018* compared a nicotine EC with a traditional cigarette and reported "no clinically significant changes", and Cobb 2021 found decreases in both the EC and QuitSmart cigarette substitute groups, with the decrease being slightly greater in the latter group. See Supplemental Table 4 for further information.

###### Data from other studies

Six studies in which all participants received a nicotine EC also reported data on heart rate; for five, changes were minimal and directions of effect were mixed, and for Caponnetto 2021* (n = 40), the rate reduced by 9 bpm at 12 weeks (see Supplemental Table 4).

##### Blood pressure

Caponnetto 2013a* found no evidence of a difference in the change in systolic blood pressure (BP) between nicotine EC and non‐nicotine EC arms (unclear risk of bias, 141 participants, MD 1.2, 95% CI ‐3.99 to 6.39; Analysis 4.6). Three studies (two at high risk of bias, one at unclear risk of bias, none reporting tobacco/vaping industry funding) compared nicotine EC to behavioural support only and reported data on systolic blood pressure; there was a small difference favouring the EC arms (MD ‐2.3, 95% CI ‐3.9 to ‐0.7, I^2^ = 24%; 298 participants; Analysis 5.6). Three further RCTs measured change in blood pressure but presented results in such a way that they could not be pooled. George 2019 compared nicotine EC and non‐nicotine EC and combined data from both groups; BP declined over time. Compared to a QuitSmart cigarette substitute, Cobb 2021 found that EC led to a greater reduction in BP. Walele 2018* found "no clinically significant changes" when comparing nicotine EC to a conventional cigarette at two weeks.

Five studies, which provided nicotine EC to all participants, reported change in blood pressure; results were clinically insignificant except for Caponnetto 2021* in which systolic BP reduced by 12 (from 134 to 122) at 12 weeks (see Supplemental Table 5 for further details on all studies reporting this outcome).

##### Oxygen saturation

Hatsukami 2020 found no evidence of a difference in blood oxygen saturation when comparing nicotine EC to cigarettes (89 participants, MD 0.20, 95% CI ‐0.30 to 0.70; Analysis 5.7). Van Staden 2013*, a short‐term pre‐post study, which measured outcomes after two weeks of EC use, found that people who smoked and switched to ECs had significant improvement in blood oxygen saturation (96.2% (standard deviation (SD) 1.8) to 97.5% (SD 1.3); 1.3% increase, 95% CI 0.6 to 2.1; P = 0.002).

##### Toxicants

Unless stated otherwise, all randomized controlled trials measuring these outcomes compared nicotine EC with no pharmacotherapy.

Two trials measured change in 3‐HPMA (one at high risk of bias). In both, the point estimate favoured the EC arm, but pooling was precluded due to differences in measurement methods (Analysis 5.8). Five further studies, in which all participants were given nicotine EC, measured 3‐HPMA; all found reductions over time (Supplemental Table 6).

Five trials measured change in NNAL and provided sufficient data to calculate summary effects (four at high risk of bias; Analysis 5.9). Three of the five studies found results favouring nicotine EC, but the final two indicated no difference; statistical heterogeneity was high (I^2^ = 96%), so pooled results were not presented. One study comparing nicotine EC to no treatment described their findings narratively and stated that "NNAL decreased more over time in the e‐cigarette group ... the e‐cigarette group had significantly lower NNAL at 4 weeks (estimate = 0.54; SE = 0.23; t = 2.37; P < 0.02), but the group difference was attenuated at 8 weeks (estimate = 0.42; SE = 0.23; t = 1.83; P < 0.07)" (Pratt 2022). Pulvers 2018 and Morris 2022*, which provided all participants with nicotine EC, found a reduction in NNAL over time and Czoli 2019, which was a cross‐over trial, found NNAL decreased when using nicotine EC compared to using traditional cigarettes (Supplemental Table 6). An additional two RCTs (one unclear and one low risk of bias; none reporting tobacco/vaping industry funding) compared nicotine EC versus non‐nicotine EC and found no evidence of difference, with wide CIs and moderate statistical heterogeneity (‐0.02 pmol/mg creatinine, 95% CI ‐0.45 to 0.41; I^2^ = 54%; 363 participants; Analysis 4.10).

One trial (n = 90, unclear risk of bias) found non‐statistically significant lower levels of 2‐HPMA, HMPMA, PhET, and AAMA in nicotine EC arms compared to control (Analysis 5.10; Analysis 5.11; Analysis 5.12; Analysis 5.14). A further two studies, in which all participants received nicotine EC, found reductions in 2‐HPMA and AAMA measures over time (Supplemental Table 6). No difference was found in the one trial (n = 90, unclear risk of bias) evaluating CEMA (Analysis 5.13).

One trial (n = 384, unclear risk of bias) found reductions in S‐PMA compared to control (MD ‐1371.00 nanograms, 95% CI ‐1995.23 to ‐746.88) (Analysis 5.15); this was consistent with the two studies in which all participants received nicotine EC that measured S‐PMA, where levels declined over time (Supplemental Table 6).

Of the 33 remaining measurements in single studies where all participants received a nicotine EC, 28 reduced over time and five increased (Supplemental Table 6).

##### Lung function

Caponnetto 2013a* measured a number of lung function parameters. FeNO increased more in the nicotine EC than the non‐nicotine EC group (MD 2.35, 95% CI 1.78 to 2.92; 90 participants; Analysis 4.7). No difference was found between nicotine and non‐nicotine EC for FEV1 or FEV1/FVC (Analysis 4.8; Analysis 4.9).

In the comparison of nicotine EC to behavioural support only/no support, pooled results from two studies (both high risk of bias, both tobacco/vaping industry funded) found improvements in FEV1 but with moderate statistical heterogeneity and CIs including no difference (standardized mean difference (SMD) 0.15, 95% CI ‐0.01 to 0.31, I^2^ = 70%; 714 participants; Analysis 5.16). Pooled data from two studies (both high risk of bias, one reporting tobacco/vaping industry funding (Walele 2018*)) showed no difference in FEF (forced expiratory flow) 25‐75, with substantial levels of statistical heterogeneity (MD ‐0.06, 95% CI ‐0.18 to 0.06, I^2^ = 73%; 2 studies, 555 participants; Analysis 5.17). In a sensitivity analysis removing Walele, the result was still consistent with no difference, though the point estimate was greater in magnitude. Data from one study at high risk of bias showed no difference in PEF (peak expiratory flow 25‐75 (litres/minute)) (387 participants, MD ‐7.10, 95% CI ‐29.14 to 14.94; Analysis 5.18). The one study reporting FEV1/FVC (327 participants, MD 1.72, 95% CI 0.74 to 2.70, high risk of bias) favoured nicotine EC (Edmiston 2022*) (Analysis 5.19).

Cobb 2021, which randomized participants to EC or the QuitSmart cigarette substitute, measured change in a number of lung function parameters: direction of effect was mixed across these, with no statistically or clinically significant between‐group differences at 12 weeks (Supplemental Table 7).

Two studies, which provided all participants with nicotine EC, measured change in lung function over time: Hickling 2019 found an increase in peak flow, and Oncken 2015 found "no significant differences" in airway function (Supplemental Table 7).

##### Study product use

Three trials (all low risk), comparing nicotine EC with non‐nicotine EC, reported the number of participants still using EC at six months or longer. Slightly more participants were still using EC in the nicotine EC arms, but CIs were wide and included no difference (RR 1.15, 95% CI 0.94 to 1.41, I^2^ = 30%; 874 participants; Analysis 4.11). Data on this outcome from single‐arm studies or RCTs where a study product (i.e. EC) was only provided in one arm can be found in a companion publication (Butler 2022) and Supplemental Table 8.

#### Direct comparisons between nicotine EC

Note, studies reported in this section are only those where participants were randomized to different nicotine EC conditions.

##### Comparisons based on nicotine dose

Six trials provided data comparing different doses of nicotine in EC (although other studies provided a range of doses, these were not randomly assigned). Only one study provided data on abstinence; in Cobb 2021 (low risk of bias), quit rates were higher in the higher‐dose arm but the 95% CI included no difference (RR 2.50, 95% CI 0.80 to 7.77; 260 participants; Analysis 6.1).

Three of the four studies that provided data on adverse events and contributed to this comparison provided them in such a way that the studies could not be pooled. Kimber 2021 reported "no changes over time or differences between condition", and Pratt 2022 and Morris 2022* did not compare AEs by nicotine strength (see Supplemental Table 1). Kanobe 2022* found slightly more participants in the lower‐dose group reported AEs; however, 95% CI incorporated the null and also the possibility that more people experienced AEs in the higher‐dose arm (RR 0.90, 95% CI 0.58 to 1.40; 68 participants; Analysis 6.2).

In Caponnetto 2013a*, no serious adverse events were reported in either arm; in Cobb 2021, there were more events in the higher‐dose arm but CIs were wide (RR 1.51, 95% CI 0.51 to 4.42; 239 participants; Analysis 6.3). In Morris 2022*, no serious adverse events occurred (Supplemental Table 2).

Point estimates favoured higher‐dose EC and CIs excluded no difference for carbon monoxide and FEV1/FVC (MD ‐0.92, 95% CI ‐1.71 to ‐0.13; 348 participants, 3 studies) (Analysis 6.4), (MD 0.91, 95% CI 0.15, 1.67; 90 participants, 1 industry‐funded study) (Analysis 6.10). Interpretation of Analysis 6.4 did not change when excluding the one study with tobacco/vaping industry funding (Caponnetto 2013a*); Analysis 6.10 includes only this study. There were no clear differences between arms for heart rate, blood pressure, other lung function measures, or NNAL (Analysis 6.5; Analysis 6.6; Analysis 6.7; Analysis 6.8; Analysis 6.9; Analysis 6.11; all include only one study). More participants in the higher‐dose nicotine group were still using EC at six months or longer, but data were from one study and CIs were wide and included no difference (RR 1.27, 95% CI 0.95 to 1.68; 260 participants; Analysis 6.12). In Yingst 2020 (cross‐over, comparing different doses and different devices), exhaled CO and reported nausea did not differ between devices; self‐reported dizziness was low overall but slightly higher in the higher‐dose arm. Further details can be found in Supplemental Table 1 and Supplemental Table 3. Morris 2022* measured a range of toxicants but did not compare these based on nicotine level assignments (Supplemental Table 6).

One further study, White 2021, also included comparisons based on nicotine levels (1.8% free‐base nicotine, designated by the authors as 'moderate', and 0.3% free‐base nicotine, designated by the authors as 'low'). This was a factorial trial (unpublished at the time of writing) which, in addition to e‐liquid nicotine content, also manipulated cigarette nicotine content and e‐liquid flavour availability. The authors reported no significant main effects for nicotine content on CO or CEMA, and no statistically significant interactions for these conditions. There also appear to have been no differences in the proportions of people experiencing adverse events, but the study terminated early and was likely underpowered to detect differences.

##### Comparisons based on flavour

One study randomized participants to different flavour conditions (1. tobacco flavour only; 2. a choice of flavours) and followed up participants for six months or longer (Xu 2023*, n = 566, industry‐funded, high risk of bias). Quit rates were lower in the choice compared to the tobacco arm, but the CIs were wide and incorporated no difference and a clinically significant increase relative to tobacco flavour (choice versus tobacco, RR 0.80, 95% CI 0.54 to 1.16; Analysis 7.1). Xu 2023* also reported on product use at six months or longer; again, there was no clear evidence of a difference, but the CIs were wide (choice versus tobacco, RR 1.10, 95% CI 0.86 to 1.40; Analysis 7.2).

One study (Edmiston 2022*, n = 300, high risk of bias, vaping/tobacco industry funding) randomized participants to different flavours (tobacco versus menthol) and provided SAE data in a way that could have been used to compute risk ratios, although no SAEs occurred in either arm (Analysis 8.1). NNAL and FEV1/FVC were lower in the tobacco flavour group but CIs were wide and included no difference (MD ‐26.10, 95% CI ‐66.73 to 14.53; Analysis 8.2; MD ‐0.46, 95% CI ‐1.67 to 0.75; Analysis 8.4). There was no evidence of a difference in FEV1 (MD ‐0.67, 95% CI ‐2.34 to 1.00; Analysis 8.3). No other outcomes from this paper were eligible for inclusion in our review.

Morris 2022*, an industry‐funded, randomized, cross‐over trial, tested the effect of 10 different flavours (as well as nicotine strengths and salt versus free‐base nicotine). Only their data on AE and SAE were eligible for inclusion in our review, but analyses were not reported by flavour (Supplemental Table 1; Supplemental Table 2).

White 2021 also contributed data to this comparison, with conditions being tobacco flavours only, or tobacco, fruit, dessert, and mint flavours. No significant main effects or interactions were found for flavours on the outcomes relevant to this review, namely CO and CEMA, and no difference was discernable in the occurrence of AEs. However, as noted above, the study terminated early and hence was underpowered to detect differences.

More information on flavour choices across the studies in this review can be found in a companion publication (Lindson 2022).

##### Comparisons based on device type

Kimber 2021 (high risk of bias) is the only study to directly compare device types (cartridge versus refillable). Outcomes eligible for this review were CO and AE. There was no difference between arms for CO, but CIs were wide (MD 0.70, 95% CI ‐4.98 to 6.38; 32 participants; Analysis 9.1). The authors reported "no changes over time or differences between condition" for AEs (see Supplemental Table 1).

##### Nicotine salt versus free‐base nicotine

One study (Russell 2021, unclear risk of bias, tobacco/vaping industry funding) contributed data to this comparison. Quit rates and study product use were both similar between arms (RR 1.25, 95% CI 0.85 to 1.83, n = 285; Analysis 10.1; and RR 1.07, 95% CI 0.82 to 1.41, n = 227; Analysis 10.2, respectively).

As described above, Morris 2022* also tested salt versus free‐base nicotine, but did not provide data broken down by these characteristics for our outcomes of interest (Supplemental Table 1; Supplemental Table 2).

#### Non‐nicotine EC

Although non‐nicotine ECs serve as a 'control group' in our primary analysis, due to their behavioural properties, they can also be considered an intervention in and of themselves. Comparisons included here are: non‐nicotine EC versus NRT; non‐nicotine EC versus behavioural support/not treatment; and non‐nicotine EC as an adjunct to NRT. All contributing data were from randomized controlled trials. None of these studies reported data on change in heart rate, blood pressure, oxygen saturation, toxicants, or lung function.

##### Cessation

When comparing non‐nicotine EC to behavioural support only, pooled results from two studies (n = 388; one at high risk of bias, neither reporting tobacco/vaping industry funding) found higher quit rates in participants randomized to non‐nicotine EC, but the confidence interval included the possibility of no difference (RR 1.63, 95% CI 0.81 to 3.25; I^2^ = 0%; Analysis 11.1). When evaluating non‐nicotine EC as an adjunct to NRT, Walker 2020 also found higher quit rates in participants randomized to non‐nicotine EC, although again the confidence interval included no difference (RR 1.67 95% CI 0.50 to 5.53; 624 participants; Analysis 12.1).

Two studies (n = 314, neither at high risk of bias, neither reporting tobacco/vaping industry funding) compared non‐nicotine EC with NRT (Klonizakis 2022; Lee 2019). The pooled estimate showed no clear evidence of a difference in quit rates between the two interventions (RR 0.99, 95% CI 0.64 to 1.54; I^2^ = 36%; Analysis 13.1).

##### Adverse events

Eisenberg 2020 found a higher rate of adverse events in the non‐nicotine EC arm than in behavioural support only, with the confidence interval excluding no difference (RR 1.28, 95% CI 1.13 to 1.44; 248 participants; Analysis 11.2). Also comparing non‐nicotine EC to behavioural support, Lucchiari 2022 reported that some AEs were lower in the non‐nicotine EC arm, some higher, and others reported at similar rates to the behavioural support arm (overall AE rates were not reported) (Supplemental Table 1).

Walker 2020 found fewer adverse events in participants receiving non‐nicotine EC + NRT compared to NRT alone, with the confidence interval excluding no difference (RR 0.70, 95% CI 0.53 to 0.91; Analysis 12.2). Lee 2019 also found that fewer participants receiving non‐nicotine EC reported adverse events than those receiving NRT, with the confidence interval excluding no difference (RR 0.33, 95% CI 0.12 to 0.87; 132 participants; Analysis 13.2).

##### Serious adverse events

Two studies reported on rates of SAEs when comparing non‐nicotine EC with behavioural support. Lucchiari 2022 reported no SAEs in either arm (RR not estimable), whereas Eisenberg 2020 found a higher rate of SAEs in the non‐nicotine EC arm than in the behavioural support‐only arm. However, confidence intervals were wide and incorporated clinically significant benefit and clinically significant harm (n = 388; RR 1.19, 95% CI 0.33 to 4.33; Analysis 11.3). In Walker 2020, more SAEs occurred in the group randomized to non‐nicotine EC + NRT than in the NRT‐alone group, but the confidence interval included no difference as well as the potential for a clinically significant difference in favour of the intervention (RR 1.69, 95% CI 0.60 to 4.74; Analysis 12.3). No SAEs were reported in either arm of Lee 2019 (non‐nicotine EC versus NRT) (Analysis 13.3).

##### Carbon monoxide

One study investigating the comparison between non‐nicotine EC and NRT reported change in CO between baseline and six‐month follow‐up. The point estimate favoured NRT; however, the CI encompassed both benefit and harm of the intervention (RR 2.00, 95% CI ‐0.50 to 4.50; n = 164; Analysis 13.4).

#### Advice to use EC to quit

Three studies did not provide EC, but instead provided participants with advice on how to use EC to stop smoking; none reported tobacco/vaping industry funding. Czoli 2019 and Vickerman 2022 were short‐term studies and contributed data to supplemental tables only. However, Martinez 2021 (low risk of bias) and Elling 2023 (high risk of bias) provided sufficient data from long‐term follow‐up to include them in meta‐analysis. In both cases, people received self‐help smoking cessation interventions with information on how to use EC to quit smoking compared to a smoking cessation intervention without the recommendation to use EC. However, Martinez 2021 specifically recruited people using both combustible cigarettes and EC (dual users) at baseline and Elling 2023 only required participants to be combustible cigarette users at baseline. Pooled quit rates provided no clear evidence of a difference between the two types of intervention provided (RR 1.02, 95% CI 0.88 to 1.19; 2652 participants; Analysis 14.1). In Vickerman 2022 more AEs occurred in the group receiving advice to use EC to quit; however, confidence intervals included no difference (RR 1.27, 95% CI 0.72 to 2.26; 52 participants; Analysis 14.2). No SAEs were reported, so RRs were not estimable (Analysis 14.3). Elling 2023 and Martinez 2021 also reported on EC use at six‐month follow‐up. Data from Elling 2023 suggested higher rates of long‐term EC use in the EC advice arm; however, the 95% CI also encompassed the possibility of lower long‐term EC use in the intervention arm (RR 1.77, 95% CI 0.83 to 3.79; Analysis 14.4). Martinez 2021 reported that 64% in the targeted booklet arm, and 66% in the generic booklet arm were still using EC. The latter data could not be incorporated into a meta‐analysis due to uncertainty about the denominator used to calculate percentages.

#### Combination therapy

##### Nicotine EC and NRT

This section covers two comparisons: studies in which all arms received NRT and participants were randomized to nicotine EC or non‐nicotine EC, and studies in which all participants received NRT and one arm was randomized to nicotine EC, in addition. All studies contributing data were randomized controlled trials. No studies in this group reported data on heart rate, blood pressure, oxygen, or toxicants.

###### Cessation

Two trials (both at high risk of bias, both testing refillable devices, neither reporting tobacco/vaping industry funding) in which all participants received NRT compared nicotine EC to non‐nicotine EC. The pooled results favoured nicotine EC, with the CI excluding no difference (RR 1.77, 95% CI 1.07 to 2.94; I^2^ = 0%; 1039 participants; Analysis 15.1).

Three studies (two high risk of bias, one unclear risk; two refillable, one cartridge; none reporting tobacco/vaping industry funding) also compared nicotine EC + NRT to NRT alone. Pooling results from all three studies resulted in high statistical heterogeneity, precluding meta‐analysis (I^2^ = 83%). This heterogeneity was driven by the study of a cartridge device (RR 1.00, 95% CI 0.64 to 1.55, 1712 participants) (Morphett 2022a); historically, cartridge devices have had poorer nicotine delivery than refillables. Once this study was removed, heterogeneity disappeared (I^2^ = 0%), but only two studies remained. In these two studies, pooled results showed more people quit in the refillable nicotine EC + NRT arm than in the NRT alone arm (RR 3.53, 95% CI 1.93 to 6.44; 980 participants; Analysis 16.1). In two of these studies, participants in both groups received nicotine patches but, in Morphett 2022b, participants in the NRT‐only arm also received a short‐acting form of NRT.

###### Adverse events

Three trials in which nicotine ECs were compared to non‐nicotine ECs (both with NRT as an adjunct) reported data on AEs. Baldassarri 2018 reported results combined across groups but noted "no significant differences by treatment group" (Supplemental Table 1). Pooled data from the other two studies (one reporting tobacco/vaping industry funding; Rose 2023*) also showed no clear evidence of difference (RR 1.11, 95% CI 0.93 to 1.32, I^2^ = 0%; 677 participants; Analysis 15.2). As expected due to the low statistical heterogeneity, the two contributing study results had similar interpretations and so removing Rose 2023* due to industry funding had no impact.

The three trials comparing nicotine EC + NRT to NRT alone that contributed data to this outcome were all at high risk of bias; none reported tobacco/vaping industry funding. Pooled results showed no evidence of a difference in AEs between arms, but with moderate statistical heterogeneity (RR 0.96, 95% CI 0.83 to 1.11; I^2^ = 64%; 1984 participants; Analysis 16.2). A further trial currently has only very limited information available from a conference abstract and trial registry (NCT04084210). This study appears to have trial arms that will allow comparison between nicotine EC + NRT and NRT alone. Data on AEs are reported in the trial registry; however, the way this is currently reported makes it hard to incorporate the data into our meta‐analysis; therefore, data available thus far are reported in Supplemental Table 1.

###### Serious adverse events

Pooled data from two studies (one high risk, one unclear; one reporting tobacco/vaping industry funding) comparing nicotine EC with non‐nicotine EC as adjuncts to NRT showed fewer SAEs in the nicotine EC group than in the non‐nicotine EC group, but the CI included no difference (RR 0.66, 95% CI 0.38 to 1.14; I^2^ = 0%; 1069 participants; Analysis 15.3). Removing the study with industry funding (Rose 2023*) had no effect on interpretation.

Four studies (all high risk of bias; none reporting tobacco/vaping industry funding) provided data on SAEs and compared nicotine EC + NRT to NRT alone. The pooled estimate favoured the NRT‐alone group, but the CI was wide and included no difference (RR 1.26, 95% CI 0.46 to 3.42: I^2^ = 0; 2245 participants; Analysis 16.3).

As noted above, there is currently very limited data available for NCT04084210. SAE data available thus far are reported in Supplemental Table 2.

###### Carbon monoxide

Walker 2020 (which compared nicotine EC + NRT, non‐nicotine EC + NRT, and NRT alone) measured change in CO levels but did not report data in a way that could be pooled. CO declined over time, with the greatest reduction seen in the nicotine EC group (see Supplemental Table 3). Pooled data from two studies (one high risk of bias, one unclear; one reporting tobacco/vaping industry funding) comparing nicotine and non‐nicotine EC as adjuncts to NRT found a greater reduction in CO in the nicotine EC group, but the CI included no clear evidence of a clinically significant difference (MD ‐2.58 ppm, 95% CI ‐5.21 to 0.05, I^2^ = 77%; 70 participants; Analysis 15.4) between groups and there was substantial statistical heterogeneity. We have pooled these studies despite the high I^2^ as the individual study effects both showed a benefit of nicotine EC with the difference being in the magnitude of effect. Removing the study at high risk of bias (Baldassarri 2018) left only Rose 2023*, with the following effect estimate: RR ‐9.10, 95% CI ‐15.83 to ‐2.37; whereas removing the study with industry funding (Rose 2023*) left only Baldassarri 2018, with the following effect estimate (RR ‐1.40, 95% CI ‐4.26 to 1.46).

###### Lung function

Baldassarri 2018, which compared nicotine EC to non‐nicotine EC, in which both groups received NRT, found no between‐group differences in FeNO, FEV1, or FVC (Analysis 15.5; Analysis 15.6; Analysis 15.7); confidence intervals were wide for all outcomes.

###### Study product use

In Walker 2020, at six months, 40% of the patches‐only arm (n = 52) were still using patches and, in the patches + nicotine EC group (n = 317), 22% were using patches only, 45% were using EC only, and 11% were using both patch and EC. In the patches + non‐nicotine EC group (n = 308), 29% were still using patches, 36% were using EC only, and 13% were using both patches and EC. In Baldassarri 2018, there was no difference between arms in product use, but only nine participants contributed data (RR 1.25, 95% CI 0.29 to 5.53; Analysis 15.8).

##### Nicotine EC and varenicline

One study, Tattan‐Birch 2023 (high risk of bias, 92 participants), evaluated nicotine EC and varenicline compared to varenicline alone. The study terminated early due to varenicline supply issues (an international recall), and the only data eligible for inclusion in this review related to AEs and SAEs. There was no evidence of a difference in AEs, though CIs were wide (RR 1.18, 95% CI 0.84 to 1.67; 92 participants; Analysis 17.1), and no SAEs occurred (Analysis 17.2).

#### Results from network meta‐analyses

We used fixed‐effect models for all three outcomes. For the cessation and SAE outcomes there was no indication of heterogeneity or inconsistency. The models fit well. The difference between the mean residual deviance and the number of data points was, at most, 2.2 (see Supplemental Table 9 for comparisons across models). Node‐splitting models raised no concerns over local inconsistency, resulting in P values greater than 0.05.

For the AE outcome, there was inconsistency in the treatment effect comparing no e‐cigarettes or pharmacotherapy to non‐nicotine e‐cigarettes, as indicated by a node‐splitting model. Only one trial, Eisenberg 2020, contributed direct evidence to this comparison. In this trial, the percentage of patients experiencing at least one AE was large, at 87%. This was the highest percentage in a very wide range (9% to 87%, mean 44%, standard deviation 24%). Percentages that are close to 100 can result in large effect sizes even when the absolute differences between the treatment groups are comparatively small. We believe that the inconsistency was caused by there being only one trial providing direct evidence, which had a high level of AEs, especially in the non‐nicotine e‐cigarette arm at 93%.

Overall, results from NMAs were consistent with those from pairwise analyses. They are summarized below, and further details on all can be found in the relevant figures and supplemental files.

The comparator for all odds ratios was the reference treatment: no e‐cigarettes or pharmacotherapy.

##### Cessation

See Figure 5 for the network map of the cessation outcome.

**5:**
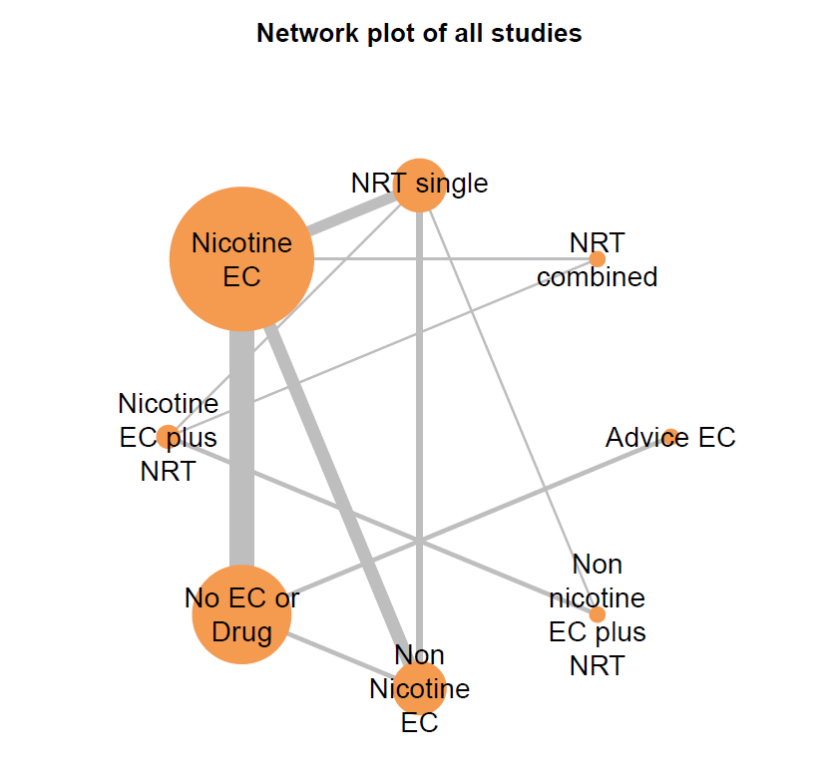
Network map for cessation

Point estimates were highest for nicotine EC combined with NRT and non‐nicotine EC combined with NRT, though 95% credibility intervals (CrI) were wide for both nodes and overlapped with those of nicotine EC. Overall, data were consistent with those from pairwise meta‐analyses, showing higher quit rates with nicotine EC than with non‐nicotine EC or NRT. Risk ratios (RRs) and corresponding credibility intervals (CrI) for each node are as follows (and illustrated in Figure 6):

**6:**
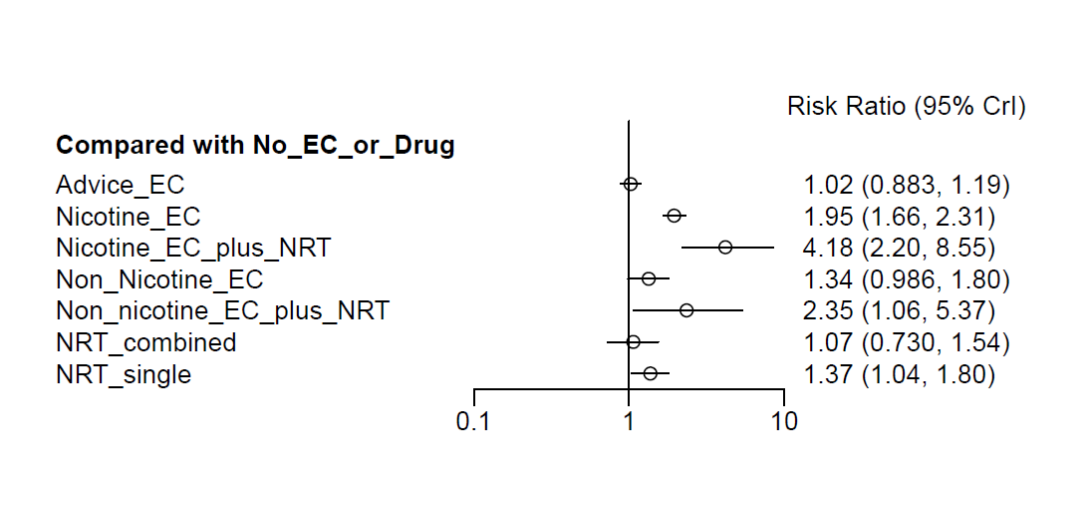
Cessation network meta‐analysis

- Nicotine EC: RR 1.95, 95% CrI 1.66 to 2.31
- Nicotine EC plus single‐form NRT: RR 4.18, 95% CrI 2.20 to 8.55
- Non‐nicotine EC: RR 1.34, 95% CrI 0.99 to 1.80
- Non‐nicotine EC plus single‐form NRT: RR 2.35, 95% CrI 1.06 to 5.37
- Combined NRT: RR 1.07, 95% CrI 0.73 to 1.54
- Single‐form NRT: RR 1.37, 95% CrI 1.04 to 1.80
- Advice on using EC: RR 1.02, 95% CrI 0.88 to 1.19

When removing studies at high risk of bias, there were insufficient data to generate a point estimate for non‐nicotine EC combined with NRT. The risk ratio of single‐form NRT against no e‐cigarettes or pharmacotherapy changed from 1.37 to 2.25, and became significant, where it had been borderline‐significant in the main analysis. All other interpretations remained unchanged (Supplemental Table 10). Results were not sensitive to the exclusion of studies with tobacco or vaping industry funding (Supplemental Table 10).

##### Adverse events

See Figure 7 for the network map of the AE outcome.

**7:**
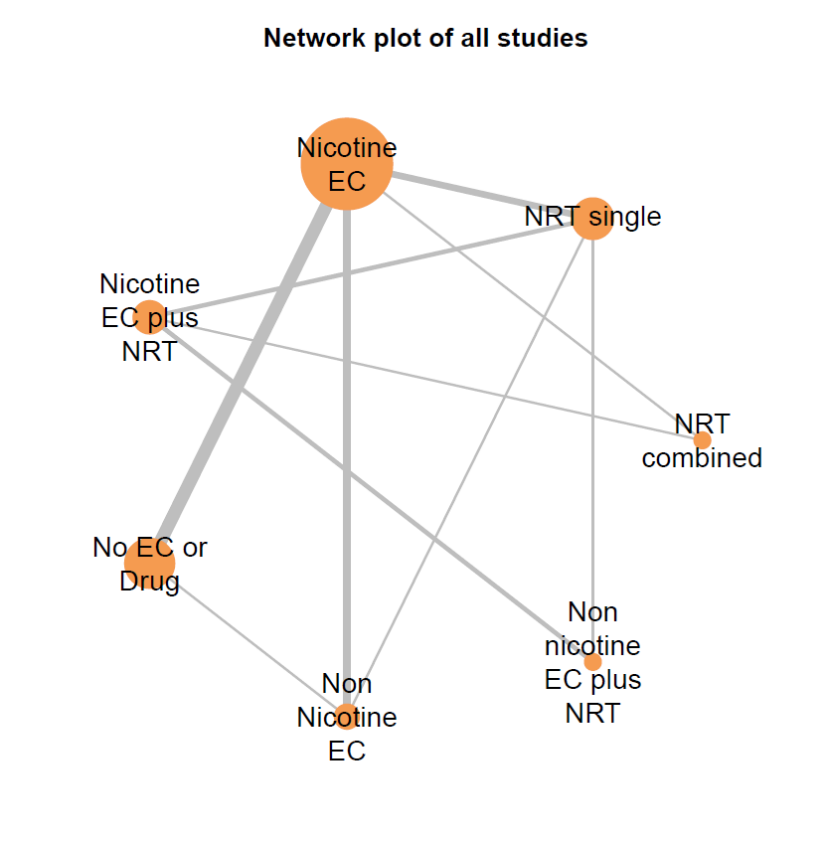
Network map for adverse events

As can be seen in Figure 8, CrIs overlapped for all nodes. All interventions tested showed an increase in AEs (non‐serious) compared to control (no e‐cigarette or pharmaceutical intervention), with CrIs excluding no difference for nicotine ECs, non‐nicotine ECs, and single‐form NRTs. The RRs and corresponding CrI for each node are as follows (and illustrated in Figure 8):

**8:**
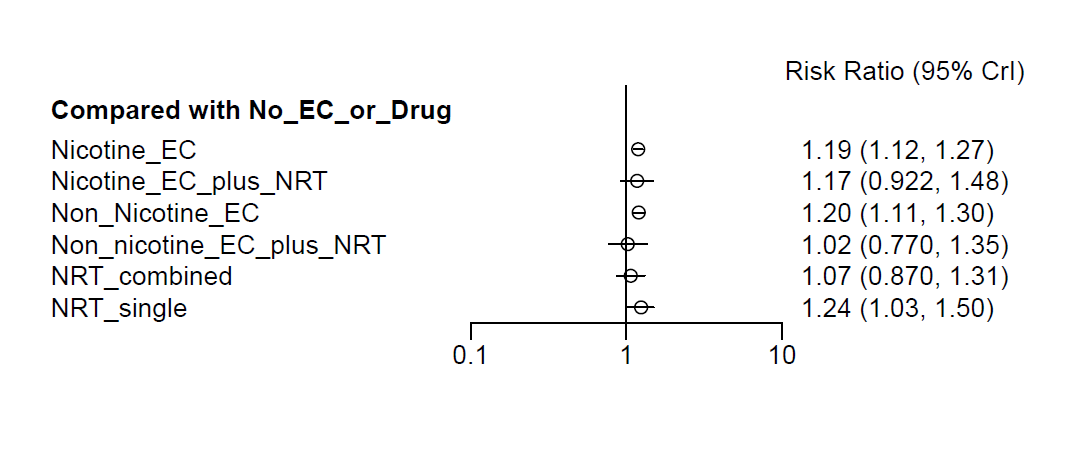
Adverse events network meta‐analysis

- Nicotine EC: RR 1.19, 95% CrI 1.12 to 1.27
- Nicotine EC plus single‐form NRT: RR 1.17, 95% CrI 0.92 to 1.48
- Non‐nicotine EC: RR 1.20, 95% CrI 1.11 to 1.30
- Non‐nicotine EC plus single‐form NRT: RR 1.02, 95% CrI 0.77 to 1.35
- Combined NRT: RR 1.07, 95% CrI 0.87 to 1.31
- Single‐form NRT: RR 1.24, 95% CrI 1.03 to 1.50

Sensitivity analyses removing studies at high risk of bias and removing studies with tobacco or vaping industry funding did not affect interpretation of the results (Supplemental Table 10).

##### Serious adverse events

See Figure 9 for the network map of the SAE outcome.

**9:**
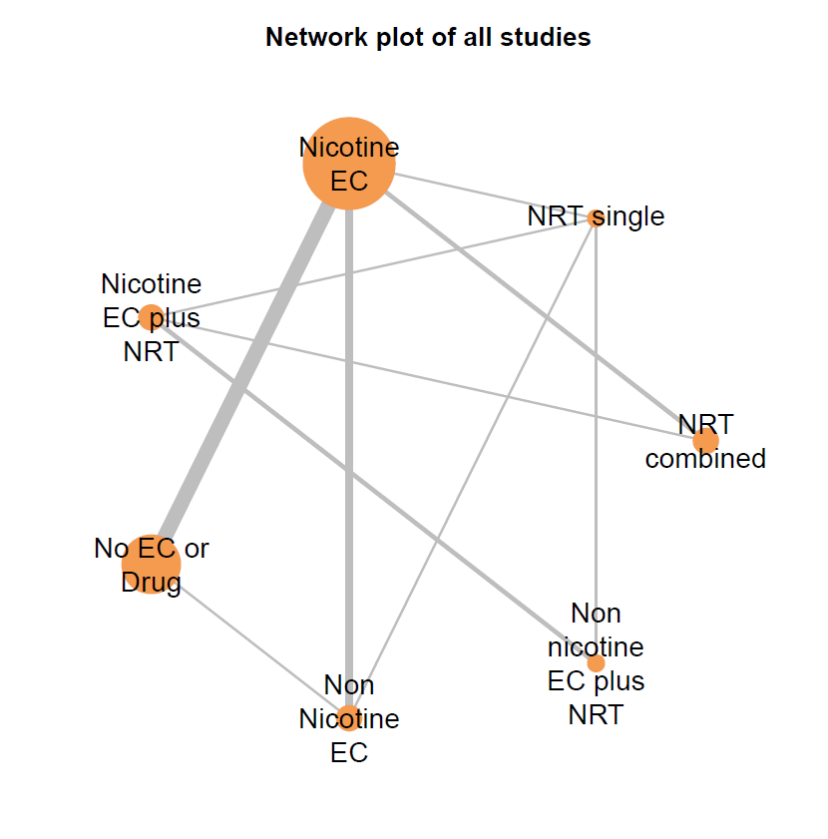
Network map for serious adverse events

As illustrated in Figure 10, CrIs were wide, overlapped, and included the possibility of no difference for all nodes, though point estimates were lowest for NRT. Only the point estimate for non‐nicotine EC combined with NRT was consistent with an increase in SAEs compared to the control condition, but here CrI were very wide and also encompassed a clinically significant reduction in SAEs in those receiving the intervention. The RRs and corresponding CrI for each node are as follows (and illustrated in Figure 10):

**10:**
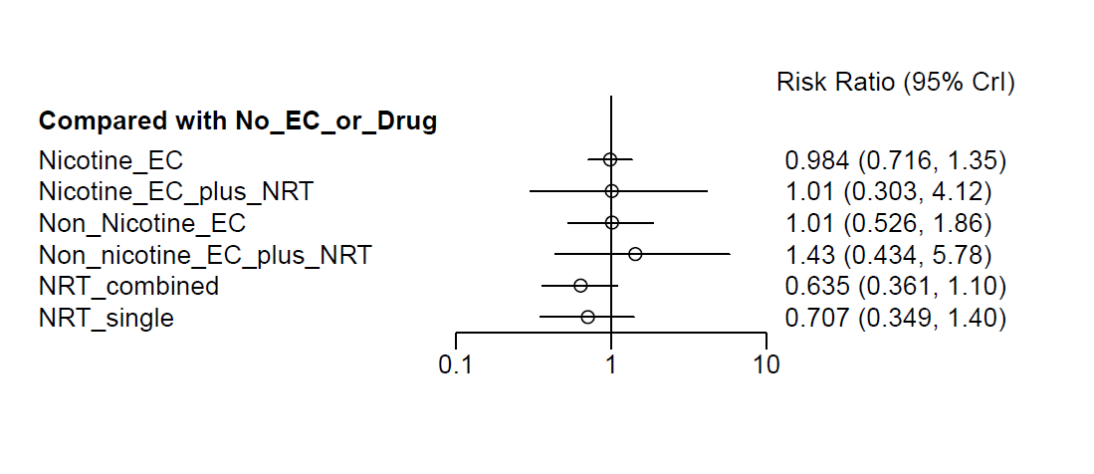
Serious adverse events network meta‐analysis

- Nicotine EC: RR 0.98, 95% CrI 0.72 to 1.35
- Nicotine EC plus single‐form NRT: RR 1.01, 95% CrI 0.30 to 4.12
- Non‐nicotine EC: RR 1.01, 95% CrI 0.53 to 1.86
- Non‐nicotine EC plus single‐form NRT: RR 1.43, 95% CrI 0.43 to 5.78
- Combined NRT: RR 0.64, 95% CrI 0.36 to 1.10
- Single‐form NRT: RR 0.71, 95% CrI 0.35 to 1.40

When removing studies at high risk of bias, only nicotine EC, non‐nicotine EC, combined NRT, and single‐form NRT had sufficient data to generate point estimates; these were consistent with those from the main model (Supplemental Table 10). When removing studies with tobacco or vaping industry funding, there was still sufficient data for all nodes to be displayed; results were consistent with those from the main analysis (Supplemental Table 10).

## Discussion

### Summary of main results

This update includes a further two studies published since the last version (January 2024). Our three main comparisons, nicotine EC compared to NRT, nicotine EC compared to non‐nicotine EC, and nicotine EC compared to behavioural support only/no support continue to show increased quit rates in people assigned to nicotine EC arms. This is high‐certainty for the comparison with NRT, moderate‐certainty for the comparison with non‐nicotine EC, and low‐certainty for the comparison with behavioural support only/no support (Table 1; Table 2; Table 3). In absolute terms, pooled data suggest an additional two to six people for every 100 would quit smoking with nicotine EC compared to NRT, an additional one to seven people for every 100 would quit smoking with nicotine EC compared to non‐nicotine EC, and an additional three to five people for every 100 would quit smoking with nicotine EC compared to behavioural support only or no support for smoking cessation. Most data come from studies of cartridges and refillable devices, although the number of studies investigating pod devices is increasing, with the two new included studies providing pod devices.

There remains moderate certainty of no evidence of a difference in rates of adverse events (AEs) with nicotine EC compared to non‐nicotine EC, and moderate certainty of no evidence of a difference in rates of AEs with nicotine EC compared to NRT. Evidence on AEs and serious adverse events (SAEs) was of low to very low certainty across all other comparisons, due to a paucity of data. Many of the studies that measured SAEs reported no such events in either study arm. For nicotine EC compared to non‐nicotine EC, pooled data suggest no evidence of a difference in the number of people experiencing AEs or SAEs. Conversely, data from comparisons between nicotine EC and behavioural support alone or no support suggest an additional 9 people per 100 assigned to nicotine EC may experience AEs, but with no evidence of a difference in SAEs; this evidence was of low and very low certainty, respectively. As with AEs from other smoking cessation treatments (e.g. NRT, Hartmann‐Boyce 2018a), AEs in these studies typically related to irritation at site (e.g. dry mouth, cough) and resolved over time. No studies in any of the different comparison conditions detected serious harms considered to be related to EC use.

Beyond AEs and SAEs, we consider data on a range of safety‐ and health‐related outcomes, including carbon monoxide and other toxins, lung function, blood pressure, pulse, and oxygen levels. Data on all of these outcome measures were limited; for most outcomes within most comparisons, only one or two studies currently contribute data. A companion paper provides more data on the measured toxicants, analyzing studies based on actual use of ECs and combustible cigarettes (Hartmann‐Boyce 2022a). Consistent with findings from this review, the companion paper found that most measured toxicants were lower in people exclusively using EC than those exclusively smoking or those both smoking and using EC. Most measured toxicants were lower in people using both EC and smoking compared to smoking only.

We also have data from studies testing nicotine EC as adjuncts to other stop‐smoking treatments. Pooled data from two studies in which all participants received NRT showed that nicotine EC led to higher quit rates than non‐nicotine EC, but we judged both studies to be at high risk of bias, meaning the effect remains uncertain. Three studies compare nicotine EC + NRT to NRT alone. Pooling cessation results from all three studies resulted in high statistical heterogeneity, precluding meta‐analysis, but this heterogeneity was driven by the one study of a cartridge device. When restricting the analyses to refillable devices, heterogeneity disappeared (I^2^ = 0%), and results showed more people quit in the nicotine EC + NRT arm than in the NRT alone arm. These results should be treated with caution as one of the two studies was judged to be at high risk of bias, but it does suggest that this is an area where further research is warranted. It is well‐established that combining short‐ and long‐acting forms of NRT ('combined NRT') leads to greater success than single‐form NRT (Theodoulou 2023) but, of note, one of the studies showing a benefit of nicotine EC in this comparison compared nicotine EC + patch to short‐acting NRT + patch, suggesting that it is not just the 'combined NRT' effect that is driving increased effectiveness.

This review also includes data on the proportion of participants still using the study product (EC or pharmacotherapy) at six months or longer. There remains no clear evidence of a between‐group difference for this outcome, which is also now explored further in a companion publication (Butler 2022). We also searched for information investigating any association between withdrawal and smoking cessation, but no studies met our inclusion criteria for this outcome.

Findings from the exploratory network meta‐analysis were consistent with those from pairwise meta‐analyses.

### Overall completeness and applicability of evidence

This field of research and EC devices themselves continue to evolve rapidly. This is the fourth update conducted as part of our 'living systematic review' approach, with which we will proceed until at least the end of 2025, meaning we can continue to rapidly incorporate new evidence (see Appendix 1). This is important, as all but two of our analyses currently demonstrate imprecision.

This update incorporates data from 2 July 2023 to 1 February 2024. Subsequent monthly searches will keep the evidence in this review current. Although studies predominantly came from the USA and UK, overall this review covers data from 15 countries. Geographical range in studies may be particularly important in this area, due to the marked differences in EC regulation between countries; for example, studies conducted in countries that limit nicotine dose in EC, or allow only certain EC devices to be tested, may observe less pronounced effects on quitting. This review includes studies on some under‐researched populations, including people not motivated to quit smoking, people with substance misuse disorders, people with serious mental health conditions, people living in socially deprived areas and people experiencing homelessness. Quit rates in these groups are traditionally lower, which may make it more difficult to detect the effects of interventions. However, it could be that these groups may particularly stand to benefit from EC if they are effective because, in absolute terms, conventional cessation methods are often not as effective for them.

As well as the rapid pace of research in this field, evolutions in EC technology pose a challenge when considering the applicability of our evidence to the present. We had downgraded the certainty of our data in the 2016 update, as the devices tested in the trials were first‐generation 'cig‐a‐like' devices which did not deliver nicotine well, meaning the studies may have yielded more conservative estimates than would be seen with newer models, as newer devices and models have tended towards improved nicotine delivery. Nicotine delivery is also relevant to the comparator NRT arms tested; use of both a shorter‐ and a longer‐acting form of NRT show the highest success, and it is important that, where possible, this be the comparator chosen for such trials (Theodoulou 2023). We no longer downgrade the evidence on this basis as studies with newer device types are now included, although there will always be a time lag between current devices and the research evidence available. Within our primary comparisons, none of the analyses of our primary outcomes signified substantial levels of statistical heterogeneity, despite the fact that different devices were used in the included studies. However, this could be because confidence intervals were wide for individual studies, and does not rule out clinically significant differences in effects between EC types. As further data emerge, we hope to be able to formally test for differences in subgroup analyses, and in head‐to‐head comparisons of different device types. As of this 2024 update, we have only three studies of pod devices contributing to our cessation analyses (Pope 2024; Russell 2021; Xu 2023*). No studies tested newer disposable devices, which data show are growing in popularity (Tattan‐Birch 2022; Tattan‐Birch 2024). There also continues to be little evidence on the impact of different devices, flavours, and nicotine delivery profiles when directly compared to one another, although this update includes the first study that both randomized participants to different flavour conditions and measured smoking cessation at a follow‐up of six months or more. A companion paper explores available data on flavours in more detail but is not as up‐to‐date as this review (Lindson 2022).

The AEs described in both the RCT and cohort studies continue to look similar, regardless of the brand of EC used or nicotine content, with placebo and nicotine‐containing ECs showing similar numbers and types of AEs in direct comparisons. They also reflect what is reported in survey data (Dawkins 2013b; Etter 2011).

The structure of our analyses follows the standard practice of the Cochrane Tobacco Addiction Group, i.e. evaluating outcomes on an intention‐to‐treat basis, meaning our pooled results represent the effect of *offering an EC intervention.* This is different from evaluating the per‐protocol effect, or the effect only on those who use the EC to quit smoking entirely, or continue to smoke whilst also using EC. Although pragmatic and hopefully of use to those designing and delivering interventions, we acknowledge that our intention‐to‐treat approach limits the ability to use the data presented here to draw conclusions about biomarkers in subgroups of participants based on subsequent EC use/smoking profiles. A companion publication attempts to address this deficit (Hartmann‐Boyce 2022a).

#### Cessation

All three comparisons found effect estimates favouring nicotine EC for smoking cessation. For nicotine EC versus NRT, we continue to judge the evidence to be of high certainty, meaning we are very confident that the true effect lies close to the estimate of the effect. For nicotine EC versus non‐nicotine EC, we continue to judge the evidence to be of moderate certainty, meaning we think the true effect is likely to be close to the estimate of effect. For nicotine EC versus behavioural support only/no support, we continue to judge the evidence to be of low certainty, meaning we have limited confidence in the effect estimate. Nicotine EC versus non‐nicotine EC comparisons isolate the effect of nicotine as provided by an EC, and nicotine EC versus NRT comparisons isolate the effect of the sensorimotor elements provided by an EC. Both of these comparisons find a benefit of nicotine EC for smoking cessation. Therefore, it might logically follow that the comparison between nicotine EC and behavioural support only/no support would find a benefit in favour of nicotine EC, since this comparison would capture both pharmacological and sensorimotor mechanisms of effect. This increases our confidence in the effect of nicotine EC when compared to behavioural support alone or to no support. NRT has also been shown to be more effective than behavioural support alone, further supporting the likelihood that nicotine EC would be more effective than behavioural support alone (Hartmann‐Boyce 2018a).

#### Adverse and serious adverse events

We have moderate certainty of no evidence of a difference in adverse events for nicotine EC compared to NRT as well as for non‐nicotine EC. For all other outcomes in this category, evidence is of low or very low certainty. Imprecision remains a key issue for these outcomes, and particularly for SAEs. None of the analyses signalled serious harm, nor did complementary data from cohort studies but, unlike our cessation analyses, many of the confidence intervals encompassed the possibility of both clinically significant harm and clinically significant benefit. This uncertainty should reduce as more studies become available.

### Quality of the evidence

We consider the certainty of the evidence as it relates to primary outcomes for our three main comparisons: nicotine EC versus NRT; nicotine EC versus non‐nicotine EC; nicotine EC versus behavioural support only/no support (Table 1; Table 2; Table 3). The certainty of evidence for all other comparisons and outcomes should be considered very low due to a paucity of data and issues with risk of bias.

Our summary of findings tables and assessments of certainty are based on the evidence from randomized controlled trials (RCTs). The cohort studies that we include are all deemed to have high risks of bias, which is inherent in the study design. Data presented from these studies need to be interpreted with caution. However, data from cohort studies were reassuringly consistent with data from RCTs.

Risk of bias did not impact on the certainty of evidence for comparisons between nicotine and non‐nicotine EC, or between nicotine EC and NRT. For the latter, we judged all seven studies to be at low or unclear risk of bias overall. For the former, removing one study at high risk of bias increased the effect estimate for our efficacy outcome. Risk of bias decreased our certainty in the effect estimates for our nicotine EC versus behavioural support only/no support comparison as, due to the nature of the comparison, blinding was not possible and different levels of support could lead to bias.

All but three of our primary outcomes for our main comparisons were downgraded for imprecision, due to wide confidence intervals and few events. Other than the risk of bias and imprecision, we identified no other issues that decreased the certainty of the primary outcomes for our main comparisons.

Due to the small number of studies contributing to individual analyses, we were unable to formally test for publication bias in most cases and cannot rule this out. For the comparison nicotine EC versus behavioural/no support, two of our outcomes had more than 10 studies contributing to meta‐analysis and therefore we did generate funnel plots. The funnel plot for the exhaled carbon monoxide outcome did not show any evidence of publication bias (Figure 4). However, the funnel plot for smoking cessation showed evidence of asymmetry (Figure 11), suggesting that smaller studies showing no benefit of nicotine EC may remain unpublished. We carried out a sensitivity analysis removing the two outlying studies (i.e. the smallest studies showing the most marked effects of EC; Dawkins 2020; Halpern 2018) and this did not change the interpretation of the pooled result. Therefore, we did not downgrade the certainty of the evidence for publication bias; however, we will continue to monitor this as the evidence is updated.

**11:**
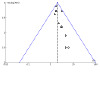
Funnel plot. Comparison: Nicotine EC vs behavioural/no support. Outcome: smoking cessation

### Potential biases in the review process

We consider the review process we used to be robust. For outcome assessment, we followed the standard methods used for Cochrane Tobacco Addiction Review Group cessation reviews. Our search strategy included CENTRAL, which incorporates findings from trial registries, and we were able to capture a number of ongoing studies. However, there may be unpublished data that our searches did not uncover. We also considered participants lost to follow‐up as continuing to smoke, which is standard practice in this field. There are concerns that frequently updating meta‐analyses can lead to issues with multiple testing; we followed Cochrane guidance in conducting this living systematic review and hence do not adjust for multiple testing (Brooker 2019).

Six of our review authors are authors of the included studies. These authors were not involved in the decisions about inclusion of their studies, or in risk of bias assessment for these studies; this approach is standard across all Cochrane reviews (regardless of subject area) and has been approved by the Cochrane editorial office as sufficient to avoid bias.

Our review includes studies funded by the tobacco/vaping industry ‐ Cochrane guidelines (not tobacco addiction‐specific) mandate that studies be included regardless of funder, in order that the reviews remain transparent and rigorous. As noted throughout the results section, we removed studies with tobacco or vaping industry funding in sensitivity analyses; our conclusions were unchanged when we did this. This means that studies funded by tobacco or vaping industries *do not influence* our conclusions. We do not receive any funding from tobacco or vaping industries, and maintain a firm stance of independence.

### Agreements and disagreements with other studies or reviews

This Cochrane review aligns with but updates the conclusions of the 2018 US National Academies of Science, Engineering, and Medicine Consensus Study Report, *Public Health Consequences of E‐cigarettes* (NASEM 2018)*,* which reviewed literature published through August 2017 to address the question, “Do e‐cigarettes help smokers quit smoking combustible tobacco cigarettes?”. Focusing on RCTs and existing systematic reviews, it used a prespecified 'Level of Evidence' framework to develop conclusions. The report’s overall conclusion was that there was “limited evidence that e‐cigarettes may be effective aids to promote smoking cessation.” Based on the RCTs available, it concluded that there was “moderate evidence” that e‐cigarettes containing nicotine were more effective for cessation than e‐cigarettes without nicotine, but “insufficient evidence” about the effectiveness of e‐cigarettes compared to no treatment or to FDA‐approved smoking cessation treatments. Our review differs on this latter point, as we find high‐certainty evidence of benefit when comparing nicotine EC with NRT; this is due to the inclusion of studies published after NASEM 2018. Reviews from the Office for Health Improvement and Disparities (formerly Public Health England) conclude that, compared to their 2018 review, there is now stronger evidence that nicotine vaping products are effective for smoking cessation (McNeill 2021; McNeill 2022).

Zhang 2021 conducted a rapid review; while their pooled analysis also suggested that EC increased quit rates compared to NRT or non‐nicotine EC, they judged the evidence to be of low certainty according to GRADE, driven by imprecision and inconsistency. Zhang 2021 combined studies with NRT comparators and those with non‐nicotine EC comparators in the same analysis and found moderate statistical heterogeneity; we evaluated these two comparisons separately and did not find evidence of statistical heterogeneity. We include more studies than Zhang 2021. Patnode 2021 reviewed evidence on tobacco cessation interventions for the US Preventive Services Task Force (USPFTS 2021). The authors stated that none of their included EC trials suggested higher rates of serious adverse events; this is in line with our analyses. However, they reported that findings across EC trials were inconsistent for effectiveness, with some finding statistically significant evidence of benefit and some finding no statistically significant difference. They did not conduct statistical meta‐analyses and included five trials, all of which are included in our cessation meta‐analyses. None of our cessation meta‐analyses, which include these trials, detected levels of heterogeneity beyond what would be expected from chance alone. Wang 2021 reviewed data both from observational studies and from randomized controlled trials; in the trials, e‐cigarettes were associated with increased smoking cessation (as with our review). In observational studies, ECs were not associated with increased smoking cessation. As discussed in Methods, although we included non‐randomized studies in which an EC intervention is provided in this review, we did not include observational studies in which no EC intervention is provided, due to known issues with confounding.

Chan 2021, Grabovac 2021, Vanderkam 2022 and Levett 2023 also reviewed evidence from randomized controlled trials and found higher quit rates in people assigned to nicotine EC than to NRT or non‐nicotine EC, although Grabovac 2021 noted that evidence was less clear at longer follow‐up when comparing nicotine EC to counselling alone. Pound 2021 only compared nicotine EC with NRT; their pooled estimate showed a higher quit rate with nicotine EC (RR 1.42), but 95% CIs were wide and included the possibility of no difference. They included two studies in their comparison that we do not: one which measured cessation at less than six months and hence was not eligible for inclusion in our cessation analysis, and one in which the nicotine level was so low that we have classified the study as assessing non‐nicotine (Lee 2019). The latter introduced statistical heterogeneity to their pooled results. We also include additional studies not available at the time of their analyses.

Findings are also broadly consistent with those from other recent reviews regarding safety and markers of harm, with some exceptions. Amato 2020 focused only on safety; consistent with our review, they found very low‐ to moderate‐certainty evidence of a range of possible adverse effects, with the most frequently reported being cough, dry mouth, shortness of breath, irritation of the mouth and throat, and headache. Consistent with our review, the studies reviewed by McNeill 2022 showed that, compared to combustible cigarettes, using ECs led to a substantial reduction in biomarkers of toxicant exposure associated with cigarette smoking; RCP 2024 and Wilson 2021 also agree with this finding. Akiyama 2021 reviewed biomarker findings from clinical studies and also concluded that the use of EC could lead to a significant reduction in exposure to harmful substances compared to traditional cigarettes; this is again consistent with findings from our review. A systematic review of 22 studies found that several carcinogens with a known link to bladder cancer were present in the urine of EC users and recommended further study on the urological safety of ECs (Bjurlin 2021). Taylor 2023 systematically reviewed tobacco‐specific nitrosamine exposure between people smoking, vaping, and doing neither. Exposure to all tobacco‐specific nitrosamines was lower amongst people who vaped compared to people who smoked. Levels were higher amongst people who vaped compared to people who neither vaped nor smoked.

Martinez‐Morata 2021 reviewed blood pressure findings and concluded that EC may result in short‐term elevations, but that more data are needed; our review also lacks sufficient data to draw any conclusions about blood pressure at one week or longer. A scoping review by Gugala 2022 looked at the pulmonary health effects of EC and found an association between EC use and negative pulmonary symptoms. EC use resulted in worse outcomes than non‐smoking, but resulted in improved outcomes when compared with combustible cigarette use or dual use of combustible cigarettes and EC. The review by McNeill 2022 found acute and short‐to‐medium exposure to most potential respiratory toxicants from ECs to be significantly lower than combustible cigarettes, with substantial reductions in some biomarkers. For the respiratory toxicants assessed at long‐term exposure, evidence was moderate. McNeill 2022 found moderate evidence that exposure to most respiratory toxicants from ECs was similar to non‐use of tobacco or nicotine products. Banks 2022 focused on the absolute risks of EC; in this review, we are interested in both their absolute and relative risks in comparison to smoking. Siddiqi 2023 carried out a systematic review of randomized and observational studies on the association between EC and cardiovascular health and concluded that EC use was associated with a significant increase in cardiovascular haemodynamic measures and biomarkers. A systematic review by Meng 2023 on the acute effects of EC on vascular endothelial function included eight RCTs. Evidence from their pooled analyses indicated that acute inhalation of EC led to negative changes in vascular endothelial function. Thiem 2023 carried out a systematic review of EC and CC on periodontal health; findings suggested that EC use might be considered a healthier alternative to CC concerning periodontal health. However, harmful effects of EC usage on periodontal health were observed.

A network meta‐analysis, with searches up‐to‐date until February 2019, used direct and indirect evidence to compare the effectiveness and safety of ECs to placebo, bupropion, NRT, and varenicline (Thomas 2022). The evidence was imprecise; however, there was evidence of a benefit of ECs with a nicotine level of 15 mg over placebo. The effect estimate also suggested a benefit of ECs with a 10 mg nicotine level, but the credibility interval indicated the possibility of both benefit and harm. Similarly, when EC was compared with individual pharmacotherapies, the direction of effect was in favour of ECs; however, imprecision means further evidence may change the interpretation of the effect. The safety data for ECs was inconclusive. A second network meta‐analysis also suffered from imprecision when comparing EC and NRT, though CIs were consistent with our results (Quigley 2021). A component network analysis updating this evidence and investigating additional components of the interventions and studies reports findings consistent with our review. Compared to placebo/no treatment, nicotine e‐cigarettes were associated with an approximate doubling of the odds of successfully quitting smoking at six months or longer (OR 2.37, 95% CrI 1.73 to 3.24; 16 RCTs, 3828 participants), placing them in the top three most effective treatments (alongside varenicline and cytisine). Evidence suggested combination NRT may also be as effective, but the point estimate was lower and this evidence was less certain (Lindson 2023a). SAEs were rare and there was no clear evidence of a difference in the proportion of people experiencing SAEs between those randomized to nicotine e‐cigarettes and to no treatment, or to nicotine replacement therapies or nicotine receptor partial agonists.

A systematic review by Liber 2023 examined the evidence on the role of EC flavours in quit rates and concluded that the evidence was inconclusive, reflecting highly heterogeneous study definitions and methodological limitations, and called for more high‐quality evidence, ideally from RCTs.

Reviews of ECs for policymaking (e.g. Banks 2023) are often broader in scope than our review, which focuses exclusively on their role in supporting smoking cessation in people who smoke. Outside of smoking cessation, there remain unanswered questions about the impact of EC availability and use, including in young people; we are evaluating this in a separate review (Hartmann‐Boyce 2022b).

## Authors' conclusions

**Implications for practice**

Evidence suggesting that nicotine electronic cigarettes (EC) can aid in smoking cessation is consistent across several comparisons. There is high‐certainty evidence that EC with nicotine increases quit rates at six months or longer compared to nicotine replacement therapy (NRT), and moderate‐certainty evidence (limited by imprecision) that EC with nicotine increases quit rates at six months or longer compared to non‐nicotine EC. There is also low‐certainty evidence (limited by risk of bias) that EC with nicotine may increase quit rates compared to behavioural support alone or to no support.

Issues with risk of bias, few studies, and differences between studies preclude strong conclusions regarding the effect of nicotine EC when added to NRT, but the data available suggest a benefit.

None of the evidence synthesized provides a clear indication that serious adverse events are increased by EC use. However, more long‐term data are needed, and this conclusion relates specifically to people using EC to quit smoking and not to people who have never smoked. The most commonly reported adverse effects are throat/mouth irritation, headache, cough, and nausea, which tend to dissipate with continued use. In some studies, reduced toxin concentrations and biomarkers of harm were observed in people who smoked and switched to vaping, consistent with reductions seen in people who stopped smoking without EC.

**Implications for research**

Further randomized controlled trials of nicotine EC are needed. All studies (including uncontrolled intervention cohort studies) should aim to assess the safety profile of EC for as long as possible (the current review only includes data up to two years), and ideally be powered to detect differences in safety outcomes, particularly serious adverse events. Safety results should be presented in both absolute and relative risk terms (in comparison to the risks of continuing to smoke tobacco).

Studies with active comparators (i.e. comparing nicotine EC to frontline smoking cessation pharmacotherapies, particularly those other than NRT) are likely to be of particular use to decision‐makers, as are those testing EC as an adjunct to existing stop‐smoking pharmacotherapies; in particular, those testing combinations of traditional NRTs with e‐cigarettes (e.g. patch plus e‐cigarettes).

Studies should offer recent devices with good nicotine delivery to participants to be most representative of what will be on the market at the time results are released. Studies should also monitor and collect data on participants switching use of other devices during trials, and use of different flavours and nicotine strengths. Protocols and statistical analysis plans should be registered in advance and openly available.

Further RCTs need to be adequately powered. Further trials of pod and newer disposable devices would be of particular value, as would RCTs providing ECs in a way that would be used in real‐world settings (e.g. taking into account individual preferences for strengths and flavours of e‐liquids and even EC devices, and also allowing for changes in preferences over time). Further studies directly comparing nicotine ECs based on characteristics including nicotine content and delivery, flavour, and device type, and reporting outcomes including cessation at six months or longer, would also be particularly useful.

Further reviews, using the best available methods, need to be conducted to evaluate the possible relationships between EC use and availability and youth uptake of EC and conventional cigarettes (such as Hartmann‐Boyce 2022b), as well as the safety of EC use in people who have never smoked.

## What's new

**Table:** 

**Date**	**Event**	**Description**
29 January 2025	New search has been performed	Updated to include two new studies; searches to 1 February 2024
29 January 2025	New citation required but conclusions have not changed	Main conclusions remain unchanged. Update triggered as first study comparing flavours and reporting abstinence at six months or longer was published (results inconclusive).

## History

Protocol first published: Issue 11, 2012 Review first published: Issue 12, 2014

**Table:** 

**Date**	**Event**	**Description**
8 January 2024	New citation required and conclusions have changed	Certainty of evidence for cessation outcome for comparison with behavioural support/no support upgraded from very low to low
8 January 2024	New search has been performed	This is a living systematic review. In this update, we incorporate data to 1st July 2023.
15 March 2023	Amended	This is a Living Systematic Review. We run and screen searches monthly. Last search date: 1st March 2023. In addition to the studies identified from August 2022 to February 2023, we found one new reference linked to a previously identified study. We will incorporate this into the review as part of a future update. We have also fixed a typo in the plain language summary. For future monthly search results, please see 'Monthly search results' via the following link: https://www.cebm.ox.ac.uk/research/electronic-cigarettes-for-smoking-cessation-cochrane-living-systematic-review-1.
4 February 2023	Amended	This is a Living Systematic Review. We run and screen searches monthly. Last search date: 1st February 2023. In addition to the studies identified from August 2022 to January 2023, we found one new included study, one new ongoing study and 2 linked references. We will incorporate these into the review as part of a future update. The DOI for the 1 new included study (Kanobe 2022) is: https://doi.org/10.1038/s41598-022-25054-z.
5 January 2023	Amended	This is a Living Systematic Review. We run and screen searches monthly. Last search date: 3rd January 2023. In addition to the studies identified from August 2022 to December 2022, we found one new ongoing study. We will incorporate these into the review as part of a future update. In addition, some minor corrections were made to the Characteristics of Included Studies table for Hajek 2022 based on a published correction to the study's primary manuscript (https://doi.org/10.1038/s41591-022-02099-1).
12 December 2022	Amended	This is a Living Systematic Review. We run and screen searches monthly. Last search date: 1st December 2022. In addition to the studies identified from August 2022 to November 2022, we found one new ongoing study and 3 records linked to previously identified studies. We will incorporate these into the review as part of a future update.
25 November 2022	Amended	This is a Living Systematic Review. We run and screen searches monthly. Last search date: 1st November 2022. We found no new eligible references. As part of this amendment, we also updated the citation for additional reference Lindson 2022, and corrected a slight error in wording in the Discussion section.
19 October 2022	New citation required and conclusions have changed	Certainty changes for some of the primary outcomes.
19 October 2022	New search has been performed	17 new included studies. Incorporates evidence up to the 1st July 2022.
7 October 2022	Amended	This is a Living Systematic Review. We run and screen searches monthly. Last search date: 1st October 2022. In addition to the studies identified from June 2021 to September 2022, we found one new included study, 3 new ongoing studies and 1 record linked to a previously identified study. The DOI for the 1 new included study is: Klonizakis 2022 (https://doi.org/10.1186/s12916-022-02451-9). We will incorporate these into the review as part of a future update.
27 September 2022	Amended	This is a Living Systematic Review. We run and screen searches monthly. Last search date: 1st September 2022. In addition to the studies identified from June 2021 to August 2022, we found two records linked to previously identified studies. We will incorporate these into the review as part of a future update.
17 August 2022	Amended	This is a Living Systematic Review. We run and screen searches monthly. Last search date: 1st August 2022. In addition to the studies identified from June 2021 to July 2022, we found two new included studies, 1 new ongoing study and 3 records linked to previously identified studies. The DOIs for the 2 new included studies are: Coffey 2020 (DOI: 10.1177/1757913920912436) and Price 2022 (DOI: https://doi.org/10.1186/s12889-022-13711-x). We will incorporate these into the review as part of a future update.
8 July 2022	Amended	This is a Living Systematic Review. We run and screen searches monthly. Last search date: 1st July 2022. In addition to the studies identified from June 2021 to June 2022, we found four new included studies, 1 new ongoing study and 8 records linked to previously identified studies. The DOIs for 3 of the new included studies are: Edmiston 2022 (DOI: 10.1093/ntr/ntac029); Tattan‐Birch 2022 (DOI: 10.1093/ntr/ntac149) and Morphett 2022a (DOI: 10.1093/ntr/ntab266). The fourth new included study was presented at SRNT 2022 (abstract reference: SYM17‐4). We will incorporate these into the review as part of a future update.
15 June 2022	Amended	This is a Living Systematic Review. We run and screen searches monthly. Last search date: 1st June 2022. In addition to the studies identified from June 2021 to May 2022, we found three new included studies (all previously listed as ongoing studies) and 2 records linked to a previously identified study. The DOIs for the new included studies are: Hajek 2022 (https://doi.org/10.1038/s41591-022-01808-0); Bonafont Reyes 2022 (https://doi.org/10.1111/jgs.17755) and Vickerman 2022 (https://doi.org/10.1093/ntr/ntac129). We will incorporate these into the review as part of a future update.
6 May 2022	Amended	This is a Living Systematic Review. We run and screen searches monthly. Last search date: 1st May 2022. In addition to the studies identified from June 2021 to April 2022, we found two new included studies (previously listed as ongoing studies), 3 new ongoing studies and 2 records linked to previously identified studies. The DOIs for the new included studies are: Skelton 2022 (doi: 10.1016/j.addbeh.2022.107328); Pratt 2022 (doi: 10.1093/ntr/ntac082). We will incorporate these into the review as part of a future update.
6 April 2022	Amended	This is a Living Systematic Review. We run and screen searches monthly. Last search date: 1st April 2022. In addition to the studies identified from June 2021 to March 2022, we found 4 new ongoing studies. We will incorporate these into the review as part of a future update.
7 March 2022	Amended	This is a Living Systematic Review. We run and screen searches monthly. Last search date: 1st March 2022. In addition to the studies identified from June 2021 to February 2022, we found 1 record linked to a study already identified as ongoing. We will incorporate these into the review as part of a future update.
11 February 2022	Amended	This is a Living Systematic Review. We run and screen searches monthly. Last search date: 1st February 2022. In addition to the studies identified from June 2021 to January 2022, we found 2 ongoing studies and 2 records linked to studies already included in the review. We will incorporate these into the review as part of a future update.
12 January 2022	Amended	This is a Living Systematic Review. We run and screen searches monthly. Last search date: 1st January 2022. In addition to the studies identified from June to December 2021, we found 4 ongoing studies and 1 record linked to a study already included in the review. We will incorporate these into the review as part of a future update.[Enter text here]
16 December 2021	Amended	This is a Living Systematic Review. We run and screen searches monthly. Last search date: 1st December 2021. In addition to the studies identified from June to November 2021, we found six new included studies, 15 ongoing studies and 18 records linked to studies already included in the review. The DOI or trial IDs for the new included studies are: NCT02433015; NCT03111537; NCT03185546; NCT03358953; Caponnetto 2021 (DOI: 10.1093/ntr/ntab005); Lum 2021 (DOI: 10.1016/j.addbeh.2021.107097). We will incorporate these into the review as part of a future update.
3 November 2021	Amended	This is a Living Systematic Review. We run and screen searches monthly. Last search date 1st November 2021. In addition to the studies identified from June to October 2021, we found one new included study. The DOI for the new included study (Okuyemi 2021) is 10.1093/ntr/ntab212. We will incorporate these into the review as part of a future update.
20 October 2021	Amended	This is a Living Systematic Review. We run and screen searches monthly. Last search date 1st October 2021. In addition to the studies identified from June to September 2021, we found one new included study two reports linked to studies already in the review, and one new ongoing. The DOI for the new included study (Morris 2021) is https://doi.org/10.1007/s11739-021-02813-w. We will incorporate these into the review as part of a future update.
16 September 2021	Amended	Change made to correct data; SAE data from Cobb 2021 moved from comparison with NRT to comparison with no‐nicotine EC. No changes to conclusions.
6 September 2021	New search has been performed	Updated with five new included studies. Incorporates evidence up to 1 May 2021.
6 September 2021	New search has been performed	This is a Living Systematic Review. We run and screen searches monthly. Last search update 1st September 2021. We found no new studies for inclusion this month; however results from searches carried out from June to August 2021 will be incorporated into a future update of the review.
6 September 2021	New citation required and conclusions have changed	New secondary outcome added (continued product use), first study of pod device contributing data to cessation meta‐analysis added, two new comparisons added (nicotine salt EC versus freebase nicotine EC; advice on how to quit smoking using EC versus no EC advice). Conclusions for primary outcomes remain largely unchanged.
5 August 2021	Amended	This is a Living Systematic Review. We run and screen searches monthly. Last search date 2nd August 2021. In addition to the studies identified from March to July 2021, we found two new ongoing studies and one report linked to a study already in the review. We will incorporate these into the review as part of a future update.
7 July 2021	Amended	This is a Living Systematic Review. We run and screen searches monthly. Last search date 1st July 2021. In addition to the studies identified from March to June 2021, we found two new included studies and two reports linked to studies already in the review. DOIs for the two new included studies are as follows: Myers‐Smith 2021: https://doi.org/10.1111/add.15628 & Kimber 2021: 10.1016/j.addbeh.2021.106909. We will incorporate these into the review as part of a future update.
9 June 2021	Amended	This is a Living Systematic Review. We run and screen searches monthly. Last search date 1st June 2021. In addition to the studies identified from March to May 2021, we found one report linked to a study already in the review, one ongoing study, and one potentially new study that we are looking into further. We will incorporate these into the review as part of a future update. As part of this new update we will also include a new outcome ‐ proportion of people still using e‐cigarettes or other pharmacotherapy at longest follow‐up.
12 May 2021	Amended	This is a Living Systematic Review. We run and screen searches monthly. Last search date 4th May 2021. In addition to the studies identified from March and April 2021, we found four new ongoing studies. We will incorporate these into the review as part of a future update.
15 April 2021	New citation required and conclusions have changed	6 new included studies added (Czoli 2019; Ikonomidis 2020a; Ozga‐Hess 2019; Pulvers 2020; Scheibein 2020; Yingst 2020), certainty in finding of no difference in adverse events between nicotine EC and non‐nicotine EC updated to moderate (from low). First study of pod EC device included.
15 April 2021	New search has been performed	Updated with six new included studies and new data from one previously included study. Most recent search 1 Feb 2021.
1 April 2021	Amended	This is a Living Systematic Review. We run and screen searches monthly. Last search date 1st April 2021. In addition to the studies identified from March 2021 we found two new ongoing studies and one paper linked to a study already included in the review. We will incorporate these into the review as part of a future update.
17 March 2021	Amended	This is a Living Systematic Review. We run and screen searches monthly. Last search date 1st March 2021. Studies identified in March are not included in this version of the review, but will be incorporated into a subsequent version. We found four new included studies, five new ongoing studies and five papers linked to studies already included in the review. The four new included studies were all conference abstracts; three of which were identified from the SRNT 2021 abstract book (SYM2A, SYM2B, PH‐353; www.srnt.org/page/2021_Meeting). The fourth is available here: dx.doi.org/10.1016/j.drugalcdep.2015.07.1091.
4 February 2021	Amended	This is a Living Systematic Review. We run and screen searches monthly. Last search date 1st February 2021. In addition to the studies identified from our December 2020 and January 2021 searches we found one paper linked to a study already included in the review (Lucchiari 2022), and have preliminary results from a study listed as ongoing (Begh 2021). We will incorporate this paper and data into the review as part of a future update.
20 January 2021	Amended	This is a Living Systematic Review. Searches are run and screened monthly. Last search date 4th January 2021. In addition to the studies identified from our December 2020 searches we found four new completed studies, one new ongoing study and one paper linked to a study already included in the review. These studies and papers will be incorporated into the review at the next update. DOIs for the four new included studies are as follows: Ozga‐Hess et al. 2019: 10.1016/j.addbeh.2019.106105; Pulvers et al. 2020: 10.1001/jamanetworkopen.2020.26324; Scheibein 2020: 10.1186/s12954-020-00406-y; Yingst et al. 2020: 10.1080/09540121.2019.1687835
15 December 2020	Amended	This is a Living Systematic Review. Searches are run and screened monthly. Last search date 1st December 2020. Searches found 3 new completed studies, 11 new ongoing studies and 9 papers linked to studies already included in the review. These studies and papers will be incorporated into the review at the next update. DOIs for the three new included studies are as follows: Czoli et al:10.1093/ntr/nty174;Bonevski et al: 10.1093/ntr/ntaa143;Eisenberg et al: 10.1001/jama.2020.18889.
20 July 2020	New search has been performed	New searches run January 2020. 35 new studies added. Living systematic review protocol incorporated
20 July 2020	New citation required and conclusions have changed	Strength of evidence increased for existing comparisons; new comparisons added
14 December 2016	Amended	Clarification on outcome data from Adriaens ‐ no changes to conclusions
23 June 2016	New citation required but conclusions have not changed	11 new included studies added; no changes to conclusions.
23 June 2016	New search has been performed	Update search run January 2016, 11 new included studies added. Reduction removed as outcome, now covered in Harm Reduction review.

## Appendices

### Appendix 1. Protocol for living systematic review

#### Justification for ‘Living Review’ status

Living systematic reviews (LSRs) offer a new approach to updating reviews, in which the review is continually updated by incorporating relevant new evidence as it becomes available (Brooker 2019). Previous versions of this Cochrane review of electronic cigarettes (ECs) for smoking cessation have informed policy worldwide (Hartmann‐Boyce 2016; McRobbie 2014). This update has found high degrees of uncertainty (low‐ and very low‐certainty evidence) for most outcomes, due to the small number of included randomized controlled trials, and the resulting imprecision in effect estimates. This means that some conclusions are likely to change substantially as new evidence emerges.

On average, Cochrane reviews are updated every three to four years. For EC, where the evidence base is rapidly evolving, this schedule impedes the ability of the review to provide the most up‐to‐date evidence to decision‐makers. As EC use, availability, and design changes, policymakers are frequently drawing on this review to inform decisions, so it is imperative that it is up‐to‐date to ensure decisions are being made on the basis of the entirety of the evidence. Regular updates have the potential to strengthen the existing conclusions of the review or to change conclusions where conflicting evidence or evidence on new outcomes emerges (e.g. comparisons between EC and other interventions; longer‐term safety data).

#### Objective of the change to ‘Living Review’ status

To implement approved Cochrane LSR methods to provide an up‐to‐date, accessible, engaging, and unbiased review of the evidence on the effect and safety of using EC to quit smoking.

#### LSR methodological considerations

The methods outlined below are specific to maintaining this review of *Electronic cigarettes for smoking cessation* as an LSR on the Cochrane Library. These methods will be ‘active’ immediately upon publication of this update. Core review methods, such as the criteria for considering studies in the review and assessment of risks of bias, are unchanged and are detailed in the main body of the review. Below we outline the methods for which specific considerations apply as a result of the change to ‘living’ status.

##### Search methods for identification of studies

We will conduct database searches monthly, beginning December 2020. These searches will be of the Cochrane Central Register of Controlled Trials (CENTRAL), MEDLINE, Embase, PsycINFO, and clinical trial registries, as detailed in the main body of the review. The funders of this LSR – Cancer Research UK (CRUK) ‐ already run monthly searches of the EC evidence, and so we will work alongside their health information officer to ensure that we are identifying all the relevant literature with our searches. We will review our search strategies on an ongoing basis every 12 months, as indexing terms and keywords may change, and new search filters may be published. Such changes will be managed by input from experienced information specialists.

##### Selection of studies

We will immediately screen any new citations retrieved by the monthly searches using Covidence, undertaking dual screening of title and abstract, and then full text, by independent review authors. Where we find multiple citations of the same study, we will group them into one study record with a single study ID. One review author (AB) will contact corresponding authors of potentially relevant ongoing studies as they are identified and ask them to advise when results are available, or to share early or unpublished data. Based on the information and projected time scales shared, we will contact corresponding authors on an ongoing basis to retrieve new evidence as it becomes available.

##### Data synthesis

Whenever we identify new studies relevant to the review, we will extract the relevant data and assess the risks of bias as detailed in the main body of the review. We will highlight the availability of this new evidence on both the Cochrane Library and on our own dedicated website. We will incorporate the new data into meta‐analyses and tables in RevMan (Review Manager 2022) and supplementary data files, and carry out GRADE assessments (GRADEpro GDT). We will conduct a full update of the review (full incorporation and interpretation of all new data within the review and re‐publishing) when the accumulating evidence leads to changes in any one of:

- the direction of effect or clinical significance of the findings for one or more outcomes;
- the certainty (e.g. GRADE rating) of one or more outcomes;
- the availability of studies investigating new settings, populations, interventions, comparisons, or outcomes.

Formal sequential meta‐analysis approaches will not be used for updated meta‐analyses, in line with Cochrane guidance for LSRs.

#### Future updates of review methods

The LSR approach acknowledges that reviews may cease to need to be ‘living’ over time, as the review findings become stable, or the question is no longer a priority for decision‐makers (Brooker 2019). We continue to evaluate the LSR approach, including the likely benefits of and challenges to continuing this methodology for this evidence base, and whether such an approach remains warranted. We held evaluation meetings in 2022, 2023, and 2024 and plan to hold a meeting in 2025. If the evidence is high certainty for all outcomes and all comparisons at that point, meaning further studies are judged very unlikely to impact the effect estimate, we would consider ceasing living mode for this review. If, as is more likely, some or all outcomes are not yet certain, we will facilitate discussions within the author team and Cochrane, as well as engaging with a wider PPI panel and key decision‐makers, e.g. policymakers, in order to determine next steps. If the decision is made to continue in living mode, we will review, and if necessary revise, the living review methods described in this Appendix before continuing.

### Appendix 2. Toxins/carcinogen names and abbreviations

**Table:**

**Abbreviation**	**Name**
‐	1‐Hydroxyfluorene
‐	1‐Hydroxyphenanthrene
‐	1‐Hydroxypyrene
2‐HPMA	2‐hydroxypropylmercapturic acid
‐	2‐Hydroxyfluorene
‐	2‐Hydroxyphenanthrene
‐	2‐Naphthol
‐	3‐, 4‐Hydroxyphenanthrenes
3‐HPMA	3‐hydroxypropylmercapturic acid
‐	3‐Hydroxyfluorene
AAMA	*N*‐acetyl‐*S*‐(carbamoylethyl)‐L‐cysteine (synonym: 2‐carbamoylethylmercapturic acid)
CEMA/CNEMA	2‐cyanoethylmercapturic acid; referred to as 'acrylonitrile' in Pulvers 2018
‐	Formic acid
HEMA	2‐hydroxyethylmercapturic acid
HMPMA/HPMMA	3‐hydroxy‐1‐methyl propylmercapturic acid
MHBMA	2‐hydroxy‐3‐buten‐1‐ylmercapturic acid
MMA	N‐nitrosodimethyamine
NNAL	4‐(methylnitrosamino)‐1‐(3‐pyridyl)‐1‐butanol
PheT	Phenanthrene tetraol
PMA	phenylmercapturic acid; referred to as 'benzene' in Pulvers 2018
S‐PMA	S‐phenylmercapturic acid

### Appendix 3. Search strategies ‐ 2020 update onwards

**Ovid databases (MEDLINE, Embase, PsycINFO)**

1. exp case control studies/ or exp cohort studies/ or Case control.tw. or (cohort adj (study or studies)).tw. or Cohort analy$.tw. or (Follow up adj (study or studies)).tw. or (observational adj (study or studies)).tw. or Longitudinal.tw.

2. (e‐cig$ or ecig$ or electr$ cigar$ or electronic nicotine).mp. or (vape or vapes or vaporizer or vapourizer or vaporiser or vapouriser or vaper or vapers or vaping).ti,ab. or exp Electronic Nicotine Delivery Systems/

3. (randomized controlled trial or controlled clinical trial).pt. or randomized.ab. or placebo.ab. or clinical trials as topic.sh. or randomly.ab. or trial.ti.

4. exp animals/ not human/

5. 3 not 4

6. 2 and 5

7. 1 and 2

8. 6 or 7

9. smoking cessation.mp. or exp Smoking Cessation/

10. tobacco cessation.mp. or "Tobacco‐Use‐Cessation"/

11. (nicotine dependence or tobacco dependence).mp.

12. exp Smoking/th

13. "Tobacco‐Use‐Disorder"/

14. Smoking reduction/ or Smoking reduction.mp.

15. exp Pipe smoking/ or exp Tobacco smoking/ or exp Tobacco Products/

16. ((quit$ or stop$ or ceas$ or giv$ or abstain* or abstinen*) adj5 (smoking or smoke* or tobacco)).ti,ab.

17. exp Tobacco/ or exp Nicotine/

18. 9 or 10 or 11 or 12 or 13 or 14 or 15 or 16 or 17

19. 8 and 18

**CENTRAL (via CRS‐Web), (also used to search CTAG Specialised Register until March 2023)**

1. (e‐cig* or ecig* or electr* cigar* or electronic nicotine):ti,ab,KY,MH,EMT,KW,XKY,EH,KY

2. (vape or vapes or vaporizer or vapourizer or vaporiser or vapouriser or vaper or vapers or vaping):ti,ab,KY,MH,EMT,KW,XKY,EH,KY

3. MESH DESCRIPTOR Electronic Nicotine Delivery Systems EXPLODE ALL

4. #3 OR #2 OR #1

### Appendix 4. MEDLINE search strategy ‐ pre‐2020

1. e‐cig$.mp. [mp=title, abstract, original title, name of substance word, subject heading word, protocol supplementary concept, rare disease supplementary concept, unique identifier]
2. electr$ cigar$.mp.
3. electronic nicotine.mp.
4. (vape or vaper or vapers or vaping).ti,ab.
5. 1 OR 2 OR 3 OR 4

Identical terms used for other databases.

Line 4 added to search strategy for 2016 update.

## Data and analyses

**Comparison 1:** Nicotine EC versus NRT

Outcome or subgroup title	No. of studies	No. of participants	Statistical method	Effect size
1.1 Smoking cessation	7	2544	Risk Ratio (M‐H, Fixed, 95% CI)	1.59 [1.30, 1.93]
1.1.1 Not selected on pregnancy	6	2225	Risk Ratio (M‐H, Fixed, 95% CI)	1.58 [1.29, 1.93]
1.1.2 Pregnant population	1	319	Risk Ratio (M‐H, Fixed, 95% CI)	1.78 [0.45, 6.97]
1.2 Adverse events	5	2052	Risk Ratio (M‐H, Fixed, 95% CI)	1.03 [0.91, 1.17]
1.2.1 4 weeks	1	29	Risk Ratio (M‐H, Fixed, 95% CI)	0.74 [0.31, 1.73]
1.2.2 12 weeks	1	350	Risk Ratio (M‐H, Fixed, 95% CI)	1.07 [0.87, 1.31]
1.2.3 6 months	2	563	Risk Ratio (M‐H, Fixed, 95% CI)	1.01 [0.82, 1.24]
1.2.4 3 months after end of pregnancy	1	1110	Risk Ratio (M‐H, Fixed, 95% CI)	1.05 [0.84, 1.31]
1.3 Serious adverse events	6	2761	Risk Ratio (M‐H, Fixed, 95% CI)	1.20 [0.90, 1.60]
1.3.1 4 weeks	1	29	Risk Ratio (M‐H, Fixed, 95% CI)	Not estimable
1.3.2 12 weeks	1	350	Risk Ratio (M‐H, Fixed, 95% CI)	1.80 [0.86, 3.79]
1.3.3 6 months	2	563	Risk Ratio (M‐H, Fixed, 95% CI)	1.53 [0.81, 2.88]
1.3.4 1 year	1	698	Risk Ratio (M‐H, Fixed, 95% CI)	1.37 [0.77, 2.41]
1.3.5 3 months after end of pregnancy	1	1121	Risk Ratio (M‐H, Fixed, 95% CI)	0.83 [0.52, 1.31]
1.4 Carbon monoxide (ppm)	4	357	Mean Difference (IV, Fixed, 95% CI)	‐1.81 [‐3.64, 0.01]
1.4.1 Absolute values at follow‐up	1	110	Mean Difference (IV, Fixed, 95% CI)	‐1.87 [‐5.15, 1.41]
1.4.2 Change from baseline	3	247	Mean Difference (IV, Fixed, 95% CI)	‐1.79 [‐3.98, 0.41]
1.5 Heart rate (bpm)	2	166	Mean Difference (IV, Fixed, 95% CI)	0.53 [‐1.76, 2.83]
1.5.1 Absolute values at follow‐up	1	111	Mean Difference (IV, Fixed, 95% CI)	‐0.74 [‐5.17, 3.69]
1.5.2 Change from baseline	1	55	Mean Difference (IV, Fixed, 95% CI)	1.00 [‐1.69, 3.69]
1.6 Systolic blood pressure	2	166	Mean Difference (IV, Fixed, 95% CI)	‐1.62 [‐3.59, 0.36]
1.6.1 Absolute values at follow‐up	1	111	Mean Difference (IV, Fixed, 95% CI)	1.00 [‐4.54, 6.54]
1.6.2 Change from baseline	1	55	Mean Difference (IV, Fixed, 95% CI)	‐2.00 [‐4.11, 0.11]
1.7 Blood oxygen saturation	2	165	Mean Difference (IV, Fixed, 95% CI)	‐0.14 [‐0.59, 0.30]
1.7.1 Absolute values at follow‐up	1	110	Mean Difference (IV, Fixed, 95% CI)	‐0.20 [‐0.72, 0.32]
1.7.2 Change from baseline	1	55	Mean Difference (IV, Fixed, 95% CI)	0.00 [‐0.83, 0.83]
1.8 3‐HPMA (pmol/mg creatinine)	1		Mean Difference (IV, Fixed, 95% CI)	Totals not selected
1.8.1 Absolute values at follow‐up	1		Mean Difference (IV, Fixed, 95% CI)	Totals not selected
1.9 NNAL (pmol/mg creatinine))	1		Mean Difference (IV, Fixed, 95% CI)	Totals not selected
1.9.1 Absolute values at follow‐up	1		Mean Difference (IV, Fixed, 95% CI)	Totals not selected
1.10 2‐HPMA (pmol/mg creatinine)	1		Mean Difference (IV, Fixed, 95% CI)	Totals not selected
1.10.1 Absolute values at follow‐up	1		Mean Difference (IV, Fixed, 95% CI)	Totals not selected
1.11 HMPMA (pmol/mg creatinine)	1		Mean Difference (IV, Fixed, 95% CI)	Totals not selected
1.11.1 Absolute values at follow‐up	1		Mean Difference (IV, Fixed, 95% CI)	Totals not selected
1.12 PheT (pmol/mg creatinine)	1		Mean Difference (IV, Fixed, 95% CI)	Totals not selected
1.12.1 Absolute values at follow‐up	1		Mean Difference (IV, Fixed, 95% CI)	Totals not selected
1.13 CEMA (pmol/mg creatinine)	1		Mean Difference (IV, Fixed, 95% CI)	Totals not selected
1.13.1 Absolute values at follow‐up	1		Mean Difference (IV, Fixed, 95% CI)	Totals not selected
1.14 AAMA (pmol/mg creatinine)	1		Mean Difference (IV, Fixed, 95% CI)	Totals not selected
1.14.1 Absolute values at follow‐up	1		Mean Difference (IV, Fixed, 95% CI)	Totals not selected
1.15 FEV1	2		Std. Mean Difference (IV, Fixed, 95% CI)	Totals not selected
1.15.1 Change from baseline	2		Std. Mean Difference (IV, Fixed, 95% CI)	Totals not selected
1.16 FEV1/FVC (%)	2	81	Mean Difference (IV, Fixed, 95% CI)	‐0.16 [‐1.83, 1.50]
1.16.1 Change from baseline	2	81	Mean Difference (IV, Fixed, 95% CI)	‐0.16 [‐1.83, 1.50]
1.17 PEF (L/min)	1		Mean Difference (IV, Fixed, 95% CI)	Totals not selected
1.17.1 Change from baseline	1		Mean Difference (IV, Fixed, 95% CI)	Totals not selected
1.18 Product use at 6+ months	5		Risk Ratio (M‐H, Fixed, 95% CI)	Totals not selected

**Comparison 2:** Nicotine EC versus varenicline

Outcome or subgroup title	No. of studies	No. of participants	Statistical method	Effect size
2.1 Smoking cessation	1		Risk Ratio (M‐H, Fixed, 95% CI)	Totals not selected
2.2 Serious adverse events	1	54	Risk Ratio (M‐H, Fixed, 95% CI)	Not estimable
2.2.1 12 weeks	1	54	Risk Ratio (M‐H, Fixed, 95% CI)	Not estimable

**Comparison 3:** Nicotine EC versus heated tobacco

Outcome or subgroup title	No. of studies	No. of participants	Statistical method	Effect size
3.1 Adverse events at 12 weeks	1		Risk Ratio (M‐H, Fixed, 95% CI)	Totals not selected
3.2 SAEs at 12 weeks	1		Risk Ratio (M‐H, Fixed, 95% CI)	Totals not selected
3.3 Carbon monoxide (ppm) absolute values at 12 weeks	1		Mean Difference (IV, Fixed, 95% CI)	Totals not selected
3.4 VO_2_ Max at 12 weeks	1		Mean Difference (IV, Fixed, 95% CI)	Totals not selected

**Comparison 4:** Nicotine EC versus non‐nicotine EC

Outcome or subgroup title	No. of studies	No. of participants	Statistical method	Effect size
4.1 Smoking cessation	6	1613	Risk Ratio (M‐H, Fixed, 95% CI)	1.46 [1.09, 1.96]
4.2 Adverse events	5	840	Risk Ratio (M‐H, Fixed, 95% CI)	1.01 [0.91, 1.11]
4.2.1 1 week	1	48	Risk Ratio (M‐H, Fixed, 95% CI)	1.50 [0.27, 8.19]
4.2.2 8 weeks	1	24	Risk Ratio (M‐H, Fixed, 95% CI)	1.18 [0.38, 3.66]
4.2.3 12 weeks	1	255	Risk Ratio (M‐H, Fixed, 95% CI)	1.01 [0.94, 1.08]
4.2.4 6 months	2	513	Risk Ratio (M‐H, Fixed, 95% CI)	0.97 [0.71, 1.34]
4.3 Serious adverse events	9	1412	Risk Ratio (M‐H, Fixed, 95% CI)	1.00 [0.56, 1.79]
4.3.1 1 week	1	48	Risk Ratio (M‐H, Fixed, 95% CI)	Not estimable
4.3.2 4 weeks	1	74	Risk Ratio (M‐H, Fixed, 95% CI)	Not estimable
4.3.3 8 weeks	1	24	Risk Ratio (M‐H, Fixed, 95% CI)	3.50 [0.16, 78.19]
4.3.4 6 months	4	1009	Risk Ratio (M‐H, Fixed, 95% CI)	0.95 [0.52, 1.72]
4.3.5 1 year	2	257	Risk Ratio (M‐H, Fixed, 95% CI)	Not estimable
4.4 Carbon monoxide (ppm)	6		Mean Difference (IV, Fixed, 95% CI)	Totals not selected
4.4.1 Change from baseline	3		Mean Difference (IV, Fixed, 95% CI)	Totals not selected
4.4.2 Absolute values at follow‐up	3		Mean Difference (IV, Fixed, 95% CI)	Totals not selected
4.5 Heart rate	1		Mean Difference (IV, Fixed, 95% CI)	Totals not selected
4.5.1 Absolute values at follow‐up	1		Mean Difference (IV, Fixed, 95% CI)	Totals not selected
4.6 Systolic blood pressure	1		Mean Difference (IV, Fixed, 95% CI)	Totals not selected
4.6.1 Absolute values at follow‐up	1		Mean Difference (IV, Fixed, 95% CI)	Totals not selected
4.7 FeNO (ppb)	1		Mean Difference (IV, Fixed, 95% CI)	Totals not selected
4.7.1 Change from baseline	1		Mean Difference (IV, Fixed, 95% CI)	Totals not selected
4.8 FEV1 (l)	1		Std. Mean Difference (IV, Fixed, 95% CI)	Totals not selected
4.8.1 Absolute values at follow‐up	1		Std. Mean Difference (IV, Fixed, 95% CI)	Totals not selected
4.9 FEV1/FVC	1		Mean Difference (IV, Fixed, 95% CI)	Totals not selected
4.9.1 Absolute values at follow‐up	1		Mean Difference (IV, Fixed, 95% CI)	Totals not selected
4.10 NNAL (pmol/mg creatinine)	2	363	Mean Difference (IV, Fixed, 95% CI)	‐0.02 [‐0.45, 0.41]
4.10.1 Change from baseline	1	148	Mean Difference (IV, Fixed, 95% CI)	15.27 [‐4.98, 35.52]
4.10.2 Absolute values at follow‐up	1	215	Mean Difference (IV, Fixed, 95% CI)	‐0.03 [‐0.46, 0.40]
4.11 Product use at 6+ months	3	874	Risk Ratio (M‐H, Fixed, 95% CI)	1.15 [0.94, 1.41]

**Comparison 5:** Nicotine EC versus behavioural support only/no support

Outcome or subgroup title	No. of studies	No. of participants	Statistical method	Effect size
5.1 Smoking cessation	11	6819	Risk Ratio (M‐H, Fixed, 95% CI)	1.96 [1.66, 2.32]
5.2 Adverse events	6	2351	Risk Ratio (M‐H, Fixed, 95% CI)	1.18 [1.10, 1.27]
5.2.1 12 weeks	2	657	Risk Ratio (M‐H, Fixed, 95% CI)	1.20 [1.11, 1.30]
5.2.2 16 weeks	1	50	Risk Ratio (M‐H, Fixed, 95% CI)	1.18 [0.67, 2.07]
5.2.3 6 months	3	1644	Risk Ratio (M‐H, Fixed, 95% CI)	1.17 [1.05, 1.30]
5.3 Serious adverse events	12	4561	Risk Ratio (M‐H, Fixed, 95% CI)	0.93 [0.68, 1.28]
5.3.1 4 to 6 weeks	2	246	Risk Ratio (M‐H, Fixed, 95% CI)	Not estimable
5.3.2 8 weeks	1	240	Risk Ratio (M‐H, Fixed, 95% CI)	0.29 [0.06, 1.35]
5.3.3 12 weeks	2	858	Risk Ratio (M‐H, Fixed, 95% CI)	3.69 [0.21, 66.17]
5.3.4 16 weeks	1	50	Risk Ratio (M‐H, Fixed, 95% CI)	Not estimable
5.3.5 6 months	5	2875	Risk Ratio (M‐H, Fixed, 95% CI)	0.89 [0.62, 1.27]
5.3.6 8 months	1	292	Risk Ratio (M‐H, Fixed, 95% CI)	1.78 [0.68, 4.70]
5.4 Carbon monoxide (ppm)	11		Mean Difference (IV, Fixed, 95% CI)	Totals not selected
5.4.1 Change from baseline	2		Mean Difference (IV, Fixed, 95% CI)	Totals not selected
5.4.2 Absolute values at follow‐up	9		Mean Difference (IV, Fixed, 95% CI)	Totals not selected
5.5 Heart rate (bpm)	1		Mean Difference (IV, Fixed, 95% CI)	Totals not selected
5.5.1 Absolute values at follow‐up	1		Mean Difference (IV, Fixed, 95% CI)	Totals not selected
5.6 Systolic blood pressure	3	298	Mean Difference (IV, Fixed, 95% CI)	‐2.30 [‐3.91, ‐0.69]
5.6.1 Change from baseline	1	168	Mean Difference (IV, Fixed, 95% CI)	‐2.68 [‐4.38, ‐0.98]
5.6.2 Absolute values at follow‐up	2	130	Mean Difference (IV, Fixed, 95% CI)	1.11 [‐3.95, 6.18]
5.7 Blood oxygen saturation	1		Mean Difference (IV, Fixed, 95% CI)	Totals not selected
5.7.1 Absolute values at follow‐up	1		Mean Difference (IV, Fixed, 95% CI)	Totals not selected
5.8 3‐HPMA (SMD)	2		Std. Mean Difference (IV, Fixed, 95% CI)	Totals not selected
5.8.1 Absolute values at follow‐up	1		Std. Mean Difference (IV, Fixed, 95% CI)	Totals not selected
5.8.2 Change from baseline	1		Std. Mean Difference (IV, Fixed, 95% CI)	Totals not selected
5.9 NNAL (SMD)	5		Std. Mean Difference (IV, Fixed, 95% CI)	Totals not selected
5.9.1 Absolute values at follow‐up	2		Std. Mean Difference (IV, Fixed, 95% CI)	Totals not selected
5.9.2 Change from baseline	3		Std. Mean Difference (IV, Fixed, 95% CI)	Totals not selected
5.10 2‐HPMA (pmol/mg creatinine)	1		Mean Difference (IV, Fixed, 95% CI)	Totals not selected
5.10.1 Absolute values at follow‐up	1		Mean Difference (IV, Fixed, 95% CI)	Totals not selected
5.11 HMPMA (pmol/mg creatinine)	1		Mean Difference (IV, Fixed, 95% CI)	Totals not selected
5.11.1 Absolute values at follow‐up	1		Mean Difference (IV, Fixed, 95% CI)	Totals not selected
5.12 PheT (pmol/mg creatinine)	1		Mean Difference (IV, Fixed, 95% CI)	Totals not selected
5.12.1 Absolute values at follow‐up	1		Mean Difference (IV, Fixed, 95% CI)	Totals not selected
5.13 CEMA (pmol/mg creatinine)	1		Mean Difference (IV, Fixed, 95% CI)	Totals not selected
5.13.1 Absolute values at follow‐up	1		Mean Difference (IV, Fixed, 95% CI)	Totals not selected
5.14 AAMA (pmol/mg creatinine)	1		Mean Difference (IV, Fixed, 95% CI)	Totals not selected
5.14.1 Absolute values at follow‐up	1		Mean Difference (IV, Fixed, 95% CI)	Totals not selected
5.15 S‐PMA (nanograms)	1		Mean Difference (IV, Fixed, 95% CI)	Totals not selected
5.15.1 12 weeks	1		Mean Difference (IV, Fixed, 95% CI)	Totals not selected
5.16 FEV1 (SMD)	2	714	Std. Mean Difference (IV, Fixed, 95% CI)	0.15 [‐0.01, 0.31]
5.16.1 Change from baseline	2	714	Std. Mean Difference (IV, Fixed, 95% CI)	0.15 [‐0.01, 0.31]
5.17 FEF 25‐75 (litres/second))	2	555	Mean Difference (IV, Fixed, 95% CI)	‐0.06 [‐0.18, 0.06]
5.17.1 Change from baseline	2	555	Mean Difference (IV, Fixed, 95% CI)	‐0.06 [‐0.18, 0.06]
5.18 PEF 25‐75 (litres/minute)	1		Mean Difference (IV, Fixed, 95% CI)	Totals not selected
5.18.1 Change from baseline	1		Mean Difference (IV, Fixed, 95% CI)	Totals not selected
5.19 FEV1/FVC	1		Mean Difference (IV, Fixed, 95% CI)	Totals not selected
5.19.1 Change from baseline	1		Mean Difference (IV, Fixed, 95% CI)	Totals not selected

**Comparison 6:** Higher versus lower nicotine content

Outcome or subgroup title	No. of studies	No. of participants	Statistical method	Effect size
6.1 Smoking cessation	1		Risk Ratio (M‐H, Fixed, 95% CI)	Totals not selected
6.2 Adverse events at 1 week	1		Risk Ratio (M‐H, Fixed, 95% CI)	Totals not selected
6.3 Serious adverse events	2		Risk Ratio (M‐H, Fixed, 95% CI)	Totals not selected
6.3.1 1 year	1		Risk Ratio (M‐H, Fixed, 95% CI)	Totals not selected
6.3.2 6 months	1		Risk Ratio (M‐H, Fixed, 95% CI)	Totals not selected
6.4 Carbon monoxide (ppm)	3	348	Mean Difference (IV, Fixed, 95% CI)	‐0.92 [‐1.71, ‐0.13]
6.4.1 Change from baseline	2	309	Mean Difference (IV, Fixed, 95% CI)	‐0.90 [‐1.70, ‐0.10]
6.4.2 Absolute values at follow‐up	1	39	Mean Difference (IV, Fixed, 95% CI)	‐1.66 [‐6.65, 3.33]
6.5 Heart rate	1		Mean Difference (IV, Fixed, 95% CI)	Totals not selected
6.5.1 12 weeks	1		Mean Difference (IV, Fixed, 95% CI)	Totals not selected
6.6 Systolic blood pressure	1		Mean Difference (IV, Fixed, 95% CI)	Totals not selected
6.6.1 12 weeks	1		Mean Difference (IV, Fixed, 95% CI)	Totals not selected
6.7 FeNO (ppb)	1		Mean Difference (IV, Fixed, 95% CI)	Totals not selected
6.7.1 12 weeks	1		Mean Difference (IV, Fixed, 95% CI)	Totals not selected
6.8 FEV1 (l)	1		Mean Difference (IV, Fixed, 95% CI)	Totals not selected
6.8.1 12 weeks	1		Mean Difference (IV, Fixed, 95% CI)	Totals not selected
6.9 FVC (l)	1		Mean Difference (IV, Fixed, 95% CI)	Totals not selected
6.9.1 12 weeks	1		Mean Difference (IV, Fixed, 95% CI)	Totals not selected
6.10 FEV1/FVC	1		Mean Difference (IV, Fixed, 95% CI)	Totals not selected
6.10.1 12 weeks	1		Mean Difference (IV, Fixed, 95% CI)	Totals not selected
6.11 NNAL (pg/mg creatinine) at 24 weeks	1		Mean Difference (IV, Fixed, 95% CI)	Totals not selected
6.12 Product use at 6+ months	1		Risk Ratio (M‐H, Fixed, 95% CI)	Totals not selected

**Comparison 7:** Choice of flavours vs. tobacco flavour only

Outcome or subgroup title	No. of studies	No. of participants	Statistical method	Effect size
7.1 Smoking cessation	1	566	Risk Ratio (M‐H, Fixed, 95% CI)	0.80 [0.54, 1.16]
7.2 Product use at 6+ months	1	522	Risk Ratio (M‐H, Fixed, 95% CI)	1.10 [0.86, 1.40]

**Comparison 8:** Tobacco vs. menthol flavour

Outcome or subgroup title	No. of studies	No. of participants	Statistical method	Effect size
8.1 Serious adverse events	1		Risk Ratio (M‐H, Fixed, 95% CI)	Totals not selected
8.2 NNAL (ng/g)	1		Mean Difference (IV, Fixed, 95% CI)	Totals not selected
8.2.1 Change from baseline	1		Mean Difference (IV, Fixed, 95% CI)	Totals not selected
8.3 FEV1 (% predicted)	1		Mean Difference (IV, Fixed, 95% CI)	Totals not selected
8.3.1 Change from baseline	1		Mean Difference (IV, Fixed, 95% CI)	Totals not selected
8.4 FEV1/FVC	1		Mean Difference (IV, Fixed, 95% CI)	Totals not selected
8.4.1 Change from baseline	1		Mean Difference (IV, Fixed, 95% CI)	Totals not selected

**Comparison 9:** Refillable versus cartridge

Outcome or subgroup title	No. of studies	No. of participants	Statistical method	Effect size
9.1 Exhaled CO	1		Mean Difference (IV, Fixed, 95% CI)	Totals not selected

**Comparison 10:** Nicotine salt EC versus free‐base nicotine EC

Outcome or subgroup title	No. of studies	No. of participants	Statistical method	Effect size
10.1 Smoking cessation	1		Risk Ratio (M‐H, Fixed, 95% CI)	Totals not selected
10.2 Product use at 6+ months	1		Risk Ratio (M‐H, Fixed, 95% CI)	Totals not selected

**Comparison 11:** Non‐nicotine EC versus behavioural support only/no support

Outcome or subgroup title	No. of studies	No. of participants	Statistical method	Effect size
11.1 Smoking cessation	2	388	Risk Ratio (M‐H, Fixed, 95% CI)	1.63 [0.81, 3.25]
11.2 Adverse events at 12 weeks	1		Risk Ratio (M‐H, Fixed, 95% CI)	Totals not selected
11.3 Serious adverse events at 24 weeks	2		Risk Ratio (M‐H, Fixed, 95% CI)	Totals not selected

**Comparison 12:** Non‐nicotine EC + NRT versus NRT

Outcome or subgroup title	No. of studies	No. of participants	Statistical method	Effect size
12.1 Smoking cessation	1		Risk Ratio (M‐H, Fixed, 95% CI)	Totals not selected
12.2 Adverse events	1		Risk Ratio (M‐H, Fixed, 95% CI)	Totals not selected
12.3 Serious adverse events	1		Risk Ratio (M‐H, Fixed, 95% CI)	Totals not selected
12.3.1 6 months	1		Risk Ratio (M‐H, Fixed, 95% CI)	Totals not selected

**Comparison 13:** Non‐nicotine EC versus NRT

Outcome or subgroup title	No. of studies	No. of participants	Statistical method	Effect size
13.1 Smoking cessation	2	314	Risk Ratio (M‐H, Random, 95% CI)	0.99 [0.64, 1.54]
13.2 Adverse events	1		Risk Ratio (M‐H, Fixed, 95% CI)	Totals not selected
13.2.1 6 months	1		Risk Ratio (M‐H, Fixed, 95% CI)	Totals not selected
13.3 Serious adverse events	1	132	Risk Ratio (M‐H, Fixed, 95% CI)	Not estimable
13.3.1 6 months	1	132	Risk Ratio (M‐H, Fixed, 95% CI)	Not estimable
13.4 Change in carbon monoxide (ppm) at 6 months	1		Mean Difference (IV, Fixed, 95% CI)	Totals not selected

**Comparison 14:** Advice to use e‐cigarettes compared to no advice to use e‐cigarettes

Outcome or subgroup title	No. of studies	No. of participants	Statistical method	Effect size
14.1 Smoking cessation	2	2652	Risk Ratio (M‐H, Fixed, 95% CI)	1.02 [0.88, 1.19]
14.2 Adverse events at 3 months	1		Risk Ratio (M‐H, Fixed, 95% CI)	Totals not selected
14.3 Serious adverse events at 3 months	1		Risk Ratio (M‐H, Fixed, 95% CI)	Totals not selected
14.4 Product use at 6+ months	1		Risk Ratio (M‐H, Random, 95% CI)	Totals not selected

**Comparison 15:** Nicotine EC + NRT versus non‐nicotine EC + NRT

Outcome or subgroup title	No. of studies	No. of participants	Statistical method	Effect size
15.1 Smoking cessation	2	1039	Risk Ratio (M‐H, Fixed, 95% CI)	1.77 [1.07, 2.94]
15.2 Adverse events	2	677	Risk Ratio (M‐H, Fixed, 95% CI)	1.11 [0.93, 1.32]
15.2.1 8 weeks	1	70	Risk Ratio (M‐H, Fixed, 95% CI)	1.25 [0.78, 1.99]
15.2.2 12 weeks	1	607	Risk Ratio (M‐H, Fixed, 95% CI)	1.09 [0.90, 1.31]
15.3 Serious adverse events	2	1069	Risk Ratio (M‐H, Fixed, 95% CI)	0.66 [0.38, 1.14]
15.3.1 8 weeks	1	70	Risk Ratio (M‐H, Fixed, 95% CI)	0.59 [0.11, 3.34]
15.3.2 6 months	1	999	Risk Ratio (M‐H, Fixed, 95% CI)	0.67 [0.37, 1.19]
15.4 Carbon monoxide (ppm)	2	70	Mean Difference (IV, Fixed, 95% CI)	‐2.58 [‐5.21, 0.05]
15.4.1 change from baseline	2	70	Mean Difference (IV, Fixed, 95% CI)	‐2.58 [‐5.21, 0.05]
15.5 FeNO (ppb)	1		Mean Difference (IV, Fixed, 95% CI)	Totals not selected
15.5.1 6 months	1		Mean Difference (IV, Fixed, 95% CI)	Totals not selected
15.6 FEV1 (%)	1		Mean Difference (IV, Fixed, 95% CI)	Totals not selected
15.6.1 6 months	1		Mean Difference (IV, Fixed, 95% CI)	Totals not selected
15.7 FVC (%)	1		Mean Difference (IV, Fixed, 95% CI)	Totals not selected
15.7.1 6 months	1		Mean Difference (IV, Fixed, 95% CI)	Totals not selected
15.8 Study product use at 6+ months	1		Risk Ratio (M‐H, Fixed, 95% CI)	Totals not selected

**Comparison 16:** Nicotine EC + NRT versus NRT

Outcome or subgroup title	No. of studies	No. of participants	Statistical method	Effect size
16.1 Smoking cessation	2	980	Risk Ratio (M‐H, Fixed, 95% CI)	3.53 [1.93, 6.44]
16.2 Adverse events	3	1984	Risk Ratio (M‐H, Fixed, 95% CI)	0.96 [0.83, 1.11]
16.2.1 12 weeks	2	421	Risk Ratio (M‐H, Fixed, 95% CI)	0.88 [0.69, 1.11]
16.2.2 7 months	1	1563	Risk Ratio (M‐H, Fixed, 95% CI)	0.99 [0.82, 1.19]
16.3 Serious adverse events	4	2245	Risk Ratio (M‐H, Fixed, 95% CI)	1.26 [0.46, 3.42]
16.3.1 5 weeks	1	7	Risk Ratio (M‐H, Fixed, 95% CI)	Not estimable
16.3.2 12 weeks	1	50	Risk Ratio (M‐H, Fixed, 95% CI)	3.00 [0.13, 70.30]
16.3.3 6 months	1	625	Risk Ratio (M‐H, Fixed, 95% CI)	1.12 [0.39, 3.27]
16.3.4 7 months	1	1563	Risk Ratio (M‐H, Fixed, 95% CI)	Not estimable

**Comparison 17:** Nicotine EC + varenicline vs. varenicline

Outcome or subgroup title	No. of studies	No. of participants	Statistical method	Effect size
17.1 Adverse events at 12 weeks	1		Risk Ratio (M‐H, Fixed, 95% CI)	Totals not selected
17.2 Serious adverse events at 12 weeks	1		Risk Ratio (M‐H, Fixed, 95% CI)	Totals not selected

## Characteristics of studies

### Characteristics of included studies [ordered by study ID]

**Table:** Adriaens 2014

***Study characteristics***		
Methods	Design: 3‐armed RCT; with all participants then assigned to nicotine EC (treated as cohort in this review) Recruitment: advertisement on university website, flyers on university campuses, emails to personnel, and advertisement in local newspaper Setting: community and laboratory, Belgium Study start date/end date: not stated	
Participants	Total N: 48 provided data Randomized to: EC1 16; EC2 17; control 17 Inclusion criteria: smoke ≥ 3 yrs; ≥ 10 cpd; not intending to quit in the near future but willing to try a less unhealthy alternative Exclusion criteria: diabetes; severe allergies; asthma or other respiratory diseases; psychiatric problems; dependence on chemicals other than nicotine; pregnancy; breastfeeding; hypertension; CV disease; currently using any kind of smoking cessation therapy; prior use of EC 56% women, mean age 44, mean cpd 19, mean FTCD 5.79, all unwilling to quit with no baseline EC use	
Interventions	**EC: Refillable** **Intervention:** 2 intervention groups (EC1 and EC2) provided with EC and instructed to use EC or smoke ad libitum (EC1 group provided with Joyetech eGO‐C, EC2 group provided with Kanger T2‐CC) and provided guidance on EC use. For both types, provided 30 mL bottles of tobacco‐flavoured e‐liquid (Dekang “Turkish Blend”), containing 18 mg/mL of nicotine. 4 bottles at baseline replenished at 4 weeks, keep any remaining after 8 weeks **Control:** 6 bottles for 2 months at week 8 (half offered EC1, half offered EC2); no guidance on use	
Outcomes	3 lab sessions over 2 months (weeks 1, 4, and 8), plus online questionnaires, further follow‐up at 3 and 6 m after last lab session Cessation: measured but definition not provided, validated with eCO 5 ppm or less Adverse events and biomarkers: eCO, salivary cotinine measured during lab sessions. Also collected craving and withdrawal symptoms via lab sessions, “benefits and complaints”, mood, EC usage	
Study funding	"No external funding for this study was obtained. Electronic cigarettes and e‐liquids were purchased at E‐cig4U (`t Rond 10, 4285 DE Woudrichem, The Netherlands; http://www.e‐cig4u.nL) with balances of previous research funds obtained by Frank Baeyens."	
Author declarations	The authors declare no conflict of interest.	
Notes	Randomization was for short‐term outcomes only. Additional data provided from authors	
***Risk of bias***		
**Bias**	**Authors' judgement**	**Support for judgement**
Random sequence generation (selection bias)	Low risk	Block randomization was performed by using a randomization tool available on the website www.randomizer.org.
Allocation concealment (selection bias)	Unclear risk	Not specified
Blinding of participants and personnel (performance bias) All outcomes	Low risk	Unblinded but as this review only included data on objective measurements and not cessation, judged unlikely to affect outcomes
Blinding of outcome assessment (detection bias) All outcomes	Low risk	Unblinded but as this review only included data on objective measurements and not cessation, judged unlikely to affect outcomes
Incomplete outcome data (attrition bias) All outcomes	Low risk	36 out of 48 completed follow‐up (11/16 in EC1 group, 12/17 in EC2 group, 13/17 in control group)
Selective reporting (reporting bias)	Unclear risk	Outcome reporting somewhat non‐traditional; for example, collecting complaints but not explicitly adverse events, and incidence of AEs not reported. Unable to find prospectively registered protocol

**Table:** Auer 2024

***Study characteristics***		
Methods	Randomized, parallel‐assignment, open‐label trial Setting: 5 sites in Switzerland: Bern, Geneva, Lausanne, St Gallen, Zurich Recruitment: free and paid advertisements (lay press, social media, healthcare facilities, on public transport) Study July 2018 to June 2021 (NCT record April 2022)	
Participants	Total: 1246 (intervention group 622, control group 624) 47% female; mean age 41.1 (median age 38 (IQR 29 to 51)); median cigarettes per day 15 (IQR 10 to 20); FTND 4.3 (SD 2.3) Inclusion criteria: Informed consent as documented by signature Persons aged 18 or older Currently smoking 5 or more cigarettes a day for at least 12 months Willing to try to quit smoking within the next 3 months Persons providing a valid phone number, a valid email address, and/or a valid postal address Exclusion criteria: Known hypersensitivity or allergy to contents of the e‐liquid Participation in another study with investigational drug within the 30 days preceding the baseline visit and during the present study where interactions are to be expected Women who are pregnant or breastfeeding Intention to become pregnant during the course of the scheduled study intervention, i.e. within the first 6 months of the study Persons having used ENDS regularly in the 3 months preceding the baseline visit Persons having used nicotine replacement therapy (NRT) or other medications with demonstrated efficacy as an aid for smoking cessation such as varenicline or bupropion within the 3 months preceding the baseline visit Persons who cannot attend the 6‐month follow‐up visit for any reason Cannot understand instructions delivered in person or by phone, or otherwise unable to participate in study procedures E‐cigarette use at baseline: not using e‐cigarette regularly in last 3 months. 15.4% of the control and 17.2% of the intervention group had previously used an e‐cigarette. Motivated to quit: ‘wanted to set a quit date’. Eligible participants asked to set a quit date.	
Interventions	a) ENDS (vaporizer/e‐cig) and smoking cessation counselling will receive: ENDS and nicotine‐containing e‐liquids, which they will be allowed to use ad libitum. Two e‐cigarette starter kits (Innokin Endura T20‐S) and 5 spare 0.8‐ohm coils. Participants could choose among 6 flavours (2 tobacco, 1 menthol, and 3 fruity) and 4 nicotine concentrations (19.6 mg, 11 mg, 6 mg, and 0 mg per millilitre). Trial nurses gave participants no more than 10 e‐liquid bottles at the baseline visit. Smoking cessation counselling: provided in person at the first clinical visit and then over the phone at the target quit date 1 week later and again at weeks 2, 4, and 8 after the target quit date. After 6 months, participants will be asked to come to a final clinical visit. Participants will be allowed to additionally use nicotine replacement therapy. NRT is not free. b) Control group will receive smoking cessation counselling only as provided for a). Participants will be allowed to additionally use nicotine replacement therapy.	
Outcomes	Baseline and 6 months Primary outcome: continuous smoking abstinence at 6 months post‐quit date measured by: Self‐report of having smoked no cigarettes from quit date, validated by urinary levels of anabasine. If anabasine is missing, validation by exhaled carbon monoxide (CO) Secondary outcomes: Continuous smoking abstinence at 6 months post‐quit date Self‐report of having smoked no cigarettes from quit date, validated by urinary levels of NNAL (4‐(methylnitrosamino)‐1‐(3‐pyridyl)‐1‐butanol). If NNAL is missing, validation by urinary levels of anabasine or exhaled carbon monoxide (CO). Self‐reported smoking abstinence allowing a 2‐week grace period at 4, 8 weeks, and 6 months post‐quit date. Validated smoking abstinence allowing a 2‐week grace period at 6 months post‐quit date: validated by urinary levels of anabasine; if anabasine is missing validation by exhaled CO; validated by urinary levels of NNAL (4‐(methylnitrosamino)‐1‐(3‐pyridyl)‐1‐butanol); if NNAL is missing, validation by urinary levels of anabasine or exhaled CO. Self‐reported smoking abstinence allowing up to 5 cigarettes at 1, 2, 4, 8 weeks, and 6 months post‐quit date. Validated smoking abstinence allowing up to 5 cigarettes at 6 months post‐quit date: validated by urinary levels of anabasine; if anabasine is missing validation by exhaled CO; validated by urinary levels of NNAL (4‐(methylnitrosamino)‐1‐(3‐pyridyl)‐1‐butanol); if NNAL is missing, validation by urinary levels of anabasine or exhaled CO. Self‐reported 7‐day PPA at 1, 2, 4, 8 weeks, and 6 months post‐quit date. Validated 7‐day PPA at 6 months post‐quit date Confirmation of having smoked no cigarettes in the past 7 days, validated by urinary levels of anabasine. If anabasine is missing validation by exhaled CO. Confirmation of having smoked no cigarettes in the past 7 days, validated by urinary levels of NNAL (4‐(methylnitrosamino)‐1‐(3‐pyridyl)‐1‐butanol). If NNAL is missing, validation by urinary levels of anabasine or exhaled CO. Number of cpd at baseline, target quit date, 1, 2, 4, 8 weeks, and 6 months post‐quit date, self‐reported. Change in number of cpd at baseline, 6 months post‐quit date, self‐reported. Successful reduction defined as 50% reduction in cpd. Use of any other smoking cessation products (NRT) at 1, 2, 4, 8 weeks, and 6 months post‐quit date, self‐reported. Withdrawal at baseline and 6 months assessed with the Minnesota Tobacco Withdrawal Scale (MTWS‐R). Fagerström Test for Nicotine Dependence at baseline and 6 months. Swiss EQ‐5D at baseline and 6 months. Use of any ENDS at 1, 2, 4, 8 weeks, and 6 months post‐quit date, self‐reported. Respiratory symptoms (COPD Assessment Test). AEs defined as any untoward medical occurrence in participants who are administered an intervention during clinical investigation; these events were not necessarily caused by the intervention. Study nurses proactively collected AEs at each FU visit; participants could report AEs to the research team at any time. SAE defined as any event that: required inpatient treatment of at least 24 hours or extended a current hospital stay; caused permanent or significant incapacity or disability; was life‐threatening or caused death; caused a congenital anomaly or birth defect. Collected proactively at each FU visit. Participants could report SAEs at any time. Study product use baseline to 6‐month FU.	
Study funding	For Auer 2024: Supported by a grant (173552) from the Swiss National Sci[1]ence Foundation, a grant (19.017477) from the Swiss Tobacco Prevention Fund, a grant (KFS4744‐02‐2019) from Swiss Cancer Research, and Lunge Zürich.	
Author declarations	The funding bodies had no role in the trial design; the collection, monitoring, analysis, or interpretation of the data; or the writing of the manuscript. There was no industry involvement in the trial. Reto Auer, Stephanie Baggio, Florent Baty, Aurelie Berthet, Philip Briggmann, Rodrigo casagrande Tango, Martin Feller, Anja Frei, Moa Haller, Nancy Hopf, Jean‐Paul Humair,Isabelle Jacot Sadowski, Julian Jakob, Nicolas Rodondi, Nicolas Sambiagio, Anna Schoeni, Alexandra Strassmann, Mirah Stuber, Kali Tal do not have any interests to disclose. Ivan Berlin: speaker at meetings (Pfizer). Member DSMB phase II trial Kinnov therapeutics. Martin Brutsche: invited scientific talks (Astra Zeneca Schweiz). Scientific advisory board GlaxoSmithKline. Head of Department Kantonsspital. Consultant Merck Sharp & Dohme. Consultant Novartis Pharma AG. See: https://www.nejm.org/doi/suppl/10.1056/NEJMoa2308815/suppl_file/nejmoa2308815_disclosures.pdf	
Notes	Linked trial registry IDs: NCT03603340; NCT03603353; NCT03612336; NCT03612375; NCT03612453; NCT03612544; NCT03632421; NCT03938298. Based on the common study name ESTxENDS study, we assume that the listed trial IDs and the numerous listed references are linked to one study. However, limited information is currently available and we will revisit this as necessary as more information becomes available. The abstract reports "participants in the control group will receive smoking cessation counselling only. Participants will be allowed to additionally use NRT." As NRT was not provided by the study, we classed this comparator arm as "behavioural support only."	
***Risk of bias***		
**Bias**	**Authors' judgement**	**Support for judgement**
Random sequence generation (selection bias)	Low risk	An automated, centralized, online randomization system in a protected environment at the Clinical Trials Unit in Bern, Switzerland, then generated randomization sequences in a 1:1 ratio.
Allocation concealment (selection bias)	Low risk	An automated, centralized, online randomization system in a protected environment at the Clinical Trials Unit in Bern, Switzerland, then generated randomization sequences in a 1:1 ratio.
Blinding of participants and personnel (performance bias) All outcomes	High risk	Blinding was not possible as a placebo was not provided in the comparator group
Blinding of outcome assessment (detection bias) All outcomes	Low risk	Cessation biochemically verified
Incomplete outcome data (attrition bias) All outcomes	Low risk	Data on smoking status and serious adverse events at 6 months were available for 90.8% of participants. Less than 20% difference and less than 50% attrition
Selective reporting (reporting bias)	Low risk	Outcomes reported

**Table:** Baldassarri 2018

***Study characteristics***		
Methods	Design: randomized, parallel‐assignment, double‐blind trial Recruitment: outpatient pulmonary and primary care clinics, Tobacco Treatment Service, referrals from medical providers Setting: Hospital outpatient and primary care clinics, USA Study start date: October 2014; Study end date: June 2014	
Participants	Total N: 40 N per arm: non‐nicotine: 20; nicotine EC: 20 Inclusion criteria: ≥ 18 years; ≥ 1 cpd; willing to quit smoking Exclusion criteria: unstable psychiatric or medical conditions requiring hospitalization within the past 4 months; acute coronary syndromes or stroke within the past 30 days; history of allergic reactions to adhesives; women who were pregnant, nursing, or not practising effective contraception; current use of an EC for the purpose of stopping tobacco cigarette smoking Women: 52.5%; mean age: 53 mean cpd: 17 mean FTND: 5.9; motivated to quit E‐cigarette use at baseline: not reported	
Interventions	**EC: Refillable** Both groups received standard care (8 weeks nicotine patch and counselling) and were randomized to **nicotine EC** or **non‐nicotine EC**. EC: eGO style EC (650 mAh battery, EVOD clearomizer, 3.7 V, 1.8 Ω single bottom coil), provided with e‐liquid purchased from an online vape shop (0 mg/mL or 24 mg/mL nicotine strength, 70/30 propylene glycol/vegetable glycerin, tobacco flavour); instructed to use it as needed as a substitute for tobacco to try to satisfy cravings to smoke. If the patch alone proved adequate to prevent withdrawal and smoking cravings, the participant was advised not to use the EC. Additional EC devices, replacement coils, and liquid were provided as needed for the first 8 weeks of the study.	
Outcomes	Questionnaires and CO measurements taken at baseline, treatment visits at week 2, 4, 6, 8, and follow‐up at week 24 Cessation: 7‐day point prevalence abstinence, eCO ≤ 6 ppm Adverse events and biomarkers: side effects were measured, although it is unclear whether a questionnaire with prespecified symptoms was used Spirometry and FeNO at baseline and 6‐month follow‐up Other outcomes: change in reported number of cpd at weeks 8 and 24; change in per cent predicted FEV1 and FVC from baseline to week 24, and EC use patterns	
Study funding	"Funding for this study was provided by the Yale School of Medicine, Section of Pulmonary, Critical Care, and Sleep Medicine and the National Heart, Lung, and Blood Institute grant T32HL007778. NHLBI had no role in the study design, collection, analysis, or interpretation of the data, writing the manuscript, or the decision to submit the paper for publication."	
Author declarations	"Dr. Toll received a grant from Pfizer for medicine only for a research study, and he receives funding as an expert witness in litigation filed against the tobacco industry. Dr. Chupp received grants from NIH, Genetech, Glaxo Smith Kline, Astra Zeneca/Medimmune and Boston Scientific. He received consulting/speaking fees from Genetech, Astra Zeneca/Medimmune, Mannkind, and Boston Scientific. There are no other conflicts of interest for the remaining authors."	
Notes	New for 2020 update. Study listed as ongoing study NCT02498145 in 2016 review update Additional data provided from authors	
***Risk of bias***		
**Bias**	**Authors' judgement**	**Support for judgement**
Random sequence generation (selection bias)	Low risk	Quote: “Participants were randomized using a random number generator with 1:1 blocked randomization (block size n = 8).”
Allocation concealment (selection bias)	Unclear risk	Both groups received standard care (nicotine patch and counselling) and were randomized to: nicotine EC or non‐nicotine EC (no further detail given).
Blinding of participants and personnel (performance bias) All outcomes	Low risk	Quote: “Treatment assignment was blinded to both the investigators and participants”.
Blinding of outcome assessment (detection bias) All outcomes	Low risk	CO biochemically validated
Incomplete outcome data (attrition bias) All outcomes	High risk	Quote: “The study had a modest loss to follow‐up (20%) at week 24.” Number lost to follow‐up in each group was not reported in the paper. Week 24 retention rate: Nicotine EC group: 19/20 (95%); non‐nicotine EC group: 13/20 (65%); > 20% difference between groups
Selective reporting (reporting bias)	Low risk	Outcomes reported align with those listed in the clinicaltrials.gov record (registered 2015; prior to study completion in 2016).

**Table:** Begh 2021

***Study characteristics***		
Methods	Individually randomized, blinded, 2‐arm trial Setting: 39 general practices, England Recruitment: primary care registries	
Participants	325 (164 intervention; 161 intervention) 47.4% female; mean age 57.8; mean cpd 20.1; mean FTCD 4.2 Inclusion criteria: current smoker ≥ 10 ppm for exhaled CO and smokes a minimum of 8 cigarettes/8 grams of tobacco per day (including pipe, cigars, or tobacco roll‐ups) with no intention of stopping immediately or seeking cessation support. Diagnosed with 1 or more of the following chronic conditions: ischaemic heart disease, peripheral vascular disease, hypertension, diabetes mellitus (Type 1 and Type 2), stroke, asthma, COPD, chronic kidney disease, depression, schizophrenia, bipolar disorder or other psychoses. Informed consent. ≥ 18 years Exclusion criteria: GP believes that switching to EC would not benefit the patient, given their current medical condition; currently using EC, NRT or other cessation therapies (e.g. bupropion, nortriptyline, or varenicline); plans to stop smoking before or at the annual review; currently enrolled in another smoking‐related study or other study where the aims of the studies are incompatible; cannot consent due to mental incapacity; pregnancy, breastfeeding	
Interventions	EC type: refillable Control: no additional support beyond standard care Intervention: practitioners gave brief advice about EC and offered participants a free EC for the purpose of switching from smoking to vaping. The instruction was to reduce their smoking. If the offer was accepted, participants received: a starter pack containing an Aspire PockeX all‐in‐one e‐cigarette, 2 x 0.6 ohm coils and 1 x 1.2 ohm coil, 3 nicotine e‐liquids in 18 mg/mL (blueberry, menthol) and 12 mg/mL (mixed fruit) strengths and an accompanying practical support booklet developed by the study team. The practical support booklet contained information on how to set up the device, correct ways to vape, common issues with use and a list of local vape shops. It included motivational support to reinforce practitioners’ advice about EC, including the benefits of cutting down on cigarettes through e‐cigarette use and addressing perceived risks and concerns. It included links to a study‐dedicated website with video demonstrations on how to use EC and testimonials. Participants could opt into receiving an introductory telephone call from an experienced vaper in the first week of receiving their EC, to guide them on technical aspects of EC use (not behavioural support). Thereafter, participants could contact the vaper by telephone for up to 2 months after receiving their kit. All: practitioners offered routine smoking cessation support to all participants. Although this varied across practices, standard care typically involved brief advice about stopping smoking and assistance to do so either by referral to the NHS stop‐smoking services or offer of pharmacotherapy. If the participant declined standard care, they were randomized by the practitioner to either the intervention or control arm. In the control arm, participants received no further support beyond standard care.	
Outcomes	0 months, consultation visit, 2 months post‐consultation, 8 months post‐consultation "Patients attended four visits at their GP practice: a baseline visit, a therapeutic visit (‘annual review’) with their GP or nurse and two follow‐up visits two months and eight months post‐consultation." Primary outcomes: 7‐day PPA from smoked tobacco at 2 months, defined as complete self‐reported abstinence from smoking – not even a puff – in the past 7 days, accompanied by a salivary anabasine concentration of < 1 ng/mL If there are technical issues with the analysis of saliva samples (e.g. if there is not enough saliva present in the sample for anabasine analysis), we will use exhaled CO as verification of abstinence (CO < 10 ppm). (Deviation from Statistical Analysis Plan: CO used due to imprecision of values for anabasine) Reduction in cigarette consumption at 2 months, defined as at least a 50% reduction in self‐reported cigarettes per day on each of the last 7 days at 2 months compared with baseline consumption, accompanied by evidence of reduced smoke intake indicated by a CO measurement lower than baseline Secondary outcomes: 7‐day PPA measured at 8 months, biochemically confirmed by an exhaled CO of < 10 ppm 6‐month prolonged abstinence using the Russell standard criteria, defined as smoking < 5 cigarettes between 2‐ and 8‐month follow‐ups, confirmed by an anabasine concentration of < 1 ng/mL at 2 months and an exhaled CO concentration of < 1 ng/mL at 8 months if CO measurement unavailable Mean change in salivary anabasine concentration and CO from baseline to 2 months Percentage reduction in self‐reported cigarettes per day from baseline to 2 months; and from baseline to 8 months SAEs and AEs reported. AEs: throat/mouth irritation; cough; headache; palpitations; nausea; dry mouth; dizziness; shortness of breath; stomach pain	
Study funding	NIHR Postdoctoral Fellowship and NIHR School for Primary Care Research funded randomized controlled trial	
Author declarations	All authors declare no competing interests.	
Notes		
***Risk of bias***		
**Bias**	**Authors' judgement**	**Support for judgement**
Random sequence generation (selection bias)	Low risk	Quote: "Participants were randomised to intervention or control with a 1:1 allocation ratio. A randomisation list was generated by the trial statistician using the current version of Stata and validated by a second statistician within the Primary Care Clinical Trials Unit (PC‐CTU). The randomisation was stratified by practice and used varying block sizes to ensure allocation concealment."
Allocation concealment (selection bias)	Low risk	Quote: "The randomisation list was passed to someone independent of the trial who created the randomisation envelopes. The trial statisticians were blinded to the treatment allocation during analyses."
Blinding of participants and personnel (performance bias) All outcomes	High risk	Due to the nature of the trial, GPs and practice nurses were aware of the participant’s treatment allocation to ensure that the correct intervention was given. Therefore, practitioners who delivered the intervention could not be blinded to treatment. While participants knew whether they had been offered support to cut down by using an e‐cigarette or not by their GP or nurse, the participant was not informed that the study investigated this specifically and, therefore, was in some respects blind to allocation. Groups not matched for face‐to‐face contact time
Blinding of outcome assessment (detection bias) All outcomes	Low risk	Quote: “7‐day point‐prevalence abstinence from smoked tobacco at two months, defined as complete self‐reported abstinence from smoking – not even a puff – in the past seven days, accompanied by a salivary anabasine concentration of < 1 ng/mL” or exhaled CO as verification of abstinence (CO < 10 ppm)"
Incomplete outcome data (attrition bias) All outcomes	Low risk	At 8 months: control 144/161; intervention 148/164
Selective reporting (reporting bias)	Low risk	Used CO above anabasine, but reported both (Deviation from Statistical Analysis Plan: CO used due to imprecision of values for anabasine) All predefined outcomes listed in the published protocol and clinical trial register were reported.

**Table:** Bonafont Reyes 2022

***Study characteristics***		
Methods	Design: "A mixed methods study" Recruitment: recruited patients with COPD, aged 21 to 75, listed as current smokers in the NYU Langone Health electronic health record by phone, mail, and MyChart Participants: patients with COPD Setting: NYU, USA Study start date: not reported	
Participants	Total N: 48 Inclusion criteria: moderate COPD (based on the COPD Assessment Test score (CAT)); interested in quitting Exclusion criteria: not reported Female 54%; mean age 60 (SD 8.2) E‐cigarette use at baseline: not reported Motivated to quit: yes	
Interventions	EC: no detail reported Arm 1 EC Arm 2 NRT Both groups: over 12 weeks, participants received 5 counselling sessions and were asked about their COPD symptoms, CC use, EC use, and nicotine withdrawal symptoms. We used Ecological Momentary Assessment (four text messages/day) to assess current EC/NRT and CC use.	
Outcomes	12 weeks Combustible cigarette use measured Dyspnoea COPD symptoms Mixed methods study assessing the relationship between race/ethnicity and switching from CC to EC; evaluated whether it is mediated by social norms, risk perception, and overall opinions of CC and EC	
Study funding	Not reported	
Author declarations	Not reported	
Notes	Student presentation New to 2022 update	
***Risk of bias***		
**Bias**	**Authors' judgement**	**Support for judgement**
Random sequence generation (selection bias)	Unclear risk	No detail: “We randomized participants to EC or nicotine replacement therapy (NRT) for switching from CC.”
Allocation concealment (selection bias)	Unclear risk	No detail
Blinding of participants and personnel (performance bias) All outcomes	Unclear risk	Two active interventions. No detail
Blinding of outcome assessment (detection bias) All outcomes	Unclear risk	No detail
Incomplete outcome data (attrition bias) All outcomes	Unclear risk	Still collecting data. Outcome data not reported. Numbers not reported
Selective reporting (reporting bias)	Unclear risk	Still collecting data

**Table:** Bullen 2013

***Study characteristics***		
Methods	Design: 3 parallel groups RCT Recruitment: people who smoke recruited from the community, via newspaper advertisements Setting: research unit, New Zealand Study start date: 6 September 2011; study end date: 5 July 2013	
Participants	Total N: 657. 289 nicotine EC (NEC), 295 patch, 73 non‐nicotine EC (PEC) Inclusion criteria: ≥ 18 years; smoked 10 or more cpd over past year; wanted to stop smoking Exclusion criteria: pregnancy and breastfeeding; using cessation medicines or using other support to quit; heart attack, stroke, severe angina in the last 2 weeks; poorly controlled medical disorder; allergies, other chemical dependence 62% women, mean age 42, ⅓ NZ Maori, smoking 18 cpd, mean FTND score 5.5 Motivated to quit E‐cigarette use at baseline: not specified	
Interventions	**EC: Cig‐a‐like** Randomized to NEC, PATCH, or PEC use for 13 weeks (from 1 week prior to TQD) **NEC:** Elusion brand 16 mg cartridges; sent product via courier **PATCH:** 21 mg/24‐hour patch; sent voucher to exchange for NRT at pharmacy (dispensing costs covered) **PEC:** as per EC, but 0 mg cartridges All participants referred to Quitline and received an invitation to access phone‐ or text‐based support. This was accessed by ≤ 40% of participants.	
Outcomes	Sustained (≤ 5 cigarettes allowed) validated (exhaled breath CO < 10 ppm) abstinence at 6 months ≥ 50% self‐reported reduction in baseline cigarettes at 6 months Participants reporting any adverse events Proportion of AEs that were serious Proportion of unrelated AEs	
Study funding	Health Research Council of New Zealand	
Author declarations	"We declare that we have received no support from any companies for the submitted work and have no non‐financial interests that might be relevant to the submitted work. ML, via his company Health New Zealand, previously did research funded by Ruyan (an e‐cigarette manufacturer). CB and HM have done research on Ruyan e‐cigarettes funded by Health New Zealand, independently of Ruyan. HM has received honoraria for speaking at research symposia, has received benefits in kind and travel support from, and has provided consultancy to, the manufacturers of smoking cessation drugs. NW has provided consultancy to the manufacturers of smoking cessation drugs, received honoraria for speaking at a research meeting and received benefits in kind and travel support from a manufacturer of smoking cessation drugs. JW has provided consultancy to the manufacturers of smoking cessation medications."	
Notes	Accessed support: NEC: 115/289; PATCH: 106/295; PEC: 26/73	
***Risk of bias***		
**Bias**	**Authors' judgement**	**Support for judgement**
Random sequence generation (selection bias)	Low risk	Computerized block randomization
Allocation concealment (selection bias)	Low risk	Computerized via study statistician
Blinding of participants and personnel (performance bias) All outcomes	Low risk	NEC and PEC were blind to treatment condition in relation to one another. No blinding for NEC/PEC vs PATCH conditions, but as NEC and PATCH were both active treatments, performance bias judged unlikely
Blinding of outcome assessment (detection bias) All outcomes	Low risk	Biochemical validation used
Incomplete outcome data (attrition bias) All outcomes	Low risk	22% lost to follow‐up (all considered to be smoking). Patch group had a higher loss to follow‐up and withdrawal than EC (loss to follow‐up 17% NEC, 27% patches, 22% PEC). However, minimal differences in per‐protocol and ITT analyses
Selective reporting (reporting bias)	Low risk	All prespecified outcomes reported

**Table:** Caponnetto 2013a*

***Study characteristics***		
Methods	Design: 3‐arm, double‐blind randomized controlled trial: EC with 7.2 mg nicotine for 12 weeks; same for 6 weeks followed by 5.2 mg for 6 weeks: EC with no nicotine for 12 weeks Recruitment: newspaper advertisements Setting: outpatient clinic, Italy Study start date: April 2010; Study end date: April 2012	
Participants	Total N: 300 Inclusion criteria: smoked ≥ 10 cpd for past 5 years; age 18 to 70; in good health; not currently or intending to quit smoking in the next 30 days Exclusion criteria: symptomatic cardiovascular or respiratory disease; regular psychotropic medicine use; current or past history of alcohol abuse; use of smokeless tobacco or NRT; pregnancy or breastfeeding 36% women; mean age 44 (SD 12.5); mean cpd 20 (IQR: 15 to 25) Not currently or intending to quit smoking in the next 30 days E cigarette use at baseline: not specified	
Interventions	**EC: Cig‐a‐like** EC presented as a healthier alternative to tobacco smoke and could be freely used, ad libitum (up to 4 cartridges a day) for 12 weeks, as a tobacco substitute EC used: 'Categoria' (model 401) with disposable cartridges **Grp A**: 12 weeks of 7.2 mg capsules ('Original') **Grp B**: 6 weeks 7.2 mg ('Original'), then 6 weeks 5.4 mg ('Categoria') **Grp C**: 12 weeks of 0 mg ('Original') Baseline visit and up to 7 follow‐up visits to receive more cartridges, hand‐in diaries, measure CO and vital signs	
Outcomes	Abstinence at 12 months (complete self‐reported abstinence from tobacco smoking since previous visit at 6 months, confirmed with CO < 7 ppm at 12 months) ≥ 50% reduction in baseline cigarettes at 12 months Recorded AEs thought to be related to tobacco smoking and EC at baseline and at each study visit (7 follow‐up visits over 12 weeks, plus at 24 and 52 weeks)	
Study funding	"This research was supported by a grant‐in‐aid from Lega Italiana AntiFumo. The study sponsor had no involvement in the study design, collection, analysis, and interpretation of data, the writing of the manuscript or the decision to submit the manuscript for publication. RP and PC are currently funded by the University of Catania, Italy. The e‐cigarette supplier had no involvement in the study design, collection, analysis, and interpretation of data, the writing of the manuscript or the decision to submit the manuscript for publication."	
Author declarations	"RP has received lecture fees and research funding from Pfizer and GlaxoSmithKline, manufacturers of stop smoking medications. He has served as a consultant for Pfizer and Arbi Group Srl, the distributor of the CategoriaTM e‐Cigarette. The other authors have no relevant conflict of interest to declare in relation to this work."	
Notes	Additional data provided from authors	
***Risk of bias***		
**Bias**	**Authors' judgement**	**Support for judgement**
Random sequence generation (selection bias)	Low risk	Computer‐generated, block size 15 (5:5:5 ratio)
Allocation concealment (selection bias)	Low risk	Randomization carried out by pharmacy, which did not have direct contact with the participants
Blinding of participants and personnel (performance bias) All outcomes	Low risk	Double‐blind Quote: “Blinding was ensured by the identical external appearance of the cartridges. The hospital pharmacy was in charge of randomization and packaging of the cigarettes”.
Blinding of outcome assessment (detection bias) All outcomes	Low risk	Biochemical validation used
Incomplete outcome data (attrition bias) All outcomes	Low risk	211 (70.3%) and 183 (61%) attended 6‐ and 12‐month follow‐up (at 12 m, 35% lost in 7.2 group; 37% lost in 5.4 group; 45% lost in no‐nicotine group).
Selective reporting (reporting bias)	Unclear risk	Unclear if original intention was to combine groups A + B or not. In sample size calculation, they compared A + B with C, but results were not always reported in this way.

**Table:** Caponnetto 2013b*

***Study characteristics***		
Methods	Design: prospective cohort Recruitment and setting: inpatients at a psychiatric institution in Italy Study start date/end date: not specified	
Participants	Total N: 14 Inclusion criteria: smoked ≥ 20 cpd for at least the past 10 years; diagnosis of schizophrenia Exclusion criteria: alcohol and illicit drug use; recent myocardial infarction; angina pectoris; high blood pressure (BP > 140 mmHg systolic or 90 mmHg diastolic, or both); diabetes mellitus; severe allergies; poorly controlled asthma or other airway diseases; inclusion based on specific population characteristic: diagnosis of schizophrenia 57% women; mean age 44.6 (SD 12.5); mean pack‐years smoked 28.8 (SD 12.9) Motivated to quit: not motivated to quit E cigarette use at baseline: not specified	
Interventions	**EC: Cig‐a‐like** Seen at baseline, given EC ('Categoria' brand) with an initial 4‐week supply of 7.4 mg nicotine cartridges. Instructed to use ad libitum up to 4 cartridges a day. EC cartridges supplied at months 1, 2, and 3 No instruction on cessation or reduction was provided.	
Outcomes	Follow‐up at 1, 2, 3, 6, and 12 months when cigarette consumption, CO, AEs, and positive and negative symptoms of schizophrenia were measured Sustained reduction of ≥ 50% for at least 30 days at 12 months 30‐day point prevalence CO‐validated abstinence at 12 months Adverse events	
Study funding	"We wish to thank Arbi Group Srl (Milano, Italy) for the free supplies of “Categoria” e‐cigarette kits and nicotine cartridges as well as their support. We would also like to thank LIAF (Lega Italiana AntiFumo) for the collaboration."	
Author declarations	"Pasquale Caponnetto, Roberta Auditore, Cristina Russo and Giorgio Carlo Cappello declare no conflict of interest. Riccardo Polosa has received lecture fees and research funding from Pfizer and GlaxoSmithKline, manufacturers of stop smoking medications. He has served as a consultant for Pfizer and Arbi Group Srl (Milano, Italy), the distributor of the CategoriaTM e‐cigarette."	
Notes		
***Risk of bias***		
**Bias**	**Authors' judgement**	**Support for judgement**
Random sequence generation (selection bias)	High risk	Prospective cohort; no randomization
Allocation concealment (selection bias)	High risk	Not randomized
Incomplete outcome data (attrition bias) All outcomes	Low risk	0/14 lost to follow‐up
Selective reporting (reporting bias)	Unclear risk	Unable to determine prespecified outcomes

**Table:** Caponnetto 2021*

***Study characteristics***		
Methods	Design: single‐arm pilot study Recruitment: authors wrote to physicians, psychiatrists, and other health care providers to inform them about the study. Flyers were posted within and outside of the Smoking Cessation Center of Catania University (Centro per la Prevenzione e Cura del Tabagismo — CPCT), at the Policlinico Vittorio Emanuele. Participants were recruited from Catania outpatient psychiatric clinics by researchers of CPCT. Clinicians from outpatient psychiatric clinics identified suitable participants and drew their attention to the study flyers. Setting: Catania, Italy Study start date: 2017. End date not stated. Recruitment September 2017 to October 2017	
Participants	Total N: 40 (single‐arm) All participants: individuals with schizophrenia spectrum disorders who smoke cigarettes Inclusion criteria: Adults attending psychiatric outpatient clinics in Catania who smoked 20 or more cigarettes daily were included. Able to meet the criteria for a schizophrenia spectrum disorder diagnosis without evidence of current exacerbation of illness Exclusion criteria: Pregnancy, breastfeeding, myocardial infarction, or angina pectoris within the past 3 months, current poorly controlled asthma or chronic obstructive pulmonary disease Female 35%; mean age 48.3 (SD 12.1); mean CPD 28 (SD 9); mean FTND 8.3 (SD 1.8) E‐cigarette use at baseline: 12 (30%) had used EC either regularly or had tried. Motivated to quit: no	
Interventions	EC: **pod** JUUL pod e‐cigarette, PAX Labs, a closed pod e‐cigarette product. The pod contains 0.7 mL of e‐liquid and up to 5% nicotine by weight. At the BL visit, participants were given a free e‐cigarette starter kit containing one JUUL device with a charger and 5% nicotine pods, Virginia tobacco flavour with instructions on how to charge, activate, and use the e‐cigarette. A 4‐week supply of pods equivalent to their current cigarette‐smoking behaviour, according to the manufacturer’s guidelines, was supplied to each participant (one pod for every packet of 20 cigarettes; mean 128, minimum 80, and maximum 200). Support: Eligible participants were invited to use a JUUL e‐cigarette for at least 12 weeks and were followed up prospectively for 24 weeks. Participants received a 4‐week supply of pods on three occasions, BL, week 4 (study visit 2), and week 8 (study visit 3). Participants were informed that the product was potentially less harmful than combustible cigarettes and could be used as a cigarette substitute as much as they liked. Limited behavioural support was provided as part of the intervention and included behaviour substitution of combustible cigarettes with e‐cigarettes and self‐monitoring of combustible cigarette consumption through the use of study diaries. Phone contact at week 2, 6, and 10. Participants attended a total of 5 study visits.	
Outcomes	Baseline, week 4 (study visit 2), week 8 (study visit 3), 12 weeks, 24‐week FU Cessation: CO Reduction: CPD AEs: dry cough, headache, throat irritation Other outcomes: vital signs (BP and HR), weight, and mental health, subjective effects (e.g. satisfied, no aversion), acceptability	
Study funding	The e‐cigarettes used in the study were donated by the manufacturer, PAX Labs (on June 13, 2017 the company became known as JUUL Labs). Acknowledgements: The authors wish also to thank PAX Labs (on June 13, 2017 the company became known as JUUL Labs) for the free supplies of JUUL e‐cigarette kits and pods. At the time the research was conducted, JUUL Labs were not part owned by Altria, a tobacco company. PAX Labs agreed also to supply pods for a further 3 months after the end of the pilot to participants who expressed a wish to continue using as JUUL was not available in Italy when this study was conducted and not currently available at the 5% nicotine strength. No separate funding was secured for the study. Altria Group (formerly Philip Morris Companies) acquired a 35% stake in JUUL Labs on December 20, 2018, but the study was completed before Altria invested in JUUL.	
Author declarations	MM is fixed‐term researcher at Centro per la Prevenzione e Cura del Tabagismo, University of Catania. JD is full‐time employee of City University of New York (United States). JK is full‐time employee of Weill Medical College of Cornell University, New York (United States). RP is full‐time employee of the University of Catania, Italy. In relation to his work in the area of tobacco control and respiratory diseases, RP has received lecture fees and research funding from Pfizer, Inc., GlaxoSmithKline plc, CV Therapeutics, NeuroSearch A/S, Sandoz, MSD, Boehringer Ingelheim, Novartis, Duska Therapeutics, and Forest Laboratories. He has also served as a consultant for Pfizer, Inc., Global Health Alliance for treatment of tobacco dependence, CV Therapeutics, NeuroSearch A/S, Boehringer Ingelheim, Duska Therapeutics, Forest Laboratories, ECITA (Electronic Cigarette Industry Trade Association, in the United Kingdom), Health Diplomat (consulting company that delivers solutions to global health problems with special emphasis on harm minimization), and Pharmacielo. RP was awarded an Investigator‐Initiated Study award programme established by Philip Morris International in 2017, but subsequently resigned from the role of Principal Investigator in 2018, before the trial began. Lecture fees from a number of European EC industry and trade associations (including Fédération Interprofessionnelle de la VAPE in France and Federazione Italiana Esercenti Svapo Elettronico in Italy) were directly donated to vaper advocacy no‐profit organizations. RP is the Founder of the Center of Excellence for the acceleration of Harm Reduction at the University of Catania (CoEHAR), which has received a grant from Foundation for a Smoke Free World to develop and carry out eight research projects. RP is also currently involved in the following pro bono activities: scientific advisor for LIAF, Lega Italiana Anti Fumo (Italian acronym for Italian Anti Smoking League) and Chair of the European Technical Committee for standardization on Requirements and test methods for emissions of electronic cigarettes (CEN/TC 437; WG4). PC is paid by the University of Catania as an external part‐time researcher and adjunct professor of clinical, addiction, and general psychology. He has been affiliated to the CoEHAR since December 2019 in a pro bono role. He is coauthor of a protocol paper supported by an Investigator‐Initiated Study award programme established by Philip Morris International in 2017. The other authors have no conflict of interests to declare.	
Notes	New to 2022 update	
***Risk of bias***		
**Bias**	**Authors' judgement**	**Support for judgement**
Random sequence generation (selection bias)	High risk	Single‐arm, open‐label
Allocation concealment (selection bias)	High risk	Single‐arm, open‐label
Incomplete outcome data (attrition bias) All outcomes	Low risk	Quote: 37 (92.5%) participants completed all study visits and attended their follow‐up visit.
Selective reporting (reporting bias)	Unclear risk	No detail

**Table:** Caponnetto 2023*

***Study characteristics***		
Methods	Design: randomized non‐inferiority switching trial Setting: Italy Recruitment: recruited among hospital/university staff, via social media, or through word of mouth Study start date: May 2019. Study end date May 2020	
Participants	220 healthy people who smoke tobacco cigarettes EC arm 110, HTP arm 110 % female: EC arm 70% (63.6), HTP arm 56% (50.9). Mean age 41.3 (EC arm 41.3 (SD 16.9), HTP arm 41.3 (SD 16.1)). Mean CPD: EC arm 22.8 (10.9), HTP arm 22.6 (SD 10.1). Mean FTND: EC arm 6 (SD 2.2), HTP arm 5.8 (SD 2.1) Motivated to quit: No	
Interventions	E‐cigarettes (EC) arm: JustFog Q16 Starter Kit consisting of a battery and a 1.9‐mL refillable tank fitted with a 1.6‐Ohm nichrome coil, 16 mg, 3 flavours 50% propylene glycol/40% vegetable glycerin/10% H2O. EC study arm, 50.9% (56/110) chose Puff Riserva Country, 30.9% (34/110) chose Puff Riserva Tuscan, and 18.2% (20/110) chose Puff Artic e‐liquid. Heated Tobacco Products (HTPs) arm: IQOS 2.4 Plus consisting of a pen‐like holder into which a tobacco stick is inserted and heated, and a battery case to recharge the holder after each use. IQOS 2.4 Plus was the only HTP available on the Italian market when this trial was designed. The device is to be used with tobacco sticks specifically processed and manufactured for IQOS (named HEETS). Participants could choose from 3 varieties of tobacco sticks (HEETS Amber, rich tobacco; HEETS Yellow, smooth tobacco; and HEETS Turquoise, menthol‐flavoured tobacco), which were available for sale on the Italian market at the time of the study. HTP study arm, 56.4% (62/110) chose HEETS Amber, 33.6% (37/110) chose HEETS Yellow, and 10.0% (11/110) chose HEETS Turquoise tobacco sticks. Both groups: “Motivational counseling was offered throughout the study to maximize study product adherence, to favor transition away from combustible tobacco cigarettes, and to prevent relapse back to smoking.”	
Outcomes	12‐week study. Follow‐up 24 weeks. Baseline, 1, 2, 4, 8, 12, and 24 weeks. Biochemically verified self‐reported continuous abstinence at 12 weeks from the previous visit. Continuous abstinence rate (CAR) weeks 4 to 12). Abstinence from smoking was defined as eCO‐verified (< 10 ppm). 7‐day point prevalence of abstinence at week 12. Secondary outcomes include: smoking reduction from baseline, adoption rates and product acceptability, tolerability, changes in step test values and in the level of selected biomarkers of exposure in exhaled breath (i.e. eCO) and in spot urine samples A follow‐up visit at 24 weeks to review product usage and smoking behaviour under naturalistic conditions of use	
Study funding	NCT record: Collaborators: Philip Morris Société Anonyme Caponnetto 2020: This research is supported by an Investigator‐Initiated Study award by Philip Morris Products Société Anonyme (PMI.IIS.2016.006).	
Author declarations	EM, DS and RP are full‐time employees of the University of Catania, Italy. PC, MC and RE are fixed‐term researchers at University of Catania, Italy. MM is a fixed‐term researcher at Centro per la Prevenzione e Cura del Tabagismo, University of Catania. BB is a full‐time employee of ARNAS Garibaldi, Catania, Italy. AP is a full‐time employee of Casa di Cura Musumeci‐Gecas, Gravina di Catania, Italy. UP is a full‐time employee of Ospedale “San Vincenzo” ‐ Taormina, Italy. In relation to his work in the area of tobacco control, RP has received lecture fees and research funding from Pfizer and GlaxoSmithKline, manufacturers of stop smoking medications. He has also received support from The Consumer Advocates for Smoke‐free Alternatives (CASAA) for publication and open access costs of one paper. He has also served as a consultant for Pfizer, Global Health Alliance for treatment of tobacco dependence, ECITA (Electronic Cigarette Industry Trade Association, in the UK), Arbi Group Srl., and Health Diplomats (consulting company that delivers solutions to global health problems with special emphasis on harm minimization). Lectures fees from a number of European electronic cigarette industry and trade associations (including FIVAPE in France and FIESEL in Italy) were directly donated to vapers advocacy no‐profit organizations. He is also currently involved in the following pro bono activities: scientific advisor for LIAF, Lega Italiana Anti Fumo (Italian acronym for Italian Anti Smoking League) and for The Consumer Advocates for Smoke‐free Alternatives (CASAA); Chair of the European Technical Committee for standardization on “Requirements and test methods for emissions of electronic cigarettes” (CEN/TC 437; WG4). The other authors have no conflict of interests to declare.	
Notes		
***Risk of bias***		
**Bias**	**Authors' judgement**	**Support for judgement**
Random sequence generation (selection bias)	Low risk	NCT record: The randomization sequence will be computer‐generated by using blocks size of 5, with an allocation ratio of 1:1 for each of the study products (IQOS, JustFog‐EC). This was not reported in the paper.
Allocation concealment (selection bias)	Low risk	From Caponnetto 2020: “assignment to one of the two groups must be made on the basis of a simple list. The “dummy” randomization list will be generated by the statistician through an SAS program (Statistical Analysis System Version 9.4). The randomization list will consist of 220 numbers of 3 digits in the form nnn (where “nnn” is a sequential number from 001 to 220). In derogation from the protocol (Study Design and Study Plan section) and in order to make it impossible to identify the subject's own arm to be randomized (before the number is assigned), the size of the blocks will not be 4 but will be variable in blocks of 4 and 6. The sequence of blocks will also be randomized and blind.”
Blinding of participants and personnel (performance bias) All outcomes	High risk	“Obviously, product assignment could not be blinded, and strong product preference (IQOS‐HTP vs JustFog‐EC) could have introduced an allocation bias.” Authors aware of a strong product preference
Blinding of outcome assessment (detection bias) All outcomes	Low risk	Self‐reported AEs. VO2 max measured by the Chester step test. eCO measured
Incomplete outcome data (attrition bias) All outcomes	Low risk	EC 101/110 12 weeks; 94/110 at 24 weeks
Selective reporting (reporting bias)	High risk	Study completed in May 2020 but 24‐week outcome data not reported in publication or in the trial register record in which 12‐week outcomes were reported

**Table:** Carpenter 2017

***Study characteristics***		
Methods	Design: randomized, parallel‐assignment, open‐label trial Recruitment: recruitment from local urban community in southeastern USA, using various media outlets Setting: community, southeastern USA Study start date: November 2014; Study end date: May 2016	
Participants	Total N: 68 N per arm: Control group: 22; ENDS group: 46 (split into 2 non‐randomized groups: BluCig 16 mg: 25; BluCig 24 mg: 21) Inclusion criteria: Age 18+ years Current smoker of ≥ 5 cpd for ≥ 1 year No recent history of cardiovascular distress, COPD, cancer (any non‐dermatologic), or uncontrolled diabetes mellitus Neither pregnant nor breastfeeding (verified) Absence of any major current psychiatric impairment, including current alcohol/drug abuse/dependence Current, active use of email At least some concern for health effects of smoking (> none at all on a Likert scale) Not used any ENDS product in the past 6 months Never purchased an ENDS product Exclusion criteria: Use of non‐cigarette tobacco products (e.g. cigarillos) in the last 30 days Current use of any smoking cessation medications Current enrolment in a smoking cessation treatment study Women: 59.7%; mean age: 42.2; mean cpd: 15.3; heaviness of smoking (0 to 6): 2.9 EC use: Control: 9%; ENDS 16 mg group: 4%; ENDS 24 mg group: 33% Motivation to quit smoking in next month (0 to 10): Control: 4.0; ENDS 16 mg: 5.0; ENDS 24 mg: 4.4	
Interventions	**EC: Cig‐a‐like** **Intervention**: At study start, choice of tobacco or menthol flavour Blu Starter Pack EC, with 16 mg/mL nicotine. Midway through study, the manufacturer of Blu altered the product and discontinued availability of the device, replaced with BluPlusþ, with 24 mg/mL nicotine. 3‐week sampling period, given up to 7 cartridges at each of 3 weekly visits. Instructions on usage "kept minimal to preserve naturalistic intent." The study team suggested that ENDS could be used "as you wish, to cut down or quit smoking, help manage smoking restrictions, or both." **Control**: own brand of cigarettes	
Outcomes	Weeks 2, 3, 4, 8, 12, and 16 Carbon monoxide, NNAL Other outcomes: cessation (< 6 months), product evaluation, EMA	
Study funding	"Support was provided by NIH R21 DA037407 (to M.J. Carpenter), P01 CA200512 (to K.M. Cummings, M.J. Carpenter, and M.L. Goniewicz), UL1 TR001450, and P30 CA138313. M.L. Goniewicz's laboratory is supported via P30 CA016056. B.W. Heckman is supported via K12 DA031794 and K23 DA041616. T.L. Wagener's effort is partially supported by the Oklahoma Tobacco Research Center, which is funded by the Oklahoma Tobacco Settlement Endowment Trust."	
Author declarations	"M.L. Goniewicz is a consultant/advisory board member for Johnson & Johnson. K.M. Cummings reports receiving a commercial research grant from and is a consultant/advisory board member for Pfizer Inc., and has provided expert witness testimony for various plaintiffs in lawsuits involving cigarette manufacturers. No potential conflicts of interest were disclosed by the other authors."	
Notes	New for 2020 update. Listed as ongoing study NCT02357173 in 2016 review update. Additional data provided from authors In all, 25 participants (54%) received the Blu Starter Pack (16 mg), and 21 participants (46%) received BluPlusþ (24 mg); no switches were made within participants. Note: this is not included in our analysis of higher v lower as assignment to nicotine dose was not done at random; 24 mg and 16 mg merged in our main analysis	
***Risk of bias***		
**Bias**	**Authors' judgement**	**Support for judgement**
Random sequence generation (selection bias)	Unclear risk	Quote: “Randomization to group was stratified by motivation to quit in the next 30 days (0–6 vs. 7–10 on a VAS scale) but proportioned 2:1 (ENDS:control) to increase precision estimates for e‐cigarette uptake and usage.”
Allocation concealment (selection bias)	Unclear risk	Not specified
Blinding of participants and personnel (performance bias) All outcomes	High risk	Not blinded and included non‐active control
Blinding of outcome assessment (detection bias) All outcomes	High risk	CO biochemically verified, but abstinence not used as outcome in this review, so rated based on adverse event reporting. Self‐report, no blinding of participants
Incomplete outcome data (attrition bias) All outcomes	Low risk	Retention rate: Week 4: control:19/22 (86%); ENDS 16 mg: 23/25 (92%); ENDS 24 mg: 20/21 (95%) Week 16: control: 16/22 (73%); ENDS 16 mg: 19/25 (76%); ENDS 24 mg: 15/21 (71%)
Selective reporting (reporting bias)	Unclear risk	Not specified
Other bias	Low risk	Midway through the study, the manufacturer of Blu altered the product and discontinued availability of the device, replaced with BluPlusþ, with 24 mg/mL nicotine, again offered in both tobacco and menthol flavourings, and with improved battery duration (4‐watt battery for both devices). In all, 25 participants (54%) received the Blu Starter Pack (16 mg), and 21 participants (46%) received BluPlusþ (24 mg); no switches were made within participants. The change in product (IRB approved) allowed us the unexpected opportunity to assess what impact, if any, the change in product design had on study outcomes. Note that the manufacturer, style of device, and packaging did not change, nor did our messaging to participants. The only difference was the strength of product. Thus, trial outcomes are reported across 3 groups: control versus 16 mg versus 24 mg ENDS. We have not rated this as high risk of bias as our analyses did not compare on nicotine strength and both nicotine arms were combined in our main analysis.

**Table:** Carpenter 2023

***Study characteristics***		
Methods	Design: RCT Setting: USA Recruitment: participants will be recruited nationally, but a subset (N = 120) will be recruited locally to allow for biomarker collection. Study start date: 24 May 2018. Study end date: 8 September 2022	
Participants	Total N: 638 427 = NJoy Pre‐Filled Tank 211 = control 53.6% female; mean age 42.3 (SD = 11.5), 68.5% white (18.5% black; 14.1% Hispanic), 30.9% ≤ HS education, and moderately dependent (mean Heaviness of Smoking Index = 14.8; SD = 7.2) Motivated to quit: limited interest in quitting smoking (mean motivation to quit on 0 to 10 VAS = 4.3; SD = 3.2; 24.1% making quit attempt in prior year). There is no requirement to quit smoking in this study, nor is there any requirement to use e‐cigarettes. Inclusion criteria: Age 21+; current smoker; regular use of email OR capacity to receive SMS text and internet access; additional smoking and health criteria determined at screening; minimal history of vaping	
Interventions	E‐cigarette: NJoy Pre‐Filled Tank, 3 mL pre‐filled tank, 15 mg/mL of nicotine. E‐cigarette samples are inclusive of a battery and self‐contained tanks of assorted flavours to last up to 4 weeks. Minimal instructions on use. Continue smoking their usual cigarettes as much or as little as they would like. Control arm: no intervention. Participants will not receive an NJOY electronic cigarette to sample and will continue smoking their usual cigarettes as much or as little as they would like.	
Outcomes	Baseline, 1, 2, 3, 4, 12, 24 weeks Uptake. Mean days of use over 4‐week sampling period (study enrolment through end of 4‐week intervention) Conversion. Percentage of participants who purchase an e‐cigarette (study enrolment through end of 6‐month follow‐up) Smoking cessation. Point prevalence abstinence from conventional smoking (6‐month follow‐up) AEs categorised as mild, moderate, or severe. The AEs most commonly reported were: cough, headaches, increased phlegm. Information collected between study enrolment and 6‐month FU. SAEs information collected between study enrolment and 6‐month FU. Study product use at 4, 12, and 24 weeks Participants will be asked to provide smoking diary data, captured electronically, daily for 4 weeks. More substantive phone assessment will track smoking and related behaviours at baseline (day 0) and +10, +17, and +24 days (weekly during initial 3 weeks, following brief lag for delays in product mailing), and at +1, +3, and +6 months.	
Study funding	Funding: NCI R01 210625	
Author declarations	Neither the tobacco industry nor any e‐cigarette manufacturer provides support of any kind for this study.	
Notes		
***Risk of bias***		
**Bias**	**Authors' judgement**	**Support for judgement**
Random sequence generation (selection bias)	Low risk	"Randomization occurred via stratified, mixed block design, stratifying on local vs. national enrollment and on desire to quit smoking: 0–6 vs. 7–10 on a VAS scale."
Allocation concealment (selection bias)	Low risk	Allocation was uploaded into REDCap meaning the research team were blinded to the sequence.
Blinding of participants and personnel (performance bias) All outcomes	High risk	Open‐label with comparator group with minimal control. Randomization did not include any masking.
Blinding of outcome assessment (detection bias) All outcomes	High risk	Self‐reported.
Incomplete outcome data (attrition bias) All outcomes	Low risk	EC: 292/427 (68%) Control: 163/211 (77%)
Selective reporting (reporting bias)	Low risk	All relevant prespecified outcomes provided by authors

**Table:** Cobb 2021

***Study characteristics***		
Methods	Design: randomized, parallel‐assignment, double‐blind trial Setting: USA. Penn State Medical Center in Hershey, Pennsylvania (n = 300); Virginia Commonwealth University in Richmond, Virginia (n = 220) Recruitment: community advertisements Study start date: June 2015; Study end date: June 2018	
Participants	Total N: 520 (though Veldheer paper only reports 263) N per arm: 130 per arm Inclusion criteria: age 21 to 65; smoke > 9 cigarettes per day; smoke regular filtered cigarettes or machine‐rolled cigarettes with a filter; CO measurement > 9 ppm at baseline; not planning to quit in the next 6 months; interested in reducing cigarette consumption; no serious quit attempt in last month, or use of FDA‐approved smoking cessation medication (varenicline, bupropion (used specifically as a quitting aid), patch, gum, lozenge, inhaler, and nasal spray) Exclusion criteria: unstable or significant medical condition in the past 12 months (recent heart attack or some other heart conditions, stroke, severe angina including high blood pressure if systolic > 159 or diastolic > 99 observed during screening); immune system disorders, respiratory diseases (exacerbations of asthma or COPD, require oxygen, require oral prednisone), kidney (dialysis) or liver diseases (cirrhosis), or any medical disorder/medication; use of any non‐cigarette nicotine delivery product (pipe, cigar, dip, chew, snus, hookah, e‐cigs, strips, sticks) in the past 7 days; uncontrolled mental illness or substance abuse or inpatient treatment for these in the past 6 months; difficulty providing blood samples; no surgery requiring general anaesthesia in the past 6 weeks; use of EC for ≥5 in the past 28 days or any use in the past 7 days; use of marijuana or any illicit drug/prescription drugs for non‐medical use daily/almost daily, or weekly in the past 3 months per NIDA Quick Screen; hand‐rolled, roll‐your‐own cigarettes; allergy to propylene glycol/vegetable glycerin; pregnancy/breastfeeding 58.8% women; mean age 47; mean cpd 18; mean FTND: not specified Motivated to quit: interested in reducing cigarette intake but not planning to quit in next 6 months EC use at baseline: none	
Interventions	**EC: Cartridge** For 24 weeks: 1) **Cigarette substitute**: QuitSmart cigarette substitute ‐ plastic tube looks like a real cigarette, designed to provide the same draw resistance as a smoker's usual cigarette. No drug delivery. 2 cigarette substitutes and a product manual are provided to participants following randomization and replacement products are provided throughout the intervention period (24 weeks). At baseline, associated user manual, research staff explain how to use product. Reduction goal to 50% at weeks 0 and 1, 75% at weeks 2 and 4, continue reducing onwards from there 2) **EC with no nicotine**: EGO e‐cigarette. Cartomizers containing 0 mg/mL nicotine provided throughout the intervention period (24 weeks). Associated user manual, research staff explain how to use product. 3) As (2) but 8 mg/mL nicotine 4) As (2) but 36 mg/mL nicotine	
Outcomes	0, 1, 2, 4, 8, 12, 16, 20, 24, 28, and 36 weeks Provision of the condition‐specific product lasted for 24 weeks (intervention period). There was a 12‐week follow‐up period after the intervention period for each condition (36 weeks). NNAL collected at baseline 4, 12, and 24 weeks Cessation: (a) intent‐to‐treat, self‐reported 7‐day point prevalence cigarette abstinence (PPA), biochemically confirmed by exhaled CO < 10ppm (7‐day PPA) for each visit up to 24 weeks after randomization (last visit of randomized phase of the trial), with those not attending visits counted as smoking. Additional outcomes included (b) self‐reported 28 or more days of cigarette abstinence at week 24 (biochemically validated by exhaled CO < 10 ppm at weeks 20 and 24), (c) the number (%) of participants in each group who reported at least one full day without smoking a cigarette (no biochemical verification), from week 1 to week 24, and (d) the total number of days on which participants self‐reported being abstinent from cigarettes from week 1 to week 24. “tobacco‐related toxicant exposure to the potent lung carcinogen NNK, as indexed by the sum of its urinary metabolite 4‐(methylnitrosamino)‐ 1‐(3‐pyridyl)‐1‐butanol (NNAL) and its glucuronides (total NNAL; pg/mg creatinine) collected at randomisation and at 4, 12, and 24 weeks.” Urine cotinine (ng/mg creatinine) at 4, 12, and 24 weeks Glutathione and 8‐Isoprostanes Exhaled CO was measured at each in‐person visit Pulmonary function tests were done at randomization, 4, 12, 24, and 36 weeks Self‐reported number of cigarettes smoked per day and daily study product use were assessed at each in‐person visit using a 7‐day timeline follow‐back procedure supplemented with paper diaries completed daily Adverse events and serious AEs Blood pressure, heart rate Other outcomes measured Drug/alcohol measures: Alcohol AUDIT‐C; NIDA Quick Screen Cigarette measures: MNWS; confidence to quit, Stage of Change, Environmental smoke, Smoking urges, 7‐day TLFB and current tobacco use Cigarette dependence Study product dependence and measures Psych measures: Kessler 6; perceived stress; CES‐D Health measures: Interheart, Clinical COPD Questionnaire Biomeasures: waist/hip ratio, weight Blood samples: complete metabolic panel, haematology panel, lipid panel, c‐reactive protein	
Study funding	This research was supported by grants P50DA036105 and U54DA036105 from the National Institute on Drug Abuse of the National Institutes of Health and the Center for Tobacco Products of the US Food and Drug Administration. Data collection was supported by UL1TR002649 at Virginia Commonwealth University and by UL1TR002014 at Penn State University from the National Center for Advancing Translational Sciences of the National Institutes of Health. The content of this manuscript is solely the responsibility of the authors and does not necessarily represent the official views of the National Institutes of Health or the US Food and Drug Administration.	
Author declarations	COC reports grants from National Institute on Drug Abuse and US Food and Drug Administration, during the conduct of the study. JF reports grants from National Institute on Drug Abuse and US Food and Drug Administration, during the conduct of the study, and grants, personal fees, and non‐financial support from Pfizer, outside of the submitted work. AAL reports grants from National Institute on Drug Abuse and US Food and Drug Administration, during the conduct of the study. JMY reports grants from National Institute on Drug Abuse and US Food and Drug Administration, during the conduct of the study. LK reports grants from National Institute on Drug Abuse and US Food and Drug Administration, during the conduct of the study. SV reports grants from National Institute on Drug Abuse and US Food and Drug Administration, during the conduct of the study. CB has previously undertaken trials of electronic cigarettes for smoking cessation (with electronic cigarettes purchased from an online retailer [NZVAPOR], electronic cigarette liquid for one trial purchased from Nicopharm, Australia, and nicotine patches supplied by the New Zealand Government via their contract with Novartis [Sydney, Australia]). Neither NZVAPOR nor Nicopharm have links with the tobacco industry. None of these parties had any role in the design, conduct, analysis, or interpretation of the trial findings, or writing of this publication. TE reports grants from National Institute on Drug Abuse and US Food and Drug Administration, during the conduct of the study, and is a paid consultant in litigation against the tobacco industry and also the electronic cigarette industry and is named on one patent for a device that measures the puffing behaviour of electronic cigarette users and on another patent for a smartphone application that determines electronic cigarette device and liquid characteristics. M‐SY reports grants from the National Institute on Drug Abuse and the US Food and Drug Administration, during the conduct of the study.	
Notes	Study listed as ongoing study Lopez 2016 in the 2016 review update and as Veldheer 2019 in 2020 and April 2021 updates. Cessation data from Foulds, which is pre‐print only	
***Risk of bias***		
**Bias**	**Authors' judgement**	**Support for judgement**
Random sequence generation (selection bias)	Low risk	"The study statistician (M‐SY) prepared site‐specific randomisation lists using the sample function in R version 3.2.0 (blocks of eight). These lists were uploaded onto a study‐specific website that interfaced with the data collection and management system (REDCap)."
Allocation concealment (selection bias)	Low risk	“Once a participant has been confirmed eligible for randomization, a computer procedure will assign the participant to the next condition on the list automatically.” "Only unmasked researchers at each site with no participant contact accessed their list to prepare cartomisers for dispensing."
Blinding of participants and personnel (performance bias) All outcomes	Low risk	Not blinded for non‐EC arms but, given similar level of support/product, performance bias judged unlikely
Blinding of outcome assessment (detection bias) All outcomes	Low risk	Not blinded for non‐EC arms but, given similar level of support/product, differential misreport judged unlikely
Incomplete outcome data (attrition bias) All outcomes	Low risk	"188 (36%) of 520 participants were lost to follow‐up by week 24; attrition did not differ by group (39 [30%] of 130 in the cigarette substitute group, 56 [43%] of 130 in the ENDS with 0 mg/mL nicotine group, 49 [38%] of 130 in the ENDS with 8 mg/mL nicotine group, and 44 [34%] of 130 in the ENDS with 36 mg/mL nicotine group; P = 0·15)."
Selective reporting (reporting bias)	Low risk	All specified outcomes available or being written up

**Table:** Coffey 2020

***Study characteristics***		
Methods	Design: 1‐armed intervention study All interested offered EC intervention. Pilot to encourage people wanting to stop using CCs to swap from CCs to ECs, conducted in 2018. The pilot was designed to enable current smokers to obtain a free e‐cigarette, charger, nicotine liquid, and support. Recruitment: E‐cigarette vouchers were provided through websites and also advertised and distributed by social housing providers and other community organizations, including pharmacies and stop smoking services. Setting: socially deprived area of the NW. Greater Manchester, NW England, UK Inclusion criteria: person using combustible cigarettes and wanting to quit Study start date: January 2018 for 3 months Study end date: March/April 2018	
Participants	Total N: 1022 (single arm) Mean age 44.7 years Mean CPD: 19.14 Motivated to quit: yes	
Interventions	All interested offered free EC intervention: 3 different ECs, different colours, 1 with longer battery life. Charger plug Two different strengths (1.0% and 1.6%) and 4 different flavours (tobacco, rolling tobacco, menthol, and mixed fruits) provided. 4 weeks' supply, distributed in 2 batches, 5 bottles at baseline and 5 bottles at week 2 E‐cigarettes were purchased from the only Independent British Vape Trading Association (IBVTA) registered e‐cigarette provider in the area (see https://www.ibvta.org.uk/about-us). Individuals obtained their e‐cigarette from a community stop‐smoking service or a pharmacy, which ensured that they obtained advice and guidance around stopping smoking and using the e‐cigarette, in addition to the device itself. People were required to return at 2 weeks in order to get their additional liquid, which helped to ensure that contact with the service could be maintained. A £10 high street shopping voucher was also offered as an alternative incentive to reduce bias in the population that returned for follow‐up.	
Outcomes	Baseline, 4 weeks CO ppm for all participants at 2 and 4 weeks follow‐up Continued EC use at 4‐week follow‐up	
Study funding	This work was commissioned by Salford City Council and carried out independently by the University of Salford. While Salford City Council and Great Manchester Health and Social Care Partnership were given the opportunity to input into the final report and the drafting of this paper, their input was limited to the context and background of the pilot, rather than the analysis or interpretation of the findings. This work was supported by funding from the Greater Manchester Health and Social Care Partnership.	
Author declarations	The author(s) disclosed receipt of the following financial support for the research, authorship, and/or publication of this article: This work was supported by funding by Greater Manchester Health and Social Care Partnership.	
Notes		
***Risk of bias***		
**Bias**	**Authors' judgement**	**Support for judgement**
Random sequence generation (selection bias)	High risk	Not randomized
Allocation concealment (selection bias)	High risk	No allocation concealment, single arm Uncontrolled study
Incomplete outcome data (attrition bias) All outcomes	Low risk	614/1022 (60%) overall LOW RISK Pharmacy: 167/362 (46.2% HIGH RISK) Community: 447/660 (67.85 LOW RISK) CO reading at 4 weeks 567/1022 = 55.5%
Selective reporting (reporting bias)	Unclear risk	No protocol/clinical record. Analysis of secondary data

**Table:** Czoli 2019

***Study characteristics***		
Methods	Design: non‐blinded, within‐participant cross‐over Recruitment: advertisements placed in newspapers, online, and in local vape shops, and received CAD 295 for participating in the study Setting: Kitchener−Waterloo and Toronto, Ontario, Canada Study start date: September 2015. Study end date: NR	
Participants	Total N: 48 29.2% female; mean age 35.9 (SD 11.7); mean cpd NR; dual EC users at baseline; not motivated to quit Inclusion criteria: > 18+ years; dual users of tobacco cigarettes and EC Exclusion criteria: serious intentions to quit smoking in the next 6 months; tobacco products, NRT, any smoking cessation medications, participation counselling programmes for smoking cessation in the past 7 days; serious cardiac health issues; heart attack or stroke within the last 3 months; cancer within the last year; asthma, chronic obstructive pulmonary disease, a seizure disorder, or any life‐threatening medical conditions with a prognosis of ≤ a year; history of psychosis, schizophrenia, bipolar disorder, or suicidal thoughts	
Interventions	**EC: own choice (mainly tank)** 3 consecutive 7‐day periods in which the use of tobacco cigarettes and e‐cigarettes was experimentally manipulated 4 study conditions: dual use (e‐cigarette and tobacco cigarette); tobacco cigarette; E‐cigarette; no product use Virtually all dual users reported using tank systems (92%) and e‐cigarettes with nicotine (94%). To control for order effects, participants were randomly assigned to 1 of 2 condition orders, A or B. Following the baseline condition of dual use: Group A participants switched to e‐cigarette use, then to tobacco cigarette use, and finally to no product use. Group B participants switched to tobacco cigarette use, then to e‐cigarette use, and finally to no product use.	
Outcomes	Baseline (visit 1) and after each of the 7‐day periods (visit 2 (week 1), visit 3 (week 2), visit 4 (week 3)) Carbon monoxide Urinary concentration of cotinine Urinary concentrations of 1‐hydroxypyrene (1‐HOP) and 4‐(methylnitrosamino)‐1‐(3‐pyridyl)‐1‐butanol (NNAL)	
Study funding	This research was supported by an Ontario Ministry of Health and LongTerm Care Health System Research Fund grant (#06697 awarded to DH). Additional support was provided by the Canadian Institutes of Health Research (CIHR), the Vanier Canada Graduate Scholarship (CDC), a CIHR and Public Health Agency of Canada, Applied Public Health Chair (DH), and an Ontario Institute for Cancer Research Investigator Award (GTF).	
Author declarations	MLG reports grants from and served as an advisory board member to pharmaceutical companies that manufacture smoking cessation drugs. DH has provided paid expert testimony in tobacco litigation on behalf of governments and class‐action plaintiffs on issues related to tobacco product science and regulation. The other authors have no competing interests to declare.	
Notes		
***Risk of bias***		
**Bias**	**Authors' judgement**	**Support for judgement**
Random sequence generation (selection bias)	Unclear risk	No details of randomization method given
Allocation concealment (selection bias)	High risk	No blinding
Blinding of participants and personnel (performance bias) All outcomes	High risk	No blinding
Blinding of outcome assessment (detection bias) All outcomes	Unclear risk	Not reported
Incomplete outcome data (attrition bias) All outcomes	Low risk	All followed up
Selective reporting (reporting bias)	Low risk	No evidence of selective reporting

**Table:** Dawkins 2020

***Study characteristics***		
Methods	Design: prospective cohort 4‐centre pragmatic cluster feasibility trial Recruitment: at homeless centres Setting: 4 homeless centres in the UK Study start date: 1 October 2018; Study end date: 31 March 2020	
Participants	Total N: 80 N per arm: EC 48; UC 32 Inclusion criteria: adults (≥ 18 years) who smoke accessing homeless support services on a regular basis and also known to staff; daily smokers; smoking status was also biochemically verified by exhaled CO breath Exclusion criteria: non‐smokers, or using another smoking cessation aid; pregnancy, or unable to consent, e.g. currently intoxicated or unable to speak English; not well known to centre staff Inclusion based on specific population characteristics: people accessing homeless centres 35% women; mean age 42.7; mean cpd 20; mean FTND: FTCD 5.51 Motivated to quit: “varied considerably; large majority expressed a desire to quit smoking in the near future” EC use at baseline: not specified	
Interventions	**EC: Refillable** **Usual care**: written information on quitting smoking (adapted from NHS Choices); signposting to the local stop‐smoking service (SSS) by centre staff **Intervention**: as usual care, plus refillable EC provided once with e‐liquid provided 1 x wk for 4 weeks, Aspire PockeX (tank style), choice of 3 flavours (fruit, menthol, tobacco) and 2 nicotine strengths (12 mg/mL or 18 mg/mL). Written info for EC use and support from centre staff, who met once a week to provide e‐liquid and troubleshoot EC use	
Outcomes	Weeks: 4, 12, 24; clinic visits and self‐report Cessation: CO‐validated sustained at 24 weeks Adverse events and biomarkers: self‐reported negative effects in EC arm only – each participant asked to rate on a scale so we could not meta‐analyse; exhaled CO; unintended consequences Other outcomes measured: Qualitative process evaluation; costs; self‐reported positive and negative affects; recruitment rates; retention; EC/other tobacco/nicotine product use at study end; HRQoL; healthcare service utilization; other drug use/dependence; unintended consequences	
Study funding	This study is funded by the National Institute for Health Research Public Health (project reference: 17/44/29).	
Author declarations	SC, AF, JL, CB, AT, DR, IU, LB, SP have no competing interests. PH has received a research grant from and provided consultancy to Pfizer. LD has provided consultancy for the pharmaceutical industry relating to the development of smoking cessation products.	
Notes	New for 2021 update. Authors provided information prior to peer review.	
***Risk of bias***		
**Bias**	**Authors' judgement**	**Support for judgement**
Random sequence generation (selection bias)	High risk	Intention was to randomize but were unable to due to practical constraints Quote: “Thus the actual allocation of centres to each arm was a pragmatic decision based on centre readiness and staff/researcher availability though we balanced potential confounders and differences in environment by ensuring each cluster (EC and UC) contained one day centre and one residential unit.”
Allocation concealment (selection bias)	Unclear risk	Quote: “Participants joined after cluster randomisation… Allocation was concealed to participants until after the baseline assessment.” Comment: But unclear if allocation was concealed for those recruiting, and allocation would have been known to new participants
Blinding of participants and personnel (performance bias) All outcomes	High risk	Not blinded and different levels of support between arms, so performance bias cannot be ruled out
Blinding of outcome assessment (detection bias) All outcomes	Low risk	Cessation (primary outcome) biochemically validated
Incomplete outcome data (attrition bias) All outcomes	High risk	13/48 (27.1%) lost to follow‐up in the intervention arm and 20/32 (62.5%) lost to follow‐up in the control arm at 24 weeks
Selective reporting (reporting bias)	Low risk	All anticipated outcomes reported

**Table:** Edmiston 2022*

***Study characteristics***		
Methods	Design: RCT Recruitment: participants were recruited to 10 clinical sites via site databases and using IRB‐approved radio and print ads. Setting: USA Study start date: 17 January 2017. Study end date: 6 November 2018	
Participants	Total N: 450 EC Test group 1: classic (tobacco) = 150 EC Test group 2: menthol = 150 Control = 150 Inclusion criteria: smoked ≥ 10 years, smoked an average of ≥ 10 manufactured cigarettes pd for 12 months. Willing and able to replace their cigarettes for 12 weeks with the assigned test e‐Vapor product. Age 30 to 65 years. Exclusion criteria: health condition that would jeopardize the safety of the participant or impact the validity of the study results; currently taking medication for depression, asthma or diabetes For a full list of inclusion and exclusion criteria, see publication. Female 51%; mean age 44.4 (SD 9.73); mean CPD 17.6 (SD 4.95)	
Interventions	EC: **cartridge** Arm 1 Experimental: Test 1 EC classic (tobacco) Exclusive ad libitum use of test product MarkTen Bold Classic (test product 1) 4.0% nicotine by weight without use of any other type of tobacco/nicotine‐containing product, for the entire duration of study participation. This replaced test e‐Vapor Product NuMark LLC, MarkTen® XL Bold CLASSIC* (as no longer sold). Arm 2 Experimental: Test 2 EC menthol Exclusive ad libitum use of test products MarkTen Bold Menthol (test product 2) 4.0% nicotine by weight Arm 1 and 2 participants were to completely replace their cigarettes with the test EVPs. Participants had 7 days to switch to EVPs prior to clinic visits. Arm 3 No Intervention: control Assigned to continue smoking their own brand cigarettes under ad libitum conditions	
Outcomes	Baseline, weeks 1, 3, 6, 9, and 12 Baseline weeks 1, 6 and 12 (blood and urine). Weeks 1, 3, 6, 9, and 12 (eCO) Lung function was assessed at screening, baseline, at week 12 of study 1, (and at weeks 18 and 24 of study 2): FEV1, per cent of predicted FEV1, FVC, percent of predicted FVC, FEV1/FVC, and percent of predicted FEV1/FVC, forced expiratory flow at 25% to 75% (FEF). NNAL urinary total 4‐(methylnitrosamino)‐1‐(3‐pyridyl)‐1‐butanol and its glucuronides (ng/g creatinine), WBC, COHb, HDLC Adverse events (AEs) and medications were recorded and monitored throughout the study.	
Study funding	This study was funded by Altria Client Services LLC. (Altria is the parent company of Philip Morris USA (producer of Marlboro cigarettes), John Middleton, Inc., U.S. Smokeless Tobacco Company, Inc., and Philip Morris Capital Corporation.)	
Author declarations	All authors were employees of Altria Client Services LLC at the time of the study.	
Notes	Paper reported 2 studies; only 1 study was eligible. New to 2022 update	
***Risk of bias***		
**Bias**	**Authors' judgement**	**Support for judgement**
Random sequence generation (selection bias)	Unclear risk	Randomised – no further information in paper or supplementary materials
Allocation concealment (selection bias)	Unclear risk	No detail provided
Blinding of participants and personnel (performance bias) All outcomes	High risk	Open‐label
Blinding of outcome assessment (detection bias) All outcomes	Unclear risk	No detail provided
Incomplete outcome data (attrition bias) All outcomes	Low risk	< 50% attrition
Selective reporting (reporting bias)	Low risk	Outcomes reported

**Table:** Edwards 2023

***Study characteristics***		
Methods	Design: pragmatic, uncontrolled, mixed‐methods trial Recruitment: targeted settings for people with HIV Setting: community, Brisbane, Australia Study start date: 21 February 2017; study end date: 26 October 2017	
Participants	Total N: 30 Inclusion criteria: diagnosis of HIV; ≥ 18 years; ≥ 5 cpd at the time of enrolment into the trial; smoking ≥ 12 months; willing to attempt to quit tobacco smoking after study enrolment Exclusion criteria: participating in a smoking‐cessation programme; pregnancy or breastfeeding; experiencing chest pain, or another cardiovascular event or procedure in the last month; being treated with oxygen therapy Inclusion based on specific population characteristics: people living with HIV 29 participants identified as male, and 1 participant did not identify as male or female; mean age: 42; mean cpd: 18 EC use at baseline: 46.7% (n = 14) never tried; 50% (n = 15) tried, never used for an extended period; 3.3% (n = 1) used on a regular (weekly) basis Willing to attempt to quit	
Interventions	**EC: Refillable** Single‐arm study. Print materials to help quit smoking. Provided booklet with instructions on how to use, store and handle EC; copies of device user manuals. Given Innokin Endura T18® vaporiser kit, Innokin Endura T22® vaporiser kit, 4 spare coils, 1 wall charger, 10 x 10 mL bottles of Nicophar® 12 mg nicotine e‐liquid. Supplies to last 12 weeks	
Outcomes	Weeks 1, 4, 8, 12, 24; self‐report and semi‐structured interviews Cessation: 7 days point prevalence at weeks 4, 8, 12, and 24. Continuous abstinence at weeks 12 and 24. No biochemical validation Adverse events Other outcomes: acceptability and use of trial products; number of quit attempts	
Study funding	"This work was supported by the HIV Foundation Queensland. The funder will play no role in the analysis and interpretation of results. All trial products were purchased and the suppliers have no involvement in the conduct of the trial or the interpretation or reporting of the results."	
Author declarations	"No other authors declare conflicts of interest. Mark Boyd has received research grant funding (paid to the institution) from AbbVie, Gilead and Merck and received honoraria for participation in HIV Advisory Boards and for the preparation and delivery of educational materials from AbbVie, Boehringer‐Ingelheim, Bristol Myers Squibb, Gilead, Janssen‐Cilag, Merck and ViiV Healthcare."	
Notes	Additional data provided from authors. New for 2020 update, relabelled from Bell 2017 to Edwards 2023 at 2023 update.	
***Risk of bias***		
**Bias**	**Authors' judgement**	**Support for judgement**
Random sequence generation (selection bias)	High risk	Uncontrolled study
Allocation concealment (selection bias)	High risk	Uncontrolled study
Incomplete outcome data (attrition bias) All outcomes	Low risk	Quote: “At week 24, 26 of the 30 participants who enrolled in the study were followed up.” (confirmed by authors)
Selective reporting (reporting bias)	Low risk	Study not published at time of data extraction, but study protocol published

**Table:** Eisenberg 2020

***Study characteristics***		
Methods	Design: 3‐arm RCT Recruitment: community Setting: Canada Study start date: November 2016. Study end date: September 2019	
Participants	Total N: 376; nicotine e‐cigarettes = 128; non‐nicotine e‐cigarettes = 127; counselling (control) = 121 47% female; mean age 52.66; mean cpd 21; mean FTND 6 (SD 2) Motivated to quit: yes Inclusion criteria: active smoker, ≥ 10 CPD for past year; ≥ 18 years; motivated to quit according to the Motivation To Stop Scale (MTSS) (level 5 or higher); provide informed consent in English or French; available for follow‐up (1 year) Exclusion criteria: medical condition with a prognosis < 1 year; current or recent cancer (≤ 1 yr in remission); pregnancy/breastfeeding; current/ recent use of any pharmacotherapy or behavioural therapy for smoking cessation (e.g. NRT, bupropion, varenicline, or counselling); any EC use (nicotine/non‐nicotine) in past 60 days, or ever use of any EC ≥ 7 days consecutively; psychosis, schizophrenia, or bipolar disorder; ≤ 1 month following myocardial infarction, life‐threatening arrhythmia, severe or worsening angina pectoris, or cerebral vascular accident; illegal drug use past yr (excluding marijuana); planned use of tobacco products other than conventional cigarettes (e.g. cigarillos, cigars, snuff, shisha, etc.) or marijuana during the study period	
Interventions	**EC: Cig‐a‐like** **Nicotine e‐cigarettes plus counselling:** 12 weeks of e‐cigarettes. Rechargeable base with prefilled, disposable, tobacco‐flavoured liquid cartridges (15 or 0 mg nicotine/mL), which were produced specifically for use in clinical studies (purchased from NJOY Inc, Scottsdale, Arizona). 21 cartridges at baseline with additional cartridges supplied as needed. Nicotine and non‐nicotine e‐cigarettes were identical in appearance. Instructed to be used as desired. No schedule for e‐cigarette tapering, but participants were aware that they would return their e‐cigarettes after 12 weeks. Participants received individual smoking cessation and relapse prevention counselling (minimum 30 minutes at baseline, 10 minutes during telephone follow‐ups, and 15 to 20 minutes at clinic visits). Individualized quit plans. **Non‐nicotine e‐cigarettes plus counselling:** As above with 0 mg nicotine/mL in liquid cartridge **Counselling (control):** Participants received individual smoking cessation and relapse prevention counselling (minimum 30 minutes at baseline, 10 minutes during telephone follow‐up, and 15 to 20 minutes at clinic visits). Individualized quit plans.	
Outcomes	Follow‐up was conducted by telephone at weeks 1, 2, 8, and 18, and at clinic visits at weeks 4, 12, 24, and 52. Self‐reported smoking (7‐day recall), adherence, and adverse events (AEs) were assessed during follow‐up contacts Biochemically‐validated 7‐day point prevalence smoking abstinence at 4, 12, and 24 weeks, defined as self‐reported abstinence in the past 7 days with exhaled carbon monoxide < 11 ppm At baseline: cpd; FTND; Glover‐Nilsson Smoking Behavioral Questionnaire (to assess behavioural dependence on smoking); and Beck Depression Inventory II (BDI‐II; to assess depressive symptoms)	
Study funding	This trial was funded by the Canadian Institutes of Health Research (CIHR; funding reference No. 133727 and 155969). Both nicotine e‐cigarettes and non‐nicotine e‐cigarettes were purchased from NJOY Inc (Scottsdale, Arizona)	
Author declarations	Dr Eisenberg reported receiving educational grants from Pfizer Inc for providing continuing medical education in cardiology. Dr Wilderman reported receiving financial compensation from Pfizer Inc for his involvement in a smoking cessation study using varenicline. Dr Filion reported receiving salary support from the Fonds de Recherche du Quebec, a William Dawson Scholar award from McGill University, and personal fees from Institut National D’excellence en Santé et Services Sociaux. No other disclosures were reported.	
Notes	New cessation and adverse event data for 2021 update. Previously listed as NCT02417467 (included with SAE data only)	
***Risk of bias***		
**Bias**	**Authors' judgement**	**Support for judgement**
Random sequence generation (selection bias)	Low risk	Eligible participants were randomized via an online central randomization system. The system used a computer‐generated randomization list containing permuted blocks of 6 and 9, stratified by centre.
Allocation concealment (selection bias)	Low risk	Participants, investigators, and study personnel were blinded to nicotine content in the e‐cigarette groups.
Blinding of participants and personnel (performance bias) All outcomes	Low risk	Participants, investigators, and study personnel were blinded to nicotine content in the e‐cigarette groups.
Blinding of outcome assessment (detection bias) All outcomes	Low risk	Participants, investigators, and study personnel were blinded to nicotine content in the e‐cigarette groups.
Incomplete outcome data (attrition bias) All outcomes	Low risk	Low numbers lost to follow‐up, treated as ITT
Selective reporting (reporting bias)	Low risk	Due to a prolonged and unforeseen delay in e‐cigarette manufacturing, enrolment was paused on 27 September 2019, and then terminated on 14 November 2019. Given reduced power, the timing of the primary endpoint was changed from 52 weeks to 12 weeks on 04 December 2019. No 12‐month follow‐up but this was for manufacturing reasons and was reported

**Table:** Eisenhofer 2015

***Study characteristics***		
Methods	Design: RCT Setting: USA	
Participants	11 EC = 4; NRT = 7 Veterans who meet DSM criteria for tobacco use disorder 18% female; mean age 52.6; mean cpd 26.4; mean FTND 7.5; 64% African American	
Interventions	EC type: cartridge Arm 1: Electronic cigarettes 16 mg cartridge. Arm 2: NRT Participants attended thrice‐weekly visits during the first 2 weeks (week 1‐“baseline” with participants smoking ad libitum) and attended 5 visits during the third week (week 3‐“efficacy” with participants smoking as little as possible while using NRT or E‐cigs)	
Outcomes	Self‐reports of cigarettes smoked in last 24 hours, confirmed by breath CO levels and salivary cotinine	
Study funding	This work was conducted at and supported by resources at the MEDVAMC, including a MEDVAMC Research Enhancement Seed Grant.	
Author declarations	NS	
Notes	Study information extracted from conference abstract only	
***Risk of bias***		
**Bias**	**Authors' judgement**	**Support for judgement**
Random sequence generation (selection bias)	Unclear risk	Quote: “Veterans were randomized to either NRT (16 mg patch; N = 7) or E‐cigs (16 mgcartridge; N = 4).” No further information provided
Allocation concealment (selection bias)	Unclear risk	No information provided
Blinding of participants and personnel (performance bias) All outcomes	Low risk	No blinding, but active interventions provided to both arms
Blinding of outcome assessment (detection bias) All outcomes	Low risk	Quote: "CO levels and salivary cotinine were recorded during each visit.' 'Self‐reports of cigarettes smoked in last 24 h, and this was confirmed by significant reductions of breath CO levels by NRT (t = 3.7, P = 0.01) and E‐cigs (t = 3.9, P = .03)."
Incomplete outcome data (attrition bias) All outcomes	Unclear risk	No information provided
Selective reporting (reporting bias)	Unclear risk	No protocol or clinical trial record available to determine whether all prespecified outcomes were reported

**Table:** Elling 2023

***Study characteristics***		
Methods	Design: RCT Setting: Netherlands, online Recruitment Study start date: recruited March 2020 to July 2020	
Participants	N = 331 Intervention = 157; control = 174 % female: 59.8%; mean age: 49.0 (SD 13.2); mean CPD: 12.6 (cigarettes per week 83.3/7); mean FTND: 3.8 (2.6) Intervention 4.1 (2.6), Control 3.5 (2.5) Inclusion criteria were that participants are at least 18 years old, have sufficient command of the Dutch language, have necessary internet literacy to use the intervention, have smoked tobacco in the past 7 days, and are motivated to quit tobacco smoking within 5 years. EC use at baseline: overall 33/331 (10%). Intervention 17 (10.8); control 16 (9.2) Motivated to quit: yes. Within next 5 years	
Interventions	Digital computer‐tailored smoking cessation intervention Arm 1: intervention condition Participants in the intervention condition will receive tailored information on e‐cigarettes based on 5 items. The computer‐tailored intervention will be based on the I‐Change model. Arm 2: control condition The control condition will not receive tailored information about e‐cigarettes.	
Outcomes	Baseline, 6 months Number of tobacco cigarettes smoked in the past 7 days; average number of tobacco cigarettes smoked per day; 7‐day point prevalence tobacco abstinence; 7‐day point prevalence e‐cigarette abstinence Smoking cessation methods; determinants of decision‐making; process evaluation	
Study funding	National Institute for Public Health and the Environment (Rijksinstituut voor Volksgezondheid en Milieu, RIVM)	
Author declarations	No conflicts of interest declared	
Notes		
***Risk of bias***		
**Bias**	**Authors' judgement**	**Support for judgement**
Random sequence generation (selection bias)	Unclear risk	Stated randomized, but the process was not described in the paper, protocol, or record
Allocation concealment (selection bias)	Unclear risk	Not reported
Blinding of participants and personnel (performance bias) All outcomes	Unclear risk	Online, so participants would be unlikely to be aware of other participants
Blinding of outcome assessment (detection bias) All outcomes	High risk	Self‐reported abstinence
Incomplete outcome data (attrition bias) All outcomes	Low risk	139/157 intervention 138/174 control
Selective reporting (reporting bias)	Low risk	Stated investigating the hypothesis of whether intervention arm smokeless at 6 months

**Table:** Ely 2013

***Study characteristics***		
Methods	Design: prospective cohort Recruitment: letter sent to family practice patients who currently smoked Setting: single family practice, Colorado, USA Study start date: 14 April 2013; Study end date: not specified	
Participants	Letters sent to 640 patients, 48 chose to participate and 44 completed the programme, 4 were lost to follow‐up Inclusion criteria: Want to quit or switch from tobacco cigarettes to ECs Exclusion criteria: None reported Of the 44 participants, 66% women, all non‐Hispanic/white, aged 20 to 75 (30% were age 51 to 60), 57% had a high school education or less Motivated to quit: want to quit or switch from tobacco cigarettes to ECs E‐cigarette use at baseline: not specified	
Interventions	**EC: Cig‐a‐like** The 6‐month smoking cessation programme was based on the '5 A's' model and transtheoretical model. Options for treatment were discussed with each participant at the start of the programme. All used an EC, with 16 using bupropion and 2 using varenicline as well. Participants were provided with written information on “blu cig” and “smoke tip” ECs, about cost, availability, nicotine dosage options.	
Outcomes	Phone follow‐ups at 2 weeks, 1 month, 3 months, and 6 months At completion of programme (using ITT) Abstinence from smoking and EC use Abstinence from smoking but not EC use ≥ 50% reduction of baseline cigarette consumption (still using ECs)	
Study funding	Not specified	
Author declarations	Not specified	
Notes		
***Risk of bias***		
**Bias**	**Authors' judgement**	**Support for judgement**
Random sequence generation (selection bias)	High risk	Prospective cohort
Allocation concealment (selection bias)	High risk	Not randomized
Incomplete outcome data (attrition bias) All outcomes	Low risk	4/48 lost to follow‐up
Selective reporting (reporting bias)	Unclear risk	Unable to determine prespecified outcomes
Other bias	Unclear risk	No definition of abstinence provided Not clear if 'completed programme' was at 6 months

**Table:** Felicione 2019

***Study characteristics***		
Methods	Design: double‐blind RCT Recruitment: people who smoke were recruited from an outpatient opioid‐maintenance clinic in West Virginia, USA Setting: outpatient opioid‐maintenance clinic in West Virginia, USA Study start date/Study end date: not reported	
Participants	Total N: 25; N per arm: placebo (non‐nicotine): 11; active (18 mg/mL nicotine): 14 Inclusion criteria: ≥ 18 years of age Report smoking ≥ 10 cpd for ≥ 1 year Report a current interest in quitting smoking Exclusion criteria: Reported regular use of any nicotine/tobacco product other than cigarettes, including EC, or were already engaged in attempts to quit smoking Inclusion based on specific population characteristics: people who smoke who were currently receiving a buprenorphine/naloxone combination in sublingual form, and had maintained sobriety from opioids and all other illicit substances for at least 90 consecutive days as verified via urinalysis 73.0% women; mean age 32.5; mean cpd 22; mean FTND 5.8 Motivated to quit: Quit ladder score (range 1 to 10): 5.6 average	
Interventions	**EC: Refillable** Compared **nicotine** (18 mg/mL) to **non‐nicotine EC** Second‐generation EC consisted of the eGo‐T battery (900mAh, 3.3 V constant output) (Joyetech; Irvine, CA) and the Kanger mini Protank‐II, 1.5 ml Pyrex glass tank with a drip tip and atomizer head coils (KangerTech; China), choice between tobacco (n = 15) and menthol (n = 10) flavoured liquid (2‐week supply). Participants were then trained in EC device operation, including assembly, liquid filling, manual battery operation, and cleaning/storage. Practised puffing on EC in the presence of a team member, and asked questions if needed. Participants instructed to use their ECIG ad libitum every day for 2 weeks.	
Outcomes	Baseline (day 1), 14 days, 28 days for clinic measures. Data also collected via text messages over 2‐week intervention period Withdrawal/side effects: every evening during the 2‐week intervention period, participants rated a variety of effects possibly experienced as a result of nicotine/tobacco withdrawal and/or use of the ECIG: nausea, dizziness, throat irritation/soreness, cough, dry mouth, headache, shortness of breath, irritability/frustration/anger, craving/urge to smoke, and other. Each item was rated on a continuous scale that ranged from 0 (not at all) to 100 (extremely). Expired air CO Other outcomes: self‐reported cigarette and EC use; readiness to quit at day 1, 14, and 28	
Study funding	Not reported	
Author declarations	Not reported	
Notes	New for 2020 update	
***Risk of bias***		
**Bias**	**Authors' judgement**	**Support for judgement**
Random sequence generation (selection bias)	Unclear risk	Quote: “Using a mixed factorial, simple randomization, double‐blind study design, participants were assigned to one of two ECIG conditions…” (No further details given)
Allocation concealment (selection bias)	Unclear risk	No details on allocation given
Blinding of participants and personnel (performance bias) All outcomes	Unclear risk	Quote: “double‐blind study design”, no further detail given
Blinding of outcome assessment (detection bias) All outcomes	Unclear risk	Quote: “double‐blind study design”, no further details given
Incomplete outcome data (attrition bias) All outcomes	Low risk	Quote: “…80.6% completed the two‐week intervention (n = 14 active; n = 11 placebo), and 70.9% also completed the follow‐up session (n = 13 active; n = 9 placebo).” Active follow‐up completion rate: 13/14 = 93%; placebo follow‐up completion rate: 9/11= 82% N.B. 6 participants were disqualified post‐randomization: Quote: “Of those individuals who were screened for the study, 93.9% were enrolled (n = 18 active; n = 13 placebo); two individuals who were ineligible provided an expired air CO level < 10 ppm. Six of the enrolled participants (n = 4 active and n = 2 placebo; n = 5 tobacco flavour and n = 1 menthol flavour) were disqualified for responding to 7 or fewer days of text messages.”
Selective reporting (reporting bias)	Unclear risk	All measures listed were reported: self‐reported cigarette use, text message‐based cigarette use, e‐cig use, expired air CO, readiness to quit ladder, withdrawal/side effects No study protocol or clinical trial record available to confirm all intended outcome measures were reported.

**Table:** George 2019

***Study characteristics***		
Methods	Design: prospective, randomized controlled trial with a parallel, non‐randomized preference cohort Recruitment: participants were recruited from local advertisements, smoking cessation databases, and visits to local businesses, as well as via the Scottish Primary Care Research Network Setting: single tertiary research centre, UK Study start date: August 2016; Study end date: July 2018	
Participants	Total N: 114 in “final evaluable dataset” (145 recruited into the trial) N per arm: tobacco cigarettes (TC): 40; EC nicotine (16 mg): 37; EC nicotine‐free: 37 Inclusion criteria: ≥ 18 years of age who had smoked ≥ 15 cigarettes/day for at least 2 years; free from established CV disease, diabetes, and chronic kidney disease (and not on medication for those conditions); willing to stop tobacco cigarettes for period of study if required and not to use EC if required. Exclusion criteria: pregnant/breastfeeding; not abstaining from sex or using effective contraception; medication for CVD; history of CVD (excluding hypertension), diabetes, active malignancy or chronic renal disease; nut allergy; participation in another clinical trial (other than observational trials and registries) with an investigational product and/or intervention within 30 days before visit 1. 65.4% women; mean age 46.9; mean cpd 18.7 Motivated to quit: TC group: no; EC nicotine (16 mg): yes; EC nicotine‐free: yes	
Interventions	**EC: Cig‐a‐like** **EC nicotine** (16 mg) arm: EC containing 16 mg nicotine (Vapourlites Starter Kit with XR5 16 mg nicotine cartomizer; Vapourlites, Peterlee, United Kingdom) **EC nicotine‐free** arm: nicotine‐free EC plus nicotine flavouring (Vapourlites Starter Kit with 0 mg nicotine cartomizer) **(non‐randomized) TC arm:** continued their usual daily smoking habits and did not use EC for the 4‐week period of the trial	
Outcomes	Week 4 Adverse events and biomarkers: BP, heart rate, adverse events Other outcomes measured: endothelial function, oxidised low‐density lipoprotein, high‐sensitivity C‐reactive protein, tissue plasminogen activator, and platelet activation inhibitor‐1	
Study funding	"The VESUVIUS (Vascular Effects of Regular Cigarettes Versus Electronic Cigarette Use) trial was funded by the British Heart Foundation (grant PG/15/64/31681); and supported by Immunoassay Biomarker Core Laboratory, University of Dundee, the Tayside Medical Sciences Centre, and the NHS Tayside Smoking Cessation Service. The funder had no role in the study design, data collection, data analysis, data interpretation, writing of the report, or in the decision to submit for publication."	
Author declarations	"Dr. Donnan has received research grants from AbbVie, Shire, and Gilead Sciences. All other authors have reported that they have no relationships relevant to the contents of this paper to disclose."	
Notes	New for 2020 update	
***Risk of bias***		
**Bias**	**Authors' judgement**	**Support for judgement**
Random sequence generation (selection bias)	Low risk	Consented participants who were willing to quit smoking were randomized to one of the EC arms in a 1:1 fashion using a centrally controlled web‐based good clinical practices– compliant randomization system to either: 1) EC containing 16 mg nicotine; or 2) nicotine‐free EC plus nicotine flavouring because it was considered by the institutional ethics committee as ethically unacceptable to randomize those who were willing to quit smoking into a smoking arm. Those unwilling to consider quitting smoking continued in the parallel preference TC cohort.
Allocation concealment (selection bias)	Low risk	Central randomization
Blinding of participants and personnel (performance bias) All outcomes	Unclear risk	Not specified
Blinding of outcome assessment (detection bias) All outcomes	High risk	Not blinded and AE/SAE data are self‐report only. For other outcomes, low risk as objectively measured: Quote: “Patients fasted overnight and measurements were conducted at baseline and 1 month according to the International Brachial Artery Reactivity Task Force guidelines (19) by a single operator (M.H.) blinded to study allocation at a single site.” “Pulse wave velocity and augmentation index were measured at baseline and 1 month by a single operator (M.H.) blinded to study allocation.”
Incomplete outcome data (attrition bias) All outcomes	Unclear risk	Number randomized not provided per group Quote: “A total of 145 patients were recruited into the trial. A final number of 114 patients (40 TC, 37 EC‐nicotine, 37 EC‐nicotine‐free) completed both visits.”
Selective reporting (reporting bias)	Low risk	Clinical trial record lists: change in FMD; change in oxidized LDL; change in PAI‐1; change in hs‐CRP; change in pulse wave velocity; change in tissue plasminogen activator; change in augmentation index @ 75 bpm All reported in the paper

**Table:** Goniewicz 2017

***Study characteristics***		
Methods	Design: longitudinal within‐participant, observational Recruitment: advertisements in the media, the internet, posted advertisements in clinics and offices, and by word of mouth Setting: University, Poland Study start date: March 2011; Study end date: June 2011	
Participants	Total N: 22 started out and 2 dropped out in the first week due to an adverse event (nausea) and inability to commit to clinic visits. This resulted in an analytic sample of 20. Inclusion criteria: 18 years or older, current daily cigarette smokers (> 5 cpd within the last 12 months) May have had interest in quitting smoking, in good health (at the clinic screening visit) Able to communicate in Polish Able to use an e‐cigarette safely Exclusion criteria: Diagnosed as having asthma, COPD, hypertension, inhaled allergies, chronic heart disease, or cancer Taking a cardiac medication Pregnant 60% women; mean age 31; mean cpd 16; mean FTND 3.9 Motivated to quit: at the time of screening, 95% of participants (n = 19) reported planning to quit smoking, with 80% (n = 16) reporting that they have made at least 1 quit attempt prior to involvement in the study E cigarette use at baseline: not reported	
Interventions	**EC: Cig‐a‐like** Pen‐style M201 e‐cigarettes for 2 weeks, with an automatically‐operated battery with an output power of 4.6 Volts (280 mAh) and the heating element resistance of 3.6 to 3.8 Ohms. At baseline, provided with EC (M201 Mild, Poland) with 20 tobacco‐flavoured cartridges a week containing 11.0 ± 1.5 mg of nicotine in a mixture of propylene glycol and vegetable glycerin (50:50). Encouraged to substitute their regular cigarettes with the e‐cigarettes for 2 weeks and refrain from smoking	
Outcomes	Day 7, Day 14 Adverse events and biomarkers: Biomarkers were metabolites of 13 major carcinogens and toxicants in cigarette smoke: 1 tobacco‐specific nitrosamine (NNK), eight volatile organic compounds (1.3‐butadiene, crotonaldehyde, acrolein, benzene, acrylamide, acrylonitrile, ethylene oxide, and propylene oxide), and 4 polycyclic aromatic hydrocarbons (naphthalene, fluorene, phenanthrene, and pyrene) Questionnaire on ‘health’: at each visit, participants were asked, “In the last week, have you experienced any of the following symptoms?”, while providing a response of “never,” “rarely,” or “often” to the following list of health effects: daytime cough, difficulty concentrating, difficulty breathing during sleep, difficulty sleeping, dizziness, headache, irritability, nausea, nighttime cough, chest pain, phlegm, shortness of breath, tightness in chest, visual disturbances, and wheezing. Responses of “rarely” or “often” were combined to indicate presence of an adverse health effect. Expired CO Other outcomes measured: 7 nicotine metabolites (3‐hydroxycotinine, cotinine, cotinine N‐oxide, nicotine N‐oxide, norcotinine, nornicotine, nicotine) Revised Minnesota Nicotine Withdrawal Scale (MNWS‐R) administered to measure ‘withdrawal symptoms’ (0‐5 rating scale)	
Study funding	“This work was supported by the Ministry of Science and Higher Education of Poland (grant number N N404 025638). Instrumentation and analytical chemistry at UCSF was supported by the National Institutes of Health, P30 DA012393 and S10 RR026437. The study sponsor had no involvement in the study design, collection, analysis, and interpretation of data, the writing of the manuscript or the decision to submit the manuscript for publication.”	
Author declarations	"MLG was a faculty member of the Medical University of Silesia, Poland during the study. He received a research grant from Pfizer, a pharmaceutical company that markets smoking cessation medications. MLG and NLB have been consultants to pharmaceutical companies that market smoking cessation medications. NLB has been an expert witness in litigation against tobacco companies. The other authors declare no potential conflicts of interest."	
Notes	New for 2020 update	
***Risk of bias***		
**Bias**	**Authors' judgement**	**Support for judgement**
Random sequence generation (selection bias)	High risk	Not randomized
Allocation concealment (selection bias)	High risk	Not randomized
Incomplete outcome data (attrition bias) All outcomes	Low risk	2 dropouts – 1 for nausea, 1 could not complete clinic visits. Analysis based on 20 completers
Selective reporting (reporting bias)	Low risk	All outcomes reported

**Table:** Guillaumier 2018

***Study characteristics***		
Methods	Design: pragmatic, open‐label, single‐centre, 2‐arm randomized controlled trial Recruitment: withdrawal service in Melbourne, Australia Setting: substance use disorder treatment setting, and following discharge, community setting, Melbourne, Australia Study start date: 1 August 2017; Study end date: April 2019	
Participants	Total N: 100 N per arm: EC intervention = 50; NRT control = 50 Inclusion criteria: ≥ 18 years; tobacco smoker on entering the residential service; capacity to consent and able to understand the participant materials Exclusion criteria: used an END containing nicotine in the past month; pregnancy/breastfeeding; currently enrolled in another study; scheduled to be transferred to a long‐term rehabilitation unit following discharge from the residential withdrawal unit. Inclusion based on specific population characteristics: participants were discharged from a smoke‐free alcohol or other drugs (AOD) residential withdrawal service. 32% women; mean age 40.9; mean cpd 21 Motivated to quit: median (SD) = 7.3 (2.4) on the 1 to 10 scale with 10 "highly motivated"	
Interventions	**EC**: Refillable Up to an hour training session, information pack. Innokin Endura T22 starter kit and refill liquid (Nicophar). 4‐week supply of liquid nicotine, with further supplies of liquid nicotine mailed twice at 4‐ week intervals. Dosing schedule of e‐liquid dependent nicotine dependence score: high‐nicotine‐dependence category assigned initial 4‐week e‐liquid supply (total 8 × 10 ml bottles) consisting of: 2 × 10 ml bottles of 18 mg e‐liquid and 6 × 10 ml bottles of 12 mg e‐liquid. The second and third batches = 8 × 10 ml bottles of 12 mg e‐liquid only. Participants scoring in the moderate‐ and low‐dependence categories: three 4‐week supplies of 8 × 10 ml bottles of 12 mg e‐liquid. Participants given 1‐week supply of nicotine patches for use while getting used to the EC. **NRT control**: information pack, 12 weeks NRT on the same schedule as for ENDs. 4‐week supply of patches plus a nicotine spray and inhaler, followed by refills including patches plus inhaler, gum, and lozenges. Both groups received proactive referral to quitline counselling (call‐back service), which provides calls on pre‐discharge and on days 1, 3, 7, 14, and 28 post‐discharge, with an emphasis on relapse prevention. Counsellors trained on the use of ENDs	
Outcomes	Week 6, 12; self‐report Adverse events collected Other outcomes measured: Acceptability and feasibility of interventions Treatment adherence Cigarettes smoked per day ‐ Heaviness of Smoking Index Frequency of cravings Minnesota Nicotine Withdrawal Scale (MNWS) 10‐item Kessler Psychological Distress Scale (Kessler‐10) Quitting self‐efficacy, motivation to quit and the Heaviness of Smoking Index were assessed at baseline.	
Study funding	"The study is supported by a VicHealth Innovation Research Grant (2016–0096). AG is supported by a post‐doctoral fellowship from the Heart Foundation. ALB is supported by an Australian National Health and Medical Research Council (NHMRC) senior research fellowship and a Faculty of Health and Medicine, University of Newcastle Gladys M Brawn senior research fellowship. BB is supported by an Australian NHMRC career development fellowship (GNT1063206) and a Faculty of Health and Medicine, University of Newcastle Gladys M Brawn career development fellowship." "This study was supported by a VicHealth Innovation Research Grant (2016‐0096)."	
Author declarations	"The authors declare that they have no competing interests." "None to declare."	
Notes	New for 2020 update; additional data originally provided by authors and subsequently published	
***Risk of bias***		
**Bias**	**Authors' judgement**	**Support for judgement**
Random sequence generation (selection bias)	Low risk	Quote: “Upon completing the baseline survey, participants were randomised 1:1 to an intervention via a computer‐sequenced 4–6 block randomisation embedded in the tablet device software.”
Allocation concealment (selection bias)	Low risk	Quote: “At the end of the baseline survey, participants will be randomised 1:1 to an intervention via a computer‐sequenced 4–6 block randomisation embedded in the iPad.”
Blinding of participants and personnel (performance bias) All outcomes	High risk	Quote: “Participants were informed of their intervention group by the RA and provided with a training session of up to one hour.” “Due to the nature of the intervention, neither participants nor staff can be blinded to allocation. However, the data safety monitoring committee and the statistician responsible for the data analysis will be blinded.”
Blinding of outcome assessment (detection bias) All outcomes	High risk	No biochemical validation, self‐report data
Incomplete outcome data (attrition bias) All outcomes	Low risk	Quote: “At 6 and 12‐weeks, 63 participants (63%) and 50 participants (50%) were followed up, respectively. While slightly higher retention rates were evidenced in the VNP group at 6‐weeks (68% vs 58% in NRT group; P = 0.30), there were no differences between groups at 12‐weeks (25 re‐contacted in both arms; i.e. 50%).”
Selective reporting (reporting bias)	Low risk	Unpublished findings provided by authors reported on all outcomes mentioned in the protocol.

**Table:** Hajek 2015a

***Study characteristics***		
Methods	Design: prospective cohort, intervention provided Recruitment: people who smoke attending stop‐smoking service Study start date: March 2014; study end date: March 2015 Setting: stop‐smoking service, London, UK	
Participants	Total N: 100 (69 of whom accepted offer of EC) Inclusion criteria: All people who smoked joining stop‐smoking service 38% women (those who accepted), 55% women (those who declined), mean age 41, mean cpd 14, all motivated to quit. EC use at baseline not specified but some who declined EC offer had used EC in the past Motivated to quit: yes E‐cigarette use at baseline: not specified	
Interventions	**EC: Cig‐a‐like and refillable** EC: offered to all people who smoke joining service; offered choice of ‘cig‐a‐like’ (Gamucci, 1.6% or 2.2% nicotine per mL) product or tank model (EVOD, 1.8%; later replaced with Aspire product due to leakage issues). 69% of those offered received an EC on TQD Medication: Offered stop‐smoking medications including NRT and varenicline as in standard protocol. Of EC users, 33% opted to also use NRT, 29% varenicline, 38% nothing Support: weekly, as in standard protocol	
Outcomes	Adverse events collected throughout, method for collection unclear Also collected: 4‐week biochemically validated abstinence, participant feedback, cost	
Study funding	"The pilot study was sponsored by City of London Corporation."	
Author declarations	"Peter Hajek received research funds from and provided consultancy to manufacturers of smoking cessation medications. The remaining authors have no conflicts of interest to declare."	
Notes		
***Risk of bias***		
**Bias**	**Authors' judgement**	**Support for judgement**
Random sequence generation (selection bias)	High risk	Not randomized
Allocation concealment (selection bias)	High risk	Not randomized
Incomplete outcome data (attrition bias) All outcomes	Unclear risk	26% lost in EC group; dropout rate in EC decliners not reported. Reasons for dropout not stated
Selective reporting (reporting bias)	Unclear risk	Unclear which outcomes authors set out to collect, no protocol available

**Table:** Hajek 2019

***Study characteristics***		
Methods	Design: multicentre, pragmatic randomized controlled trial to examine the efficacy of e‐cigarettes compared with nicotine replacement therapy Recruitment: participants attending UK stop‐smoking service and via social media Setting: UK National Health Service stop‐smoking services Study start date: 1 April 2015; study end date: 31 March 2018	
Participants	Total N: 886 N per arm: EC: 439; NRT: 447 Inclusion criteria: Adults who smoke (aged 18 years or over) with no strong preference to use or not to use nicotine replacement or e‐cigarettes, and were currently not using either type of product Able to read/write/understand English Exclusion criteria: Pregnant or breastfeeding Strong preference to use or not use NRT or EC, currently not using either type of product 48% women; median age 41; median cpd 15 ; mean FTND 4.6; 41.5% reported past use of ECs Motivated to quit: not reported	
Interventions	**EC: Refillable** **NRT:** Informed of range of NRT products (patch, gum, lozenge, nasal spray, inhalator, mouth spray, mouth strip, and microtabs) and selected preferred product, encouraged to use combination. Participants free to switch products. Supplies provided for up to 3 months **EC:** Starter pack (1 Kit, Aspire UK) provided along with 30 mL bottle of Tobacco Royale flavour e‐liquid, concentration 18 mg/mL. Participants showed how to use and asked to purchase future e‐liquid online or from local vape shops and to buy different EC device if the 1 provided did not meet their needs. Encouraged to experiment with e‐liquids of different strengths and flavours. If unable to obtain own supply, provided with further 10‐mL bottle (not proactively offered). Oral and written info on how to operate EC Both arms received multi‐session behavioural support as per UK stop‐smoking service practice (one‐to‐one sessions weekly with local clinicians, exhaled CO monitored for at least 4 weeks post‐TQD); signed behavioural contract not to use other therapy for at least 4 weeks	
Outcomes	Weeks 4, 26, and 52 Cessation: sustained and biochemically validated CO < 8 ppm Adverse events and biomarkers: “adverse reactions”: presence or absence of nausea, sleep disturbance and throat and mouth irritation, and respiratory symptoms (presence or absence of shortness of breath, wheezing, coughing, and phlegm), death Other outcomes measured: Use and ratings of trial products Rating of withdrawal symptoms (weeks 1 to 6) Reduction of cigarette consumption Cost‐effectiveness	
Study funding	“Supported by the National Institute for Health Research (NIHR) Health Technology Assessment Programme (project number, 12/167/135) and by a grant (A16893) from the Cancer Research UK Prevention Trials Unit.”	
Author declarations	From ICJME disclosure forms: “Miss Natalie Bisal has nothing to disclose. Dr. Dawkins reports personal fees from Johnson & Johnson, outside the submitted work; Dr. Goniewicz reports personal fees from Johnson and Johnson, outside the submitted work; Dr. Hajek reports grants and personal fees from Pfizer, outside the submitted work; Ms. Li reports grants from NCCHTA, during the conduct of the study; Dr. McRobbie reports grants from NIHR HTA programme, during the conduct of the study; personal fees from Pfizer, personal fees from Johnson & Johnson, outside the submitted work; Dr. Myers Smith has nothing to disclose. Dr. Parrott has nothing to disclose. Dr. Pesola has nothing to disclose. Mrs Anna Phillips‐Waller has nothing to disclose. Dr. Przulj reports grants from Pfizer, outside the submitted work; Dr. Ross has nothing to disclose. Dr. Sasieni has nothing to disclose. Ms. Wu has nothing to disclose."	
Notes	New for 2020 update, listed as ongoing study ISRCTN60477608 in 2016 review update Note higher use of allocated product at 12 m in intervention group compared to control group: “Among participants with 1‐year abstinence, 80% (63 of 79) were using e‐cigarettes at 52 weeks in the e‐cigarette group and 9% (4 of 44) were using nicotine replacement in the nicotine‐replacement group.”	
***Risk of bias***		
**Bias**	**Authors' judgement**	**Support for judgement**
Random sequence generation (selection bias)	Low risk	Quote: “Randomization took place on the quit date to limit differential dropout. Randomization sequences (1:1 ratio in permuted blocks of 20, stratified according to trial site) were generated with the use of a pseudorandom number generator in Stata software and were embedded into an application that only revealed the next treatment assignment once a participant had been entered into the database.”
Allocation concealment (selection bias)	Low risk	Refer to 'Random sequence generation'
Blinding of participants and personnel (performance bias) All outcomes	Low risk	Not blinded, but as both arms contained active interventions, performance bias judged unlikely
Blinding of outcome assessment (detection bias) All outcomes	Low risk	Biochemical validation used
Incomplete outcome data (attrition bias) All outcomes	Low risk	At 12 months: EC arm: 356/439 NRT arm: 342/447
Selective reporting (reporting bias)	Low risk	All prespecified outcomes reported

**Table:** Hajek 2022

***Study characteristics***		
Methods	Design: RCT multicentre Participants: pregnant smokers (12 to 24 weeks gestation) who smoke daily and are interested in stopping smoking Setting: maternity services across the UK. 23 hospital sites across England and one National Health Service Stop Smoking Service in Scotland Recruitment: Recruitment was managed by research midwives in England and by the Stop Smoking Service in Scotland. Participants were identified from patient records and sent study information and invitation letters or invited via telephone, email or text; approached in person when attending antenatal hospital appointments; referred by community midwives or stop‐smoking advisors; or self‐referred via posters advertising the study at the sites’ antenatal clinics. Study start date: 1 May 2017. Study end date: 26 November 2020	
Participants	Total N: 1140 EC arm: 571 NRT arm: (nicotine patches) 569 Inclusion criteria: 12 to 24 weeks pregnant, daily smoker, wants help with stopping smoking. Willing to be randomized to use either NRT or EC and agreeing to use only the allocated stop‐smoking product for at least the first 4 weeks of their quit attempt Exclusion criteria: Allergy to nicotine skin patches. Current daily use of NRT or e‐cigarettes, and serious medical problems or high‐risk pregnancy Inclusion based on specific population characteristics: pregnant women Female 100%; mean age 27; mean CPD 10 E‐cigarette use at baseline: no Motivated to quit: yes	
Interventions	EC: **Refillable** Arm 1: EC Participants were sent an EU Tobacco Product Directive‐compliant refillable e‐cigarette starter kit (One Kit by the UK E‐cig Store), together with two 10 mL bottles of tobacco‐flavoured e‐cigarette liquid (1.8% nicotine; 70% propylene glycol and 30% vegetable glycerol), a pack of 5 replacement coils, and an instruction leaflet (Supplementary Data, Appendix 5). Further supplies of e‐cigarette liquid were posted on request for up to 8 weeks. A lower‐strength e‐cigarette liquid (1.1%) and e‐cigarette liquid with fruit flavour were available as alternatives. Participants were encouraged to source e‐cigarette liquids of the strength and flavour they liked, as well as different e‐cigarette devices, and arrange their own supplies after 8 weeks if needed. The cost of the kit provided by the study was GBP 22.75 and the cost of e‐cigarette liquid was up to GBP 24 for an 8‐week supply. Products used during the initial 4 weeks (n = 344) # * N (%): refillable e‐cigarettes 324 (94.2%); cig‐a‐like 1 (0.3%); cartridge/pod 1 (0.3%); information missing 18 (5.2%) Nicotine strength N (%): 0 mg/mL 7 (2.0); 1 to 10 mg/mL 47 (13.7); 11 to 20 mg/mL 199 (57.9); information missing 91 (26.5) Arm 2: NRT ‐ Nicotine patches Participants were sent an initial 2‐week supply of Nicorette Invisi 15 mg 16 h nicotine patches with manufacturer instruction leaflets and instructed to apply patches every day upon waking, and remove them before bedtime. Further supplies were posted on request for up to 8 weeks. A lower strength patch (10 mg 16 h) was available as an alternative. Participants were encouraged to access further supplies themselves via their general practitioner or local Stop Smoking Service. This could be patches and/or other NRT products such as nicotine chewing gum, inhalator or mouth spray, to use in addition to the patch alone if needed. In the United Kingdom, pregnant women who smoke receive NRT free of charge. Behavioural support was given that accompanied both study arms. Participants received 6 phone calls from stop‐smoking advisors who followed the practice of the UK Stop Smoking Service 61.	
Outcomes	Baseline, weeks 1 to 4 after target quit date (TQD) (phone call), end of pregnancy (EOP) at least 6 months (saliva and CO), 3 months post‐partum (phone call) Cessation: saliva samples and carbon monoxide readings collected AEs and SAEs Continued use of study product Flavours EC nicotine strength	
Study funding	The study was funded by the National Institute of Health Research, Health Technology Programme (ref. no. 15/57/85). The funder had no role in the design and conduct of the study; collection, management, analysis, and interpretation of the data; preparation, review, or approval of the manuscript; or the decision to submit the manuscript for publication. For part of the trial, F.P. was supported by Cancer Research UK (grant no. C8162/A25356).	
Author declarations	P.H. provided consultancy to and received research funding from Pfizer. D.P. received research funding from Pfizer. H.M. has received honoraria for speaking at smoking cessation educational events and sitting on an advisory board organized by Pfizer. All other authors have no competing interests.	
Notes	New to 2022 update	
***Risk of bias***		
**Bias**	**Authors' judgement**	**Support for judgement**
Random sequence generation (selection bias)	Low risk	Quote: "An independent statistician developed the randomization sequence using permuted block randomization with a block size of at least 6 and a maximum of 12".
Allocation concealment (selection bias)	Low risk	Quote: "The randomization list was accessible only to the independent statistician, on a secure server. Researchers conducting randomization used the database application to inform the participants of their study arm allocation. Researchers conducting follow‐up calls were blind to treatment allocation until the follow‐up contact was made".
Blinding of participants and personnel (performance bias) All outcomes	Low risk	Participants received equally intensive interventions.
Blinding of outcome assessment (detection bias) All outcomes	Low risk	Quote: "Researchers conducting follow‐up calls were blind to treatment allocation until the follow‐up contact was made. Once contact was made and the trial application was opened, condition‐specific questions were visible on the computer screen. The trial statistician was blind to participant allocation until the analysis of the primary and secondary outcomes was complete. This was achieved by extracting and importing into Stata only the baseline characteristics, study arm and smoking status variables in the first stage of the analysis. Variables coding treatment adherence and product use were extracted only after the primary and secondary outcome analyses were completed".
Incomplete outcome data (attrition bias) All outcomes	Low risk	EC arm 515/571; NRT arm 495/569
Selective reporting (reporting bias)	Low risk	Analysis prespecified

**Table:** Halpern 2018

***Study characteristics***		
Methods	Design: randomized clinical trial Recruitment: eligible participants were employees and their spouses at 54 companies that used Vitality wellness programmes. Setting: online resources via workplace setting (54 companies), USA Study start date: first phase of recruitment October 2014, second phase November 2015 (to meet recruitment target); study end date: 20 April 2017	
Participants	Total N: 6006 N per arm: usual care: 813; free e‐cigarettes: 1199; free cessation aids: 1588; reward incentives plus free cessation aids: 1198; redeemable deposit plus free cessation aids: 1208 Inclusion criteria: At least 18 years old Reported current smoking on a health risk assessment within the previous year Employees and their spouses that used Vitality wellness programmes Exclusion criteria: Participants who express wanting to opt out of this programme 51.1% women; median age 44; median cpd 10 E‐cig use at baseline: 10.7% current use; 23.1% past but not current use; 39.7% never used ECs Motivated to quit: unselected sample (total sample): 9.2% no plan to quit; 61.6% want to quit later; 27.7% want to quit/need help	
Interventions	**EC: Cig‐a‐like** a) **Usual care:** Standardized Vitality programme aimed at promoting tobacco cessation. This programme includes existing employee benefits for quitting and the use of text/email messages to encourage tobacco cessation. b) as (a), plus **free EC**: Free NJOY e‐cigarettes (including battery sticks, a USB charger, and up to 20 chambers with 1.0% to 1.5% nicotine per week in participants’ chosen flavours). Use of all products was free until 6 months after the quit date. c) as (b) plus access to free NRT, bupropion or varenicline d) as (c) plus incentives across 6 m for testing negative for tobacco use e) as (c) plus provide money at start and lose money from this fund if they do not test negative across 6 m	
Outcomes	Months 1, 3, 6, and 12 Cessation: sustained smoking abstinence for 6 months, biochemical validation (urine cotinine, anabasine and blood carboxyhaemoglobin) Other outcomes measured: costs	
Study funding	"Supported by a grant from the Vitality Institute to the University of Pennsylvania Center for Health Incentives and Behavioral Economics."	
Author declarations	"Disclosure forms provided by the authors are available with the full text of this article at NEJM.org. Check these and: Dr. Troxel reports others from VAL Health, outside the submitted work. Dr. Volpp reports grants and personal fees from CVS Health, personal fees from VAL Health, grants from Humana, grants from Merck, grants from Weight Watchers, grants from Hawaii Medical Services Association, grants from Oscar Health Insurance, outside the submitted work. All of the other authors state that they have nothing to disclose."	
Notes	New for 2020 update. Study listed as ongoing study NCT02328794 in 2016 review update Only arms (a) and (b) included in our analyses	
***Risk of bias***		
**Bias**	**Authors' judgement**	**Support for judgement**
Random sequence generation (selection bias)	Unclear risk	Not specified
Allocation concealment (selection bias)	Unclear risk	Not specified
Blinding of participants and personnel (performance bias) All outcomes	High risk	Not blinded and different amounts of support given to each group
Blinding of outcome assessment (detection bias) All outcomes	Low risk	Biochemical validation
Incomplete outcome data (attrition bias) All outcomes	High risk	At 12 months, very low numbers completed biochemical validation. Submitted a sample n = CG: 1, free e‐cigs; 4, free cessation: 5, rewards: 14, deposits: 16
Selective reporting (reporting bias)	Low risk	Expected outcomes reported and checked with trial registration

**Table:** Hatsukami 2020

***Study characteristics***		
Methods	Design: randomized trial Recruitment: media advertisements Setting: clinic visits in community, USA Study start date: 25 November 2014; study end date: 2 December 2018	
Participants	Total N: 264 N per arm: usual brand: 36; AD‐E: 76; CS‐E: 76; CS‐NRT: 76 Inclusion criteria: ≥ 18 years of age; smoking ≥ 5 cpd for the past year with a breath CO ≥ 10 ppm or NicAlert test = level 6 if CO < 10 ppm; unstable physical and mental health Exclusion criteria: serious quit attempt in the past 3 months; recent (< 3 months) alcohol or drug abuse problems; regular use of other nicotine or tobacco products (e.g. > 9 days per month to minimize confounding effects of these products on biomarker outcomes); planning to quit smoking in the next 3 months; chronic conditions affecting results of biomarker analyses (e.g. liver disease); using NRT or other cessation medications; pregnancy/breastfeeding 49% women; mean age 45.2; mean cpd 15.2; mean FTND 3.4 E‐cigarette use at baseline: not reported Motivated to quit: initially uninterested	
Interventions	**EC: Cig‐a‐like**, but the only cig‐a‐like product with high nicotine content **Usual brand** arm: purchased their own usual brand of cigarettes; at end of clinical trial phase (week 8), offered ECs or NRT for up to 8 weeks, with a choice of product and no specific instructions for use **EC AD‐E** arm: use EC whenever you like instead of a cigarette; can smoke as many or as few cigarettes as you want **EC CS‐E** arm: complete substitution with e‐cigarettes (i.e. “you will stop smoking cigarettes and use only e‐cigarettes”) The primary e‐cigarette product was Vuse Solo (4.8% nicotine, manufactured by RJ Reynolds, Inc). Initially, a choice of Blu cigarettes (cartridge‐based system, marketed previously by Lorillard) and Fin (prefilled tanks system, manufactured by Fin Branding Group) was offered; but because Vuse attained the highest market share during the early phase of the study, they switched exclusively to Vuse. Participants could choose 1 of 4 flavours: tobacco, mint, menthol, and berry. Participants were provided 7 cartridges a week with the option of returning to the clinic before their next visit to obtain additional cartridges if needed. All products provided free to the participants. All unused products and used EC cartridges were collected at each visit. **CS‐NRT** arm: complete substitution with 4 mg nicotine gum or lozenge, with the participant choosing what product they would like to use (i.e. “you will stop smoking cigarettes and use only nicotine gum or lozenge”). The 4 mg was down‐titrated to 2 mg if adverse side effects were experienced. Nicotine gum came in mint, cinnamon, and fruit flavours, while the nicotine lozenge was mint or cherry flavours. All these products were provided free to the participants and unused products were collected at each visit. Behavioural support: **CS‐E arm** and **CS‐NRT arm**: received brief counselling on how to avoid smoking cigarettes	
Outcomes	2‐week baseline period (weeks ‐1 and 0) Week 1, 2, 3, 4, 6, and 8 Adverse events and biomarkers: Urinary total nicotine equivalents (total nicotine + total cotinine + total 3′‐hydroxycotinine; TNE) Exhaled CO Urinary 4‐(methylnitrosamino)‐1‐(3‐pyridyl)‐1‐butanol and its glucuronides (total NNAL, biomarker for NNK) Urinary phenanthrene tetraol (PheT, an indicator of carcinogenic polycyclic aromatic hydrocarbons) Urinary metabolites of VOCs (mercapturic acids)—2‐cyanoethylmercapturic acid (CEMA, biomarker for acrylonitrile), 3‐hydroxypropylmercapturic acid (3‐HPMA, biomarker for acrolein), 3‐hydroxy‐1‐methylpropylmercapturic acid (HMPMA, biomarker for crotonaldehyde/methylvinyl ketone), 2‐hydroxypropylmercapturic acid (2‐HPMA, biomarker for propylene oxide), and N‐acetyl‐S‐(carbamoylethyl)‐L‐cysteine(AAMA, biomarker for acrylamide) A safety check for adverse events was conducted at week 20 follow‐up Blood pressure, heart rate, and oxygen saturation Other outcomes measured: Cessation (< 6 months)	
Study funding	"supported by grants U19CA157345 from the National Cancer Institute (DKH/PS), UL1 TR000062 and UL1 TR002494 from the National Center for Advancing Translational Science of the National Institutes of Health, and T32 DA007097 from the National Institute of Drug Abuse (EM). The content is solely the responsibility of the authors and does not necessarily represent the official views of the funding agencies".	
Author declarations	"RJC is a member of the FDA Tobacco Products Scientific Advisory Committee. PGS serves or has served as an expert witness in tobacco company litigation on behalf of plaintiffs".	
Notes	New for 2020 update. AD‐E arm not included in this review Additional data provided from authors	
***Risk of bias***		
**Bias**	**Authors' judgement**	**Support for judgement**
Random sequence generation (selection bias)	Unclear risk	Not specified
Allocation concealment (selection bias)	Unclear risk	Not specified
Blinding of participants and personnel (performance bias) All outcomes	Unclear risk	Not blinded and some interventions contained different levels of support
Blinding of outcome assessment (detection bias) All outcomes	Low risk	Not blinded but all relevant outcomes for our analyses were objective
Incomplete outcome data (attrition bias) All outcomes	Low risk	Quote: “There was a significant difference in dropout rates across groups following study entry (P = 0.04), with the highest dropout rates observed in the complete substitution groups, particularly in the NRT group…” AD‐E: week 1 = 73/76; week 2 = 73/76; week 4 = 69/76; week 6 = 66/76; week 8 = 65/76 = 85% CS‐E: week 1 =69/76; week 2 = 67/76; week 4 = 66/76; week 6 = 61/76; week 8 = 58/76 = 69.7% CS‐NRT: week 1 =72/76; week 2 = 65/76; week 4 = 60/76; week 6 = 57/76; week 8 = 53/76 = 69.7% UB: week 1 = 35/36; week 2 = 35/36; week 4 = 33/36; week 6 = 33/36; week 8 = 32/36 = 88.8%
Selective reporting (reporting bias)	Low risk	Table in the supplementary section notes that heart rate, blood pressure, and oxygen levels were measured, but findings not reported in paper; however, provided by authors upon request

**Table:** Hickling 2019

***Study characteristics***		
Methods	Design: single‐group assignment – pre‐test post‐test pilot study Recruitment: Participants were referred from community mental health teams within the South London and Maudsley NHS Foundation Trust. Setting: healthcare setting, UK Study start date: 24 September 2014; Study end date: 2 May 2017	
Participants	Total N: 50 Inclusion criteria: 18 to 70 years; daily smoker (unwilling to quit soon); exhaled CO ≥ 5 ppm; established clinical diagnosis of schizophreniform, schizophrenia, schizoaffective disorder or bipolar disorder, or attending an early detection service in a high‐risk state Exclusion criteria: use of EC ≥ 2 occasions in past 30 days; intention to quit smoking in the next 30 days; medication use that may reduce smoking (including, bupropion, nicotine replacement therapies, acamprosate, varenicline, baclofen, clonidine, naltrexone, buprenorphine, nortriptyline, disulfiram and anti‐seizure medications); hospitalisation/change in dose of psychotropic medication(s) in the last 30 days; unstable physical health in the past 3 months; previous serious stomach ulcer and/or phaeochromocytoma; severe heartburn, stroke, unstable kidney/liver disease, an uncontrolled overactive thyroid gland past 3 months; meet the Diagnostic and Statistical Manual of Mental Disorders, Fourth Edition (DSM‐IV) criteria for illicit/alcohol drug dependency; contraindications to nicotine; asthma; suicidal ideation/suicide attempt in the past month; pregnancy Inclusion based on specific population characteristics: people who smoke tobacco with a psychotic disorder (established clinical diagnosis of schizophreniform, schizophrenia, schizoaffective disorder or bipolar disorder, or attending an early detection service in a high‐risk state) 24% women; mean age 38.96; mean cpd 17.94; mean FTND not reported Motivated to quit: “unwilling to quit soon” E‐cigarette use at baseline: must not have used e‐cigarettes on more than 2 occasions in the past 30 days	
Interventions	**EC: Cig‐a‐like** Participants provided with free tobacco‐flavoured NJOY traditional bold disposable e‐cigarette (4.5% nicotine) in an "amount equivalent to 150% of their daily tobacco use (as recommended by the manufacturer)" for 6 weeks. Participants were instructed in the use of EC; not required to stop smoking tobacco, but were encouraged to replace it with EC as much as possible. Followed up at 4 weeks and encouraged to continue EC use, informed about EC types and where these could be purchased	
Outcomes	Weeks 1, 2, 3, 4, 5, 6, 7, 8, 9, 10, 24 Self‐reported and biochemical validation Cessation: tobacco use, as measured by the Time Line Follow Back. Tobacco cigarette use was also indexed weekly by measuring exhaled CO levels with a Smokerlyzer ED50 CO meter (Bedfont Instruments, UK) Adverse events and biomarkers: Side effects associated with e‐cigarette use – reported weekly Respiratory symptoms: lung capacity (measured by Wright’s Mini Peak‐flow Meter (Clement Clarke International Ltd., UK) at baseline, weeks 6, 10, and 24; peak flow was obtained 3 times at each assessment Heart rate and blood pressure Occurrence of (serious) adverse events was assessed on a weekly basis. In a subsample of participants (N = 8), 3‐hydroxypropylmercapturic acid (3‐HPMA, a measure of the toxicant acrolein) and formic acid were measured at baseline and week 6. These participants were chosen as their tobacco intake had decreased by more than 50% in this period. The measurement of 3‐HPMA and formic acid was also performed by validated LC‐MS/MS assays. Other outcomes measured: Urinary cotinine Weight Motivation to Stop Scale (MTSS) Smoking Consequences Questionnaire‐Adult (SCQ‐A) Positive and Negative Syndrome Scale (PANSS) Calgary Depression Scale for Schizophrenia (CDSS)	
Study funding	"This work was funded by the Maudsley Charity (grant number 715); and supported by the National Institute for Health Research (NIHR) Biomedical Research Centre at South London and Maudsley NHS Foundation Trust and King’s College London."	
Author declarations	"R.P‐I. has received honoraria and speaker support from Lundbeck. L.D. has provided consultancy for the pharmaceutical industry (Johnson & Johnson 2015, 2017) and acted as an expert witness for an e‐cigarette patent infringement case (Porzio, Bromberg & Newman Attorneys at Law, 2015). Between 2011 and 2013, she conducted research for several independent electronic cigarette companies (Totally Wicked, SKYCIGS and E‐Lites) for which the University of East London received funds. The e‐cigarette companies involved had no input into the design, conduct or write up of these projects and she has not received any funds from e‐cigarette companies in the last 4 years. She has no links with, and has not received any funds from, the tobacco industry, although two e‐cigarette companies that she worked with in 2013 were subsequently acquired by the tobacco industry (SKYCIGs and E‐Lites). L.H., T.R., K‐V.S., J.M., A.M. and P.M. have no conflicts of interest."	
Notes	Study listed as ongoing study NCT02212041 in the 2016 review update Additional data provided from authors	
***Risk of bias***		
**Bias**	**Authors' judgement**	**Support for judgement**
Random sequence generation (selection bias)	High risk	Uncontrolled study
Allocation concealment (selection bias)	High risk	Uncontrolled study
Incomplete outcome data (attrition bias) All outcomes	Low risk	Follow‐up: week 6: 46/50; week 10: 42/50; week 24: 40/50
Selective reporting (reporting bias)	Low risk	Reported all outcomes listed on clinical trials.gov [http://clinical trials.gov] except NNAL. Authors confirmed that they had intended to test for NNAL but had major issues with the assays.

**Table:** Holliday 2019

***Study characteristics***		
Methods	Design: pilot RCT Recruitment: recruited via the Newcastle Dental Hospital and by primary care practitioners working in the north‐east England region Setting: dental clinical research facility (DCRF), located in the Newcastle Dental Hospital, Newcastle upon Tyne, UK Study start date: 20 September 2016; study end date: 31 July 2018	
Participants	Total N: 80 N per arm: Intervention group: 40; control group: 40 Inclusion criteria: Aged over 18 years old; smoker (≥ 10 cigarettes/day) Willing and able to come to the DCRF for the required study visits Having a minimum of 16 natural teeth (excluding third molars) Being diagnosed with periodontitis Exclusion criteria: Having used an e‐cigarette for more than 2 days in the last 30 days Infectious or systemic diseases that may be unduly affected by participation in this study Haemodynamically unstable Patients taking the medication adenosine (due to drug interaction risk) Lack of capacity to be able to consent to the research project or inability to follow study instructions, or both Participation in a dental research study within the previous 20 days Pregnant by medical history, or nursing Received any non‐surgical periodontal therapy other than a routine scale and polish in the last 6 months Currently undergoing or requiring extensive dental, orthodontic or implant treatment, or treatment for peri‐implantitis Inclusion based on specific population characteristics: periodontitis 52.5% women; mean age 44.36; mean cpd 17.4; mean FTND 5 Motivated to quit: not selected on motivation and not reported E‐cigarette use at baseline: not currently using an e‐cigarette, or not having used 1 for more than 2 days in the last 30 days	
Interventions	**EC: Refillable** All participants given standard stop‐smoking advice (10 to 15 minutes in duration) and offer of referral to stop‐smoking services **Intervention**: given EC starter kit (Vype eTank clearomizer) and brief training on its use by a dentist. Provided with an approximately 2‐week supply of e‐liquid (20 ml) with a choice of flavour (Blended Tobacco, Crisp Mint, Dark Cherry and Vpure (flavourless)) and nicotine strength (0 mg/mL, 6 mg/mL, 12 mg/mL, 18 mg/mL) and information on where to buy more. EC intervention delivered directly following the standard stop‐smoking advice and was expected to be 10 to 15 minutes in duration. **Control group**: no further intervention	
Outcomes	Months 1 and 6; self‐report and biochemical validation of smoking status Cessation: rates of continuous eCO‐verified smoking abstinence at 6 months were calculated following the Russell Standard (RS6) Adverse events and biomarkers: expired air CO, adverse events monitored at each study visit Other outcomes measured: Feasibility outcomes Oral health outcomes Smoking behaviour outcomes comprised: self‐reported tobacco and e‐cigarette use, eCO, e‐salivary cotinine, salivary anabasine, FTND and Mood and Physical Symptoms Scale	
Study funding	"Richard Holliday is funded by a National Institute for Health Research Doctoral Research Fellowship (DRF‐2015‐08‐077). This paper presents independent research funded by the National Institute for Health Research (NIHR). The views expressed are those of the authors and not necessarily those of the NHS, the NIHR or the Department of Health and Social Care."	
Author declarations	"The authors declare that they have no competing interests."	
Notes	New for 2020 update	
***Risk of bias***		
**Bias**	**Authors' judgement**	**Support for judgement**
Random sequence generation (selection bias)	Low risk	Randomization was performed using a secure password‐protected web‐based system.
Allocation concealment (selection bias)	Low risk	Quote: "The randomisation allocation schedule will be generated by a statistician with no other involvement in the study to achieve concealment of allocation."
Blinding of participants and personnel (performance bias) All outcomes	High risk	Nature of study precluded blinding; different levels of support across intervention arms
Blinding of outcome assessment (detection bias) All outcomes	Low risk	Biochemical validation
Incomplete outcome data (attrition bias) All outcomes	Low risk	Attrition < 50%
Selective reporting (reporting bias)	Low risk	All prespecified outcomes were reported.

**Table:** Humair 2014

***Study characteristics***		
Methods	Design: prospective cohort Recruitment: people attending an outpatient clinic Setting: university hospital outpatient clinic, Switzerland Study start date/end date: not specified	
Participants	Total N: 17 Inclusion criteria: Wish to reduce tobacco use or had failed to stop smoking using varenicline, bupropion or NRT in the past Inclusion based on specific population characteristics: no Mean 23 cpd, 82% had a psychiatric illness Motivated to quit: yes E‐cigarette use at baseline: not specified	
Interventions	**EC: Cig‐a‐like** Offered an EC with nicotine 59% also reported using NRT or varenicline in addition to EC.	
Outcomes	Smoking cessation and reduction by at least 30% at 12 months (self‐report) Adverse events No significant side effects	
Study funding	Not specified	
Author declarations	Not specified	
Notes	Abstract only, hence little detail available Not clear if EC was provided by clinic or if participants had to buy their own	
***Risk of bias***		
**Bias**	**Authors' judgement**	**Support for judgement**
Random sequence generation (selection bias)	High risk	Prospective cohort
Allocation concealment (selection bias)	High risk	Not randomized
Incomplete outcome data (attrition bias) All outcomes	Unclear risk	Numbers lost to follow‐up not reported
Selective reporting (reporting bias)	Unclear risk	Unable to determine prespecified outcomes

**Table:** Ikonomidis 2018

***Study characteristics***		
Methods	Design: (acute phase) randomized, cross‐over assignment (outcomes measured within hours of the intervention and hence do not meet the criteria of 1 week or more); chronic phase: non‐randomized, single‐group assignment Recruitment: hospital smoking cessation unit Setting: hospital smoking‐cessation unit, Greece Study start date: 31 January 2017; study end date: estimated completion date: December 2021	
Participants	Total N: 90 Inclusion criteria: Active conventional cigarette smoker Adults 18 to 60 years Exclusion criteria: Health condition adversely affected by smoking, history or presence of cardiovascular disease Inclusion based on specific population characteristics: no 54% women; mean age 50.2; mean cpd 23.4; mean FTND: not reported Motivated to quit: yes – recruited from smoking cessation unit E‐cigarette use at baseline: not reported	
Interventions	**EC: not clear** E‐cigarette details: in the chronic phase, all 70 participants were instructed to replace their conventional cigarettes (con‐cig) with an e‐cig containing nicotine (12 mg/dL (e‐cig fluid with nicotine concentration of 12 mg/mL (propylene glycol 74.3%, glycerin 20%, flavouring 4.5%, nicotine 1.2%))) for 1 month.	
Outcomes	1 month; self‐report and objective measures Cessation: self‐report cessation at 1 month. CO measured at 1 month. Cessation data not used as < 6 months Adverse events and biomarkers: Exhaled CO concentration Heart rate; blood pressure Other outcomes measured: Oxidative stress as assessed by malondialdehyde (MDA) plasma concentrations Aortic stiffness as assessed by pulse wave velocity (PWV) and augmentation index (AIX75)	
Study funding	This study was supported by a grant from the Hellenic Cardiology Society and Hellenic Society of Lipidiology and Atherosclerosis.	
Author declarations	None	
Notes	New for 2020 update. Acute phase of trial not relevant for the review as very short‐term outcomes.	
***Risk of bias***		
**Bias**	**Authors' judgement**	**Support for judgement**
Random sequence generation (selection bias)	Unclear risk	Not specified
Allocation concealment (selection bias)	Unclear risk	Not specified
Blinding of participants and personnel (performance bias) All outcomes	High risk	Not blinded and differential levels of support given
Blinding of outcome assessment (detection bias) All outcomes	Unclear risk	Objective measures used for all outcomes reported
Incomplete outcome data (attrition bias) All outcomes	Low risk	70 participants and 20 controls recruited – no dropout
Selective reporting (reporting bias)	Unclear risk	NCT record stated that chronic endothelial integrity, platelet aggregation and high‐shear stress‐dependent platelet function would be assessed but these outcomes were not reported in this research letter.
Other bias	Unclear risk	Few details – written as commentary. Trial registration suggests this is an ongoing study.

**Table:** Ikonomidis 2020a

***Study characteristics***		
Methods	RCT Recruitment: smoking cessation clinic of second cardiology department of National and Kapodistrian University of Athens, Attikon General Hospital Setting: hospital smoking‐cessation unit, Greece Study start date: NS	
Participants	N = 40; Arm 1 e‐cigarette n = 20; Arm 2 conventional tobacco cigarette n = 20 80% female; mean age 44.8 (SD 11.3); mean cpd: 25.8 (C‐cig 25.5 (SD 9.3), E‐cig: 26.2 (SD 9.1)) Inclusion criteria: smokers without cardiovascular disease, who used to smoke 25.8 ± 9.2 conventional cigarettes per day of their choice Exclusion criteria: abnormal renal function; hepatic failure (bilirubin > 2 mg/dL); active malignancy; people treated with drugs that affect platelet function; history of coronary artery disease or peripheral artery disease; history of cardiomyopathy; thrombocytopenia (PLTs < 100 × 109 /L); anaemia (HCT < 28%); alcohol or drug abuse; age < 21 years; pregnancy; risk factors for cardiovascular disease	
Interventions	**EC: Refillable** E‐cig: second‐generation e‐cig device and popular in Greek Market e‐liquid (NOBACCO eGo Epsilon BDC 1100, eGo battery, 1100 mAh, operating at 3.9 V ‐ propylene glycol 74.3%, glycerin 20%, flavouring 4.5%, nicotine 1.2%/12 mg/mL)	
Outcomes	Baseline, 4 months: exhaled CO concentration; blood pressure Also, cpd; platelet function by Platelet Function Analyzer PFA‐100 and Light Transmission Aggregometry; pulse wave velocity; plasma malondialdehyde levels as oxidative stress index	
Study funding	"There was no funding for this study".	
Author declarations	"The authors declare that they have no known competing financial interests or personal relationships that could have appeared to influence the work reported in this paper."	
Notes		
***Risk of bias***		
**Bias**	**Authors' judgement**	**Support for judgement**
Random sequence generation (selection bias)	Low risk	Table of random numbers as reproduced from the online randomization software www.graphpad.com/quickcalcs/index
Allocation concealment (selection bias)	Unclear risk	Not specified
Blinding of participants and personnel (performance bias) All outcomes	Unclear risk	Not possible
Blinding of outcome assessment (detection bias) All outcomes	Low risk	Only CO outcomes used here, which were objectively measured
Incomplete outcome data (attrition bias) All outcomes	Low risk	All followed up (confirmed via contact with authors)
Selective reporting (reporting bias)	Unclear risk	No protocol or clinical trial record available to confirm whether all prespecified criteria were reported

**Table:** Ikonomidis 2020b

***Study characteristics***		
Methods	Design: RCT Setting: Smoking Cessation Clinic of Second Cardiology Department of National and Kapodistrian University of Athens, Attikon General Hospital, Greece	
Participants	40 Inclusion criteria: current smokers without cardiovascular disease	
Interventions	EC type: NS Conventional cigarette (conv‐cig) or an electronic cigarette (e‐cig) with nicotine concentration 12 mg/dL for 1 month	
Outcomes	a) Perfused boundary region (PBR) of the sublingual arterial micro vessels (range 5 to 25 micrometres), a marker inversely related with glycocalyx thickness b) Pulse wave velocity (PWV), central systolic blood pressure (cSBP), and augmentation index (AIX) c) Platelet function by 2 different methods, namely the novel Platelet Function Analyzer PFA‐100 and the traditional Light Transmission Aggregometry (LTA) d) Exhaled CO level (ppm) as a smoking status marker e) Plasma malondialdehyde (MDA) levels, as an oxidative stress burden index	
Study funding	Funding acknowledgement: type of funding source: none	
Author declarations	NS	
Notes	Information extracted from a conference abstract	
***Risk of bias***		
**Bias**	**Authors' judgement**	**Support for judgement**
Random sequence generation (selection bias)	Unclear risk	No detail given Quote: "40 current smokers (mean age 48 years±5) without cardiovascular disease were randomized to smoke either a conventional cigarette (conv‐cig) or an electronic cigarette (e‐cig) (electronic cigarette fluid with nicotine concentration of 12 mg/dL) for one month."
Allocation concealment (selection bias)	Unclear risk	NS
Blinding of participants and personnel (performance bias) All outcomes	High risk	No blinding
Blinding of outcome assessment (detection bias) All outcomes	Unclear risk	NS
Incomplete outcome data (attrition bias) All outcomes	Unclear risk	NS
Selective reporting (reporting bias)	High risk	Results reported in summary form, not fully, with only some significant changes reported. Information taken from a conference abstract only

**Table:** Ioakeimidis 2018

***Study characteristics***		
Methods	Design: randomized controlled trial Recruitment: not specified Setting: hospital, Greece Study start date/Study end date: not specified	
Participants	Total N: 54 N per arm: arm 1: 27; arm 2: 27 Inclusion criteria: ≥ 10 cpd; motivation to quit; hospitalized with acute coronary syndrome (ACS); ≥ 18 years Exclusion criteria: prior EC use; history of neuropsychiatric disorders; prior varenicline use or use of smoking cessation pharmacotherapy at time of ACS; cardiogenic shock or renal impairment; hepatic impairment prior to ACS; excessive alcohol use or current use of marijuana or non‐cigarette tobacco products Inclusion based on specific population characteristics: People who have experienced acute coronary syndrome 65% women; mean age 52; mean cpd 21; mean FTND 5.6 Motivated to quit: yes E‐cigarette use at baseline: no prior EC use	
Interventions	**EC:** information on whether cig‐a‐like or refillable not provided Both arms given "low intensity counselling" **Intervention 1:** 12‐week use of EC 12 mg/mL nicotine **Intervention 2:** 12‐week varenicline	
Outcomes	Weeks: 4, 12, 24 Cessation: 7‐day PP at 24 weeks, self‐report Adverse events and biomarkers: unclear how these were reported. Abstract says no SAEs, poster implies this may have just been CV or neuropsychiatric SAEs. Abstract says nothing about AEs but nausea and sleeping disorders given in table in poster. Implies (S)AEs collected during treatment period only Other outcomes measured: not specified	
Study funding	Not reported	
Author declarations	Not reported	
Notes	New for 2020 update. Abstract and poster only; limited data available	
***Risk of bias***		
**Bias**	**Authors' judgement**	**Support for judgement**
Random sequence generation (selection bias)	Unclear risk	Not specified
Allocation concealment (selection bias)	Unclear risk	Not specified
Blinding of participants and personnel (performance bias) All outcomes	Low risk	Not specified but equal amounts of contact and support between arms so performance bias judged unlikely
Blinding of outcome assessment (detection bias) All outcomes	Low risk	Self‐report only but equal amounts of contact between arms; no reason to suspect differential misreport
Incomplete outcome data (attrition bias) All outcomes	Unclear risk	Not specified
Selective reporting (reporting bias)	Unclear risk	Abstract/poster only so not able to judge
Other bias	High risk	Abstract and poster only. Two different figures presented for quit rate in EC arm (no difference in those presented in varenicline arm) between abstract and poster. Poster percentage aligns with figure, so using that (16.5%) as opposed to abstract figure (32.5%). Contacted authors but no reply. Calculated n quit based on percentages but unclear what denominators were; EC calculates back to whole number for EC but not for varenicline.

**Table:** Kanobe 2022*

***Study characteristics***		
Methods	Design: 2‐centre, randomized, controlled, switching, open‐label, parallel‐group, clinical confinement study Higher versus lower nicotine study Setting: USA. Two clinical research sites (Overland Park, KS and Minneapolis, MN), contracted by Altasciences Clinical Research as the contract research organization (CRO) Recruitment: potential participants completed a pre‐screening telephone interview and also attended a screening visit at the clinical research site within 30 days before study entry. Study start date: April 2017. Study end date: September 2017	
Participants	125 randomized: Kanobe 2022 reports EC Vuse Solo arm EC (Vuse Solo) group: 35 Female 42.9% female; mean age 41.2 years; mean CPD: 18.3 (SD 10.9); mean FTND: 6.1 (SD 1.6) Kanobe 2023 reports on Vuse Vibe and Vuse Ciro arms: 37 Vuse Vibe group: 37 37 Vuse Ciro group: 37 35.6% (32) female; mean age 40 [Abstinence arm: 16 (this arm is ignored)] Inclusion criteria: M or F; 21 to 64 yrs; smoker using non‐menthol tobacco cigarettes (at least 10 cpd); not planning to quit Exclusion criteria: medical conditions; weight of ≤ 110 pounds; planning to quit within 30 days EC use at baseline: no Motivated to quit: no	
Interventions	EC: Vuse Solo Original ENDS product referred to as Vuse Solo (G2) or Vuse Solo. Pod. Rechargeable Vuse Solo power unit (battery capacity: ≥ 270 mAh; wattage: 3.00 W) and a closed cartridge containing 0.5 mL of original (tobacco‐flavoured) e‐liquid, a mix of propylene glycol (PG), glycerin (PG:glycerin ratio of 21:79), water, and flavouring ingredients. The e‐liquid included nicotine at 4.8% nicotine by weight (57.4 mg/mL) and contained nicotine salts. EC: Vuse ciro 1.5% nicotine EC. pre‐filled, closed ENDS products (RJR Vapor Company, Winston‐Salem, NC, USA. Original, tobacco‐flavored, nicotine containing e‐liquids. The Vuse Ciro product was originally marketed under the brand name “Vuse Solo+”. The product was subsequently renamed to “Vuse Ciro”. Tobacco flavour. EC: Vuse vibe, 3% nicotine EC. The Vuse Vibe power unit is powered by a ≥ 550 ‐mAh rechargeable battery and is paired with a cartridge containing 1.9 mL of e‐liquid with 3% nicotine content by weight (36 mg/mL) and a propylene glycol‐to‐glycerin ratio (PG/VG) of 20/80. The maximum puff duration for Vuse Vibe is 6 s. Tobacco flavour	
Outcomes	Day 1, 5, 7 (urine) AEs, SAEs monitored throughout study from day 1 to day 8 (discharge) Most outcomes were measured at day 5 and not eligible for extraction.	
Study funding	“This study was funded by RAI Services Company.“ Tobacco industry funded. Parent company is Reynolds American. Reynolds American manufacture and market a variety of tobacco products, including cigarettes (Newport, Camel, Pall Mall, Kent, Doral, Misty, Capri, and Natural American Spirit brands), electronic cigarettes (Vuse brand), and moist snuff (Grizzly and Kodiak brands). Kanobe 2023: This research was funded by RAI Services Company, a wholly owned subsidiary of Reynolds American Inc., which is an indirect, wholly owned subsidiary of British American Tobacco plc.	
Author declarations	M.K., P.C., J.D., P.M., E.R., J.W.C., and E.S. are full‐time employees of RAI Services Company, and B.G.B., B.A.J., G.L.P., and P.N. are former full‐time employees of RAI Services Company. RAI Services Company and R.J. Reynolds Tobacco Company are wholly owned subsidiaries of Reynolds American Inc., which is a wholly owned subsidiary of British American Tobacco plc. G.L.P. is an independent consultant. B.N. is a full‐time employee of JTI Leaf Services (US) LLC. Kanobe 2023: M.N.K., P.M., P.C. and J.W.C. are full‐time employees of RAI Services Company, E.K.R. is a full‐time employee of BAT (Investments) Limited, and B.G.B., G.L.P. and P.R.N. are former full‐time employees of RAI Services Company. G.L.P. works as an independent scientific consultant. RAI Services Company is a wholly owned subsidiary of Reynolds American Inc., which is an indirect, wholly owned subsidiary of British American Tobacco plc	
Notes	Participants were compensated for their time and participation. For the purposes of our analyses, this study is treated as a single‐arm study and reported narratively.	
***Risk of bias***		
**Bias**	**Authors' judgement**	**Support for judgement**
Random sequence generation (selection bias)	Low risk	A computer software program was used by the statistician at the CRO to generate the randomization sequences.
Allocation concealment (selection bias)	Low risk	A computer software program was used by the statistician at the CRO to generate the randomization sequences.
Blinding of participants and personnel (performance bias) All outcomes	High risk	“The study was unblinded by necessity due to the very different visual appearances of the study products”
Blinding of outcome assessment (detection bias) All outcomes	Low risk	Relevant outcomes are biomarkers, which are objectively measured
Incomplete outcome data (attrition bias) All outcomes	Low risk	Vuse solo group 35/35; Vuse Vibe group 33/37; Vuse Ciro group 35/37.
Selective reporting (reporting bias)	High risk	The NCT record stated that some biomarkers would be measured at 5 days. Extra biomarkers have been reported at 7 days follow‐up.

**Table:** Kerr 2020

***Study characteristics***		
Methods	Design: RCT. Pragmatic, 2‐armed, single‐centre, randomized controlled pilot study Recruitment: community. Recruitment from NHSGGC Smokefree Services. GP surgeries. Secondary care QEUH, GRI, New Victoria Hospital and Glasgow Dental School. Advertized on NHSGGC staff payslips, local newspapers, community magazines, and on Gumtree. The British Heart Foundation, University of Glasgow and NHSGGC released a joint press release, which generated media coverage in 4 newspapers. Social media. Public engagement “pop up stands” were held collaboratively with smoke‐free services and the VAPOUR study team in the entrance foyer of the QEUH and at the Celtic Football Club Healthy Hoops Event. Setting: Scotland, UK Study start date: 1 December 2015. Study end date: 13 July 2018	
Participants	Total N: 55 EC arm 28. NRT arm 27 Inclusion criteria: Aged 18 to 65 yrs; habitual tobacco smokers (smoking on average 1 to 15 tobacco cigarettes pd > 6 months); willing to quit tobacco smoking; with either the use of nicotine replacement patches or an EC with nicotine‐containing e‐liquid, in addition to engaging with NHSGGC Community Smokefree Service's 12‐wk behavioural support programme. No established history of cardiovascular disease Exclusion criteria: Pregnant or breastfeeding; had used an EC or nicotine replacement patch in the last 3 months; were allergic to the active substances in either of the nicotine replacement products; history of illicit drug use, major depressive illness or other psychiatric conditions, peripheral arterial occlusive disease (PAOD), COPD, renal impairment (eGFR < 45 mL/min), uncontrolled hypertension (BP ≥ 165/95 mmHg), or CVD. Female 43.7%; mean age 44.2; mean CPD 15; mean FTND 7 E‐cigarette use at baseline: not reported Motivated to quit: participants were willing to quit	
Interventions	EC: **Refillable** EC arm E‐cigarette starter pack contained two commercially available second‐generation e‐cigarette devices with charging devices, 11 replacement atomisers and 12 x 10 bottles of nicotine containing e‐liquid, tobacco‐flavoured e‐liquid, 18 mg/mL nicotine. Each e‐cigarette device consisted of a 1300 mAh variable voltage rechargeable battery, a tank and an atomiser, and the charging device comprised of USB e‐cigarette charger and USB mains adaptor (SmokeMax; Groove Trading Ltd, 194 Glasgow UK), and written instructions. At baseline, oral and written information was given on how to operate the e‐cigarette, and for the duration of the study, participants were asked only to use the study e‐cigarette and e‐liquid they were provided with. NRT arm: NRT nicotine replacement patches, 12‐week reducing nicotine regimen (21 mg, 14 mg, 7 mg) of Nicotinell® Patches Weekly supply of nicotine replacement patches. If required, participants were also permitted to use additional other licensed nicotine replacement products (gum, lozenges, nasal spray, inhalers, and micro‐tabs), in combination with the nicotine replacement patches. Both arms: all participants received 12 weeks of behavioural support provided by NHSGGC Smokefree Community Services. Following the baseline visit, participants were asked to define their “quit date”.	
Outcomes	Baseline and 12 weeks CO confirmed smoking cessation Secondary outcomes: cardiovascular function (heart rate SBP, DBP), lung function, weight	
Study funding	Grant from British Heart Foundation (Centre of Research Excellence Award, reference number RE/13/5/30177)	
Author declarations	There were no conflicts of interest with the tobacco industry.	
Notes	New to 2022 update	
***Risk of bias***		
**Bias**	**Authors' judgement**	**Support for judgement**
Random sequence generation (selection bias)	Low risk	Quote: "Using a secure website (Sealed Envelope Ltd, 2016), participants were randomised in a 1:1 fashion, to either the e‐cigarettes combined with behavioural support or nicotine replacement patch group combined with behavioural support. Using a permuted block design with a computer random number generator, block randomisation with block sizes of 4, 6 and 8 was used to reduce bias and achieve balance in the allocation of participants to treatment arms".
Allocation concealment (selection bias)	Low risk	Quote: “Secure website (Sealed Envelope Ltd, 2016) participants randomised in a 1:1 fashion, ……Using a permuted block design with a computer random number generator, etc.”
Blinding of participants and personnel (performance bias) All outcomes	Low risk	Not blinded, but as both arms contained active interventions, performance bias judged unlikely
Blinding of outcome assessment (detection bias) All outcomes	Low risk	BP, heart rate, and oxygen saturation measured. Abstinence at 12 weeks, self‐report was CO validated.
Incomplete outcome data (attrition bias) All outcomes	Low risk	31/55 (56.3%) EC arm 18/28; NRT arm 14/27
Selective reporting (reporting bias)	Low risk	Reported outcomes ‘per protocol’

**Table:** Kimber 2021

***Study characteristics***		
Methods	Design: RCT Recruitment: participants were screened via phone interviews Setting: East London, UK Study start date: participants recruited between December 2015 and December 2016	
Participants	Total: N = 50 people who smoked combustible cigarettes and were e‐cigarette naive. Initial sample (N = 70) attended first session; all analyses were conducted on the N = 50 who returned for their 2nd and 3rd session. Cig‐a‐likes: N = 11 Tank18: N = 20 Tank6: N = 19 Inclusion criteria: Smoke daily ≥ 5 cigarettes, have smoked for ≥ 1 year, not currently using an EC, willing to abstain 1 hr before the start of the session and willing to make a quit attempt Exclusion criteria: < 18 years, not fluent in English, pregnancy or breastfeeding, or a known neurobiological or heart condition 64% women; mean age 29.5 (SD 9.31); mean CPD 13.09 (SD 6.66), mean FTND 4.14 (SD 2.45) Motivated to quit: "Willing to make a quit attempt" E‐cigarette use at baseline: no	
Interventions	EC: **cartridge and refillable** Arm 1: cig‐a‐like (18 mg/mL) The ‘Blu’ (n = 13) and ‘TECC Go e‐cigarette’ models (n = 11) were used for the Cig‐a‐like condition, due to issues of leakages with the latter. Non‐adjustable battery power output and could be recharged, a supply of spare disposable cartridges were provided. Arm 2: a tank model containing 18 mg/mL (Tank18) Arm 3: a tank model containing 6 mg/mL (Tank6) For both conditions, Tank18 and Tank6 the ‘*Totally Wicked mini curve’* was mounted with a 2 mL capacity tank which housed a standard atomiser of 1.5 ohm resistance. E‐liquid ingredients composition and flavours were kept consistent across all conditions using the same ratio of propylene glycol and vegetable glycerin (PG/VG: 50/50) and tobacco flavour. Intervention. All 3 group participants vaped 20 min ad libitum in 3 separate sessions (baseline, 1 and 2 weeks post‐baseline). Ahead of their baseline session, participants were instructed to abstain from smoking for an hour. Rated their craving and withdrawal symptoms (at the beginning and end of the session), before receiving instructions on how to use their EC and to vape ad libitum for 20 mins Positive and adverse effects were measured at the end of the last puff. All vaping sessions were video‐recorded. At the end of each session, participants were given the EC and were instructed to keep a record of the number of cigarettes smoked at the end of each day until their next and subsequent sessions. Each participant was provided with a weekly supply of either, 60–80 mL of e‐liquid in refill bottles for those in the tank conditions, or 15 cartridges for those in the cig‐a‐like condition at the end of each testing session. The session was repeated the following week, then one week later. Participants were asked to keep the device and encouraged to try and replace as many tobacco cigarettes as they could with the use of their EC.	
Outcomes	Baseline, week 1, week 2 CO was measured at baseline, wk 1 and wk 2. Self‐reported CPD. Adverse events. Puff duration, puff number, inter‐puff intervals (IPI). Cigarette dependence, craving, withdrawal, and subjective effects	
Study funding	This work was funded by the University of East London through a PhD studentship award. The funder had no role in the study design, collection, analysis or interpretation of data, writing the manuscript and the decision to submit the manuscript for publication.	
Author declarations	CK and KS have no conflicts of interest to declare. LD has provided consultancy for the pharmaceutical industry relating to the development of smoking cessation products.	
Notes	New to 2022 update	
***Risk of bias***		
**Bias**	**Authors' judgement**	**Support for judgement**
Random sequence generation (selection bias)	Low risk	Participants were randomly allocated (using SPSS).
Allocation concealment (selection bias)	Unclear risk	No detail
Blinding of participants and personnel (performance bias) All outcomes	Low risk	Intervention arms received equally intensive interventions.
Blinding of outcome assessment (detection bias) All outcomes	Low risk	CO measured
Incomplete outcome data (attrition bias) All outcomes	High risk	There was a > 20% difference in FU between arms at 2 weeks. Overall, 50/70 = 71.43%. Cig‐a‐like 11/24 = 45%. Tank6 19/23 = 82.61%. Tank18 20/23 = 86.96%
Selective reporting (reporting bias)	High risk	Reported on AEs but excluded those who had reported AEs (as they did not complete all sessions (due to AEs)

**Table:** Klonizakis 2022

***Study characteristics***		
Methods	Design: RCT. Pragmatic, 3‐group, randomized, assessor‐blinded, single‐centre trial Setting: Centre for Sport and Exercise Science (CSES) of Sheffield Hallam University, UK Recruitment: from the community in the wider Sheffield area via: i) low‐cost newspaper and post‐office advertisement, ii) posters in local pharmacies, libraries, mosques, churches, and clubs, iii) social media or search engine advertisement (Facebook, Google ads) iv) notices in newsletters or participation in outreach events of community organizations (such as Sheffield U3A and AGE UK), iv) a study website, and v) out‐reach events in local ethnic community centres or places of worship Study start April 2017. Study end December 2020	
Participants	Actual enrolment: 248 participants Nicotine EC arm = 84 Non‐nicotine arm = EC 82 NRT arm = 82 % female: nicotine EC = 55%; non‐nicotine EC = 50%; NRT = 44% Mean age 44 Mean CPD: nicotine EC = 18 (SD7); non‐nicotine EC = 16 (SD7); NRT = 18 (SD7) Mean FTND: 6 (SD2) Inclusion criteria: Age > 18 years of either sex People who smoke (at least 10 cpd for the past year) Willing (by declaration) to attempt to quit smoking by using the NHS services or e‐cigarettes Exclusion criteria: Inability to walk Recent (within 6 months) cardiovascular disease event (e.g. stroke, myocardial infarction) or cardiac surgery Insulin‐controlled diabetes mellitus or with co‐existing skin conditions, leg ulcers, vasculitis or deep venous occlusion (as these may affect their cardiovascular function) Pregnancy Requiring major surgery during the course of the study Contra‐indications/unsuitability for NRT Current daily use of e‐cigarettes Currently undertaking a cessation attempt supported by a smoking cessation clinic Motivated to quit: yes	
Interventions	a) Complimentary **e‐cigarette equipment** and refills (Tornado V5, Joyetech, Shenzhen, China, Totally Wicked, Blackburn, UK) at allocation stage, together with instructions on the correct usage of e‐cigarettes. They will also receive behavioural support for a 3‐month period. The nicotine strength of Group A cartridges will be up to 18 mg/mL nicotine strength. Participants, on average, received 20 bottles of 10 mL during the intervention period. Participants could choose ice menthol or tobacco flavour. b) As a), but with **nicotine‐free liquid** c) Referral to **NHS smoking cessation clinics** and will receive NRT in conjunction with behavioural support. Received money or shopping vouchers (depending on personal preference) as reimbursement for NRT prescription charges All groups received the same level and type of behavioural support as currently offered as standard by stop‐smoking services, in the form of regular face‐to‐face or telephone appointments as per relevant guidelines, e.g. minimum of 6 support sessions within the 3‐month period.	
Outcomes	Follow‐up: within 3 days of “quit date”, 3 and 6 months past quit date Outcome measures: Macrovascular function (FMD assessment) Microvascular function Smoking status at 3 and 6 months, self‐reported and biochemically validated by exhaled air measurement of < 10 ppm CO Change in CVD risk using Q‐risk assessment Health economic effects using EQ‐5D‐L Total cholesterol and high density lipoprotein via fingerprick blood sample Participant experiences' assessment	
Study funding	The trial was funded by Heart Research UK under a Translational Research Grant (RG2658). The funder had no role in the study’s design, conduct, and reporting.	
Author declarations	The authors declare that they have no competing interests.	
Notes	New to 2023 (previously ongoing)	
***Risk of bias***		
**Bias**	**Authors' judgement**	**Support for judgement**
Random sequence generation (selection bias)	Low risk	“Randomized remotely into three groups by an independent statistician using a computer‐generated (nQuery Advisor 6.0, Statistical Solutions, Ireland) block randomization stratifed by gender and “pack‐years” (number of packs (20 cigarettes per pack) per day times number of years smoked) Unique trial number for each participant for the study duration”
Allocation concealment (selection bias)	Low risk	“Outcome assessors were blinded to group allocation and participants were reminded regularly not to share their group allocation with assessors or those providing behavioural support. The study statistician/health economist was blinded to group allocation.”
Blinding of participants and personnel (performance bias) All outcomes	Low risk	Interventions equally intensive
Blinding of outcome assessment (detection bias) All outcomes	Low risk	Outcome assessors were blinded to group allocation and participants were reminded regularly not to share their group allocation with assessors or those providing behavioural support. The study statistician/health economist was blinded to group allocation. Those delivering the intervention were only blinded in relation to which e‐cigarette group the participants belonged to as the NRT group was receiving support through the stop‐smoking service. All measures biochemically validated or measured
Incomplete outcome data (attrition bias) All outcomes	Low risk	Nic EC 66/84 at 6 months Non‐Nic EC 64/82 NRT 72/82
Selective reporting (reporting bias)	Unclear risk	Not all secondary outcomes reported in this paper, but more publications likely to follow

**Table:** Kumral 2016

***Study characteristics***		
Methods	Design: prospective randomized clinical trial Recruitment: all patients admitted to a smoking cessation clinic at the Department of Otorhinolaryngology‐Head and Neck Surgery, Okmeydanı Training and Research hospital Setting: smoking cessation clinic, Turkey Study start date: March 2013; Study end date: November 2013	
Participants	Total N: 98 but analysis excluded 16 from intervention and 10 from control who did not stop smoking; thus 72 analyzed N per arm: EC: 58 (42 analyzed); non‐EC 40 (30 analyzed) Inclusion criteria: Smoked at least 1 pack of cigarettes a day for at least 5 years Exclusion criteria: History of allergic rhinitis, chronic sinusitis, vasomotor rhinitis, asthma, malignancy, or surgery in upper respiratory tract Age under 18 years Use of psychoactive drugs Inclusion based on specific population characteristics: no 44% women; mean age 36; mean cpd and mean FTND not specified Motivated to quit: “All patients were willing to quit smoking”. E‐cigarette use at baseline: not specified	
Interventions	**EC: Unclear** **EC arm**: “used EC to quit smoking” – allowed to select brand and flavour, used “medium density” liquid (11 to 12 mg/mL) (no further detail given) **Non‐EC arm**: received cognitive behavioural therapy (no further detail given)	
Outcomes	3 months Sinonasal outcome test (SNOT‐22) via self‐administered questionnaire, to evaluate changes in subjective symptoms. Saccharin transit test to evaluate nasal mucociliary clearance (MCC) function, which authors stated is “an important defence mechanism”	
Study funding	Not specified	
Author declarations	Not specified	
Notes		
***Risk of bias***		
**Bias**	**Authors' judgement**	**Support for judgement**
Random sequence generation (selection bias)	Unclear risk	Quote: “Patients participating in the study were randomly divided into two groups; EC smokers (group 1) and non‐EC smokers (group 2).” No further detail provided
Allocation concealment (selection bias)	Unclear risk	No information given
Blinding of participants and personnel (performance bias) All outcomes	High risk	Participants were not blinded. The trial is described as single‐blinded and outcome assessors were blinded. No placebo used
Blinding of outcome assessment (detection bias) All outcomes	High risk	Self‐reported outcome data, participants not blinded and unequal amounts of support between arms
Incomplete outcome data (attrition bias) All outcomes	Unclear risk	Dropout rate not clear. Only analyzed people who quit
Selective reporting (reporting bias)	Low risk	All expected outcomes reported

**Table:** Lee 2018

***Study characteristics***		
Methods	Randomized, parallel‐assignment, double‐blind pilot trial Setting: San Francisco Veterans Affairs Medical Center (SFVAMC), USA Recruitment: veterans awaiting surgery Recruitment: from VA hospital presenting for surgery Study start date: August 2015; study end date: May 2016	
Participants	Total N: 30 N per arm: NRT: 10; END: 20 Inclusion criteria: presented to the anaesthesia preoperative clinic for elective surgery 3 or more days before surgery; currently smoked ≥ 2 CPD, having smoked at least once in the last 7 days Exclusion criteria: exclusively used other forms of tobacco or marijuana only; pregnancy/breastfeeding; unstable cardiac condition; currently using smoking cessation pharmacotherapy; already enrolled in a smoking cessation trial; using EC on a daily basis Inclusion based on specific population characteristics: patients awaiting elective surgery 10% women; mean age 54; mean cpd 14; mean FTND 3.3 Motivated to quit: not specified E‐cigarette use at baseline: not specified but excluded daily users	
Interventions	**EC: Cig‐a‐like** Both groups received: i) referral to the California Smokers' Helpline, ii) brief advice lasting less than 2 minutes, iii) a brochure from the ASA about quitting smoking before surgery. **EC arm**: 6‐week supply of NJOY e‐cigarettes (disposable, first generation). Instructed to use Bold (4.5%) ad lib for 3 weeks, then Gold (2.4%) ad lib for 2 weeks and then study (0%) ad lib for final week. Number of ECs issued corresponded to baseline cpd, assuming 1 EC = 10 cigarettes. Asked to refrain from the use of all study products at the end of 6 weeks **NRT arm**: 5‐week Nicoderm CQ patches, 1 week placebo patches. Dose based on cpd at baseline: ≥ 10 cpd, 21 mg/day for 3 weeks, 14 mg/day for 1 week, 7 mg/day for 1 week, 0 mg/day for 1 week. < 10 cpd at baseline: 14 mg/day for 3 weeks, 7 mg/day for 2 weeks, 0 mg/day for 1 week	
Outcomes	30 days (phone), 8 weeks (in person), 6 months (phone) Cessation: 7‐day PP at 30 days (not validated), 8 weeks (CO‐validated), 6 months (not validated). Smoking cessation for at least 48 hours on day of surgery (CO‐validated) Adverse events and biomarkers: Adverse events, side effects, and surgical complications by self‐report at 30 days, 8 weeks At 8 weeks exhaled CO, FEV1, and FVC Other outcomes measured: Attitudes and usage Salivary cotinine Smoking reduction	
Study funding	“This work was funded by internal UCSF Department of Anesthesia and Perioperative Care funds (San Francisco, California, United States of America) and the UCSF Resource Allocation Program grant, administered by the Helen Diller Family Comprehensive Cancer Center developmental funds from the National Cancer Institute Cancer Center Support Grant (P30 CA 82103‐16). E‐cigarettes were purchased from NJOY using these funds. NJOY had no involvement in the design, execution, or analysis of the study. The funders had no role in study design, data collection and analysis, decision to publish, or preparation of the manuscript.”	
Author declarations	“The authors declare there are no competing interests”.	
Notes	3 NRT participants used EC, 2 EC participants used nicotine patch Study listed as ongoing study NCT02482233 in the 2016 review update	
***Risk of bias***		
**Bias**	**Authors' judgement**	**Support for judgement**
Random sequence generation (selection bias)	Low risk	Quote: “Randomization was computer‐generated, with randomly permuted block sizes of 3 or 6, in a 2:1 ratio using the ralloc program”.
Allocation concealment (selection bias)	Low risk	Quote: “Allocation was concealed by consecutively numbered, sealed, opaque envelopes”.
Blinding of participants and personnel (performance bias) All outcomes	Low risk	Not blinded but both interventions active with equal amounts of support so performance bias judged unlikely
Blinding of outcome assessment (detection bias) All outcomes	Low risk	Self‐report only at 6 months and participants not blinded to condition, but similar level of support given to both groups so differential misreport judged unlikely
Incomplete outcome data (attrition bias) All outcomes	Low risk	1 NRT and 1 ENDs loss to follow‐up at 6 months
Selective reporting (reporting bias)	Low risk	All expected outcomes reported

**Table:** Lee 2019

***Study characteristics***		
Methods	Design: randomized controlled trial Recruitment: recruited from motor company Setting: motor company, medical office in Korea Study start date: 5 January 2012; study end date: 31 August 2012	
Participants	Total N: 150 N per arm: EC: 75; NRT: 75 Inclusion criteria: Male At least 10 cpd in previous year Smoked for at least 3 years Motivate to stop smoking entirely or reduce consumption Exclusion criteria: Past history of serious clinical disease Attempted to stop smoking in past 12 months by using NRTs Inclusion based on specific population characteristics: no 0% women; mean age 42.3; mean cpd: not reported, 1.01 packs per day; mean FTND 4.05 Motivated to quit: yes, or to reduce E‐cigarette use at baseline: not specified	
Interventions	**EC: Refillable** Both arms received 50 mins of education sessions on smoking cessation and use of smoking cessation aids in the medical office (no further detail given). Asked to return to the medical office every 4 weeks (to 24 weeks?) for “evaluation and counselling by an independent health practitioner” **Arm 1:** 50‐min education sessions on smoking cessation and the use of smoking‐cessation aids, instructed to visit the medical office each month for evaluation and counselling by a health practitioner who was unaffiliated with the study. Participants supplied with eGo‐CTM EC (nicotine 0.01 mg/mL) from Ovale in 12‐wk supply **Arm 2:** As (1) but instead of EC given 2 mg nicotine gum in 12‐wk supply	
Outcomes	12, 24 weeks (in person) Cessation: continuous abstinence from 9 to 24 weeks, exhaled CO < 10 ppm, negative urine cotinine Adverse events and biomarkers: yes, but just note ‘adverse events’ Other outcomes measured: 7‐day PPA, cigarette reduction	
Study funding	“none”	
Author declarations	“none declared”	
Notes	Study listed as ongoing study KCT0001277 in the 2016 review update	
***Risk of bias***		
**Bias**	**Authors' judgement**	**Support for judgement**
Random sequence generation (selection bias)	Low risk	Quote: “computer‐generated randomization sequence with a block size of 2”
Allocation concealment (selection bias)	Low risk	Quote: “The enrolment and assignment of all subjects were performed by a clinical research coordinator not involved in the study”.
Blinding of participants and personnel (performance bias) All outcomes	Low risk	Not blinded but both interventions active with equal amounts of support, so performance bias judged unlikely
Blinding of outcome assessment (detection bias) All outcomes	Low risk	Participants not blinded but results biochemically validated
Incomplete outcome data (attrition bias) All outcomes	Low risk	61/75 NRT and 71/75 EC FU at 24 weeks
Selective reporting (reporting bias)	Low risk	All prespecified outcomes reported

**Table:** Lucchiari 2022

***Study characteristics***		
Methods	Design: randomized, parallel‐assignment, double‐blind trial Recruitment: participants enrolled in lung cancer screening programme Setting: early lung cancer detection programme (Cosmos II) at European Institute of Oncology, Italy Study start date: September 2014; study end date: January 2016	
Participants	Total N: 210 N per arm: 70 participants per arm At 12 months: nicotine EC 60; non‐nicotine EC 58; support only 60 Inclusion criteria: Participants are involved in the COSMOS II study Participants are 55 years or more and have smoked at least 10 cigarettes a day for the past 10 years Participants wish to reduce tobacco smoking (motivational score higher than 10) who are not treated at a smoking centre Signed informed consent Exclusion criteria: Symptomatic cardiovascular disease Symptomatic severe respiratory disease Regular psychotropic medication use Current or past history of alcohol abuse Use of smokeless tobacco or NRT Participation in another anti‐smoking programme in the current year Inclusion based on specific population characteristics: 55 years of age or older 37% women; mean age 62.8; mean cpd 19.38; mean FTND 4.37 Motivated to quit: yes E‐cigarette use at baseline: excluded people who smoke who had ever regularly used e‐cigarettes for more than 1 week alone or in combination with tobacco cigarettes	
Interventions	**EC: Cig‐a‐like** Both arms received “low intensity counselling” – phone at week 1, 4, 8, and 12, approx. 10 mins each **Nicotine EC arm**: e‐cigarette kit and 12 x 10 mL liquid cartridges (8 mg/mL nicotine concentration). During the first week, participants could use the e‐cigarette ad libitum. At the end of the first week, asked to use only EC for the next 11 weeks. **Nicotine‐free EC (placebo) arm**: nicotine‐free EC – same as above but with nicotine‐free EC	
Outcomes	Months 3, 6, and 12 Cessation: continuous abstinence for previous month, CO ≤ 7 ppm Adverse events and biomarkers: FOR EC ARMS ONLY: Exhaled CO Leicester Cough Questionnaire (LCQ) Respiratory symptoms (self‐report) Side effects using checklist Other outcomes measured: Motivational questionnaire HADS EC use	
Study funding	This study was supported by a grant from Fondazione Umberto Veronesi (FUV).	
Author declarations	The authors declared no conflicts of interest.	
Notes	Listed as ongoing study Lucchiari 2016 (NCT02422914) in 2016 review; new for 2020 update, relabelled from Lucchiari 2020 to Lucchiari 2022 at 2023 update.	
***Risk of bias***		
**Bias**	**Authors' judgement**	**Support for judgement**
Random sequence generation (selection bias)	Low risk	Quote: “A randomization list using a permuted block design (40 blocks of 6 subjects randomly assigned to 1 of the 3 treatment arms) had been previously prepared by independent personnel.”
Allocation concealment (selection bias)	Low risk	Double‐blind, active and placebo e‐cigarettes labelled by independent personnel; researcher and participants blind
Blinding of participants and personnel (performance bias) All outcomes	Low risk	“double blind” for nicotine vs no nicotine EC but limited info given; however, as similar levels of support across arms performance bias judged unlikely
Blinding of outcome assessment (detection bias) All outcomes	Low risk	Biochemical validation used
Incomplete outcome data (attrition bias) All outcomes	Low risk	Approx. 73% followed up in each group at 6 months, very little difference between groups At 12 months > 70% FU
Selective reporting (reporting bias)	High risk	Papers stated CO collected but data not presented

**Table:** Martinez 2021

***Study characteristics***		
Methods	Design: RCT Recruitment: participants were recruited throughout the USA through Facebook and multimedia advertisements (newspapers, radio, TV, e‐cigarette forums, and so on) for a study measuring attitudes and behaviours about cigarettes and e‐cigarettes. Setting: USA Study start date: 31 March 2015. Study end date 30 June 2019	
Participants	2896 dual users of nicotine EC and combustible tobacco cigarettes Assessment only n = 575; generic smoking cessation booklets n = 1154; targeted booklets n = 1167 37% female; mean age 29.9 Mean cpd: 1 to 10, 1663 (57%); 11 to 20, 972 (34%); > 20, 259 (9%). Mean FTND 3.6. E‐cigarette use at baseline Inclusion criteria: age 18 years or older, smoked 1 or more combustible cigarettes per week over the preceding year, used e‐cigarettes 1 or more times per week over the preceding month, not currently enrolled in a face‐to‐face smoking cessation programme, and able to speak and read English. The original inclusion criteria required daily smoking. However, early in the trial, it became apparent that many dual users were skipping smoking on some days. Therefore, to better reflect the dual‐using population, we amended the use frequency criteria to equate them for smoking and vaping at 1 or more uses per week. The protocol was amended on 25 September 2016. We had recruited 652 participants up to that date. Participants were not necessarily seeking treatment or motivated to quit smoking or vaping. Participation was limited to 1 individual per street address. Participants gave oral informed consent.	
Interventions	EC type: n/a Assessment only (n = 575) Generic smoking cessation self‐help booklets previously shown to be efficacious in smokers (n = 1154) (an introductory *Stop smoking for good* brochure, 10 x *Stop smoking for good* didactic booklets, and 9 *How I quit smoking* pamphlets) Booklets specifically targetting dual users (n = 1167 (*If you vape: a guide to quitting smoking*), which included an introductory *If you vape* brochure, a series of 10 x *If you vape: guide to quitting smoking* booklets, and 9 x *My story* pamphlets) Participants assigned to the GENERIC or eTARGET groups were sent the intervention materials by post, with the option of also receiving them electronically.	
Outcomes	7‐day PPA at each assessment point. Sustained abstinence: 30‐day and 90‐day PPA Breath CO and saliva samples (for cotinine analysis) were collected at the 12‐ and 24‐month follow‐up points for participants who reported abstinence and resided within 100 miles of the home institution. Cut‐offs of 8 ppm for CO and 10 ng/mL for cotinine were used to determine abstinence. The disconfirmation rates from this sample were used to estimate adjusted smoking rates for the full sample. Full follow‐up assessments: 6, 12, 18, and 24 months. Abbreviated assessments: 3, 9, 15, and 21 months after baseline	
Study funding	This work was supported by the National Institute on Drug Abuse of the NIH (R01DA037961). The content is solely the responsibility of the authors and does not necessarily represent the official views of the NIH. This work has also been supported in part by the Biostatistics and Bioinformatics Shared Resource and the Participant Research, Interventions, and Measures Resource at the H Lee Moffitt Cancer Center and Research Institute, a National Cancer Institute designated Comprehensive Cancer Center (P30CA76292)	
Author declarations	Quote: "THB has received research support from the US National Institutes of Health (NIH), the American Cancer Society, the Florida Department of Health, and Pfizer; has collaborated on funded research with Voxiva, Optum, and the University of East Anglia (Norwich, UK); spent sabbatical at the Trimbos Institute and Utrecht University (Utrecht, Netherlands); is on the advisory board of, and holds restricted stock in, Hava Health, which is developing a pharmaceutical grade electronic nicotine delivery system for smoking cessation; participated in a Best Brains Exchange for Health Canada, providing advice on e‐cigarette policy; and consulted for the Australian Government Solicitor regarding plain tobacco packaging. UM has received research support from the NIH and the Galician Plan of Research, Innovation, and Growth (Spain); and has received funding from the Barrie Foundation to receive predoctoral training at the University of Newcastle (Callaghan, NSW, Australia). VNS has received research support from the NIH and the Florida Department of Health. SKS has received research support from the NIH, the American Cancer Society, the Florida Department of Health, and Pfizer. DJD has received research support from the NIH, the American Cancer Society, and the Florida Department of Health, and has provided paid expert testimony in litigation against tobacco companies. MMB has received funding from the NIH, the Florida Department of Health, the US Department of Veterans Affairs, the US Centers for Disease Control and Prevention, the National Science Foundation, and the US Department of Housing & Urban Development; and has received research support from Gilead Sciences, Florida Blue Foundation, Bristol Myers Squibb Foundation, Merck Foundation, Maine Cancer Foundation, and Pfizer. PTH has received research support from the NIH, US Food and Drug Administration (FDA), and Virginia Foundation for Healthy Youth. TE conducts research supported by the National Institute on Drug Abuse of the NIH and the Center for Tobacco Products of the FDA; is a paid consultant in litigation against the tobacco industry and the electronic cigarette industry; is named on one patent for a device that measures the puffing behaviour of electronic cigarette users and on another patent for a smartphone app that determines electronic cigarette device and liquid characteristics; owns shares in a variety of mutual funds, the exact stock makeup of which he has no control, and owns shares in three publicly traded companies, none of which are in any way related to the tobacco industry, the electronic cigarette industry, or any other aspect of this work; and has served as a special government employee of the US Government in the context of his service on the FDA’s Tobacco Products Scientific Advisory Committee and the Department of Health and Human Services Secretary’s Advisory Committee on Human Research Protection. CRB has received research support from the New Zealand Ministry of Health, the Health Research Council of New Zealand, CureKids Foundation, Heart Foundation, Health Promotion Agency, and Auckland Council and Sanitarium; collaborates on funded research with Newcastle University (Australia) through a grant from the Australian National Health and Medical Research Council, with Zhejiang University (Hangzhou, China) and Kunming University (Yunnan, China) on an Education New Zealand Tripartite grant, and with the University of Malaya (Kuala Lumpur, Malaysia) on a University of Malaya Grand Challenges grant; received funding from Pfizer Australasia for a survey of the impact of COVID‐19 on health workers in low‐income and middle‐income countries and from Johnson & Johnson Japan for consultancy on smoking cessation medication; and was a consultant to Moffit Cancer Center on this study through an NIH grant. LRM and KOB declare no competing interests. The employees of Moffitt Cancer Center—UM, VNS, SKS, DJD, LRM, KOB, MMB, and THB—are eligible for sharing of any revenue that might be generated by products developed during their employment, including the intervention used in this study."	
Notes	Quote: "Participants were compensated US$10–20 for the first eight assessments and $40 for the final one, and they were eligible for $40–60 bonuses for completing at least seven assessments. Participants returning assessments within 1 week were sent inexpensive appreciation gifts." Appendix: "Participants were not aware in advance of the interview that they would be asked for biosamples, and new informed consent was obtained at that time. Participants received $20 for completing a biochemical verification interview and $15 for providing biosamples."	
***Risk of bias***		
**Bias**	**Authors' judgement**	**Support for judgement**
Random sequence generation (selection bias)	Low risk	Quote: "We used computer generated randomisation with balanced permuted blocks (block size 10, with 2‐4‐4 ratio) to allocate participants to assessment only (ASSESS group), generic smoking cessation self‐help booklets (GENERIC group), or booklets targeting dual users (eTARGET group)."
Allocation concealment (selection bias)	Low risk	Computer‐generated (see above)
Blinding of participants and personnel (performance bias) All outcomes	Low risk	Tailored versus generic booklet judged low risk as similar intensity; this is the comparison used in the meta‐analysis. Tailored versus no support would be high risk due to differential levels of support provided and no blinding.
Blinding of outcome assessment (detection bias) All outcomes	Low risk	As above, tailored versus generic similar intensity so judged to be low risk of differential misreport (self‐reported cessation only)
Incomplete outcome data (attrition bias) All outcomes	Low risk	All > 50% at 24 months ASSESS: 361/575*100 = 62.8% GENERIC: 619/1154*100 = 53.6% eTARGET: 642/1167*100 = 55%
Selective reporting (reporting bias)	Low risk	All prespecified outcomes reported in the trial register reported in the publication

**Table:** Martner 2019

***Study characteristics***		
Methods	Design: a non‐concurrent multiple baseline across participants design. Three phases were included: baseline, EC, and EC + CM. Half the participants received the EC phase following baseline; the other half received EC + CM following baseline. Recruitment: community Setting: set‐up meetings occurred at the University of Florida Behavioral Health and Technology Research Clinic, USA Study start date/study end date: not specified	
Participants	Total N: 12 Inclusion criteria: 18 to 65 years old; smoked ≥ 2 years; smoked ≥ 8 cpd on average; smoked in the past 24 hours; expressed a desire to quit smoking (yes/no); reliable access to the internet and a computer or smartphone; breath CO ≥ 10 ppm at set‐up Exclusion criteria: current or previous medical condition that would pose an increased risk to participation; use of benzodiazepines, cocaine, or opiates in the previous 6 months; smoking marijuana more than twice a month; exposed to elevated CO levels (e.g. spouse smokes in house); pregnant or expected to become pregnant in the next 6 months 58.3% women; mean age 37.5; mean cpd 16.25; mean FTND 5 Motivated to quit: expressed a desire to quit smoking E‐cigarette use at baseline: 3 participants never tried an EC prior to the study; 2 owned an EC but quit using it more than a month prior to the study; the remaining 7 had tried an EC more than a year prior to the study but never owned one	
Interventions	**EC: Refillable** All participants provided with smokio electronic cigarettes (second‐generation ECs) and V2 e‐liquid with a concentration of 24 mg/mL (2.4%) of nicotine. Researchers provided participants with a copy of the National Cancer Institute’s brochure *Clearing the Air* (smokefree. gov [http://smokefree. gov]). Then researchers and participants read through a manual that described the study procedures, and showed participants how to use the software to measure CO and how to use the EC. Participants initially received EC without contingency for a period of 14 days following the quit attempt. If participants failed to reduce CO levels during this phase, they received contingency management in addition to EC.	
Outcomes	4 weeks Adverse events and biomarkers: adverse events collected in 4‐day smoking behaviour questionnaires; eCO Other outcomes measured: acceptability and use of EC; overall experience of study	
Study funding	"The study was supported in part by crowd‐sourced funding enabled by Experiment.com. Preparation of this paper was supported in part by Grant P30DA029926."	
Author declarations	"The authors declare no conflicts of interest."	
Notes	N of 1 (within‐participants randomized design, not between groups). New for 2020 update	
***Risk of bias***		
**Bias**	**Authors' judgement**	**Support for judgement**
Random sequence generation (selection bias)	High risk	Not randomized
Allocation concealment (selection bias)	High risk	Not randomized
Incomplete outcome data (attrition bias) All outcomes	Unclear risk	No details provided.
Selective reporting (reporting bias)	Unclear risk	AEs measured in behavioural change questionnaire but not reported

**Table:** McRobbie 2015

***Study characteristics***		
Methods	Design: prospective cohort Recruitment: advertisements in free London newspapers Setting: smokers' clinic, East London, UK Study start date: February 2013; study end date: September 2013	
Participants	Total N: 40 Inclusion criteria: People who smoke daily who want to quit Aged 18 and older Exclusion criteria: Pregnant and breastfeeding women Current serious medical illness EC use for more than 1 week in the past 45% women, mean age 47 (SD 12), mean cpd 19 (SD 10), mean FTND 5.2 (SD 2.8), 65% in full‐time employment Motivated to quit: yes E‐cigarette use at baseline: excluded those who had used EC for more than 1 week in the past	
Interventions	**EC: Cig‐a‐like** Participants attended a baseline session 1 week prior to their TQD. On the TQD, participants were provided with an EC (Green Smoke, 1st generation device, 2.4% nicotine cartridges). 2 cartridges a day were supplied initially, with the supply adjusted to actual use later. Attended 4 weekly follow‐up sessions and received standard behavioural support.	
Outcomes	Cigarette consumption and CO readings collected at each session. Urine sample for cotinine and 3‐HPMA analysis collected at baseline and 4 weeks post‐TQD. Change in urinary 3‐HPMA (ng/mg creatinine) at 4 weeks Change in urinary cotinine (ng/mg creatinine) at 4 weeks Change in CO at 4 weeks	
Study funding	"This study was funded by a grant given to P. Hajek, H. McRobbie, and M.L.Goniewicz from the UK Medicines and Healthcare Products Regulatory Agency (MHRA). The costs of publication of this article were defrayed in part by the payment of page charges. This article must therefore be hereby marked advertisement in accordance with 18 U.S.C. Section 1734 solely to indicate this fact."	
Author declarations	"H. McRobbie is Clinical Director at The Dragon Institute; reports receiving commercial research grant from Pfizer; and has received speakers bureau honoraria from Johnson&Johnson and Pfizer. M.L. Goniewicz reports receiving a commercial research grant from Pfizer. P. Hajek has received speakers bureau honoraria from and is a consultant/advisory board member for the manufacturers of stop‐smoking medications. No potential conflicts of interest were disclosed by the other authors."	
Notes		
***Risk of bias***		
**Bias**	**Authors' judgement**	**Support for judgement**
Random sequence generation (selection bias)	High risk	Prospective cohort
Allocation concealment (selection bias)	High risk	Not randomized
Incomplete outcome data (attrition bias) All outcomes	Low risk	7/40 participants were lost to follow‐up.
Selective reporting (reporting bias)	Low risk	All predefined outcomes reported

**Table:** Meier 2017

***Study characteristics***		
Methods	Design: randomized, cross‐over trial (e‐cig vs placebo) Recruitment: via local media outlets Setting: community, USA Study start date/study end date: not specified	
Participants	Total N: 24 Inclusion criteria: ≥ 18+ years People who smoke daily (≥ 10 cpd) Not interested in quitting in the next 30 days English‐speaking Interested in using EC Exclusion criteria: Using cessation meds Use of ECs in last 6 m Exhaled CO < 6 ppm History of CV trauma or uncontrolled hypertension Pregnant Inclusion based on specific population characteristics: no 25% women; mean age 48.5; mean cpd 16.3; FTND not reported Motivated to quit: no (eligibility criteria was to not want to quit in next 30 days) E‐cigarette use at baseline: 8/24 (33%) had previously tried an EC, average 9.4 months since last use, average length of use 3.6 days	
Interventions	**EC: Cig‐a‐like** Smoked “as usual” for 1 week followed by 2 weeks of either placebo or active 1st generation EC BluCig starter kit with up to 7 cartridges (prefilled, with either active 16 mg or 0 mg nicotine solution) Participants were instructed “this e‐cig may or may not contain nicotine; we ask that you try it at least once, but use it however you like; smoke regular cigarettes as you wish.” Shown how to charge the device and sampled the product during the visit. Provided a handout on how to use the product (e.g. switching cartridges) and general information about ECs	
Outcomes	1 week in each condition, in person Adverse events and biomarkers: Adverse events, not clear how collected Exhaled CO Other outcomes measured: Vaping Regular smoking Perceived reward from ECs Intentions/confidence to quit Cotinine Withdrawal symptoms	
Study funding	“…supported by grants P01 CA138389, P30 CA138313 (Hollings Cancer Center Support Grant) from the National Cancer Institute of the National Institutes of Health and UL1 TR000062 from the National Center for Advancing Translational Science of the National Institutes of Health. BWH was supported by K12DA031794”.	
Author declarations	“KMC has received grant funding from Pfizer, Inc., to study the impact of a hospital‐based tobacco cessation intervention. He also receives funding as an expert witness in litigation filed against the tobacco industry. We have no other declarations of interests to declare”.	
Notes	New for 2020 update	
***Risk of bias***		
**Bias**	**Authors' judgement**	**Support for judgement**
Random sequence generation (selection bias)	Unclear risk	Quote: “Participants were randomized to receive either an active or placebo EC first”; no further information provided
Allocation concealment (selection bias)	Unclear risk	Refer to 'random sequence generation'.
Blinding of participants and personnel (performance bias) All outcomes	Low risk	Quote: “Participants and research staff conducting sessions were blinded to dose. All cartridges were pre‐loaded by the manufacturer. Labeling was removed by a research team member not involved in participant contact to mask placebo versus active ECs. We restricted flavour options to regular tobacco flavour or menthol to most closely match usual cigarette brand flavour profile and reduce unwanted variance in product”.
Blinding of outcome assessment (detection bias) All outcomes	Low risk	Quote: “Participants and research staff conducting sessions were blinded to dose. All cartridges were pre‐loaded by the manufacturer. Labeling was removed by a research team member not involved in participant contact to mask placebo versus active ECs. We restricted flavour options to regular tobacco flavour or menthol to most closely match usual cigarette brand flavour profile and reduce unwanted variance in product”.
Incomplete outcome data (attrition bias) All outcomes	Unclear risk	Not specified
Selective reporting (reporting bias)	Low risk	All expected outcomes reported

**Table:** Morphett 2022a

***Study characteristics***		
Methods	Design: RCT Setting: Australia Recruitment: commercial market research panel and supplemental recruitment by study researchers. An invitation to participate was available to 46 857 I‐view panel members, including those with no smoking status recorded. In addition, advertisements to the general public were used to recruit additional participants. Study start date: 2014	
Participants	Total: N 1712 (table 1 full sample 1562) Arm A [Standard cessation advice & NRT (NRT short‐term)] flow diagram 368 (324 received intervention) Arm B [Quit or substitute advice and NRT: advice to use NRT as a longer‐term substitute for smoking if required to maintain smoking cessation] flow diagram 671 (620 received intervention) Arm C [Quit or substitute advice and NRT and/or e‐cigarettes] flow diagram 673 (619 received intervention) Inclusion criteria: Current daily smoking (at least 6 cpd). Agree to try samples of nicotine products. 18+ years Exclusion criteria: Currently being treated for serious cardiovascular disease, cancer, taking regular medication for mental health condition, uncontrolled high blood pressure, stomach ulcer, kidney or liver disease, overactive thyroid or adrenal gland cancer. Use insulin for diabetes. Asthma or chronic throat disease. Pregnant or planning to become pregnant/breastfeeding Female 64%; mean age 46.7 (SD 12.3); mean CPD 18.2 (SD8.7) E‐cigarette use at baseline: 28% had previously tried EC Motivated to quit: yes; 58% want to quit a lot	
Interventions	EC type: **cartridge** Arm C only: disposable e‐cigarette available in two strengths (free‐base nicotine 3.0% and 4.5%). A rechargeable version of the same brand with replaceable cartridges (3.0% v/v and 4.5% v/v) was substituted when the disposables were discontinued by the manufacturer (September 2014). The e‐cigarettes contained only nicotine, vegetable glycerin, and water, and were unflavoured. a) Arm A (usual care smoking cessation practice in Australia) comprising quit with NRT. Factsheet explaining the relative harm of NRT compared to smoking, free sample of NRT, participant chooses preferences, has the intervention free for 3 weeks then offered at a subsidized rate for further 6 months. The NRT products included nicotine gum, lozenges, inhalator, and mouth spray. Lozenges and gum were offered at two strengths. b) Arm B as (a), but with additional information provided: advice to quit or substitute with NRT c) Arm C as (a), but additional information on electronic cigarettes and emphasis on cessation, and may select electronic cigarettes as well as NRT	
Outcomes	Baseline, 7 months and 12 months, self‐report Continuous abstinence Self‐reported 7‐day point prevalence abstinence NRT and EC use Interest in quitting smoking and in quitting NRT Cigarette consumption Product orders and use Quit attempts AEs	
Study funding	Funding was from the National Health and Medical Research Council, Australia (#GNT1020123). The e‐cigarettes supplied in this trial were Vype brand, and were purchased from the manufacturer Nicoventures Trading Ltd., a UK‐based company that was a division of British American Tobacco. The other nicotine products were purchased in Australia from various distributors. Participant recruitment and survey data collection was managed by I‐View Social Research. None of these entities had any role in study design, data collection, data analysis, data interpretation, or writing of this paper.	
Author declarations	No authors have received financial support for the submitted work from any companies with a financial interest in the products under investigation. C.B. has undertaken consultancy for J&J Japan, a manufacturer of nicotine replacement therapy. N.W. and C.B. have completed a smoking cessation trial in which cytisine was supplied by Achieve Life Sciences, and a smoking cessation trial in which varenicline and matching placebo were supplied by Pfizer under their investigator‐initiated research programme. N.W. and C.B. have previously undertaken two trials of e‐cigarettes for smoking cessation (with e‐cigarettes purchased from a NZ e‐cigarette online retailer [NZVAPOR, https://www.nzvapor.com/], e‐liquid for one trial purchased from NicoPharm, Australia and nicotine patches supplied by the NZ government via their contract with Novartis [Sydney, Australia]). Neither NZVAPOR nor NicoPharm have links with the tobacco industry. None of the authors' spouses, partners, or children have financial relationships that may be relevant to the submitted work. All authors have no non‐financial interests that may be relevant to the submitted work.	
Notes	New to 2022 update	
***Risk of bias***		
**Bias**	**Authors' judgement**	**Support for judgement**
Random sequence generation (selection bias)	Low risk	Quote: "After completing the baseline survey, participants were block randomized in a 1:2:2 ratio to one of the three conditions by a computer generated random number sequence".
Allocation concealment (selection bias)	Low risk	Quote: "Participants and researchers were not blind to the allocated treatment; however, participants were not advised that there were different treatment conditions".
Blinding of participants and personnel (performance bias) All outcomes	Low risk	Quote: "Participants and researchers were not blind to the allocated treatment; however, participants were not advised that there were different treatment conditions".
Blinding of outcome assessment (detection bias) All outcomes	High risk	Self‐report
Incomplete outcome data (attrition bias) All outcomes	Low risk	Quote: "72.5% of participants who received the allocated intervention in Condition A completed the 7‐month survey, compared with 74.4% of Condition B and 72.9% of Condition C". At 12 months: arm A 233/324; arm B 457/671; arm C 448/673
Selective reporting (reporting bias)	Low risk	Specified outcomes reported

**Table:** Morphett 2022b

***Study characteristics***		
Methods	Design: RCT, pragmatic, randomized, partial cross‐over Setting: Australia Recruitment: not stated Study start date: recruited in 2018‐2019	
Participants	Total: N 355 Arm A: 181 Arm B: 174 Inclusion criteria: Diagnosed with/treatment for HIV or hepatitis C (HCV) or receiving opioid substitution therapy (OST). Diagnosed with or receiving treatment for priority health conditions in the past 12 months. Referral to Quitline counselling and smoking cessation support programme (standard care) but has not begun quit attempt. 18+ years; currently smoke 10+ cigarettes per day; willing to make a quit attempt Exclusion criteria: Already started quit attempt (i.e. post‐quit day) or enrolled in another smoking cessation clinical trial or using varenicline or bupropion or used a nicotine vaporizer product in the last 30 days. Health reason (e.g. CVD, terminal illness, recent hospitalization for mental health reason, pregnancy)	
Interventions	EC type: **refillable** **Arm 1)** Referral to Quitline telephone smoking cessation counselling + **Nicotine patches** (15 mg/16‐hr) delivered at baseline + **refillable nicotine vaporizer device** (2 x kits) + nicotine vaporising liquid (in high and low strength ‐ high strength: nicotine 1.8%; low strength: nicotine 0.6%). 1 patch to be applied daily to skin for up to 84 days. The vaporizer with nicotine liquid is to be used as needed, up to 3.5 mL per day to treat withdrawal symptoms for up to 2 years (concurrently with patches for the first 84 days) to assist smoking cessation and relapse prevention. Participants start on high‐strength nicotine liquid and may decrease their dose to low strength to assist with dose reduction prior to stopping use of the vaporizer. **Arm 2)** Referral to Quitline telephone smoking cessation counselling + **Nicotine patches** (15 mg/16‐hr) + participant’s choice of either **nicotine gum or nicotine lozenges** (up to 800 x 4 mg pieces to be used up to 8 per day) delivered at baseline. Between 6 and 9 months post‐baseline ‐ participants in Arm 2 who are smoking (either failed to quit or relapsed) will be offered: refillable nicotine vaporizer (2 x kits) + nicotine vaporizing liquid (in high and low strength ‐ high strength: nicotine 1.8%; low strength: nicotine 0.6%) to make a second quit attempt. Participants will have until 2 years from baseline to use the vaporizer for smoking cessation and relapse prevention. Arm B participants who were smoking at 6 months were offered the NVP intervention (NVPs as second‐line therapy). Switched over to EC intervention, called Arm C	
Outcomes	Baseline 6 months, 12 months, 24 months Primary outcomes: Continuous abstinence from smoking from weeks 12 to 26 assessed at 26 weeks from baseline by self‐report (bio‐confirmed) Secondary outcomes: Continuous abstinence from smoking AEs at 12 weeks and 26 weeks Abstinence is assessed through study‐specific survey questions in Module CS. Combustible Smoking Questions – urine specimens will be batch‐tested for anabasine and cotinine at 6‐, 12‐, and 21‐month time points.	
Study funding	National Health and Medical Research Council	
Author declarations	Not reported	
Notes	Abstract only New to 2022 update	
***Risk of bias***		
**Bias**	**Authors' judgement**	**Support for judgement**
Random sequence generation (selection bias)	Unclear risk	No detail
Allocation concealment (selection bias)	Unclear risk	No detail
Blinding of participants and personnel (performance bias) All outcomes	Low risk	Intervention arms both received interventions.
Blinding of outcome assessment (detection bias) All outcomes	Low risk	Participants that self‐report abstinence from smoking will be asked for a urine specimen for bio‐confirmation.
Incomplete outcome data (attrition bias) All outcomes	Unclear risk	No detail in conference abstract
Selective reporting (reporting bias)	Unclear risk	Conference abstract. Outcomes may be reported more fully at a later date.

**Table:** Morris 2022*

***Study characteristics***		
Methods	Design: randomized, cross‐over, open‐label, 2‐part study Recruitment: study participants were recruited from areas surrounding the study sites (Celerion, Lincoln, NE; Frontage, Secaucus, NJ) using standard advertising methods and were compensated for their participation in the study. Setting: 2 clinical research centres USA (Celerion, Lincoln, NE; Frontage, Secaucus, NJ). Participants were confined to the respective clinics for the full duration of the study. Study start date: November 2019. Study end date: January 2020	
Participants	Total N: 79 (single‐arm) Inclusion criteria: Smoking an average of > 10 manufactured combustible cigarettes pd for at least 12 months Exclusion criteria: Relevant illness history; presence of clinically significant mental or physical health conditions; high blood pressure; acute illnesses (e.g. upper respiratory infection, viral infection); relevant medication use; use of prescription smoking cessation treatments, anti‐diabetic or insulin drugs or medications known to interact with Cytochrome P450 2A6; body mass index (BMI) > 40 kg/m^2^ or < 18 kg/m^2^; allergy to propylene glycol or glycerin; planning to quit smoking during the study; pregnancy/breastfeeding; urine screen for alcohol or drugs of use Participants were between 21 and 65 years. CPD 'at least 10' cpd. Percentage women not reported Motivated to quit: no E‐cigarette use at baseline: no	
Interventions	EC: **Pod** Myblu™ two‐piece closed system comprised of a rechargeable 350 mAh battery and disposable pod containing an e‐liquid Sixteen commercial disposable liquid pod variants; different flavours; 5 different strengths (12, 24, 25, 36, 40 mg/mL); 2 forms of nicotine: nicotine salt or free‐base nicotine The e‐liquid mixtures consisted of VG, PG, nicotine, and a proprietary blend of favours; pods contained 1.5 mL of e‐liquid, equating to approximately 200 puffs under standardized machine puffing conditions. The ENDS were charged and assembled for the participants and product information sheets provided. On each study day, fresh pods and a fully charged device were provided. All participants received training from clinic staff on how to operate their ENDS and to ensure compliance in the clinic; all participants used their products under the supervision of suitably qualified staff.	
Outcomes	Baseline (‐2 day), day 9, day 14 15 biomarkers of exposure (BoE) to selected harmful and potentially harmful constituents (HPHCs) associated with tobacco smoking AEs	
Study funding	This work was funded entirely by Fontem US LLC, a subsidiary of Imperial Brands PLC.	
Author declarations	This work was funded by Fontem US LLC, a subsidiary of Imperial Brands PLC, and manufacturers of the myblu™ products used in this study. This work was done by Imperial Brands PLC on behalf of Fontem US LLC as a service provider. Work was contracted to Celerion, who conducted the study and analysed the data. At the time of the study and/or writing, PM, SM, FC, TV, XC, MS, JT, NC and GOC were employees of Imperial Brands PLC.	
Notes	Part 1 study data used only New to 2022 update	
***Risk of bias***		
**Bias**	**Authors' judgement**	**Support for judgement**
Random sequence generation (selection bias)	High risk	Study randomized, though for our analyses we treat as single‐arm. Quote from protocol: “Subjects who complete the study screening assessments and meet all the eligibility criteria and are randomized will be assigned a unique randomization identification number on day 1 for part 1 and on day 10 for part 2, and will receive study products according to the randomization scheme generated by Celerion”.
Allocation concealment (selection bias)	Unclear risk	No detail
Incomplete outcome data (attrition bias) All outcomes	Low risk	Quote: “Seventy‐two subjects completed the study and met the conditions for inclusion in the data analysis (out of a total of 79 recruited).”
Selective reporting (reporting bias)	High risk	FEV1 and FVC clinical trial registry outcomes not included in paper

**Table:** Myers‐Smith 2022

***Study characteristics***		
Methods	Design: RCT Recruitment: Clients of Queen Mary University of London's community stop‐smoking service who did not manage to stop smoking with routine treatment were invited to take part. Also recruited eligible smokers seeking help with quitting via social media. Setting: Queen Mary University of London, community stop‐smoking service Study start date: April 2017 to August 2018	
Participants	Total: N = 135 N per arm: E‐cigarette 68; NRT 67 Inclusion criteria: History of failed quit attempts using stop‐smoking medications and/or stop‐smoking services. Willing to use their allocated harm‐reduction strategy for at least 4 weeks. 18+ years Exclusion criteria: Currently using EC or any stop‐smoking products. Strong preference to use or not to use NRT or EC. Pregnancy or breastfeeding Women: 49%; mean age 40; median CPD 15 (IQR 10); median FTND: 5 EC arm, 4 NRT arm; motivated to quit E‐cigarette use at baseline: percentage tried EC earlier: 31% EC arm, 49% NRT arm	
Interventions	EC: **Refillable** EC Arm. EC starter pack and instructions to purchase further e‐liquids of flavour and strength of their choice (voucher for up to £40). Participants paid for further supplies themselves. They were encouraged to try e‐liquids of different strengths and flavours if the initial purchase did not meet their needs. Up to 8 weeks supply. Minimal behaviour support NRT Arm: NRT of choice. The choice of products included nicotine patch, chewing gum, nasal spray, microtab, inhalator, and mouth spray. Up to 8 weeks supply. At the baseline visit, participants selected an NRT product or product combination. Minimal behavioural support Both groups: 2 face‐to‐face sessions (baseline and week 1). Baseline: Participants selected products of their choice and received instructions on how to obtain them. Week 1: Smokers bring allocated product to the session, receive advice on use, test and start product use. Commitment to not using unallocated products for the next 4 weeks. Those wishing to stop smoking altogether were asked to set a target quit date (TQD). Participants received phone calls 1 and 4 weeks later to monitor product use and smoking status and to provide brief support.	
Outcomes	Baseline (week 0), week 1, week 4, week 24 Primary outcome measure: Cigarette consumption per day, assessed by self‐report in the follow‐up survey created for the purpose of the study at 1, 4, and 24 weeks post‐quit date/preparation date. Those who report ≥ 50% smoking reduction will be validated with a CO reading in the clinic. Secondary outcome measures: 1. Use of allocated harm reduction strategies. 2. Strategy ratings. 3. Changes in smoking behaviour. 4. Proportion of people still using allocated strategy at 6 months. All measured by the follow‐up survey created for the purpose of the study at 1, 4, and 24 weeks post‐quit date/preparation date. AEs	
Study funding	The study was funded by a Tobacco Advisory Group project grant, Cancer Research UK (C6815/A20503).	
Author declarations	PH and HM have received research funding from and provided consultancy to Pfizer, a manufacturer of stop‐smoking medications. DP has received research funding from Pfizer. All other authors had no conflicts to declare.	
Notes	Participants invited for CO readings at 4 weeks and 6 months received £10 in compensation for their time and travel at both visits. New to 2022 update	
***Risk of bias***		
**Bias**	**Authors' judgement**	**Support for judgement**
Random sequence generation (selection bias)	Low risk	Quote: “Randomisation sequences (1:1 ratio in permuted blocks of 20) were produced by an independent statistician using computer generated randomisation codes.“
Allocation concealment (selection bias)	Low risk	Quote: “Codes were sealed in opaque envelopes and marked with a unique randomisation number. Study staff allocated randomisation numbers sequentially. Staff opened the next envelope and entered the allocation onto the clinical record form (CRF) and randomisation log.”
Blinding of participants and personnel (performance bias) All outcomes	Low risk	Quote: “Data analysis was completed blind by an independent statistician.” Both arms active intervention
Blinding of outcome assessment (detection bias) All outcomes	Low risk	Quote: “Data analysis was completed blind by an independent statistician.”
Incomplete outcome data (attrition bias) All outcomes	Low risk	Quote: “Follow‐up rates were 85% and 88% at 4 weeks and 88% and 70% at 6 months in the EC and NRT group, respectively.”
Selective reporting (reporting bias)	Low risk	Outcomes registered were all reported.

**Table:** NCT02648178

***Study characteristics***		
Methods	Setting: medical centre, USA Recruitment: people with cancer Design: non‐randomized, single‐group assignment trial Recruitment: clinical settings, including outpatient clinics and the infusion suite Study start date: June 2016; study end date: May 2018	
Participants	Total N: 19 Inclusion criteria: histological or cytological diagnosis of aerodigestive tract cancers or bladder cancer within the past 5 years (≥ 1 tobacco‐related malignancy is allowed); AJCC stages I‐IV; daily smoking (≥ 10 CPD for 10 years) and breath CO^2^ ≥ 8 ppm; does not wish to quit smoking now (anyone wishing to quit smoking will be referred for smoking cessation counselling through the WRJ VAMC or DHMC programme); may be receiving anti‐cancer agents; age ≥ 18+ years Exclusion criteria: cancer surgery or radiation planned in the next 9 weeks; actively trying to quit smoking, or planning to in the next 30 days; any EC use in the past 30 days; pregnant or trying to get pregnant Inclusion based on specific population characteristics: patients with stage I‐IV aerodigestive tract cancers or bladder cancer who smoke daily 42.1% women; mean age: not reported ‐categories 18 to 65 years: N = 9, > 65 years: N = 10; cpd and FTND: not reported Motivated to quit: no (inclusion criterion) E‐cigarette use at baseline: not specified but EC use within 30 days is an exclusion criterion	
Interventions	**EC: Cig‐a‐like and refillable** Instructed on the use of EC, and given a supply that is "approximately equivalent to their current nicotine intake". Given Halo Triton EC (leak‐proof refillable tank system) or Halo G6 leak‐proof prefilled cartomizers. Began participants with 18 mg/mL and moved nicotine content up or down based on participant preference. Choice of flavours, provided for 9 weeks	
Outcomes	Weeks 3, 6, 9, 12. Self‐report at clinic visits Adverse events and biomarkers: Adverse events assessed with a checklist for commonly occurring side effects from e‐cigarettes and nicotine products Exhaled carbon dioxide Expired carbon monoxide Urine propylene glycol Urine 4‐ (methylnitrosamino)‐1‐(‐3pyridyl)‐1butanol (NNAL) 40 and 1‐ hydroxy naphthalene (1‐HOP) Other outcomes measured: Timeline Follow‐Back Questionnaire (TLFB) EC appeal assessed with attitudinal ratings, on a 5‐point Likert‐type scale e‐cigarette ease of use, satisfaction, and enjoyment, and willingness to continue to purchase e‐cigarettes in the future Change in daily cigarette smoking given 10 or more E‐cig sessions Average number of E‐cigs used per day The co‐ordinators will conduct and audiorecord a 10‐ to 15‐minute qualitative interview at 9 weeks soliciting perceptions about e‐cigarettes to be transcribed and analyzed for common themes that could be useful in developing the larger intervention. Urine nicotine and cotinine	
Study funding	Sponsors Dartmouth‐Hitchcock Medical Center	
Author declarations	Not reported – data extracted from clinical trial registry record	
Notes	Study listed as ongoing study in the 2016 review update	
***Risk of bias***		
**Bias**	**Authors' judgement**	**Support for judgement**
Random sequence generation (selection bias)	High risk	Not randomized, single‐group assignment
Allocation concealment (selection bias)	High risk	Not randomized, single‐group assignment
Incomplete outcome data (attrition bias) All outcomes	Low risk	19 enrolled; 10 participants followed up at 12 weeks
Selective reporting (reporting bias)	Unclear risk	The following measures were not reported: exhaled carbon dioxide; urine propylene glycol; urine nicotine, cotinine, NNAL and 1‐ hydroxy naphthalene (1‐HOP), and Timeline Follow‐Back Questionnaire (TLFB). Data at 6, 12 months also not reported

**Table:** NCT02918630

***Study characteristics***		
Methods	Design: RCT Recruitment: clinics Setting: SMI clinics, USA Study start date: October 2016; study end date: August 2017	
Participants	Total N: 7 N per arm: NRT: 4; EC + NRT 3 Inclusion criteria: diagnosed with schizophrenia (or other SMI, not clear); be in stable medical condition (DSM‐V); report smoking ≥ 10 tobacco cigarettes/day; breath CO ≥ 10 ppm; report wanting to reduce their cigarette smoking; stable living situation Exclusion criteria: pregnant or breastfeeding; report wanting to quit smoking in the immediate future; test positive for illicit drugs except THC; any illness, medical condition, or use of medications, which in the opinion of the study physicians would preclude safe or successful completion of the study, or both Inclusion based on specific population characteristics: yes ‐ SMI (schizophrenia and schizoaffective disorder, bipolar disorder, or PTSD) 43% women; mean age 48.3; mean cpd: NR; mean FTND: NR Motivated to quit: Wanted to quit or reduce their cigarette smoking but did not want to quit in the immediate future (this was an exclusion criterion). NB – trial registry stated wanted to reduce and protocol stated wanted to quit or reduce as inclusion criteria. E‐cigarette use at baseline: not specified	
Interventions	**EC: Refillable** Both arms received a nicotine patch 21 mg for 4 weeks. **EC + NRT**: 4 weeks: 1) a 3.3 V, 1000 mAh battery; and 2) a 1.5 Ohm, dual‐coil cartomizer (SmokTech; Shenzhen, China). Nicotine concentrations 36 mg/mL. Verbal and written instructions on how to use and maintain the e‐cigarettes at week 1 visit. Nicotine patch (21 mg) **NRT arm:** NRT only (nicotine patch, 21 mg)	
Outcomes	5 weeks Cessation: n/a but “change in smoking” Adverse events and biomarkers: Breath CO, COPD‐related symptoms, EC side effects (e‐cig side effects questionnaire), AEs, SAEs Other outcomes measured: Urinary cotinine, cpd, tobacco dependence, craving, withdrawal symptoms, desire to quit, confidence to quit, EC dependence, EC use, satisfaction with EC, nicotine dependence, schizophrenia symptoms (brief psychiatric rating scale), cognitive domains associated with schizophrenia (MATRICS consensus cognitive battery), changes in positive symptoms of schizophrenia (scale for the assessment of positive symptoms), changes in negative schizophrenia symptoms (scale for the assessment of negative symptoms), suicide ideation (Columbia Suicide Severity Rating Scale)	
Study funding	Sponsors: The University of Texas Health Science Center, Houston	
Author declarations	Not reported	
Notes	New for 2020 update. Information from clinical.trials.gov [http://clinical trials gov] registry and unpublished protocol; discrepancies between the two in terms of trial methods. Feasibility for future NIH grant application. Intended to recruit 20 participants, but only 7 started and completed.	
***Risk of bias***		
**Bias**	**Authors' judgement**	**Support for judgement**
Random sequence generation (selection bias)	Unclear risk	Not reported
Allocation concealment (selection bias)	Unclear risk	Not reported
Blinding of participants and personnel (performance bias) All outcomes	Unclear risk	“double‐blind” but “open‐label” elsewhere, no further info given
Blinding of outcome assessment (detection bias) All outcomes	Unclear risk	Not reported
Incomplete outcome data (attrition bias) All outcomes	Unclear risk	Not reported
Selective reporting (reporting bias)	High risk	Schizophrenia and COPD outcomes not reported
Other bias	Unclear risk	Some discrepancies between clinical trials record and protocol linked to from record, including when NRT started and inclusion criteria (just schizophrenia or all SMI). Target sample size was 20 but only 7 people recruited

**Table:** NCT04084210

***Study characteristics***		
Methods	Design: RCT Setting: USA. University of Wisconsin Center for Tobacco Research and Intervention Recruitment: participants from the greater Madison and Milwaukee, Wisconsin areas will be recruited via media recruitment methods (i.e. television, newspaper, and earned media) that have recruited thousands of smokers. Investigators will also use Internet/Facebook advertisements. Study start date September 2019. Study end date May 2023	
Participants	Randomised 209 (160 completed both switch weeks) Number analyzed: Juul EC + active and placebo patch 54 VLNC + active and placebo patch 53 No product + active and placebo patch 53 60% female; 21.9% African‐American; 51.9 (SD 12.1) age; 16.8 (SD 9.3) CPD Inclusion criteria: Smoking > 4 cigarettes/day for the previous 6 months, (CO) > 6 ppm No plans to quit smoking in the next 30 days Not currently taking smoking cessation medication Willing and medically able to use nicotine patches 21+ years Exclusion criteria: Currently in treatment for psychosis or bipolar disorder EC use within the last month Pregnant or breastfeeding Motivated to quit: no	
Interventions	EC: pod Juul Electronic cigarette. The Juul e‐cigarette pods contain 0.7 mL nicotine by volume/5% nicotine by weight. Tobacco or menthol flavour Very Low Nicotine Cigarettes: These very low nicotine cigarettes (VLNCs) consist of reduced nicotine cigarettes containing 0.03 mg of nicotine; these VLNCs were obtained from the National Institute on Drug Abuse (NIDA's) Drug Supply Program (NOT‐DA‐14‐004). Active nicotine patches, with dosing based on the package insert (> 10 cigs/day = 21 mg patch and < 11 cigs/day = 14 mg patches) Placebo patch containing no nicotine Arm 1: Active comparator: Juul + active patch in wk 1 and placebo patch in wk 2 Participants will be given Juul e‐cigarettes for 4 weeks; in switch week 1, participants will also use active nicotine patches; in switch week 2, participants will use placebo patches. Arm 2: Active comparator: Juul + placebo patch in wk 1 and active patch in wk 2 Participants will be given Juul e‐cigarettes for 4 weeks; in switch week 1, participants will also use placebo patches; in switch week 2, participants will use active nicotine patches. Arm 3: Active comparator: VLNC + active patch in wk 1 and placebo patch in wk 2 Participants will be given very low nicotine cigarettes (VLNCs) for 4 weeks; in switch week 1, participants will also use active nicotine patches; in switch week 2, participants will use placebo patches. Arm 4: Active comparator: VLNC + placebo patch in wk 1 and active patch in wk 2 Participants will be given very low nicotine cigarettes (VLNCs) for 4 weeks; in switch week 1, participants will also use placebo patches; in switch week 2, participants will use active nicotine patches. Arm 5: No product + active patch in wk 1 and placebo patch in wk 2 Participants will be given no alternative nicotine delivery products, but in switch week 1, participants will use active nicotine patches; in switch week 2, participants will use placebo patches. Arm 6: No product + placebo patch in wk 1 and active patch in wk 2 Participants will be given no alternative nicotine delivery products for 2 weeks, but in switch week 1, participants will use placebo patches; in switch week 2, participants will use active nicotine patches.	
Outcomes	Weeks 1 to 4 Weeks 1 through 4, participants will use a smartphone to record, in the moment, each time they use their own cigarettes or any alternative product. Primary outcome: number of conventional cigarettes smoked during each switch week Secondary outcome: number of VLNCs or Juul pods used during each switch week SAE, AE	
Study funding	Sponsors and Collaborators University of Wisconsin, Madison National Cancer Institute (NCI)	
Author declarations	Not available; abstract and trial registry only	
Notes	New to 2023 (previously ongoing) as adverse event data now included in trial registry. However, distribution of AEs across randomised groups is unclear and so data is thus far only included in narrative synthesis and Supplementary Materials. When further information is available, these will be moved to meta‐analyses where appropriate.	
***Risk of bias***		
**Bias**	**Authors' judgement**	**Support for judgement**
Random sequence generation (selection bias)	Low risk	Electronic randomization using trial database
Allocation concealment (selection bias)	Unclear risk	Not described
Blinding of participants and personnel (performance bias) All outcomes	High risk	No placebo e‐cigarette
Blinding of outcome assessment (detection bias) All outcomes	High risk	Different intensities of interventions. AEs self‐reported
Incomplete outcome data (attrition bias) All outcomes	Low risk	160/209, 76.5%
Selective reporting (reporting bias)	Low risk	Results posted on NCT record

**Table:** Nides 2014*

***Study characteristics***		
Methods	Design: open‐label, non‐comparative study Recruitment: study site database and community advertisements Setting: Clinical Trials Unit, USA Study start date: April 2013; study end date: 10 July 2013	
Participants	Total N: 29 Inclusion criteria: age 18 to 65 years; good health; BMI 18 to 35; smoking 10+ cpd; CO > 10 ppm Exclusion criteria: pregnancy or breastfeeding; other drug dependency; use of any psychiatric or opioid medications; EC within the previous 14 days; use of NRT in last 30 days' want to reduce or quit smoking within the next 30 days Exclusion criterion: EC within the previous 14 days; use of NRT in last 30 days 44% women; mean age 43; mean cpd 20.1; mean FTND 4.5 Motivated to quit: no E‐cigarette use at baseline	
Interventions	**EC: Cig‐a‐like** Participants attended 3 clinic visits at 1‐week intervals Visit 1: baseline Visit 2: Provided with 1st generation type ‐ 'NJOY® King Bold' (NJOY, Inc. Scottsdale, AZ), with 26 mg nicotine. Used ad libitum for 20 minutes in the clinic, then ad libitum use over the next week. Recorded use of regular cigarettes and puffs on EC Visit 3: Participants abstained from all sources of nicotine for 12 hours prior to visit.	
Outcomes	Adverse events	
Study funding	Funding for this study was provided by NJOY, Inc., Scottsdale, AZ.	
Author declarations	Dr Nides has received compensation from NJOY, Inc. and GlaxoSmithKline. Dr Leischow has received compensation from GlaxoSmithKline, Pfizer, and Cypress Bioscience. Mr Simmons and Ms Bhatter have no conflict of interest to report.	
Notes		
***Risk of bias***		
**Bias**	**Authors' judgement**	**Support for judgement**
Random sequence generation (selection bias)	High risk	Prospective cohort
Allocation concealment (selection bias)	High risk	Not randomized
Incomplete outcome data (attrition bias) All outcomes	Low risk	2 participants dropped out between visits 1 and 2.
Selective reporting (reporting bias)	Low risk	Planned comparisons reported

**Table:** Okuyemi 2022

***Study characteristics***		
Methods	Design: RCT Recruitment: The study identified African‐American current cigarette smokers using the electronic health record in the University of Minnesota Fairview Health system. The authors sent recruitment letters that provided a brief description of the study and invited recipients who were interested in the study to contact study staff. Participants were also invited to refer contacts outside their family to the study. Setting: Study visits were conducted in the Delaware Clinical Research Unit (DCRU) through the Clinical and Translational Science Institute (CTSI) at the University of Minnesota, USA. Study start date: June 2018. Study end date September 2019 (NCT record: Start date: 15 November 2016. End date: 8 March 2019)	
Participants	Total N: 234 Nicotine EC arm: 118 Non‐nicotine EC arm: 116 Inclusion criteria: 1) Self‐identification as African‐American or black, 2) smoked ≥ 5 cigarettes daily for the past year, smoking status confirmed by expired CO ≥ 5 ppm or positive NicAlert screen, 3) willingness to use EC, 4) 18 to 79 years Exclusion criteria: 1) Recent unstable or untreated psychiatric diagnosis including substance abuse (DSM‐IV criteria), 2) EC use in the past 30 days, 3) planning to quit smoking in the next 30 days, 4) pregnancy or nursing, 5) CO < 5 ppm and no cotinine detected in the urine Female: 43.9%; mean age 50.8 (SD 11.2); mean CPD 11.5 (SD 6.0) Motivated to quit: no E‐cigarette use at baseline: no	
Interventions	EC: **Refillable** EC with 24 mg of nicotine added. Nicotine EC rechargeable Halo G6 brand 2.4% nicotine (24 mg, equivalent to the nicotine content of combustible cigarettes) EC ‐ No‐nicotine EC rechargeable Halo G6 brand 0% nicotine (0 mg) For both groups: A free Halo G6 brand rechargeable EC starter kit with the accessories including the charger, batteries, and a 2‐week supply of liquid cartridges. The Halo G6 device was 3.3 to 4.2 (average 3.7) volts. The prefilled cartomizers coil resistance was 2.2 to 2.8 ohms. At the week‐2 visit, participants received an additional 4‐week supply of cartridges. Participants were given oral and written instructions about how to use the products. Ad lib for 6 weeks. All participants were provided with EC by the study; menthol and non‐menthol flavoured EC cartridges were available. Participants could purchase their own if needed after 6 weeks. Participants were compensated for their time and transportation: USD 40 at baseline, USD 40 at week 2, USD 50 at week 6, and USD 20 at week 12, for a maximum of USD 150 over 12 weeks.	
Outcomes	Baseline, 2, 6, and 12 weeks (all visits were in‐person except week 12, which was a telephone survey) Biomarkers at baseline and 6 weeks. Urinary biomarkers (NNAL, NNK) and total nicotine equivalents (TNE, total nicotine + total cotinine + total 3‐hydroxycotinine + nicotine N‐oxide). Expired carbon monoxide (CO) Combustible cigarettes self‐reported number of cigarettes smoked per day (and baseline). EC use (Penn State EC Dependence Index) EC dependence (10‐item Penn State EC Dependence Index) Nicotine withdrawal symptoms (baseline, week 2, 6, 12) (modified Minnesota Nicotine Withdrawal (MNWS)) Data collection was self‐administered and collected on electronic tablets.	
Study funding	ClearWay Minnesota Grant Award #RC‐2014‐0009	
Author declarations	The authors declared that they had no competing interests.	
Notes	New to 2022 update	
***Risk of bias***		
**Bias**	**Authors' judgement**	**Support for judgement**
Random sequence generation (selection bias)	Unclear risk	Quote: "To ensure balance in the number of intervention assignments between the study groups, randomization was blocked (block size unknown to staff or investigators) by nicotine versus no nicotine e‐cigarettes." No other information given
Allocation concealment (selection bias)	Unclear risk	No information given
Blinding of participants and personnel (performance bias) All outcomes	Low risk	Single‐blind. Interventions equally intensive
Blinding of outcome assessment (detection bias) All outcomes	Low risk	Biomarkers measured
Incomplete outcome data (attrition bias) All outcomes	Low risk	Nicotine EC arm: 109/118 Non‐nicotine EC arm: 106/116
Selective reporting (reporting bias)	Low risk	Reported outcomes in NCT record

**Table:** Oncken 2015

***Study characteristics***		
Methods	Design: randomized, cross‐over study Recruitment: newspaper advertisements, radio announcements, and from local general medicine practices Setting: lab‐based study, Connecticut, USA Study start date: October 2012; study end date: June 2015	
Participants	Total N: 27 Inclusion criteria: non‐treatment‐seeking people who smoke who were willing to try EC for 2 weeks and abstain from conventional cigarette smoking; 18 to 55 years of age who smoked at least 10 cpd Exclusion criteria: pregnant; previous myocardial infarction or stroke; uncontrolled hypertension (blood pressure (BP) > 160/100); insulin‐dependent diabetes; COPD or current asthma; known allergy to propylene glycol. 45% women; mean age 42; 70% white; 15% Hispanic, 15% black; mean cpd 16; 45% had tried EC at baseline, 50% smoked menthol cigarettes Motivated to quit: no E‐cigarette use at baseline: not specified	
Interventions	**EC: Cig‐a‐like** Prescribed Joye eGo‐C (www.joyetech.com) and e‐Juice (18 mg/mL nicotine) procured from American eLiquid (www.americanliquid.com) Cross‐over study between menthol‐flavoured and non‐menthol tobacco‐flavoured EC. Requested not to smoke their regular cigarettes during study period, but most (60%) reported intermittently smoking cigarettes during study	
Outcomes	Follow‐up at 1 week and 2 weeks BP, heart rate, body plethysmography, static lung volumes and airways resistance (Raw), and specific conductance (sGaw) – taken at lab visits after abstaining from EC for at least 2 hrs, then taken again after inhaling EC and repeated 5 mins later Adverse events also reported but method for measuring not stated Also measured nicotine concentrations, rates of cigarette and EC use	
Study funding	This project was supported by Academic Enhancement funds from the Department of Medicine at the University of Connecticut Health Center (to CO) and the Clinical Research Center at the University of Connecticut Health Center.	
Author declarations	CO is currently receiving study medication (nicotine inhaler and placebo) from Pfizer pharmaceuticals for an NIH‐funded nicotine inhaler for smoking cessation during pregnancy.	
Notes		
***Risk of bias***		
**Bias**	**Authors' judgement**	**Support for judgement**
Random sequence generation (selection bias)	Unclear risk	Method not stated Quote: "Subjects were then randomly assigned to use the menthol or plain e‐cigarette cartridge for one week, switching to the other cartridge for the second week".
Allocation concealment (selection bias)	Unclear risk	No detail given
Blinding of participants and personnel (performance bias) All outcomes	Low risk	No detail given on blinding but equal levels of support between arms, so performance bias judged unlikely
Blinding of outcome assessment (detection bias) All outcomes	Low risk	Some subjective outcomes but equal levels of support between arms, so differential misreport judged unlikely
Incomplete outcome data (attrition bias) All outcomes	Low risk	20/27 followed up
Selective reporting (reporting bias)	Unclear risk	Unable to determine prespecified outcomes

**Table:** Ozga‐Hess 2019

***Study characteristics***		
Methods	Design: RCT Recruitment: Cigarette smokers were recruited from the community via fliers, online postings, and word of mouth. Setting: Morgantown, West Virginia, USA Study start date: not reported. Study end date: not reported	
Participants	Total N: 60; E‐cigarette plus own brand = 30. Own brand cigarette (control) = 30 38.3% female; mean age completers 35.1 (SD 11) (N = 34), non‐completers 36.8 (SD 12.9) (N = 26); mean cpd completers 16.7 (SD 4.9), non‐completers 19.6 (SD 6.1); mean FTND completers 5.3 (SD 1.8), non‐completers 5.9 (SD 1.9) Inclusion criteria: 18 to 60 years; smoking ≥ 10 cigarettes per day for ≥ 1 year; exhaled air carbon monoxide (CO) level of ≥ 10 ppm (Micro+™ basic monitor; CoVita; Haddonfield, NJ); contemplation or Preparation Stage of Change (indicating interest in a quit attempt within the next 1 to 6 months) Exclusion criteria: reported chronic health or psychiatric conditions; past month use of marijuana ≥ 5 days; past month use of any other illicit drugs, or regular use of ECIGs or other tobacco products (i.e. ≥ 1 day per week); individuals in the Precontemplation (no interest in quitting) or Action (actively trying to quit) Stage of Change; pregnancy/breastfeeding	
Interventions	**EC: Refillable** **E‐cigarette** (18 mg/mL) plus own brand cigarette. Kanger mini Protank‐II, which is a 1.5 mL Pyrex glass tank with a drip tip and atomizer head coils (KangerTech; China), and a 3.3 V constant output, 900 mAh, eGo‐T battery (Joyetech; Irvine, CA). The liquid (The Vapor Room, Sky Vapors LLC, Frostburg, MD) was labelled as 70% propylene glycol and 30% vegetable glycerin, with a nicotine concentration requested of 18 mg/mL. Participants could choose tobacco, menthol or wild berry flavour and could switch between sessions. Ad libitum use for 4 weeks **Own brand cigarette** ad libitum use for 4 weeks	
Outcomes	Assessments days 8, 15, 22, and 29. FU after 1 month Daily for salivary cotinine samples. Daily self‐monitoring device to log e‐cigarette and cigarette use. Collected used cigarette filters Weekly CO breath test Attended the laboratory weekly for assessments (days 8, 15, 22, and 29). Then completed a follow‐up visit 1‐month post‐intervention Self‐reported withdrawal symptoms Reported experience of specific symptoms rated using a visual analogue scale with a range from 0 (not at all) to 100 (extremely). e.g. craving, irritability, dry mouth, throat irritation, and cough	
Study funding	Financial support provided to MDB and GAD by WVU Senate Grant for Research, and to GAD, MDB, and NAT by Cooperative Agreement Number 1‐U48‐DP‐005004 from the Centers for Disease Control and Prevention (CDC) to the West Virginia Prevention Research Center. Support provided to NJF and JEOH by the National Institute of General Medical Sciences (NIGMS T32 GM081741). Additional support provided by WV Tobacco Cessation QuitLine	
Author declarations	Author SGF has consulted for various pharmaceutical companies on matters relating to smoking cessation. All other authors declared that they had no conflicts of interest.	
Notes		
***Risk of bias***		
**Bias**	**Authors' judgement**	**Support for judgement**
Random sequence generation (selection bias)	Unclear risk	Quote: "Using a simple randomized design" Comment: not adequately explained
Allocation concealment (selection bias)	Unclear risk	Not adequately described in the paper
Blinding of participants and personnel (performance bias) All outcomes	High risk	Participants and investigators were not blind.
Blinding of outcome assessment (detection bias) All outcomes	Low risk	Biochemical validation
Incomplete outcome data (attrition bias) All outcomes	High risk	40% retention, but no difference between groups
Selective reporting (reporting bias)	Low risk	All outcomes reported

**Table:** Pacifici 2015

***Study characteristics***		
Methods	Design: uncontrolled pre‐post pilot study Recruitment: word of mouth Setting: hospital‐based smoking cessation clinic, Italy Study start date/end date: not specified	
Participants	Total N: 34 Inclusion criteria: adults who smoke, unwilling to quit smoking tobacco cigarettes and who have never tried a quit‐smoking protocol or have refused any smoking cessation treatment, or both Exclusion criteria: none stated Inclusion based on specific population characteristics: no 47.1% women, mean age 40.6, mean cpd 21.5 No EC use at baseline, not motivated to quit	
Interventions	**EC: Refillable** Participants were given commercially available EC (AVATAR device, Battery 550 mAh/3.9 V, W: 7.8, cartomizer with 2, 2 ohm resistance, tank capacity 1.5 mL, temperature of the aerosol: 55/65 degrees), 2 different chargers for each EC and PUFFIT e‐liquids with nicotine content matching the individual nicotine daily intake and tobacco and/or other flavours freely chosen by each participant. W1: nicotine‐free e‐liquid W2&3: Own EC with personal nicotine dosage, encouraged to use as substitute for traditional cigarettes W4: Encouraged to forego all traditional cigarettes Throughout: assistance at any time of day from centre staff with any EC‐related problem, plus follow‐up group sessions and smartphone messaging application Behavioural support: Multi‐component medically assisted training programme with monitoring of nicotine intake as a biomarker of correct EC use, including information about general working principles, safety and risks of EC, together with medically assisted face‐to‐face training on how to correctly use the device to absorb nicotine vapour	
Outcomes	Follow‐up at 1, 4, and 8 m Cessation (measure not defined) Adverse events Exhaled CO, COT, 3‐HCOT concentration cpd	
Study funding	The authors thank Renata Solimini, Adele Minutillo, Emilia Marchei and Maria Concetta Rotolo for their technical assistance. This work was supported by the Department of Therapeutic Research and Medicines Evaluation Istituto Superiore di Sanità, Roma, Italy.	
Author declarations	The authors declared no conflict of interest.	
Notes		
***Risk of bias***		
**Bias**	**Authors' judgement**	**Support for judgement**
Random sequence generation (selection bias)	High risk	Not controlled
Allocation concealment (selection bias)	High risk	Not controlled
Incomplete outcome data (attrition bias) All outcomes	Low risk	All participants followed up
Selective reporting (reporting bias)	High risk	AEs measured but not reported

**Table:** Polosa 2011*

***Study characteristics***		
Methods	Design: prospective cohort Recruitment: advertisements in local hospital in Catania, Italy Setting: not specified Study start date: February 2010; study end date: June 2010	
Participants	Total N: 40, hospital staff Inclusion criteria: healthy people who smoke; 18 to 60 years; smoking ≥ 15 cpd for ≥ past 10 years, and not wanting to quit smoking at any time in the next 30 days Exclusion criteria: history of alcohol and illicit drug use; psychiatric illness; recent myocardial infarction; angina pectoris; high blood pressure (BP > 140 mmHg systolic or 90 mmHg diastolic, or both); diabetes mellitus; severe allergies; poorly‐controlled asthma or other airways diseases 35% women, mean age 42.9 (SD 8.8), median cpd 25 (IQR 20 to 30), median FTND 6.0 (IQR 6 to 8) Motivated to quit: no E‐cigarette use at baseline: not specified	
Interventions	**EC: Cig‐a‐like** Seen at baseline, given EC ('Categoria' brand) with an initial 4‐week supply of 7.4 mg nicotine cartridges. Instructed to use ad libitum up to 4 cartridges per day. EC cartridges supplied at months 1, 2, and 3 No instruction on cessation or reduction was provided.	
Outcomes	Follow‐up at 1, 2, 3, 6, 18, and 24 months when cigarette consumption, CO, and AEs were measured, incl. 30‐day PP CO‐validated abstinence at 6 months and CO‐validated abstinence at 18 and 24 months (not otherwise defined) Adverse events	
Study funding	"We wish to thank Arbi Group Srl (Milano, Italy) for the free supplies of ‘Categoria’ e‐Cigarette kits and nicotine cartridges as well as their support. We would also like to thank the study participants for all their time and effort and LIAF (Lega Italiana AntiFumo) for the collaboration."	
Author declarations	"None of the authors have any competing interests to declare, but RP has received lecture fees from Pfizer and, from Feb 2011, he has been serving as a consultant for Arbi Group Srl.Arbi Group Srl (Milano, Italy), the manufacturer of the e‐Cigarette supplied the product, and unrestricted technical and customer support. They were not involved in the study design, running of the study or analysis and presentation of the data."	
Notes	Smoking cessation services provided to those who spontaneously asked for assistance with quitting. These participants were excluded from the study protocol.	
***Risk of bias***		
**Bias**	**Authors' judgement**	**Support for judgement**
Random sequence generation (selection bias)	High risk	Prospective cohort
Allocation concealment (selection bias)	High risk	Not randomized
Incomplete outcome data (attrition bias) All outcomes	Low risk	13/40 were lost to follow‐up, but used ITT analysis
Selective reporting (reporting bias)	Unclear risk	Unable to determine prespecified outcomes

**Table:** Polosa 2014b*

***Study characteristics***		
Methods	Design: prospective cohort study Recruitment: volunteers, leaflets, cessation service kiosk in hospital Setting: smoking cessation clinic, Italy Study start date: January 2013; study end date: November 2013	
Participants	Total N: 50 Inclusion criteria: healthy people who smoke; 18 to 60 years; smoking ≥ 15 conventional cpd ≥ 10 years; unwilling to quit Exclusion criteria: none stated 40% women; mean age 41; mean cpd 25; mean FTND 6.0 No EC use at baseline, not motivated to quit	
Interventions	**EC: Refillable** 2nd generation devices (personal vaporisers ‐ PVs): EGO/CE4 model, filled with tobacco aroma e‐liquid containing 9 mg/mL nicotine; instructed to use the study products ad libitum (up to a maximum of 5 ml/day; i.e. half vial) Behavioural support: Participants were instructed how to charge, fill, activate and use the EC. Key troubleshooting was addressed and phone numbers were supplied for assistance. “No emphasis on encouragement, motivation and reward for the smoking cessation‐related efforts were provided during the study.”	
Outcomes	4, 8, 12, and 24 weeks 30‐day PP verified by CO ≤ 10 ppm Adverse events Cpd, exhaled CO, reduction rates, product usage, and opinions of the EC products	
Study funding	"The authors wish to thank FlavourArt (Oleggio, NO, Italy; www.flavourart.it). Authors wish to thank LIAF, Lega Italiana Anti Fumo (Italian acronym for Italian Anti Smoking League) for supporting this research".	
Author declarations	"RP has received lecture fees and research funding from Pfizer and GlaxoSmithKline, manufacturers of stop‐smoking medications. He has also served as a consultant for Pfizer and Arbi Group Srl, an Italian distributor of e‐Cigarettes. RP is currently scientific advisor for LIAF, Lega Italiana Anti Fumo (Italian acronym for Italian Anti Smoking League). PC, MM, JBM, and CR have no relevant competing interest to declare in relation to this work".	
Notes		
***Risk of bias***		
**Bias**	**Authors' judgement**	**Support for judgement**
Random sequence generation (selection bias)	High risk	Not controlled
Allocation concealment (selection bias)	High risk	Not controlled
Incomplete outcome data (attrition bias) All outcomes	Low risk	76% followed up, ITT analysis used, no significant differences in baseline characteristics between completers and those lost to follow‐up
Selective reporting (reporting bias)	Unclear risk	Unable to determine prespecified outcomes

**Table:** Polosa 2015*

***Study characteristics***		
Methods	Design: prospective cohort Recruitment: professional retail staff in participating vape shops Setting: 7 vape shops in Catania province, Italy Study start date/end date: not specified	
Participants	Total N: 71 Inclusion criteria: adults who smoke (≥ 18 years); making first purchase at participating vape shop (definition of smoker not stated) Exclusion criteria: none stated 38% women, mean age 41.7, mean cpd 24.9, mean FTND 5 No EC use at baseline	
Interventions	**EC: Refillable** Instructed how to charge, fill, activate and use EC; key troubleshooting advice provided; phone number available for technical support; “Encouraged to use these products in anticipation of reducing the number of cig/day smoked”	
Outcomes	6 and 12 m follow‐up 30‐day PPA via self‐report Details of product purchase Sustained 50% and 80% reduction in cpd from baseline	
Study funding	Authors wish to thank the local participating Vape Shops and LIAF, Lega Italiana Anti Fumo (Italian acronym for the Italian Anti‐Smoking League) for supporting this research.	
Author declarations	Riccardo Polosa has received lecture fees and research funding from Pfizer and GlaxoSmithKline, manufacturers of stop‐smoking medications. He has also served as a consultant for Pfizer and Arbi Group Srl, an Italian distributor of e‐Cigarettes. Riccardo Polosa is currently scientific advisor for LIAF, Lega Italiana Anti Fumo (Italian acronym for Italian Anti‐Smoking League). Jacques Le‐Houezec is a consultant for Johnson & Johnson France, a manufacturer of nicotine replacement therapy, and was reimbursed for travel and accommodation to present at a conference in Shenzhen (China) organized by the e‐cig manufacturer association (CECMOL). Pasquale Caponnetto and Fabio Cibella have no relevant conflict of interest to declare in relation to this work.	
Notes		
***Risk of bias***		
**Bias**	**Authors' judgement**	**Support for judgement**
Random sequence generation (selection bias)	High risk	Not controlled
Allocation concealment (selection bias)	High risk	Not controlled
Incomplete outcome data (attrition bias) All outcomes	Low risk	69% follow‐up at 12 m. Participants lost to follow‐up considered as continuing smokers
Selective reporting (reporting bias)	Unclear risk	Unable to determine prespecified outcomes

**Table:** Pope 2024

***Study characteristics***		
Methods	RCT Two‐arm pragmatic, multicentre, parallel‐group, individually randomised controlled trial carried out at 6 UK NHS Emergency Departments Study start date January 2022	
Participants	People attending the Emergency Department who smoke Total N: 972. Intervention: 484; control: 488 Female 38%; mean age 40.5 (SD 13.65); median CPD 15 (IQR 10 to 20). Mean FTND 4.89 (SD 2.31). Motivation to quit score, mean 4.13 (SD 1.60). Inclusion: adults (aged 18 years or older) who reported smoking tobacco daily, attending the ED for medical treatment. Exclusion: expired carbon monoxide (CO) of < 8 per million (ppm), required immediate medical treatment, were in police custody, had a known allergy to nicotine, were current dual users (defined as daily e‐cigarette use).	
Interventions	EC intervention: 1) EC starter kit (DotPro, manufactured by Liberty Flights, an independent e‐cigarette manufacturer not funded by the tobacco industry) ‘pod’ device. The kit included 11 pods (3 tobacco flavoured, 4 berry flavoured and 4 menthol flavoured) of 20mg/mL nicotine strength. Training in use of EC. 2) Brief smoking cessation advice. 3) Referral to stop smoking services. FU consisted of telephone call offering support &, if taken up, offer of free NRT. Control: Treatment‐as‐usual. Signposting to NHS smoking cessation services through provision of written information about local services.	
Outcomes	Baseline, 1, 3, and 6 months Continuous smoking abstinence, 6 months after randomization, CO confirmed Smoking status 1, 3, and 6 months after randomization. Abstinence prevalence, CO validated (≥ 8 ppm) Number of cpd; number of times using an EC per day; self‐reported dry cough; mouth or throat irritation; use of GP services; use of smoking cessation services. Quality of Life questionnaire. Adverse events (6 months).	
Study funding	National Institute of Health Research, Health Technology Assessment (129438)	
Author declarations	None declared	
Notes	Data new to 2024 update	
***Risk of bias***		
**Bias**	**Authors' judgement**	**Support for judgement**
Random sequence generation (selection bias)	Low risk	Computer‐generated randomization employed varying block sizes and stratified by the recruitment sites, which allowed for concealment of allocation
Allocation concealment (selection bias)	Low risk	Computer‐generated randomization allowed for concealment of allocation
Blinding of participants and personnel (performance bias) All outcomes	High risk	Due to the participatory nature of the intervention, it was not feasible to blind participants or those delivering the intervention to group allocation Interventions not equally intensive
Blinding of outcome assessment (detection bias) All outcomes	Low risk	CO measured
Incomplete outcome data (attrition bias) All outcomes	Low risk	EC 351/484 = 72.5% Control 317/488 = 65%
Selective reporting (reporting bias)	Low risk	Reported as pre‐specified. Cessation and AEs

**Table:** Pratt 2016

***Study characteristics***		
Methods	Design: 0bservational study – uncontrolled experimental study Recruitment: community mental health centre through self‐referral and clinician referrals Setting: community mental health centre (USA) Study start date: October 2013; study end date: June 2014	
Participants	Total N: 19 (21 originally recruited, however 2 participants did not return for any weekly visits so 19 analyzed) Inclusion criteria: Age ≥ 18 years Primary DSM‐IV axis I diagnosis, based on chart review and confirmation by the community mental health centre team psychiatrist, of schizophrenia, schizoaffective disorder, or bipolar disorder SMI defined by at least moderate impairment in multiple domains of life functioning due to mental illness Smoking at least 10 cigarettes per day History of failed treatment‐facilitated quit attempts Voluntary informed consent for participation Exclusion criteria: Current use of e‐cigarettes Medical instability Primary diagnosis of dementia or significant cognitive impairment defined as a Mini‐Mental Status Examination (MMSE) score < 24 Inclusion based on specific population characteristics: psychiatrically stable, in‐treatment, people who smoke, with a schizophrenia spectrum disorder or bipolar disorder 68% women; mean age 42; mean cpd: only cigarettes per week reported: 192 (SD = 159.3). This would be an average of 27 cpd; mean FTND 5.5 Motivated to quit: “None of the participants was actively engaged in a quit attempt during the study”. E‐cigarette use at baseline: E‐cig use was an exclusion criterion.	
Interventions	**EC: Cig‐a‐like** E‐cigarette details: (NJOY brand) based on each participant's level of use of combustible tobacco. Each e‐cigarette cartridge was approximately equivalent to 2 packs of combustible cigarettes. Trained research interviewers instructed participants on the proper use of e‐cigarettes.	
Outcomes	Week 1, 2, 3, 4 Adverse events and biomarkers: Breath CO level Possible side effects Other outcomes measured: Use of tobacco products Fagerström nicotine dependence scores Appeal of EC Level of enjoyment of EC Satisfaction with EC compared with usual combustible tobacco Willingness to purchase EC	
Study funding	“Financial support to purchase the e‐cigarettes and pay small stipends to the participants in this unfunded pilot study came from Dr. Mary Brunette's discretionary reserve account.”	
Author declarations	“All authors declare that they have no conflicts of interest”.	
Notes		
***Risk of bias***		
**Bias**	**Authors' judgement**	**Support for judgement**
Random sequence generation (selection bias)	High risk	Not randomized
Allocation concealment (selection bias)	High risk	Not randomized
Incomplete outcome data (attrition bias) All outcomes	Low risk	2 dropouts (9.5%) failed to return to clinic. Analysis based on 19 participants
Selective reporting (reporting bias)	Low risk	All expected outcomes reported

**Table:** Pratt 2022

***Study characteristics***		
Methods	Design: RCT Recruitment: clinician referrals, posters/brochures and mailings. After eligibility confirmation, potential participants were invited for an informational meeting, and interested individuals returned to review the consent form and provide written informed consent. Setting: 2 urban mental health agencies (Kentucky and Massachusetts) serving primarily Medicaid beneficiaries with SMI; USA Study start date: 1 March 2017. Study end date: 31 January 2021	
Participants	Total: N = 240 EC = 120 Assessment only = 120 Inclusion criteria: 1) Diagnosis of schizophrenia, schizoaffective disorder, or bipolar disorder, 2) enrolled in services at the research site for a minimum of 3 months, 3) regular smoker (approx. 10 cigarettes for the past 5 years) with a history of at least 1 quit attempt, 4) 18+ years Exclusion criteria: 1) Regular use of EC in the past month, 2) current interest/plan to quit smoking, 3) regular use of NRT or bupropion or varenicline to quit smoking, 4) use of emergency room or hospitalization for psychiatric reasons in the past 30 days, 5) pregnancy, 6) psychiatric instability (hospitalization in the past month), 7) active substance use disorder Female 47.9%; mean age 45.9 (SD 11.9); mean CPD 18.7; mean FTND 6.9 (SD 1.5) E‐cigarette use at baseline: no Motivated to quit: no	
Interventions	EC: **Cartridge** Arm 1: EC The Study Coordinator provided participants with a 2‐week supply of e‐cigarettes (EC) and instructions on their safe use. Per product packaging, each disposable EC provided up to 300 puffs, roughly the equivalent of 20 cigarettes. Participants were given the opportunity to practice using EC before leaving the appointment to ensure proper use. The Study Co‐ordinator also provided brief information on safety (e.g. keeping EC out of the reach of children) and they gave participants additional 2‐week supplies at 2, 4, and 6 weeks. The EC arm was provided with 8 weeks of free ECs based on self‐report of regular tobacco use. Participants assigned in this arm were asked to switch combustible tobacco with ECs. The appeal of EC and health impacts were measured, but the authors were not targeting quitting combustible tobacco or reducing craving. Arm 2: Assessment only (no intervention) EC was given at final FU visit. Following randomization, Study Co‐ordinators provided participants with appointments for follow‐up study visits, asked them to refrain from using ECs, and reminded participants that they would receive a 4‐week supply of ECs at the final follow‐up visit.	
Outcomes	Baseline, 2, 4, 6, 8, 13, and 26 weeks Breath CO was measured by the blinded Research Interviewers at each visit using the Smokerlyzer Breath Carbon Monoxide Monitor (Bedfont Scientific) as a biologic measure of toxin exposure. CO, CPD, nicotine dependence, EC use (EC count), NNAL AEs: Cough, itchy throat, bad throat Stated that the following would be collected in the NCT record, but not yet reported: change in cancer‐related toxin, 4‐(Methylnitrosamino)‐1‐(3‐pyridyl)‐1‐butanol (time frame: baseline, 4 weeks, 8 weeks, 13 weeks, 26 weeks)	
Study funding	The study was funded by the National Institute on Drug Abuse (NIDA, 1R01DA041416) in the United States.	
Author declarations	None declared	
Notes	New to 2022 update	
***Risk of bias***		
**Bias**	**Authors' judgement**	**Support for judgement**
Random sequence generation (selection bias)	Low risk	Quote: “Unblinded Study Coordinator randomly assigned participants within site using an automated program that stratified by diagnosis (schizophrenia vs. bipolar disorder) and amount of daily smoking (> 20 vs. ≤ 20 cigarettes), in blocks of four to assure balance between arms (1:1 ratio).”
Allocation concealment (selection bias)	Low risk	As above, automated programme
Blinding of participants and personnel (performance bias) All outcomes	High risk	Interventions of different intensity. EC vs assessment only (control)
Blinding of outcome assessment (detection bias) All outcomes	Low risk	Quote: "Blinded Research Interviewers”
Incomplete outcome data (attrition bias) All outcomes	Low risk	240 randomized participants. Amongst those participants, 210 (87.5%) were assessed at 8 weeks, and 214 (89.2%) were assessed at 26 weeks.
Selective reporting (reporting bias)	Unclear risk	Some outcomes not reported here

**Table:** Price 2022

***Study characteristics***		
Methods	Design: single‐arm intervention All CC users offered smoking cessation support and free EC Setting: pharmacy‐supported e‐cigarette smoking cessation intervention delivered by 6 pharmacies (funded by the local authority) Greater Manchester, Northwest England, UK. Socially deprived area Recruitment: advertising conducted through newspapers and in pharmacies, press release, workplace marketing and posters in social housing facilities and by word of mouth. Attendance was incentivized by free equipment and refills. Pharmacies were chosen that had existing skills in delivering smoking cessation services. Study dates: smoking cessation scheme delivered January to June 2019	
Participants	Total N: 871 (single arm) Inclusion criteria: combustible cigarette smoker Exclusion criteria: not stated 57% female Age 18 to 21 (9.3%); 25 to 34 (21.7%); 35 to 44 (17.3%); 45 to 54 (24.8%); 55 to 64 (19%); 65+ (7.9%) Category of smoking: 197/860 lowest (1 to 10); 240/860 medium (1 to 19); 396/860 highest (20+) Motivated to quit: self‐presented assumed interested in quitting/interested in free EC	
Interventions	EC intervention EC, charger, and fluids. All equipment was sourced from a partner e‐cigarette supplier and provided by pharmacies. EC included: bigger Arc Slim variety, which had longer‐lasting batteries and adjustable strength Pharmacies gave practical advice and support on smoking cessation and e‐cigarette use, which was available throughout the 3‐month programme. The pharmacy consultations were seen as an opportunity to give tailored advice to service users and support and encourage them during the quitting process. The use of a carbon monoxide monitor at the pharmacies was described as a useful way of motivating service users to quit.	
Outcomes	Baseline, 2 weeks and 4 weeks (endpoint), 3 months, 12 months Self‐report quit at 12 months. CO confirmed quit 4 weeks. Participants asked to report back to the pharmacy after 4 weeks, 3 months, and 12 months. If happy to be contacted at 3 months service users gave their phone number. After 12 months, the borough council sent text messages to all service users with a valid phone number on record and who had provided consent to be contacted. At 12 months, 355 texts successfully delivered and 77 users returned surveys.	
Study funding	Funding for the project was provided by Trafford Metropolitan Borough Council.	
Author declarations	The authors stated that they had no competing interests to declare.	
Notes		
***Risk of bias***		
**Bias**	**Authors' judgement**	**Support for judgement**
Random sequence generation (selection bias)	High risk	Not randomized
Allocation concealment (selection bias)	High risk	Uncontrolled
Incomplete outcome data (attrition bias) All outcomes	High risk	77/871 at 12‐month follow‐up
Selective reporting (reporting bias)	Unclear risk	No protocol/clinical record

**Table:** Pulvers 2018

***Study characteristics***		
Methods	Design: observational, uncontrolled, experimental study Recruitment: community Setting: visits took place in university labs, USA Study start date: January 2015; Study end date: April 2015	
Participants	Total N: 40 Inclusion criteria: ≥ 18 years; cigarette smoking ≥ 4 days of the past 30 days for at least 1 year; never using EC regularly (less than 25 lifetime uses); not having used EC on ≥ 3 of the past 30 days; willing to switch from smoking regular cigarettes to ECs; fluency in English; regular access to a telephone and transportation to attend appointments; willing to abstain from using marijuana during the study Exclusion criteria: any use of other tobacco products (OTPs) including smokeless tobacco, cigarillos, pipes, cigars, hand‐rolled cigarettes, and hookah in the past 30 days; currently in a smoking cessation programme or another clinical trial; past 30‐day use of NRT or medication which aids smoking cessation including bupropion, clonidine, nortriptyline, or varenicline; uncontrolled asthma, severe allergies, or diabetes mellitus; taking prescription medication for emotional distress, depression, or other psychological problems; current dependence on a substance other than nicotine; presence of any cardiovascular or pulmonary illnesses in the past 6 months; pregnancy Inclusion based on specific population characteristics: no 27% women; mean age 30.08; mean cpd 8.76; FTND not reported Motivated to quit: over half either did not intend to quit at all or did not intend to quit in the next 6 months 22/40 (55%). E‐cigarette use at baseline: inclusion criteria included the following: Never using EC regularly (less than 25 lifetime uses) Not having used EC for more than 3 of the past 30 days	
Interventions	**EC: Refillable** 2nd generation EC starter kit with 2 e‐Go C batteries (3.7 volts/650 MaH), a USB connection cord, an AC adapter, and a carrying case, and a supply of Saturn V4i atomizers (2.4 ohms) filled with liquid in their preferred flavour (28 atomizers total; 2/day). Provided 24 mg/mL dosage vegetable glycerin liquid in a tester sample to all participants. Those who reported the 24 mg was too strong were provided 12 mg/mL dosage liquid. The first session included brief education, training, action planning for making a complete switch to EC. A referral to the California Smokers’ Helpline was made at the final visit (week 4).	
Outcomes	3 lab visits (baseline, week 2, and week 4) and 2 phone visits (week 1 and week 3). Biological samples were taken at all 3 in‐person visits (baseline, week 2, and week 4). However, due to budgetary restrictions, only the baseline and week 4 biological data were analyzed. Adverse events and biomarkers: Biochemical measures only: breath samples were taken with a Micro + (Bedfont, Haddonfield, NJ) to measure CO Urine samples taken to test for change in tobacco toxicant exposure by the following measures: Concentrations of NNAL measured by liquid chromatography–tandem mass spectrometry (LC–MS/MS) Metabolites of a panel of potentially toxic VOCs, including benzene (PMA), ethylene oxide (HEMA), N‐nitrosodimethylamine (MMA), acrylonitrile (CNEMA), acrolein(3‐HPMA), propylene oxide (2‐HPMA), acrylamide (AAMA), and crotonaldehyde (HPMMA) measured by LC–MS/MS, 2 Other outcomes measured: Cotinine, change in tobacco consumption (cpd using TLFB interview), change in frequency of EC use, change in nicotine dependence and attitudes/behaviour, change in 30‐day nicotine exposure	
Study funding	“This study was funded by the University of Minnesota (JSA), P30 DA012393 (NLB), P50 CA180890 (NLB), and California State University San Marcos (KP).”	
Author declarations	“Benowitz is a consultant to pharmaceutical companies that market smoking cessation medications and has been an expert witness in litigation against tobacco companies. The other authors have no conflicts of interest.”	
Notes	New for 2020 update	
***Risk of bias***		
**Bias**	**Authors' judgement**	**Support for judgement**
Random sequence generation (selection bias)	High risk	Not randomized
Allocation concealment (selection bias)	High risk	Not randomized
Incomplete outcome data (attrition bias) All outcomes	Low risk	37/40 provided follow‐up data.
Selective reporting (reporting bias)	Low risk	All outcomes reported

**Table:** Pulvers 2020

***Study characteristics***		
Methods	Design: RCT, unblinded, 2:1 ratio Recruitment: Participants were recruited from the San Diego, California, and Kansas City, Missouri and Kansas, metropolitan areas. Setting: USA Study start date: May 2018. Study end date: May 2019	
Participants	Total N = 186; Electronic cigarettes = 125. Own brand cigarette = 61 40.3% female; mean age 43.3 (SD 12.5); mean cpd 12.1 (SD 7.2). E‐cigarettes use at baseline: 0.05 (0.3%) Inclusion criteria: > 21 years of age; smoked cigarettes on > 25 of past 30 days; smoked > 5 cpd on days smoked; smoked cigarettes > 6 months; carbon monoxide > 5 PPM at baseline; systolic BP of < 160 mmHg and diastolic BP of < 105 mmHg at baseline; Hispanic/Latino or African‐American/black; fluent in English or Spanish; willing to switch from smoking cigarettes to ECs for 6 weeks; regular access to telephone; transportation to attend appointments (KC Only). Exclusion criteria: primary use of other tobacco products or equal use of cigarettes and other tobacco products; EC use on > 4 of the past 30 days; currently in a smoking cessation programme or another clinical trial; use of NRT or medication which aids smoking cessation in the past 30 days; hospitalization for a psychiatric issue in the past 30 days; heart‐related event in the past 30 days (e.g. heart attack, stroke, severe angina (i.e. chest pain), ischaemic heart disease, and vascular disease); uncontrolled blood pressure; planning to move out of study centres (San Diego or Kansas City) in the next 6 weeks; another person in the household enrolled in the study; pregnancy/breastfeeding; unstable mental status or health status	
Interventions	**EC: pod** **Electronic‐cigarettes**: JUUL (5% nicotine); choice of flavours (Menthol, Mango, Cool Mint, Virginia Tobacco); given 1 pod per pack of cigarettes; given a 2‐week supply at baseline and then a further 4‐week supply at week‐2 visit. At each follow‐up appointment (week 1, telephone call; week 2, in‐person visit; and week 4, telephone call), barriers and benefits of switching to e‐cigarettes were discussed and action planning for exclusive switching was revisited. Compensated on a schedule of USD 20 at baseline, USD 40 at week 2 and USD 60 at week 6 **Own brand cigarettes**: compensated on a schedule of USD 20 at baseline, USD 40 at week 2, and USD 60 at week 6	
Outcomes	Baseline, week 2 and week 6. Telephone survey at 6 months Change in past 7‐day combustible cigarette use measured by 7‐day timeline follow‐back interview 30‐day point prevalence at 6 months (EC group only) Reduction in toxicant exposure, as measured by NNAL excretion Cotinine CO Lung function; pulmonary function test of small airway disease that is most sensitive to the effects of cigarette smoking; mean mid‐expiratory phase of forced expiratory (FEF 25% to 75%); respiratory symptoms as measured with the American Thoracic Society Questionnaire (scores range from 0 to 32, with higher scores indicating greater respiratory symptoms) Blood pressure Adverse events: respiratory symptoms	
Study funding	Drs Pulvers and Nollen and Ms Rice were supported by grant No. 5SC3GM122628 from the National Institutes of Health (NIH). Drs Schmid and Ahluwalia were supported in part by grant No. P20GM130414, from the NIH‐funded Center of Biomedical Research Excellence (COBRE). Dr Schmid was partially supported by Institutional Development Award No. U54GM115677 from the National Institute of General Medical Sciences of the NIH, which funds Advance Clinical and Translational Research (Advance‐CTR).	
Author declarations	Dr Schmid reported serving as a consultant for legal firms representing Eli Lilly, Boehringer‐Ingelheim, and Gilead outside the submitted work. Dr Benowitz reported receiving personal fees from Pfizer and Achieve Life Sciences and serving as a consultant to pharmaceutical companies that market smoking cessation medications and as an expert witness in litigation against tobacco companies outside the submitted work. Dr Ahluwalia reported receiving personal fees from Lucy Goods outside the submitted work. No other disclosures were reported.	
Notes	Additional data provided by the authors	
***Risk of bias***		
**Bias**	**Authors' judgement**	**Support for judgement**
Random sequence generation (selection bias)	Low risk	Randomization sequence was generated with an Excel (Microsoft) random number formula applied to each site (2:1 ratio).
Allocation concealment (selection bias)	Low risk	Allocation was placed into sealed individual envelopes labelled with participant identification numbers for each site, retrieved from a locked cabinet monitored by the project manager, and opened individually following consent of each participant.
Blinding of participants and personnel (performance bias) All outcomes	High risk	Could not be blinded
Blinding of outcome assessment (detection bias) All outcomes	Low risk	Carbon monoxide validation
Incomplete outcome data (attrition bias) All outcomes	Low risk	E‐cig: 115/126 Own brand: 54/61
Selective reporting (reporting bias)	Low risk	Per‐protocol reporting

**Table:** Rose 2023*

***Study characteristics***		
Methods	Design: randomized 2 × 2 factorial design; 'switching study' Design changed mid‐study to: nicotine EC + nicotine skin patch vs no nicotine EC + nicotine skin patch Recruitment: "Participants were recruited from North Carolina" Setting: North Carolina, USA	
Participants	Total N: 94 Placebo pod/Placebo patch arm n = 13; Nicotine pod/Placebo patch arm n = 9; Placebo pod/Nicotine patch arm n = 25; Nicotine pod/Nicotine patch arm n = 29 Study changed to 2 arms: Nicotine EC + nicotine skin patch vs No Nicotine EC + nicotine skin patch Inclusion criteria: having smoked for the past year; currently smoking at least 10 cigarettes/day; age of 21 to 65 years; baseline expired air carbon monoxide (CO) reading of at least 10 ppm, body weight Exclusion criteria: a number of major medical conditions; illicit drug use or alcohol abuse/dependence; pregnancy; prior adverse reactions to nicotine patch; use of smoking cessation medications within 30 days; use of tobacco products other than cigarettes within 7 days; or anyone seeking treatment for nicotine dependence 40.4% women; mean age 47.2 (SD 10.3); mean cpd 18.5 (SD 7.3); mean FTND 5.9 (SD 2); mean CO 28.9 ppm (SD14.2) E‐cigarette use at baseline: exclusion criteria use of tobacco products other than cigarettes within 7 days Motivated to quit: not reported	
Interventions	At start of study 4 groups: ENDS (with vs without nicotine); skin patch (with vs without nicotine). Later in the study, all participants were provided with nicotine skin patches and randomized to ENDS with vs without nicotine. Arms after study change (all participants were provided with nicotine skin patch) Arm 1: Nicotine EC + nicotine skin patch JUUL e‐cigarette, a breath‐actuated, rechargeable closed e‐cigarette system. Each pod was pre‐filled with 0.7 mL of e‐liquid, comprising glycerol, propylene glycol, benzoic acid, flavour, 5% nicotine by weight (59 mg/mL). Nicotine skin patch: Nicoderm (GlaxoSmithKline, Philadelphia, PA), delivering 21 mg/24 h nicotine purchased from Rejuvenation Labs (Salt Lake City, UT). Arm 2: No Nicotine EC + nicotine skin patch JUUL e‐cigarette, a breath‐actuated, rechargeable closed e‐cigarette system. Each pod was pre‐filled with 0.7 mL of e‐liquid, comprising glycerol, propylene glycol, benzoic acid, flavour, 0% nicotine (placebo). Nicotine skin patch: Nicoderm (GlaxoSmithKline, Philadelphia, PA), delivering 21 mg/24 h nicotine purchased from Rejuvenation Labs (Salt Lake City, UT). ECs were offered in 2 flavours, “Cool Mint” and "Virginia Tobacco".	
Outcomes	Baseline 2, 4, 6, and 8 weeks CO measured (in the laboratory, or, after the study went remote, by shipping Bedfont coVita CO breathalyzers to participants with direct observation of CO breath testing through remote televisit (via secure Zoom Videoconference Platform)). Cessation to 8 weeks 6 SAEs reported, all determined by medical review to be unrelated to treatment.	
Study funding	This study was supported by grant 5P50DA027840 from the National Institute on Drug Abuse (NIDA).	
Author declarations	JER discloses research support from Foundation for a Smoke‐Free World, Philip Morris International, Altria, Embera Neuro Therapeutics, Inc., Otsuka Pharmaceutical, JUUL Labs, consulting with Revive pharmaceuticals, and consulting and patent purchase agreements with Philip Morris International. JMD discloses fnancial support from Predictably Human Inc., including funding for research, consulting fees, and equity. Predictably Human Inc. is focused on development of a prescription treatment for smoking and e‐cigarette use.	
Notes	Study renamed from NCT03492463 to Rose 2023 in 2024 update	
***Risk of bias***		
**Bias**	**Authors' judgement**	**Support for judgement**
Random sequence generation (selection bias)	Unclear risk	No detail about randomization process: “participants randomly assigned to receive nicotine vs. placebo pods to use in their ENDS devices, and nicotine vs. placebo skin patches”
Allocation concealment (selection bias)	Unclear risk	As above
Blinding of participants and personnel (performance bias) All outcomes	Low risk	Equally intensive
Blinding of outcome assessment (detection bias) All outcomes	Low risk	CO measured
Incomplete outcome data (attrition bias) All outcomes	Low risk	62/76 at 8 week FU
Selective reporting (reporting bias)	Low risk	All expected outcomes are reported
Other bias	Low risk	Study design changed midway due to COVID‐19 and recruitment issues prioritised two arms, therefore participants were not evenly split between the 2x2 design

**Table:** Russell 2021

***Study characteristics***		
Methods	Design: RCT Setting: London, UK	
Participants	426, 53% M NRT = 141; Myblu plus NSPs group = 145; Myblu plus FBNPs group = 140 Inclusion criteria: established daily cigarette smokers aged 18 years and older were recruited in London, UK	
Interventions	EC type: pod 3 arms: NRT; mybluTM containing nicotine salt e‐liquid pods (NSPs); myblu plus freebase nicotine e‐liquid pods (FBNPs) NRT: over‐the‐counter nicotine replacement therapies (NRTs). Free for 3 months Myblu plus NSPs group: a closed system pod e‐vapour product (mybluTM) containing nicotine salt e‐liquid pods (NSPs). Free for 3 months Myblu plus FBNPs group: a closed system pod e‐vapour product (mybluTM) containing freebase nicotine e‐liquid pods (FBNPs). Free for 3 months Participants of both myblu arms were given a primary device, a backup device, and reimbursement for retail purchases of up to 12 e‐liquid pods (6 packs of x2 pods) per month for 3 months. Participants were encouraged to use their assigned e‐vapour product and to choose and change flavours and nicotine concentrations of their assigned e‐liquid pods as they wished.	
Outcomes	Online surveys, administered at study enrolment and then at 1, 2, 3, and 6‐months post‐enrolment, assessed self‐reported past 30‐day consumption of conventional cigarettes and use of NRTs and assigned e‐vapour products. Self‐reported 6‐month past 30‐day cigarette abstinence rate Reduction in smoking	
Study funding	E‐cigarette/alternative nicotine products industry	
Author declarations	NS	
Notes	Conference abstract	
***Risk of bias***		
**Bias**	**Authors' judgement**	**Support for judgement**
Random sequence generation (selection bias)	Unclear risk	No information provided
Allocation concealment (selection bias)	Unclear risk	No information provided
Blinding of participants and personnel (performance bias) All outcomes	Low risk	Unclear if participants were blinded (conference abstract only) but all participants received active interventions, so performance bias judged unlikely
Blinding of outcome assessment (detection bias) All outcomes	Low risk	Self‐report only but all participants received active interventions, so differential misreport judged unlikely
Incomplete outcome data (attrition bias) All outcomes	Low risk	The 6‐month retention rate was 85.8% in the NRT group, 85.5% in the myblu plus NSPs group, and 73.6% in the myblu plus FBNPs group.
Selective reporting (reporting bias)	Unclear risk	No protocol or clinical trial record available to determine whether all prespecified outcomes were reported

**Table:** Scheibein 2020

***Study characteristics***		
Methods	Design: non‐randomized, single‐arm Recruitment: from supported temporary accommodation service; project workers and support staff identified potential study participants who smoked and wished to quit Setting: Dublin Simon Community, Ireland Study start date: recruitment February 2019 (overall trial start date March 2018). Study end date: June 2019	
Participants	Total N: 23 but only report baseline for the 9 that completed the study. % female 8.7% (2/23) at baseline, (22.2% 2/9) completed and reported; mean age 43.89 (SD 7.36); mean cpd 25.22 (SD 7.77); mean FTND 7.89 (SD 1.2); mean CO 21.89 (SD 14.4 corresp) E‐cigarettes use at baseline: no Motivated to quit: yes Inclusion criteria: > 5 CO ppm (carbon monoxide); active smoking status; expressed intention to quit using ENDS‐device Exclusion criteria: self‐reported pregnancy; exhibition of florid psychotic or substance‐use‐related symptoms which could have affected ability to consent	
Interventions	**EC: Refillable** Electronic‐cigarette: Endura T22e Electronic Nicotine Delivery System and 2 10 mL bottles of fluid strengths (0, 6, 11, 18, and 20 mg/mL) and flavours ('Purple Berry', 'Ice Menthol', 'Regular Blend', and 'American Tobacco')	
Outcomes	Baseline (‘week 1’), week 4, week 8, week 12: CO, adverse events Also number of cigarettes smoked; Fagerström Test Scores	
Study funding	This study was completed as part of a Tobacco Harm Reduction Scholarship funded by Knowledge Action Change.	
Author declarations	FS was a recipient of a Tobacco Harm Reduction Scholarship provided by Knowledge Action Change. He is currently the recipient of an Enhanced Scholarship from the same organization. AM and KM acted as mentors for both the Tobacco Harm Reduction Scholarship and Enhanced Scholarship. AM is an associate of New Nicotine Alliance. KM is a recipient of a grant from the Foundation for a Smoke Free World. JW declares no interests. WR declares no interests.	
Notes		
***Risk of bias***		
**Bias**	**Authors' judgement**	**Support for judgement**
Random sequence generation (selection bias)	High risk	Only 1 arm
Allocation concealment (selection bias)	High risk	Not randomized
Blinding of participants and personnel (performance bias) All outcomes	High risk	Not randomized
Blinding of outcome assessment (detection bias) All outcomes	High risk	Self‐reported adverse events
Incomplete outcome data (attrition bias) All outcomes	High risk	9/23 completed. Reason was many people moved away so not linked to unacceptability of the study. Incomplete paperwork to enable it to be followed
Selective reporting (reporting bias)	Unclear risk	Protocol published afterwards

**Table:** Skelton 2022

***Study characteristics***		
Methods	Design: RCT Recruitment: clients of AOD (alcohol or drug) centre. Informed of study and given PIS. Interested clients were telephoned by RA. Setting: 2 AOD clinical programmes, an opiate agonist treatment (i.e. methadone or buprenorphine) programme and cannabis clinic (behavioural treatment for cannabis misuse and cannabis use disorder) located within one local health district service in New South Wales, Australia Study start date: April 2018. Study end date: July 2019	
Participants	Total N: 66 (67 in flow diagram) EC abrupt CC cessation = 30 (flow diagram 32) EC gradual CC cessation = 30 (flow diagram 35) Inclusion criteria: 1) Client of participating HNELHD Alcohol Or Drug (AOD) programme, 2) 18+ years, 3) daily tobacco smoker, 4) interested in making a serious quit attempt in the next 30 days, 5) has not used an END containing nicotine in past month Exclusion criteria: pregnancy or breastfeeding Female 26.9%; mean age 42.3 (SD 8); mean CPD 22 (SD 14.2) Motivated to quit: yes E‐cigarette use at baseline: no	
Interventions	EC: **Refillable** For both arms: All study participants received T22 and T18 starter kits (both Innokin Endura®). The T22 kit had a 1.5 O atomizer, 2000 mAh battery, and 4.1 mL tank. The T18 kit had a 1.5 O atomizer, 1000 mAh battery, and 2.5 mL tank. Both kits included an additional atomizer and micro USB cable. An additional 5 atomizers were provided with the starter kits as replacements. A prescription for 12 mg/10 mL nicotine e‐liquid was provided for all study participants. Participants received a total of 24 bottles (8 bottles per month). At weeks 3 and 7, participants were provided with their next supply of e‐liquid nicotine by either post or face‐to‐face at the AOD programme for which they were recruited. Training day to learn how to use the VNP devices Arm 1: EC abrupt CC cessation Arm 2: EC gradual CC cessation At their training day, participants were provided with a personalized gradual cessation schedule based on the baseline number of cigarettes smoked per day recorded in the baseline survey. Participants were told their quit date was 4 weeks hence. Participants were instructed to reduce the number of cigarettes smoked by 25% at week 1, 50% at week 2, 75% at week 3, and 100% at week 4. Safety was monitored: All participants were briefly contacted to complete safety check‐ins by telephone at weeks 1, 3, 5, 7, and 10 following their training day.	
Outcomes	Baseline. Training day. 6 weeks post‐training day. 12 weeks post‐training day Continuous abstinence Seven‐day point prevalence abstinence, biochemically verified by CO breath test: ≤ 8 ppm Feasibility outcomes – acceptability, quit type preference, adherence, CPD Safety: not reported	
Study funding	We acknowledge HMRI and Hunter New England Local Health District for the present funding, as well as the participating trial sites staff who notified present clients about the study.	
Author declarations	The authors declared that they had no known competing financial interests or personal relationships that could have appeared to influence the work reported in this paper.	
Notes	Safety data not reported. No outcomes to extract New to 2022 update	
***Risk of bias***		
**Bias**	**Authors' judgement**	**Support for judgement**
Random sequence generation (selection bias)	Low risk	Quote: “The randomisation sequence (1:1 in blocks of 4 or 6, stratified by AOD program) was generated by the study statistician using SAS software”.
Allocation concealment (selection bias)	Low risk	Quote: "generated by the study statistician using SAS software and were uploaded into REDcap"
Blinding of participants and personnel (performance bias) All outcomes	Low risk	Both arms received the intervention.
Blinding of outcome assessment (detection bias) All outcomes	Low risk	Quote: “investigators will be blinded for outcome assessments”.
Incomplete outcome data (attrition bias) All outcomes	Low risk	Arm 1: 25/31. Arm 2: 27/35
Selective reporting (reporting bias)	High risk	Not all protocol‐defined outcomes reported

**Table:** Smith 2020

***Study characteristics***		
Methods	Design: double‐blind randomized controlled trial Recruitment: recruited from the local area via advertising on Craigs List social media Setting: laboratory and electronic diaries, USA Study start date/Study end date: not specified	
Participants	Total N: 30 N per arm: PG/VG ratio 70/30 = NR; PG/VG ratio 50/50 = NR; PG/VG ratio 0/100 = NR Inclusion criteria: adults aged ≥ 18 years who have been smoking at least 5 cigarettes daily for the past year (expired CO > 8); usual brand is non‐menthol; use of ENDS on 5 or fewer lifetime occasions; regular use of email or smartphone ownership with capacity to receive SMS text and internet access (necessary for electronic diaries) Exclusion criteria: unwilling to use ENDS/EC as part of the trial; use of smokeless, hookah, or tobacco products other than cigarettes ≥ 10 days in the past 30 days; pregnancy/breastfeeding; recent history of cardiovascular distress in the last 3 months (arrhythmia, heart attack, stroke, uncontrolled hypertension); current use of cessation medications; another household member currently enrolled in the study. 30% women; mean age 43.7; mean cpd 18.5; mean FTND 5.4 Motivated to quit: not specified E‐cigarette use at baseline: participants had used an e‐cigarette an average of 1.6 times in their life, and no one reported use in the last 30 days.	
Interventions	**EC: Cig‐a‐like** EC provided for 1 week. All aspects of the ENDS device and e‐liquid were held constant between groups with the exception of PG/VG ratio: **PG/VG ratio 70/30; PG/VG ratio 50/50; PG/VG ratio 0/100.** Ego‐T 1100 mAh battery and disposable cartomizers (510 Smoketech, 1.5‐Ω dual coil). E‐liquid was tobacco‐flavoured (Classic Tobacco, American E‐liquid) and contained 18 mg/mL of nicotine.	
Outcomes	1 week; 2 lab visits pre and post and participant diaries Adverse events and biomarkers: participants provided a CO sample at each visit Other outcomes measured: cpd, ENDS puffs	
Study funding	Funding for this project was provided by pilot funding from the National Cancer Institute (P01CA200512 to K.M.C.). Salary support provided by the National Institute on Drug Abuse (K12DA031794 to T.T.S., K23DA041616 to B.W.H.)	
Author declarations	M.J.C. has received consulting honoraria from Pfizer. K.M.C. has received payment as a consultant to Pfizer, Inc., for service on an external advisory panel to assess ways to improve smoking cessation delivery in healthcare settings. He also has served as paid expert witness in litigation filed against the tobacco industry.	
Notes	Additional data provided from authors. New for 2020 update	
***Risk of bias***		
**Bias**	**Authors' judgement**	**Support for judgement**
Random sequence generation (selection bias)	Unclear risk	Quote: “At the conclusion of the lab visit, participants were randomized and assigned to take home one of the three e‐liquids to use at home for a 1‐week sampling period (10 participants/ratio).” Quote: “Participants were randomly assigned to receive one e‐liquid to take home for 1 week.” (no further detail given).
Allocation concealment (selection bias)	Unclear risk	Not specified
Blinding of participants and personnel (performance bias) All outcomes	Low risk	Quote: “PG/VG ratio was blinded from participant and staff members who conducted experimental sessions.”
Blinding of outcome assessment (detection bias) All outcomes	Low risk	Biochemical validation
Incomplete outcome data (attrition bias) All outcomes	Low risk	Number of participants at follow‐up not reported, but this may be due to the 1‐week follow‐up, and it seems that all participants (excluding 1 participant who was not randomized) were followed up.
Selective reporting (reporting bias)	Unclear risk	No protocol. Few details for CO measurements, just percentage change for each group, but mean CO data provided by author on request

**Table:** Stein 2016

***Study characteristics***		
Methods	Design: non‐controlled, open‐label, experimental study Recruitment: a flyer posted at a large methadone maintenance treatment programme Setting: methadone maintenance treatment programme, USA Study start date: April 2015; study end date: not specified	
Participants	Total N: 12 Inclusion criteria: current moderate or heavy cigarette use (10+ cpd for at least 12 months prior to enrolment); current MMT for at least 3 months; ready to make a smoking quit attempt in the next 14 days; plan to remain on MMT for at least 12 weeks Exclusion criteria: used EC on ≥ 2 of the past 30 days; currently used medications that may reduce smoking (bupropion, varenicline, NRT); had unstable medical or psychiatric conditions (past month suicidal ideation or past year suicide attempt, hospitalization for myocardial infarction or stroke in the prior 3 months); had regular use of marijuana (self‐report or positive urine drug test) Inclusion based on specific population characteristics: people receiving MMT for opoid use disorder 50% women; mean age 45.9; mean cpd 17.8; mean FTND: not reported Motivated to quit: yes E‐cigarette use at baseline: had not used e‐cigarettes for more than 2 of the past 30 days	
Interventions	**EC: Cig‐a‐like** 2‐week supply of NJOY e‐cigarettes at week 1 (quit day), consisting of 5 packs of NJOY e‐cigarettes (15 in total). Participants could request an additional 5 packs (20 in total) for the following 2‐week study period, if they ran out before a study visit. Participants instructed to use EC exclusively for a total of 6 weeks (end of treatment). They were referred to the state telephone QuitLine for supportive counselling at the quit‐day visit (week 1)	
Outcomes	Participants quit and received e‐cigs at week 1. Assessments were carried out at week 3, 5, 7, and 9. Adverse events and biomarkers: “Side effects” of e‐cigarettes were recorded. Side effects were rated none, slight, mild, moderate, and severe at every assessment visit. An adverse effect possibly related to e‐cigarette use was defined as positive if the value at baseline was either none or slight AND the value at any of 3, 5, or 7 weeks was mild or more severe. Other outcomes measured: Reduction in the average cpd E‐cig adherence Nicotine withdrawal	
Study funding	“MDS is a recipient of National Institute on Drug Abuse Award K24 DA000512. This award funded the project described here.”	
Author declarations	“None declared”	
Notes		
***Risk of bias***		
**Bias**	**Authors' judgement**	**Support for judgement**
Random sequence generation (selection bias)	High risk	No randomization
Allocation concealment (selection bias)	High risk	No randomization
Incomplete outcome data (attrition bias) All outcomes	Low risk	Quote: “One individual dropped out after week 3 and did not return; another completed all follow‐up assessments except week 7.”
Selective reporting (reporting bias)	Low risk	All expected outcomes reported

**Table:** Strasser 2016

***Study characteristics***		
Methods	Design: randomized, factorial trial (participants were randomized to one of the 5 brands of e‐cigarettes – although only 4 brands analyzed) Recruitment: media ads Setting: recruitment from the community; study took place at university, USA Study start date/study end date: not specified	
Participants	Total N: analysis based on 24 (28 originally recruited, but the first 4 participants enrolled experienced malfunctioning NJOY e‐cigs and withdrew – the project was removed from the market before the 5th participant was randomized). N per arm: Blu: 6; Green Smoke: 6; V2: 6; White Cloud: 6 Inclusion criteria: age 18 to 65 and self‐reported smoking at least 10 cigarettes per day Exclusion criteria: use of other tobacco or nicotine‐containing products, including e‐cigarettes (no more than 3 previous episodes of use and not currently using); current diagnosis or evidence of substance abuse or dependence or major depression; current or history of psychotic or bipolar disorder; history of suicide attempt; history of cancer or cardiovascular disease; uncontrolled hypertension; use of smoking cessation medications; current plans to try to quit smoking; pregnancy or lactation Inclusion based on specific population characteristics: not applicable 29% women; mean age 43.3; mean cpd 17; mean FTND 3.7 Motivated to quit: Participants had no current plans to try to quit smoking (eligibility criterion). E‐cigarette use at baseline: no more than 3 previous episodes of use and not currently using (eligibility criterion)	
Interventions	**EC: Cig‐a‐like** All participants received nicotine EC and were instructed to use them exclusively for 9 days. The 5 brands selected, including brand‐reported nicotine levels, were: (1) NJOY (18 mg nicotine) – this brand was discontinued and not analyzed as the e‐cigs provided malfunctioned; (2) V2, 18 mg nicotine; (3) Green Smoke, 18.9 to 20.7 mg nicotine; (4) Blu, 20 to 24 mg nicotine; and (5) White Cloud, 23 to 24 mg nicotine. Each brand advertised the delivery of the same level of nicotine (appropriate for about a pack/day smoker), provided the standard tobacco flavour (no other flavours made available), and used a disposable cigarette‐like device.	
Outcomes	Day 10 was the only testing point of interest for this review, but participants were also tested at days 1 and 5. Adverse events and biomarkers: Breath CO Direct effects of nicotine (e.g. dizziness, nausea, headache) ‐ visual analogue scale with a single word scored from 0 (not at all) to 100 (extremely). Total scores were summed such that higher scores indicated negative responses. Other outcomes measured: E‐cigarette use Direct effects of the e‐cigarette (e.g. satisfying, calming, pleasant, smoke another right now) ‐ visual analogue scale with a single word scored from 0 (not at all) to 100 (extremely). Total scores were summed such that higher scores indicated positive responses. cotinine Withdrawal and craving	
Study funding	“National Cancer Institute (NCI) of the National Institutes of Health (NIH) and FDA Center for Tobacco Products (CTP) under Award Number P50CA179546, as well as grants from the National Cancer Institute (P50 CA143187, P30 CA16520, and P30 DA12393)”	
Author declarations	“Dr Benowitz has served on scientific advisory boards for Pfizer and GlaxoSmithKline related to smoking cessation medications and has been an expert witness in litigation against tobacco companies. Dr Schnoll receives medication and placebo free of charge from Pfizer and has provided consultation to Pfizer and GlaxoSmithKline. These companies had no involvement in this study. Dr Strasser has received funding through the Pfizer GRAND programme, an independent peer‐reviewed grant programme funded through Pfizer (2008‐2011); all investigators have received funding from the United States National Institutes of Health”.	
Notes		
***Risk of bias***		
**Bias**	**Authors' judgement**	**Support for judgement**
Random sequence generation (selection bias)	Unclear risk	Although participants were randomized to different brands of EC, no description on how randomization was carried out
Allocation concealment (selection bias)	Unclear risk	Not specified
Blinding of participants and personnel (performance bias) All outcomes	Unclear risk	No description of whether groups were blind to other conditions, but given similar levels of support between arms, so performance bias judged unlikely
Blinding of outcome assessment (detection bias) All outcomes	Low risk	Unclear whether any blinding took place, some outcomes were measured using objective measures and there was no difference in contact between arms.
Incomplete outcome data (attrition bias) All outcomes	High risk	For blu, Green Smoke, and V2 groups, 83% of participants completed the 10‐day study; only 33% of participants randomized to White Cloud completed the 10‐day study; meaning loss to follow‐up was considerably higher in this group.
Selective reporting (reporting bias)	Low risk	All expected outcomes reported

**Table:** Tattan‐Birch 2023

***Study characteristics***		
Methods	Design: RCT Recruitment: stop‐smoking services in England, free access for smokers trying to quit. Services recruited participants and delivered the intervention during one‐to‐one in‐person counselling sessions with trained stop‐smoking advisors. Setting: 6 stop‐smoking services, England, UK Study start date: April 2019. Study end date: November 2021	
Participants	Total: N 92 Arm 1: E‐cigarette‐varenicline group 48 Arm 2: Varenicline‐only group 44 Inclusion criteria: 1) aged 18+ years, 2) smoker, 3) attending SSS one‐to‐one specialist support in London Local Authorities, 4) firm target quit date, 5) elect to use varenicline to support quit attempt, 6) willing to try e‐cigarettes Exclusion criteria: pregnancy or breastfeeding Female 51%; mean age 43.9 (SD 13.1) E‐cigarette use at baseline: no Motivated to quit: yes	
Interventions	EC **refillable** Arm 1: EC + varenicline EC: a nicotine e‐cigarette starter‐kit, Aspire PockeX e‐cigarette e‐liquid to last for approximately 4 weeks, and an information booklet. Participants could choose a total of eight 10 mL e‐liquid bottles (from Aspire or Totally Wicked) in any combination from a selection of 3 flavours (fruit, menthol, and tobacco) and 3 nicotine concentrations (6, 12, and 18 mg/mL). Participants were encouraged to buy further bottles from local vape shops. Varenicline: same for both arms. Prescribed the standard 12‐week course of varenicline, starting approximately 2 weeks prior to their target quit date. They were advised to take one 0.5 mg pill daily for the first 3 days, then two 0.5 mg pills daily for days 4 to 7, and finally two 1 mg pills daily for the remaining 11 weeks. As this was a pragmatic trial, participants were not asked to avoid using e‐cigarettes. Behavioural support: weekly or fortnightly support until 12 weeks after their quit date. Behavioural support aimed to minimise participants’ motivation to smoke, maximise their motivation to remain abstinent, and guide their use of pharmacotherapy. Arm 2: Varenicline only Same as Arm 1 for varenicline and behavioural support	
Outcomes	Baseline, 9 to 12 weeks Smoking abstinence, self‐reported between weeks 9 and 12 from the target quit date and validated by an expired air CO concentration of below 10 ppm at week 12 Adherence to varenicline During each session, advisors recorded smoking status, exhaled CO, adherence, adverse events, and respiratory symptoms using existing software (QuitManager or PharmOutcomes).	
Study funding	This project was funded by the Global Research Awards for Nicotine Dependence (GRAND) unrestricted research grant programme supported by Pfizer. Additional funding was provided by Cancer Research UK (PRCRPG‐Nov21\100002). All authors are members of the UK Centre for Tobacco and Alcohol Studies (UKCTAS), funded under the auspices of the UK Clinical Research Collaboration (MR/K023195/1).	
Author declarations	LS has received a research grant and honoraria for a talk and travel expenses from manufacturers of smoking cessation medications (Pfizer and Johnson & Johnson). JB has received unrestricted research funding from Pfizer to study smoking cessation. RW has received travel funds and hospitality from, and undertaken research and consultancy for, pharmaceutical companies that manufacture or research products aimed at helping smokers to stop. The other authors have no conflicts of interest to declare. None of the authors have ever received personal fees or research funding of any kind from electronic cigarette or tobacco companies.	
Notes	“The trial was stopped early due to COVID‐19 restrictions and a varenicline recall (92/1266 participants used).” “The evidence is tentative because our sample size was smaller than planned — caused by Coronavirus Disease 2019 (COVID‐19) restrictions and a manufacturing recall. This meant our effect estimates were imprecise, and additional evidence is needed to confirm that providing e‐cigarettes and varenicline together helps more people remain abstinent than varenicline alone.”	
***Risk of bias***		
**Bias**	**Authors' judgement**	**Support for judgement**
Random sequence generation (selection bias)	Low risk	Quote: "Participants were randomised [by] 1:1 ratio in blocks of 6 or 8 participants, stratified by service, using a computer‐generated random sequence with allocation concealed within opaque envelopes. Due to the nature of the intervention, participants and advisors could not be blinded to treatment assignment."
Allocation concealment (selection bias)	Low risk	Quote: "with allocation concealed within opaque envelopes"
Blinding of participants and personnel (performance bias) All outcomes	Low risk	2 active interventions
Blinding of outcome assessment (detection bias) All outcomes	Low risk	CO‐validated smoking abstinence at 6 months following the target quit‐date was measured. Trial stopped at 12 weeks
Incomplete outcome data (attrition bias) All outcomes	High risk	EC + varenicline: 26/48 Varenicline only: 20/44
Selective reporting (reporting bias)	Low risk	Preregistered and all expected outcomes reported (some outcomes not reported, but that was judged due to early termination)

**Table:** Tseng 2016

***Study characteristics***		
Methods	Design: 2‐arm; double‐blind, placebo‐controlled RCT Recruitment: advertisements placed in Craigslist as well as flyers distributed on the street and placed in New York City venues with details for how to contact study staff Setting: community, USA Study start date: July 2014 to 2015 (month unclear); study end date: not specified	
Participants	Total N: 99 (100 were randomized, but 1 participant randomized to the control arm was found to be ineligible between randomization and baseline) N per arm: nicotine EC: 50; placebo EC: 49 Inclusion criteria: age 21 to 35; daily smoker; smoked ≥ 10 cigarettes a day (verified by a CO level of ≥ 8 ppm); interested in reducing cigarette consumption; able to provide consent; had a cell phone and was willing/able to receive text messages and counselling on their cell phone; willing to use an EC for 3 weeks Exclusion criteria: pregnant and/or breastfeeding; history of asthma, other airways diseases, or heart disease; currently using smoking cessation medications (including other forms of NRT, bupropion, or varenicline), or enrolled in a smoking cessation programme or another cessation trial; use of EC in the past 14 days or any other tobacco products (pipe, cigar, cigarillos, snuff, chewing tobacco, rolling tobacco, or hookah/shisha) in the past 30 days; moderate‐to‐severe drug use disorder defined as a score of ≥ 5 on the Drug Abuse Screening Test‐10 and/or a hazardous or active alcohol use disorder defined as at least 7 for men and at least 5 for women on the Alcohol Use Disorders Identification Inclusion based on specific population characteristics: young adults 32.3% women; mean age 28.43; mean cpd 14.33; FTND not measured but time to first cigarette was measured categorically. The mode category was 6 to 30 mins (39/99; 41.5%). Smoking behavioural dependence scale (11 items): mode category ‘Moderate’ (51/99; 51.5%) Motivated to quit: readiness to quit (1 to 10 scale, 1 to 8 apply to current people who smoke): 5.57 ± 1.49 E‐cigarette use at baseline: no use of e‐cigs in past 14 days (eligibility criterion)	
Interventions	**EC: Cig‐a‐like** E‐cigarette details: 3 weeks of disposable 4.5% nicotine NJOY, King Bold (NJOY, Inc, Scottsdale, AZ) which resemble conventional cigarettes. NJOY also manufactured the non‐nicotine placebo EC. Both nicotine and placebo ECs were tobacco‐flavoured. The products were purchased by the investigators and provided to the participants free of charge. Other stop‐smoking pharmacotherapies: none Behavioural support: Prior to receiving the ECs, participants were required to complete a 20‐ to 30‐minute telephone counselling session with a trained tobacco cessation Counsellor. The purpose of the telephone counselling was to review current smoking patterns and offer behavioural and environmental change strategies. These included specific smoking reduction options, such as eliminating cigarettes at work and in the home, carrying only those cigarettes needed for that day, dropping cigarettes associated with less intense triggers first, avoiding smoking triggers, and other strategies to manage urges. 18 participants were asked to reduce the number of cigarettes smoked daily by at least 50% of the total number of cigarettes smoked per day at baseline. To mimic real‐life EC use, minimum EC use instruction was provided. Participants were encouraged to replace cigarettes with as much or as little use of an EC as needed in order to reduce nicotine withdrawal symptoms.	
Outcomes	Week 1, 3 Cessation: not applicable Adverse events and biomarkers: adverse events and symptoms related to EC use Other outcomes measured: Self‐reported reduction of at least 50% in the number of cpd Percentage reduction in number of cpd Use of ECs Satisfaction with ECs	
Study funding	“This work was supported by the National Center for Advancing Translational Sciences at the National Institutes of Health (grant number UL1TR000038).”	
Author declarations	“None declared”	
Notes	Study listed as ongoing study NCT02628964 in the 2016 review update	
***Risk of bias***		
**Bias**	**Authors' judgement**	**Support for judgement**
Random sequence generation (selection bias)	Low risk	Quote: “computer generated”
Allocation concealment (selection bias)	Unclear risk	Quote: “…was concealed from research assistants. Blinding of the allocation of nicotine or placebo EC was ensured by the identical appearance of the ECs”. However, not enough information given on how allocation was concealed at the point of randomization
Blinding of participants and personnel (performance bias) All outcomes	Low risk	Quote: “Blinding of the allocation of nicotine or placebo EC was ensured by the identical appearance of the ECs”.
Blinding of outcome assessment (detection bias) All outcomes	Low risk	Quote: "Blinding of the allocation of nicotine or placebo EC was ensured by the identical appearance of the ECs”.
Incomplete outcome data (attrition bias) All outcomes	Low risk	Nicotine EC lost to follow‐up: 10/50; placebo EC lost to follow‐up: 10/49
Selective reporting (reporting bias)	Low risk	All expected outcomes reported

**Table:** Valentine 2018

***Study characteristics***		
Methods	Design: open‐label prospective cohort study Recruitment: recruited from within the Department of Veterans Affairs (VA) Connecticut Healthcare System by word of mouth Setting: receiving psychiatric services from Department of Veterans Affairs healthcare system, USA Study start date/study end date: not specified	
Participants	Total N: 50 (sample analyzed for primary outcomes on week 1 completers – N = 43) Inclusion criteria: no immediate intention to stop smoking; smoking history of ≥ 5 CPD for the past year Exclusion criteria: current untreated medical or psychiatric or substance use disorders, or both, as determined by a review of the veteran’s electronic medical record; current use of nicotine replacement or other cessation pharmacotherapies; use of e‐cigarettes or smokeless tobacco products for more than 2 of the past 30 days Inclusion based on specific population characteristics: military veteran people who smoke who had no immediate intention to stop smoking and were currently receiving psychiatric services from the Department of Veterans Affairs healthcare system 7% women; mean age 56.9; mean cpd 16.6; mean FTND 4.9 Motivated to quit: had no immediate intention to stop smoking E‐cigarette use at baseline: E‐cigarettes or smokeless tobacco products may have been used for less than 2 of the past 30 days.	
Interventions	**EC: Refillable** All given eVic Supreme (Joyetech), "a commercial, variable‐power, tank‐type device". 6.5 mL tank (Delta 23, Joyetech) and a C3 triple coil atomizer head (Joyetech) with a total resistance of 1.8 ohms. Participants could choose flavour (menthol or tobacco) and nicotine concentration (12 or 24 mg/mL). Participants taught how to use EC, with additional materials dispensed as needed. Participants were informed that they could use the study e‐cigarette or regular tobacco cigarettes, or both, ad libitum during study participation.	
Outcomes	Week 1, 2, 3, 4, 8 (weekly lab visits and 1‐month follow‐up) Adverse events and biomarkers: Alveolar (breath) CO levels (ppm) Other outcomes measured: Number of cpd The frequency of e‐cigarette use (mean days/week) The amount of money spent on combustible cigarettes (US dollars/week) Fagerström Test of Nicotine Dependence Contemplation Ladder E‐cigarette questionnaire (assessed changes in perceptions about e‐cigarettes (e.g. harmfulness, benefits, cost), motivations to use (or not use) them, and the reasons for e‐cigarette or combustible cigarette preferences) (measured at baseline and follow‐up) Cotinine	
Study funding	"This research was supported by the New England Mental Illness Research, Education and Clinical Center and the U.S. Department of Veterans Affairs. Statistical analyses, biochemical assays, and analyses of e‐cigarette solutions were supported by the Administrative and Laboratory cores of P50DA036151 (Yale TCORS) from the National Institutes of Health and the U.S. Food and Drug Administration Center for Tobacco Products. The content is solely the responsibility of the authors and does not necessarily represent the official views of the National Institutes of Health or of the U.S. Food and Drug Administration."	
Author declarations	"Ralitza Gueorguivea, PhD, discloses consulting fees for Palo Alto Health Sciences and Mathematica Policy Research and a provisional patent submission by Yale University: Chekroud, A. M., Gueorguieva, R., & Krystal, K. H. “Treatment Selection for Major Depressive Disorder” (filing date June 3, 2016, USPTO docket number Y0087.70116US00). The authors report no other financial relationships with commercial interests."	
Notes	New for 2020 update	
***Risk of bias***		
**Bias**	**Authors' judgement**	**Support for judgement**
Random sequence generation (selection bias)	High risk	Uncontrolled cohort study
Allocation concealment (selection bias)	High risk	Uncontrolled cohort study
Incomplete outcome data (attrition bias) All outcomes	Unclear risk	Follow‐up: 31/50 at week 8
Selective reporting (reporting bias)	Unclear risk	No protocol or clinical trial record

**Table:** Van Staden 2013*

***Study characteristics***		
Methods	Design: single‐group, within‐subject design Recruitment: participants from a military hospital in South Africa Setting: South Africa Study start date/end date: not specified	
Participants	Total N: 15, mean age 38 years, smoked 20 cpd (range 10 to 30), for an average of 17 years (range 5 to 27) Total N: 13 completed the study (5 women) Inclusion criteria: adults who smoke daily, ≥ 10 cpd Exclusion criteria: history of lung disease Inclusion based on specific population characteristics: no Motivated to quit: not specified E‐cigarette use at baseline: not specified	
Interventions	**EC: Cig‐a‐like** Participants were asked to use an EC only for 2 weeks (i.e. no cigarettes). EC: 'Twisp eGo' cartridge 0.8 mL containing 0.0144 mg of nicotine	
Outcomes	The following measurements were taken at baseline and 2‐week follow‐up: Blood pressure and pulse Arterial and venous COHb and blood oxygen saturation	
Study funding	"We are grateful for the sponsorship of the eGo e‐cigarette packs by Twisp and also for the valuable advice and laboratory assistance given by Col. (Dr) J Lubbe, Chemical Pathologist, 1 Military Hospital, Pretoria with regard to the measurement of the cotinine levels. We also wish to acknowledge Professor Martin Veller for his insightful contributions during the preparation of this manuscript and also Dr Richard van Zyl‐Smith for his assistance and review."	
Author declarations	"The sponsor of the Twisp e‐cigarette had no role in the design and conduction; the collection, analysis and interpretation of the study; or in the preparation, review or approval of the manuscript."	
Notes	Dropouts (N = 2) were due to illness (headache and fever) and undertaking a military course associated with high stress and exposure to others' smoking, making it difficult to abstain from cigarettes. The paper stated that the EC cartridge contained 0.8 mL of solution with 0.0144 mg of nicotine. This would be an unusually low concentration of nicotine, and we have assumed an error in units where milligrams should have been grams (0.0144 grams of nicotine would make the concentration 18 mg/mL).	
***Risk of bias***		
**Bias**	**Authors' judgement**	**Support for judgement**
Random sequence generation (selection bias)	High risk	Prospective cohort
Allocation concealment (selection bias)	High risk	Not randomized
Incomplete outcome data (attrition bias) All outcomes	Low risk	2/15 lost to follow‐up
Selective reporting (reporting bias)	Unclear risk	Unable to determine prespecified outcomes

**Table:** Vickerman 2022

***Study characteristics***		
Methods	Design: RCT Recruitment: Participants were recruited from incoming callers to the Oklahoma Tobacco Helpline (OTH) between November 2018 and March 2020 who were smoking and had used e‐cigarettes in the past 30 days. The OTH is a free, public health resource offering tobacco cessation services via phone, web, text, and/ or print materials, as well as NRT. Setting: Seattle, Washington, USA Study start date: May 2018; study end date: October 2020	
Participants	Total: N = 110 55 in arm 1: enhanced e‐cigarette coaching (EEC) 54 in arm 2: quitline treatment‐as‐usual (TAU) Dual users of EC and combustible cigarettes Inclusion criteria: 1) Requested quitline coaching services and enrolled in 5‐call Oklahoma Tobacco Helpline (OKHL) programme, smoked at ≥ 1 cigarette per day (CPD), 2) using EC or planning to use EC, 3) willing to quit cigarettes, 4) consented to receive automated phone outreach via the quitline, 5) Android smartphone, regular access to email, 6) 18+ years Exclusion criteria: 1) schizophrenia, heart attack, stroke or heart condition in past 2 weeks, 2) taking varenicline or bupropion, 3) pregnancy Female 61.5%; mean age 40.6 (SD 13.5); mean CPD 19.2 (SD 11.3) E‐cigarette use at baseline: yes Motivated to quit: yes	
Interventions	No EC intervention. Advice only Arm 1: Enhanced e‐cigarette coaching (EEC) [Quitline (NRT available) + EC advice (no EC intervention)] The EEC protocol added 4 intervention components to standard evidence‐based quitline treatment: EC education, a shared decision‐making model (SDM) for quit plan development (offering selection of NRT, EC, both NRT and EC, or no nicotine replacement aid; in these conversations, coaches discussed EC as a quitting tool similar to NRT and as an alternative form of nicotine replacement), behavioural support tailored to the selected quit plan, and a requirement for coaches to assess and address EC use on every call. Arm 2: Quitline treatment‐as‐usual (TAU) (NRT available) The TAU protocol included recommending participants stop both cigarettes and e‐cigarettes on their quit date when they start NRT. Both arms: Standard benefits available through the OTH (2 to 8 weeks of patch, gum, and/or lozenge) The quitline intervention focused on 5 key strategies for quitting: committing to a quit date, coping with urges, using medications effectively, disposing of tobacco paraphernalia, and utilizing social support. The 5 coaching calls for the 2 groups were delivered over approx. 2 months, with call 1 intended to take approx. 20 to 25 mins, and later calls to take approx. 15 mins. Participants downloaded a smartphone app to complete daily questionnaires on product use for 12 weeks. Participants received USD 20 for completing the baseline survey and up to USD 110 for daily questionnaires.	
Outcomes	Cessation 3 months (CO confirmed) AEs Continued EC use	
Study funding	This work was supported by the National Institute on Drug Abuse (R21DA042960 to KAV) and used the InsightTM mHealth Platform and Android smartphone app, which was supported by the National Cancer Institute Cancer Center Support Grant P30CA225520 awarded to the University of Oklahoma Stephenson Cancer Center. The content is solely the responsibility of the authors and does not necessarily represent the official views of the National Institutes of Health or Optum.	
Author declarations	KAV, KMC, LNM, JMH, and KAW are employees of Optum, the provider of quitline services for the Oklahoma Helpline in this study. THB is on the Advisory Board of Hava Health, Inc., a start‐up (with no connections to the tobacco industry) that is developing a therapeutic e‐cigarette. All other authors declared no conflicts of interest.	
Notes	New to 2022 update	
***Risk of bias***		
**Bias**	**Authors' judgement**	**Support for judgement**
Random sequence generation (selection bias)	Low risk	Quote: “participants were then randomized to receive quitline treatment as usual (TAU) or EEC, using blocked randomization stratified by gender (male vs female; other response options for gender were not included for the quitline at the time of this trial, but have since been added) with an allocation ratio of 1:1. The quitline participant record software accessed a randomization table to automatically assign a participant to a group after a coach clicked a randomize button".
Allocation concealment (selection bias)	Low risk	As above
Blinding of participants and personnel (performance bias) All outcomes	Unclear risk	No detail
Blinding of outcome assessment (detection bias) All outcomes	Low risk	CO measured
Incomplete outcome data (attrition bias) All outcomes	Low risk	EEC: 34/55 (46) TAU: 32/54 (50)
Selective reporting (reporting bias)	Low risk	Outcomes from NCT record reported

**Table:** Wadia 2016

***Study characteristics***		
Methods	Design: uncontrolled experimental study Recruitment: dental hospital staff were recruited – not specified how Setting: dental hospital, UK Study start date: April 2015; study end date: December 2015	
Participants	Total N: 20 (18 of the 20 attended the reassessment visit) Inclusion criteria: 18 to 65 years; systemically healthy; smoked ≥ 10 CPD for at least 5 years; ≥ 24 natural teeth (excluding third molars) and had no probing pocket depths over 4 mm at any site; did not wish to quit Exclusion criteria: systemic condition known to exacerbate or modulate periodontitis (for example, diabetes); antibiotics in the previous 3 months; anti‐inflammatory drugs or other medication likely to affect the periodontal tissues were taken routinely; pregnancy/breastfeeding Percentage of women in study, age, cpd and FTND: not specified Motivated to quit: enrolled people who smoke who did not intend to quit smoking, but were prepared to attempt to substitute smoking with the use of e‐cigarettes for 2 weeks E‐cigarette use at baseline: not specified	
Interventions	**EC: Refillable** Participants provided with a blu PROTM e‐cigarette kit (Electric Tobacconist^®^), an extra bottle of blu PRO Tobacco™ e‐Liquid (Electric Tobacconist) and written instructions. The e‐Liquid was Classic Tobacco‐flavoured and contained 18 mg of nicotine (medium strength). The participants agreed to substitute their regular smoking habits with the use of e‐cigarettes for 2 weeks. They were asked to make a note of any cigarette smoking during the 2 weeks if complete abstinence was unsuccessful.	
Outcomes	2 weeks Adverse events and biomarkers: adverse effects Other outcomes measured: Cigarette use Dental outcomes	
Study funding	Not specified	
Author declarations	Not specified	
Notes		
***Risk of bias***		
**Bias**	**Authors' judgement**	**Support for judgement**
Random sequence generation (selection bias)	High risk	No randomization
Allocation concealment (selection bias)	High risk	No randomization
Incomplete outcome data (attrition bias) All outcomes	Low risk	2 lost to follow‐up
Selective reporting (reporting bias)	Low risk	All expected outcomes reported

**Table:** Wagener 2023

***Study characteristics***		
Methods	Design: RCT Setting: Ohio State University Comprehensive Cancer Center, USA Recruitment: recruit and enrol recent smoking cessation treatment failures from a state QL Study start date: 15 October 2020. Primary completion date: 31 January 2023.	
Participants	N = 350 EC = 175 Quitline treatment as usual (NRT) = 175 Participants: Quitline treatment failures % female 61.3; age 54.72; CPD 17.10 (8.18) Predominately serves priority populations (e.g. low socioeconomic status, high levels of mental health conditions) Inclusion criteria: smoke ≥ 5 cigarettes per day for the past year; report at least minimal interest in switching to an alternative product (> "not at all" on a Likert scale); participation in the Oklahoma Tobacco Helpline or South Carolina Tobacco Helpline within the last 4 to 7 months Exclusion criteria: < 21 years old; report NRT use or making a quit attempt within the last 7 days; current daily use of an e‐cigarette over last month; unstable or significant medical condition or psychiatric conditions; pregnant/breastfeeding	
Interventions	EC: JUUL pod Experimental: E‐cigarette. 5% nicotine JUUL e‐cigarette (tobacco or menthol flavour). Will also receive counselling Active comparator: Quitline Treatment‐As‐Usual. NRT consisting of nicotine patches and lozenges along with standard assessments. Will also receive counselling. All participants will receive 3 calls from QL coaches and EC and NRT will be provided at no cost for 8 weeks. The final follow‐up will occur 12‐weeks post baseline.	
Outcomes	Baseline, 12 weeks Smoking behaviour, product use patterns and continued use, and carbon monoxide, AEs, SAEs. Self‐reported 7‐day point prevalence abstinence from cigarettes (time frame: daily for 12 weeks), carbon monoxide validated At 12 weeks: Minnesota Nicotine Withdrawal Scale Tiffany‐Drobes Questionnaire of Smoking Urges: Brief Form (QSU) Cigarette/EC Dependency Scale Tobacco Use History Questionnaire‐Years of Smoking Change in motivation rulers Drug effects/liking Questionnaire Cigarette purchase task ‐ breakpoint Change in motivation rulers (scale) from baseline to week 12	
Study funding	Ohio State University Comprehensive Cancer Center National Institute on Drug Abuse (NIDA)	
Author declarations		
Notes		
***Risk of bias***		
**Bias**	**Authors' judgement**	**Support for judgement**
Random sequence generation (selection bias)	Unclear risk	NCT record and SRNT abstract ‐ no detail provided
Allocation concealment (selection bias)	Unclear risk	NCT record and SRNT abstract ‐ no detail provided
Blinding of participants and personnel (performance bias) All outcomes	Unclear risk	NCT record and SRNT abstract ‐ no detail provided
Blinding of outcome assessment (detection bias) All outcomes	Unclear risk	NCT record and SRNT abstract ‐ no detail provided
Incomplete outcome data (attrition bias) All outcomes	Unclear risk	NCT record and SRNT abstract ‐ no detail provided
Selective reporting (reporting bias)	Unclear risk	NCT record and SRNT abstract ‐ no detail provided
Other bias	Unclear risk	NCT record and SRNT abstract ‐ no detail provided

**Table:** Walele 2018*

***Study characteristics***		
Methods	Design: RCT (short‐term, Cravo 2016) followed by cohort study (Walele 2018*) in which all participants were given nicotine EC Recruitment: community Setting: 2 centres in the UK (Covance Clinical Research Unit Ltd, Leeds and Simbec Research Ltd, Wales) Study start date: December 2013; study end date: December 2016	
Participants	20 participants Inclusion criteria differ per study phase: Cravo 2016 (short‐term RCT), inclusion: 21 to 65 years; BMI 18 to 35 kg/m^2^; 5 to 30 CPD for ≥ 1 year (self‐reported); in good health (determined by medical history, a physical examination, a 12‐lead ECG, lung function tests and clinical laboratory evaluations); people who smoke (urinary cotinine ≥ 3 and exhaled CO ≥ 6 ppm) Additional criteria for Walele 2018 (participants from Cravo 2016): Participants assessed by PI as being compliant in Cravo 2016 (e.g. having attended outpatient visits and having been compliant with study procedures) Participants had to be willing to use the study product as the only nicotine‐containing product for the duration of the study, and, as deemed by PI, had to have no clinically significant abnormalities in 12‐lead electrocardiogram, vital signs, spirometry and clinical laboratory assessments in the preceding study. In addition, participants who were assigned to the conventional cigarette (CC arm) in Cravo 2016 had to be established people who smoke CCs, which was assessed by urinary cotinine levels (a score of 3 and above on a NicAlert™ test strip was considered positive), eCO levels (a readout > 6 ppm was considered positive) and by review of a smoking history questionnaire. Exclusion criteria: Cravo 2016: Use of NRT, snuff or chewing tobacco in 14 days previous, or intended to use during study; trying to stop smoking or considering quitting; clinically‐significant illness or disorder, history of drug or alcohol abuse within 2 years prior to study start; woman of “childbearing potential” unwilling to use “acceptable contraceptive measure” during study. Walele 2018 (participants from Cravo 2016): people who had taken or received any form of NRT, snuff or chewing tobacco during the previous study or intended to use it during this study; relevant illness history; history of drug or alcohol abuse; lung function test or vital signs considered unsuitable; trying to stop smoking; women who are pregnant, or unwilling to use acceptable contraceptive method for the duration of the study **Cravo 2016** Total N: 419 randomized, 408 analyzed (excludes 11 who were excluded prior to any product use) N per arm: EVP: 306; control: 102 45% women; mean age 34.6; mean cpd: most 11 to 20 cpd (56% intervention, 62% control); mean FTND: most moderate (57% intervention, 54% control) Motivated to quit: no E‐cigarette use at baseline: not excluded based on prior EC use **Walele 2018** Total N: 209 (147 pre‐EVP group; 62 pre‐CC group) 45% women; mean age 36.6; mean cpd 2.6 (data from figure): not reported; FTND: not reported; motivated to quit: as reported for Cravo 2016 E‐cigarette use at baseline: not reported	
Interventions	**EC: Cig‐a‐like** **Cravo 2016** **EC**: EVP prototype (2.0% nicotine), developed by Fontem Ventures B.V. (Amsterdam, the Netherlands). Instructed to only use EVP for study period. It consisted of a rechargeable battery (voltage range of 3.0e4.2 V), an atomiser and a capsule (small cartridge) containing e‐liquid. The capsules were replaceable and the battery and atomiser were reusable. Could choose between 2 different e‐liquids, which differed solely in their flavour: a menthol‐flavoured e‐liquid with 2.0% nicotine (2.7 mg nicotine/capsule) and a tobacco‐flavoured e‐liquid with 2.0% nicotine (2.7 mg nicotine/capsule) **Control**: used their own usual conventional cigarette brand **Walele 2018** E‐cigarette details: commercially available Puritane™ (closed system EVP) consists of a lithium‐ion rechargeable battery and a replaceable cartomizer comprising of an e‐liquid reservoir pre‐filled by the manufacturer, a heating element and a mouthpiece; 1.6% nicotine (16 mg/g). Available in tobacco or menthol. 2 weeks before baseline, participants had a familiarization session with Puritane™, where they could see and try the EVP.	
Outcomes	**Cravo 2016:** weeks 1, 2, 4, 6, 8, 10, and 12 **Walele 2018:** starting on the last day of the previous trial: months 1, 2, 3, 6, 9, 12, 15, 18, 21, and 24 Study centre visits for assessments Adverse events and biomarkers: “adverse events” (coded using Medical Dictionary for Regulatory Activities version 16.1, 2013, collected via diary cards and questionnaires) Vital signs (systolic and diastolic blood pressure, pulse rate and oral temperature) Lung function (FEV, FEF, PEF) Urine biomarkers (nicotine equivalents (NEQs: nicotine, cotinine, nicotine‐N‐glucuronide, cotinine‐Nglucuronide, trans 3’‐hydroxycotinine and trans 3’‐hydroxycotinine glucuronide); S‐PMA; 3‐HPMA; PG; total NNAL (NNAL þ NNAL‐glucuronide)); exhaled CO Blood COHb Other outcomes measured: Number of conventional cigarettes smoked EVP capsules used ECG (categorized them as normal, abnormal‐not clinically significant (NCS) or abnormal‐clinically significant (CS)) MWS‐R (revised Minnesota Nicotine Withdrawal Scale) QSUBrief (Brief Questionnaire of Smoking Urges) questionnaires Clinical chemistry (blood levels of aspartate aminotransferase (AST), alanine aminotransferase (ALT), alkaline phosphatase, gamma‐glutamyl transferase (GGT), sodium, potassium, chloride, calcium, inorganic phosphate, glucose, urea nitrogen (BUN), total bilirubin, creatinine, total protein, albumin, cholesterol (HDL, LDL, and total)); clinical haematology (white blood cell count (WBC), red blood cell count (RBC), haemoglobin, haematocrit (PCV), mean cell volume (MCV), mean cell haemoglobin (MCH), mean cell haemoglobin concentration (MCHC), platelet count, differential WBC);urinalysis (pH, protein, glucose, ketones, urobilinogen, blood and specific gravity)	
Study funding	**Cravo 2016** "This work was funded and supported by Fontem Ventures B.V. Imperial Brands plc is the parent company of Fontem Ventures B.V. the manufacturer of the EVP prototype used in this study". **Walele 2018** "This work was funded and supported by Fontem Ventures B.V. Imperial Brands Group plc is the parent company of Fontem Ventures B.V., the manufacturer of the EVP used in this study".	
Author declarations	**Cravo 2016** "Dr. Cravo has nothing to disclose. Mrs Martin reports personal fees from Fontem Ventures B.V. during the conduct of the study; personal fees from tobacco and pharmaceutical industries outside the submitted work. Dr. Sharma reports others from Fontem Ventures B.V. during the conduct of the study. Dr. Bush reports others from Fontem Ventures B.V. during the conduct of the study. Mrs Savioz reports personal fees from Fontem Ventures B.V. during the conduct of the study; personal fees from tobacco and pharmaceutical industries outside the submitted work. Mr Craige has nothing to disclose. Mr Walele has nothing to disclose." **Walele 2018** (copied from Transparency documents) "Dr. Koch reports others from Fontem Ventures B.V., during the conduct of the study; Dr. Martin reports personal fees from Fontem Ventures B.V., during the conduct of the study; personal fees from tobacco and pharmaceutical industries, outside the submitted work; Dr. O'Connell has nothing to disclose. Dr. Bush reports others from Fontem Ventures B.V., during the conduct of the study; Dr. Savioz reports personal fees from Fontem Ventures B.V., during the conduct of the study; personal fees from tobacco and pharmaceutical industries, outside the submitted work; Dr. Walele has nothing to disclose."	
Notes	Sponsor: Imperial Tobacco Group PLC Study listed as ongoing studies NCT02029196 and NCT02143310 in 2016 review update. Treated as single study in this review due to including the same participants, and no time lag between studies. "The same subjects who participated in our previous clinical trial (ClinicalTrials.gov, #NCT02029196) conducted in the same centres, with another EVP (Cravo 2016), were invited to participate in the study by Walele 2018*. All volunteering subjects were assigned to switch to using Puritane™, a closed system EVP, for two years, starting on the last day of the previous trial (End of Study [EoS] visit), which corresponded to the baseline visit of Walele 2018*."	
***Risk of bias***		
**Bias**	**Authors' judgement**	**Support for judgement**
Random sequence generation (selection bias)	Low risk	Quote: “Randomisation was performed using an Interactive Web Response System (IWRS; Almac Clinical Technologies)”.
Allocation concealment (selection bias)	Low risk	Quote: "Randomisation was performed using an Interactive Web Response System (IWRS; Almac Clinical Technologies)”.
Blinding of participants and personnel (performance bias) All outcomes	High risk	Open‐label, no blinding, differential levels of support/product use so performance bias cannot be ruled out.
Blinding of outcome assessment (detection bias) All outcomes	High risk	Open‐label, no blinding, with differential levels of support/product use and subjective outcomes
Incomplete outcome data (attrition bias) All outcomes	Low risk	Cravo: 286/306 int and 101/102 control completed study but all who received product included in analysis. In EVP group, 14 withdrew consent, 2 experienced AEs, 1 death, 3 “other”. CC group 1 AE Walele 2018*: High 209/387 enrolled for study Walele 2018*. A total of 102 participants (48.8%; EVP: 75/145 (51%); CC: 27/61 (43.5%)) completed the study.
Selective reporting (reporting bias)	Low risk	Cravo 2016: low All anticipated outcomes reported (study registered prior to study completion) Walele 2018*: low All anticipated outcomes reported (study registered prior to study completion)

**Table:** Walker 2020

***Study characteristics***		
Methods	Design: RCT Recruitment: national media advertising Setting: community‐based, New Zealand Study start date: recruitment between March 2016; study end date: Aug 2018	
Participants	N per arm: patches‐only group: 125; patches plus nicotine e‐cigarette group: 500; patches plus nicotine‐free e‐cigarette group: 499 Inclusion criteria: living in New Zealand; 18 years or older; smoked tobacco (amount not specified); motivated to quit in the next 2 weeks; able to provide verbal consent; prepared to use any of the trial treatments; access to a telephone Exclusion criteria: pregnancy or breastfeeding; used an EC for smoking cessation for ≥ 1 week any time in the past year; currently using smoking cessation medication; enrolled in another cessation programme or study; history of severe allergies; poorly controlled asthma; cardiovascular event in the 2 weeks before enrolment; only 1 participant per household was permitted. 69% women; mean age 41.6; mean cpd 17.3; mean FTND 5.2 Motivated to quit: yes E‐cigarette use at baseline: not reported but use of an e‐cigarette for smoking cessation for more than 1 week any time in the past year was an exclusion criterion.	
Interventions	**EC: Refillable** Moderate‐intensity behavioural support was available for all participants immediately after randomization, then once a week for 6 weeks. This support consisted of 10 to 15 mins of withdrawal‐oriented behavioural support and advice on using their allocated treatment, delivered proactively over the phone by researchers who had received standardized training in delivery of such support. Assigned to: 1) **Nicotine patch** for 14 weeks, including 2‐week prequit. 21 mg, 24‐hr nicotine patch (Habitrol) 2) **Nicotine patch and nicotine‐free EC** for 14 weeks. As 1, plus 14‐week supply at no cost. A 2nd generation eVOD (Kangertech, Shenzhen GuangDong, China) starter kit, with a choice of 1 of 2 tobacco e‐liquid flavours. Advised to start using the e‐cigarette 2 weeks before their quit date, as and when necessary or desired, and in accordance with the manufacturer’s written instructions, to become familiar with its use. Participants were instructed to stop smoking from their quit date and continue with their allocated treatment for 12 weeks (ad libitum use of the e‐cigarette), irrespective of any lapses to smoking. 3) **Nicotine patch and nicotine EC** for 14 weeks. As above, but 18 mg/mL nicotine	
Outcomes	Quit date, 1, 3, 6, and 12 months Continuous abstinence at 6 months with CO validation Adverse events and biomarkers: known side effects associated with e‐cigarette use and nicotine patch use; SAEs Other outcomes measured: Relapse Self‐reported treatment adherence Tobacco withdrawal symptoms and urge to smoke Urge to vape Self‐reported weight Concomitant medication Treatment cross‐over Use of other smoking cessation support or medication Continued use of allocated treatment past 14 weeks Changes in shortness of breath, cough, asthma, COPD, and mental health problems Belief in ability to quit and remain tobacco‐free Smoking identity and views on their allocated treatment for smoking cessation and whether they would recommend it to other people who smoke who want to quit In people still smoking at each follow‐up call, outcomes were number of cigarettes smoked per day and reduction in smoking. Participants allocated e‐cigarettes were asked about their urge to vape; whether they changed devices or e‐liquid, or both; whether they accessed any e‐cigarette support.	
Study funding	Funding: Health Research Council of New Zealand. "The sponsor of the study had no role in the study design, data collection, data analysis, data interpretation, or writing of the report. The corresponding author had full access to all the data in the study and had final responsibility for the decision to submit for publication."	
Author declarations	NW, CB, MV, GL, ML, and VP report grants from the Health Research Council of New Zealand, during the conduct of the study. NW, CB, MV, and VP report grants from Pfizer, outside the submitted work. GL chairs the organization End Smoking New Zealand, which advocates for harm‐reduction approaches to tobacco control. E‐cigarettes were purchased from a New Zealand e‐cigarette online retailer (NZVAPOR, https://www.nzvapor.com/), e‐liquid was purchased from Nicopharm, Australia (https://www.nicopharm.com.au/), and nicotine patches were supplied by the New Zealand Government via their contract with Novartis (Sydney, Australia). NZVAPOR also provided, at no cost to participants, on‐line and phone support regarding use of the e‐cigarettes. Neither NZVAPOR nor Nicopharm have links with the tobacco industry. None of the above parties had any role in the design, conduct, analysis, or interpretation of the trial findings, or writing of this publication.	
Notes	Study listed as ongoing study NCT02521662 in the 2016 review update	
***Risk of bias***		
**Bias**	**Authors' judgement**	**Support for judgement**
Random sequence generation (selection bias)	Low risk	Computer‐generated randomization sequence
Allocation concealment (selection bias)	Low risk	Quote: “We ensured allocation concealment because the statistician who generated the random allocation was not the person randomising participants.”
Blinding of participants and personnel (performance bias) All outcomes	High risk	Quote: “Participants and researchers collecting outcome data were masked to the nicotine content of the e‐liquid” but those allocated to patch only would be aware they did not have an E‐cigarette. Quote: “Third, while we attempted to minimise detection bias by masking the nicotine content of the e‐liquid, we were only 30% successful, and thus some bias in favour of nicotine e‐cigarettes could have occurred.”
Blinding of outcome assessment (detection bias) All outcomes	Low risk	Biochemical validation
Incomplete outcome data (attrition bias) All outcomes	Low risk	< 50% lost to follow‐up, similar rates of attrition between groups (within 20%)
Selective reporting (reporting bias)	Unclear risk	CO‐verified abstinence at 12 months stated as a secondary outcome, but data were not reported in the main text. However, stated in the appendix that too few people in each group were followed up to 12 months (36/1124), so no data were presented for this time point

**Table:** White 2021

***Study characteristics***		
Methods	Design: RCT (2 x 2 x 2 factorial design) Recruitment: Setting: Winston‐Salem, NC, Philadelphia, PA and the respective surrounding areas, USA Study start date: 6 August 2018. Study end date: 13 March 2020	
Participants	Total study N: 50 50 were randomized, 32 completed study Inclusion criteria: Self‐reported smoking 5 to 50 cpd, CO and urinary cotinine confirmed Reported vaping on at least 2 separate occasions, without an allergic or otherwise adverse experience 18+ years Exclusion criteria: Self‐reporting frequent use of e‐cigarettes (> 15 days in the past month) Female 40%; mean age 41.6 (SD 11.7); mean CPD 22.8 (SD 10.5); mean FTDN 5.7 (SD 1.8) E‐cigarette use at baseline: 30% Motivated to quit: NR	
Interventions	EC type: **refillable** EC: Halo Triton (3.7 V battery; 650 mAh) vape pen and compatible Triton 2.4 mL refillable tanks with 2.2‐2.4 ohm coils. E‐liquid, sourced from Syndicate Distribution (Westchase, Florida, US) in 10 mL bottles; had a base of 70% propylene glycol and 30% vegetable glycerin Combustible cigarettes: participants received Spectrum research cigarettes matched to their usual brand cigarette menthol preference. All received study cigarettes and a vape pen with e‐liquid to use for 12 weeks. Study cigarettes (very low nicotine content (0.4 mg/g of tobacco)) Study cigarettes (normal nicotine content (15.8 mg/g of tobacco; double‐blind)) Vape pen ‐ E‐liquids contained low nicotine (0.3% free‐base nicotine) levels of nicotine. Vape pen ‐ E‐liquids contained moderate nicotine (1.8% free‐base nicotine; open‐label). Flavours: 3 tobacco flavours (robust tobacco, light tobacco, tobacco/menthol blend), mint, fruit and dessert flavour options (mixed berry, watermelon, berry, cookies and crème, chocolate, caramel, spearmint, peppermint, and menthol) Arm 1: Normal Nicotine Content (NNC) cigarette + moderate nicotine e‐liquid (1.8% free‐base nicotine) + tobacco flavours Arm 2: NNC cigarette + low nicotine e‐liquid (0.3% free‐base nicotine) + tobacco flavours (0.3% free‐base nicotine) Arm 3: NNC cigarette + moderate nicotine e‐liquid + varied flavours Arm 4: NNC cigarette + low nicotine e‐liquid + varied flavours Arm 5: Very Low Nicotine Content (VLNC) cigarette + moderate nicotine e‐liquid + tobacco flavour Arm 6: VLNC cigarette + low nicotine e‐liquid + tobacco flavours Arm 7: VLNC cigarette + moderate nicotine e‐liquid + varied flavours Arm 8: VLNC cigarette + low nicotine e‐liquid + varied flavours Participants could be compensated up to USD 1070 for completing all study procedures.	
Outcomes	Week 1, 2, 3, 4, 6, 8, 10, 12 CPD during week 12 was the primary outcome. CO up to 12 weeks. Measure of cigarette smoke exposure using data on expired breath CO. CO (ppm) was captured using Covita Smokelyzer devices and standard exhalation procedures. TCD N‐acetyl‐S‐(2‐cyanoethyl) cysteine (CEMA), the mercapturic acid metabolite of acrylonitrile, which serves as a biomarker of cigarette smoke exposure Anatabine levels from the spot urine samples to assess whether participants assigned to VLNC cigarettes adhered to only using VLNC cigarettes Total nicotine equivalents	
Study funding	The research reported in this paper was funded by the National Institute on Drug Abuse and the U.S. Food and Drug Administration (FDA) Center for Tobacco Products (U54DA031659, ECD & DKH). Research reported in this publication was supported by National Institutes of Health (NIH) grant P30 CA77598 utilizing the Biostatistics and Bioinformatics Core shared resource of the Masonic Cancer Center, University of Minnesota and by the National Center for Advancing Translational Sciences of the National Institutes of Health Award Number UL1TR0002494. Author support also included NIDA U54DA036105 (COC) and NIDA R36DA054481 (CMW). The content of this article is solely the responsibility of the authors and does not necessarily represent the official views of the NIH or the FDA.	
Author declarations	Pre‐print. NR	
Notes	Study recruitment ended early due to concerns about pending regulations and the availability/relevance of the study vaping products. New to 2022 review	
***Risk of bias***		
**Bias**	**Authors' judgement**	**Support for judgement**
Random sequence generation (selection bias)	Unclear risk	No detail on how randomization was carried out
Allocation concealment (selection bias)	Unclear risk	No detail
Blinding of participants and personnel (performance bias) All outcomes	Low risk	Cigarette nicotine content was masked to participants and researchers.
Blinding of outcome assessment (detection bias) All outcomes	Low risk	CO measured
Incomplete outcome data (attrition bias) All outcomes	High risk	> 50% dropout in 2 arms At FU: 4/5; 2/6; 4/5; 7/10; 4/7; 5/6; 2/5; 4/6
Selective reporting (reporting bias)	High risk	Outcomes not all reported

**Table:** Xu 2023*

***Study characteristics***		
Methods	Design: "open‐label randomized trial" Recruitment: random sampling from a specially constructed panel assembled using probabilistic address‐based sampling (i.e. by inviting a stratified random sample of all US residential addresses drawn from the US Postal Service’s master file) Setting: general population, USA Study start date September 2018. Study end date October 2019	
Participants	Total N: 837 285 – JUUL Virginia tobacco; 281 – JUUL flavour choice; 271 – quit advice group 50.2% female; mean age 45.99 (SD 11.48); mean CPD 18.76 (SD 7.86) Not motivated to quit. EC use at baseline: lifetime ENDS ever use 62.4%, 63.8%, 62.8%. Ever‐used ENDS fairly regularly 15.6%. Inclusion criteria: (a) age 21 to 65 years; (b) smoked ≥ 100 cigarettes in lifetime; (c) smoke cigarettes daily and smoke ≥ 10 cigarettes/day; (d) willing to attempt to substitute use of ENDS (and JUUL specifically) for some cigarette smoking (“Would you be willing to try using JUUL e‐cigarettes in place of smoking at least some regular cigarettes for 6 months?”) Exclusion criteria: (a) use of ENDS or smoking cessation medications (e.g. NRT, bupropion, varenicline), or behavioural smoking‐cessation counselling in the past 30 days; (b) serious medical or psychiatric condition; (c) living with a study enrollee; (d) participation in another clinical study in the past 30 days; (e) employed by or related to an employee of Juul Labs, Inc. or Pax Labs, Inc.; or (f) pregnancy or breastfeeding	
Interventions	EC arms: JUUL products were provided to JUUL group participants for up to 6 months; a 12‐month follow‐up was completed 6 months later. Participants in the JUUL groups initially received two JUUL devices, a Universal Serial Bus charging dock and the instructions for use included with purchase, as well as 2 to 5 packs of pods (4 pods per pack, 5.0% nicotine by weight (59 mg/mL)), based on baseline cigarette consumption. Participants could order up to 15 four‐packs of pods and additional devices each month for the first 6 months. The Virginia Tobacco group was only provided Virginia Tobacco pods. The flavour choice group could order their preferred flavour(s) available at time of study (Virginia Tobacco, Classic Tobacco, Mint, Menthol, Mango, Creme, Fruit, and Cucumber). Comparator arm: Quit Advice. Printed materials explaining why quitting smoking is important and behavioural advice about how to quit smoking (Centers for Disease Control and Prevention (CDC) and https://Smokefree.gov about reasons to quit smoking and means of cessation) and given contact information for smoking‐cessation services. This was meant to represent basic advice to adults who smoke and were not necessarily ready to quit or seek treatment.	
Outcomes	Baseline, 1, 3, 6, 12 months Cessation at 6 and 12 months AEs and SAEs were not assessed by a medical monitor in this study and were not reported. EC use at FU Flavour use in past 30 days	
Study funding	This study was funded by Juul Labs, Inc.	
Author declarations	Yingying Xu was a full‐time employee of Juul Labs, Inc. when the article was drafted. Nicholas I. Goldenson is a full‐time employee of Juul Labs, Inc. Shivaani Prakash was a full‐time employee of Juul Labs, Inc. when the article was drafted. Erik M. Augustson was a full‐time employee of Juul Labs, Inc. when the article was drafted. Saul Shiffman is a senior advisor to PinneyAssociates, Inc, through which he provides consulting services on tobacco harm reduction on an exclusive basis to Juul Labs, Inc. The Centre for Substance Use Research (CSUR; https://www.csures.com) developed the study instruments and protocol. Yingying Xu, Shivaani Prakash, and Erik M. Augustson was a full‐time employee of Juul Labs, Inc., during the time that this study was conducted and article was drafted.	
Notes	New to 2024 update	
***Risk of bias***		
**Bias**	**Authors' judgement**	**Support for judgement**
Random sequence generation (selection bias)	Low risk	Participants were randomized in equal numbers to the 3 study groups (JUUL Virginia Tobacco vs JUUL flavour choice vs Quit Advice) via a blocked randomization scheme (within 36 strata defined by age, gender, race/ethnicity, and educational attainment, to ensure balanced allocation) using a random number generator in Statistical Package for the Social Sciences.
Allocation concealment (selection bias)	Low risk	Neither participants nor study staff knew the allocation in advance.
Blinding of participants and personnel (performance bias) All outcomes	High risk	Interventions were not equally intensive
Blinding of outcome assessment (detection bias) All outcomes	High risk	Self‐reported
Incomplete outcome data (attrition bias) All outcomes	Low risk	Among randomized participants, 84.7%, 91.8%, 93.0%, and 92.2% completed the 1‐, 3‐, 6‐, and 12‐month FU.
Selective reporting (reporting bias)	Unclear risk	Study not pre‐registered. In the main publication, the study does not break down abstinence data by flavour arm. However, the study authors provided these data to us upon request.

**Table:** Yingst 2020

***Study characteristics***		
Methods	Design: cross‐over study Recruitment: Participants were recruited from people living with HIV/AIDS (PLWHA) (who smoked) seeking care at the Penn State Health HIV Comprehensive Care Program. Setting: USA Study start date: not reported	
Participants	Total N: 17; 41.2% female; mean age 49.1 (SD 8.8); mean cpd 16.9 (SD 7.9); mean CO 22.4 (13.1) E‐cigarettes use at baseline: not reported Motivated to quit: no Inclusion criteria: adult (age ≥ 18 years); smokers (≥ 10 cigarettes daily); not planning to quit smoking; documented history of a positive HIV status Exclusion criteria: not reported	
Interventions	**EC: Cig‐a‐like; Refillable** **Cig‐a‐like device** (Blu), nicotine concentration 24 mg/mL. Propylene glycol/ vegetable glycerin ratio 70/30. Nicotine delivery 4.56 ng/mL after 20 puffs in 10 minutes **Button‐operated device** (eGO), nicotine concentration 36 mg/mL. Propylene glycol/ vegetable glycerin ratio 70/30. Nicotine delivery 6.9 ng/ml after 10 puffs in 5 minutes (refillable)	
Outcomes	Visits: baseline, day 7, day 14, day 21 CO measured (day 0, 7, 14, 21); adverse events (nausea, dizziness) Also: number of tobacco cigarettes smoked per day (self‐report); EC puffs per day (self‐report)	
Study funding	This study was supported by the National Institute on Drug Abuse of the National Institutes of Health under Award Number P50DA036107 and the Center for Tobacco Products of the U.S. Food and Drug Administration. JY is also funded by the Penn State Cancer Institute (PSCI) and TE is also supported by U54DA036105. The content is solely the responsibility of the authors and does not necessarily represent the official views of the National Institutes of Health or the Food and Drug Administration.	
Author declarations	JF has done paid consulting for pharmaceutical companies involved in producing smoking cessation medications, including GSK, Pfizer, Novartis, J&J, and Cypress Bioscience. TE is a paid consultant in litigation against the tobacco industry and the electronic cigarette industry and is named on a patent application for a device that measures the puffing behaviour of electronic cigarette users.	
Notes		
***Risk of bias***		
**Bias**	**Authors' judgement**	**Support for judgement**
Random sequence generation (selection bias)	Unclear risk	Used 2 ENDS in a random order – not enough information
Allocation concealment (selection bias)	Unclear risk	Not reported
Blinding of participants and personnel (performance bias) All outcomes	Low risk	Unable to blind, but interventions judged equally intensive
Blinding of outcome assessment (detection bias) All outcomes	Low risk	Objective outcome ‐ CO monitoring (CO < 10 ppm)
Incomplete outcome data (attrition bias) All outcomes	Low risk	No loss to follow‐up
Selective reporting (reporting bias)	Unclear risk	Unclear what outcomes were prespecified

ACS: acute coronary syndromes AE: adverse event AIDS: acquired immunodeficiency syndrome AIX: augmentation index AJCC: American Joint Committee on Cancer  ALT: alanine aminotransferase AOD: alcohol or other drugs AST: aspartate aminotransferase BDI: Beck Depression Inventory  BL: baseline  BMI: body mass index BoE: biomarkers of exposure BP: blood pressure BUN: blood urea nitrogen CAR: continuous smoking abstinence rate CAT: COPD assessment tool CC: combustible cigarette CDSS: Calgary Depression Scale for Schizophrenia CEMA: 2‐cyanoethylmercapturic acid CM: contingency management CO: carbon monoxide COHB: carboxyhaemoglobin conv‐cig: conventional cigarette COPD: chronic obstructive pulmonary disease COT: cotinine CPD/cpd: cigarettes per day CRF: cardiovascular risk factors CRO: contract research organization CS: complete substitution CV: cardiovascular CVD: cardiovascular disease DBP: diastolic blood pressure DCRF: dental clinical research facility DHMC: Dartmouth Hitchcock Medical Center DSM‐IV/V: Diagnostic and Statistical Manual of Mental Disorders IV/V ECG: electrocardiogram EC(IG): electronic cigarette eCO: expired carbon monoxide EEC: enhanced e‐cigarette coaching  eGO: brand of electronic cigarette eGFR: estimated glomerular filtration rate EMA: ecological momentary assessment ENDS: electronic nicotine delivery system EOP: end of pregnancy EoS: end of study EQ‐5D: EuroQol‐5 EVP: electronic vaping product FEF: forced expiratory flow FEV1: forced expiratory volume FMD: flow‐mediated dilation FTCD: Fagerström Test for Cigarette Dependence FTND: Fagerström Test for Nicotine Dependence FU: follow‐up FVC: forced vital capacity GGT: gamma‐glutamyl transferase GP: general practitioner doctor H2O: water HADS: Hospital Anxiety and Depression Scale HCT: haematocrit HCV: hepatitis C HDL(C): high‐density lipoprotein HEETS: tobacco sticks specifically processed and manufactured for IQOS HIV: human immunodeficiency virus HPHC: harmful and potentially harmful constituents HR: heart rate HRQoL: health‐related quality of life HS: high school hs‐CRP: high‐sensitivity C‐reactive protein HTP: heated tobacco products LCQ: Leicester Cough Questionnaire IPI: inter‐puff intervals IQOS: a heat‐not‐burn product that uses real tobacco IQR: interquartile range ITT: intention‐to‐treat LDL: low density lipoprotein LTA: light transmission aggregometry MCC: mucociliary clearance MCH: mean cell haemoglobin MCHC: mean cell haemoglobin concentration MDA: malondialdehyde MMSE: Mini Mental Status Examination MMT: methadone maintenance treatment MNWS(‐R): modified Minnesota Nicotine Withdrawal MPS: Mood and Physical Symptoms scale MTSS: Motivation to Stop Scale MWS‐R: Revised Minnesota Nicotine Withdrawal scale n/a: not applicable NCS: not clinically significant NCT: National Clinical Trials NEC: nicotine electronic cigarette NEQ: nicotine equivalents Nic: nicotine NNAL: carcinogen found in tobacco smoke (4‐(methylnitrosamino)‐1‐(3‐pyridyl)‐1‐butanol) NNC: non‐nicotine cigarette NNK: nicotine‐derived nitrosamine ketone NR: not reported NRT: nicotine replacement therapy NS: not stated NSP: nicotine salt e‐liquid pods NVP: nicotine vaping product OKHL: Oklahoma Tobacco Helpline OST: opioid substitution therapy OTH: Oklahoma Tobacco Helpline OTP: other tobacco products PAI‐1: plasminogen activator inhibitor type 1 PANSS: Positive and Negative Syndrome Score PAOD: peripheral arterial occlusive disease PBR: perfused boundary region PCV: haematocrit pd: per day PEC: placebo electronic cigarette PEF: peak expiratory flow PG: propylene glycol PI: principal investigator PIS: patient information sheet  PLTs: platelets PLWHA: people living with HIV/AIDS PP(A): point prevalence (abstinence) ppm: parts per million PTSD: post‐traumatic stress disorder PV: personal vaporizers PWV: pulse wave velocity QL: quitline QSU(Brief): Questionnaire of Smoking Urges‐Brief RA: research assistant RBC: red blood cells SAE: serious adverse event SBP: systolic blood pressure  SCQ‐A: Smoking Consequences questionnaire‐Adult SD: standard deviation SDM: standardised mean SMI: serious mental illness SMS: short message service SNOT‐22: Sino‐Nasal Outcomes Test SRNT: Society for Research on Nicotine and Tobacco SSS: stop‐smoking services TAU: treatment‐as‐usual TC: total cigarettes, tobacco cigarettes TCD: transcranial doppler THC: tetrahydrocannabinol TLFB: timeline follow‐back TNE: total nicotine equivalents TQD: target quit date UC: usual care USB: universal serial bus VA: Veterans Association VAMC: Veterans Affairs Medical Center VAS: visual analogue scale VG: vegetable glycerine VLNC: very low nicotine content VNP: vaporized nicotine products VO2: oxygen consumption WBC: white blood cells

### Characteristics of excluded studies [ordered by study ID]

**Table:**

Study	Reason for exclusion
Adkison 2013	Although this study uses a prospective cohort design, no data on EC use were collected at baseline, with EC use data only being available at follow‐up
Al‐Delaimy 2015	Observational study with no intervention provided ‐ included in previous versions, but excluded from 2020
Bianco 2019	Ineligible intervention
Caponnetto 2019	Ineligible intervention
Chaumont 2019	Ineligible intervention
ChiCTR2100044062	Wrong intervention
Collins 2019	Ineligible intervention
Cox 2019a	Short‐term abstinence only (< 6 months)
Douptcheva 2013	Longitudinal study, but no data are reported for smoking cessation or reduction or for adverse events
Dyer 2023	Wrong outcomes
Farsalinos 2013a	Included people that had already stopped smoking conventional cigarettes
Grana 2014b	Observational study with no EC intervention provided ‐ included in previous versions, but excluded from 2020
ISRCTN54776958	Wrong outcomes
James 2016	Follow‐up at 12 weeks, AE data not collected
Kasza 2013	Longitudinal study, but no data were reported for smoking cessation or for adverse events
Kimber 2023	No valid short‐term outcomes. Study 12 weeks (less than 6 months).
Kotlyar 2022	Wrong intervention
Krysinski 2021	Participants will be given a choice of a range of smoke‐free products including EC and heat not burn.
Manzoli 2015	Observational study with no EC intervention provided ‐ included in previous versions, but excluded from 2020
Miura 2015	Tests a device that is not an EC
NCT03249428	The study no longer includes e‐cigarettes
NCT03575468	Ineligible intervention
NCT05658471	Wrong patient population
Nolan 2016	Short‐term abstinence only (< 6 months)
Palamidas 2014	Short‐term EC use only
Pearson 2012	Longitudinal study, but no data were reported for smoking cessation or reduction or for adverse events
Song 2020	Ineligible patient population
St.Helen 2020	Ineligible intervention
Tucker 2018	Short‐term abstinence only (< 6 months)
Wagener 2014	EC use for up to 1 week, but did not report on any adverse events
Walele 2016a	RCT but follow‐up too short
Walele 2016b	RCT but follow‐up too short

AE: adverse events EC: electronic cigarette RCT: randomized controlled trial

### Characteristics of studies awaiting classification [ordered by study ID]

**Table:** UMIN000051684 2023

Methods	RCT Setting: dental surgery, West Java, Indonesia
Participants	Target sample 34 Inclusion criteria: 18 to 65 years, smoke CC Exclusion criteria: acute illness that required treatment (subjects who had a viral infection) within 4 weeks before the enrolment visit, having oral soft tissue disease or any type of gingival overgrowth other than those caused by plaque, suffering from periodontitis, having a history of tooth loss due to periodontitis, being treated with anticoagulant therapy in the previous 6 months, using prostheses or orthodontic appliances, history of alcoholism or drug/chemical abuse, taking any drug or substance (other than tobacco) that interferes with the cyclooxygenase pathway (e.g. anti‐inflammatory drugs including aspirin and ibuprofen) in the 14 days prior to the enrolment visit, taking antibiotics in the 14 days prior to the enrolment visit, breastfeeding or pregnant women.
Interventions	2 arms EC vs control (continued CC use)
Outcomes	Investigating effect of switching to vaping from tobacco cigarettes on gingival health
Notes	New to 2024 Contact: Indra Mustika, Universitas Padjadjaran, West Java Indonesia. Email: indra.mustika@unpad.ac.id

**Table:** UMIN000051685 2023

Methods	RCT, 2‐arm, non‐blinded Setting: dental surgery, West Java, Indonesia
Participants	Target sample 34 Inclusion criteria: 18 to 65, current CC use Exclusion criteria: having an acute illness that required treatment (subjects who had a viral infection) within 4 weeks before the enrolment visit, having oral soft tissue disease or any type of gingival overgrowth other than those caused by plaque, suffering from periodontitis, having a history of tooth loss due to periodontitis, being treated with anticoagulant therapy in the previous 6 months, using prostheses or orthodontic appliances, history of alcoholism or drug/chemical abuse, taking any drug or substance (other than tobacco) that interferes with the cyclooxygenase pathway (e.g. anti‐inflammatory drugs including aspirin and ibuprofen) in the 14 days prior to the enrolment visit, taking antibiotics in the 14 days prior to the enrolment visit, breastfeeding or pregnant women
Interventions	Intervention: EC Control: continued CC use
Outcomes	Impact of switching from tobacco smoking to vape use on the number of buccal and palatal mucosal micronucleus cells
Notes	New to 2024 Contact: Indra Mustika, Universitas Padjadjaran, West Java Indonesia. Email: indra.mustika@unpad.ac.id

CC: combustible cigarette EC: electronic cigarette RCT: randomized controlled trial

### Characteristics of ongoing studies [ordered by study ID]

**Table:** ACTRN12619001787178

Study name	Project NEAT: NicotinE As Treatment for tobacco smoking following discharge from residential withdrawal services
Methods	RCT Project NEAT: A randomized controlled trial to examine the efficacy of vaporised nicotine products and telephone quitline support compared with nicotine replacement therapy and telephone quitline support when used following discharge from residential withdrawal services Setting: Australia (New South Wales, Queensland, Victoria) Recruitment 4 hospital sites: Belmont Hospital, Belmont; St Vincent's Hospital, Darlinghurst; Turning Point Drug and Alcohol Centre, Richmond; Royal Brisbane & Womens Hospital, Herston
Participants	Target sample size: 926 Inclusion criteria: Aged 18 or over Daily tobacco smoker (10 or more cigarettes) on entering withdrawal unit Accessing treatment from participating services Want to quit smoking in the next 30 days Has capacity to consent and able to understand the participant materials and follow the study instructions and procedure (e.g. sufficient English language ability and not too unwell as judged by medical staff) Exclusion criteria: Pregnant or breastfeeding Enrolled in another study Scheduled to be transferred to a long‐term residential rehabilitation service following discharge from the withdrawal unit Used VNP (containing nicotine) in the last 30 days Currently engaged in Quitline’s call‐back services No ready access to a phone
Interventions	Condition 1: vaporised nicotine products and Quitline Condition 2: current best practice treatment for tobacco smoking (combination nicotine replacement therapy and Quitline)
Outcomes	9 months after inpatient withdrawal unit discharge: Self‐reported 7 months continuous abstinence from tobacco smoking Biochemically verified 7‐month continuous abstinence from tobacco smoking 3 and 9 months after inpatient withdrawal unit discharge: 30‐day point prevalence abstinence 7‐day point prevalence abstinence Abstinence from all nicotine/tobacco products
Starting date	Anticipated enrolment: 19 December 2019. Anticipated date last data collection: 19 September 2022
Contact information	Prof Billie Bonevski, Billie.Bonevski@newcastle.edu.au
Notes	Funding: National Health and Medical Research Council (grant number: G1800272), Canberra ACT 2601

**Table:** ACTRN12621000148875

Study name	HARMONY: HARM reduction for Opiates, Nicotine and You
Methods	Design: randomized, single‐blinded, parallel‐group trial
Participants	Inclusion criteria: written, informed consent; 18 to 65 years; accessing opioid agonist treatment from a participating service; current daily tobacco smokers on self‐report; want to quit or cut down their tobacco smoking; willing and able to comply with requirements of study (including having access to a phone) Exclusion criteria: breastfeeding or pregnant; severe medical disorder assessed by study medical officer (such as, but not limited to, unstable cardiovascular/peripheral vascular disease, poorly controlled hypertension); severe and unstable psychiatric disorder assessed by study medical officer (such as, but not limited to, acute psychosis, severe anxiety and/or mood disorder, intent to harm self or others); current enrolment in a clinical trial involving any investigational drug; gular use (more than one day per week) of VNP or EC containing nicotine in the last 30 days; not available for FU Motivated to quit
Interventions	Comparison of a 12‐week course of liquid nicotine delivered via EC or Vaporised Nicotine Product (VNP) to best practice Nicotine Replacement Therapy (NRT) Condition 1: EC (VNP) (Innokin Endura T18‐II starter kit) • Device loaded with one bottle of 12 mg/mL e‐liquid • An additional seven (7) bottles of 12 mg/mL e‐liquid • A brief information session on how to use the VNP • A VNP information pack including safe storage of e‐liquid nicotine and disposal • 1‐week supply of nicotine patches • Training on the use of NRT patches • Where possible, ensure that participant uses the VNP before leaving and leaves wearing an NRT patch. Adherence will be measured via questionnaires. In addition to the VNP, liquid nicotine and NRT patches, participants will be shown the New Zealand website vapingfacts.health.nz/ and encouraged to visit the site as an online resource throughout the trial. Participants will also receive training in the forms of brief videos, information pamphlets, user manuals and interactive discussions with research staff.
Outcomes	Baseline, 12, 24 weeks Primary outcome: self‐reported 7‐day PPA from tobacco smoking assessed in the following dichotomous question “In the last 7 days, have you smoked a cigarette, even a puff?" (Week 12) Secondary outcome: a cost consequence study setting out detailed comparative costs of treatments ‐ from the perspective of healthcare provider and primary and secondary outcomes of the VNP and NRT Adverse events recorded Biochemically verified PPA: this will be measured via a CO monitor breath test for participants who self‐report 7‐day PPA at end of treatment (Week 12) Changes in nicotine craving and withdrawal symptoms
Starting date	Registered Feb 2021. Record updated Jan 2024.
Contact information	Adrian Dunlop, adrian.dunlop@health.nsw.gov.au
Notes

**Table:** Berlin 2019

Study name	Randomized, placebo‐controlled, double‐blind, double‐dummy, multicentre trial comparing electronic cigarettes with nicotine to varenicline and to electronic cigarettes without nicotine: the ECSMOKE trial protocol
Methods	3‐arm, randomized, placebo‐controlled, multicentre, double‐blind, double‐dummy, parallel‐group phase III type trial Setting: smoking cessation clinics of both academic and community hospitals Recruitment is either local (a) directly by the centres or centralized (b) using a web page and a centralized study‐specific phone number and email address. People who smoke, intending to quit smoking, are recruited by advertisement in pharmacies, physicians’ offices situated in the catchment area of each investigator’s centre, by local newspapers and in public places of the centres’ healthcare facilities. Candidates to participate can register by the study’s website, unique email address, and phone number. Registration is followed by a phone screening before dispatching to the study centres. Only 1 person by household will be recruited.
Participants	Estimated enrolment: 650 participants Inclusion criteria: people who smoke, ≥ 10 CPD (factory‐made or roll‐your‐own) in the past year; aged 18 to 70; motivated to quit, defined as a score > 5 on a visual rating scale ranging from 0 (not motivated at all) to 10 (extremely motivated); informed consent; understanding and speaking French; women of childbearing age can be included if they use an effective contraceptive method: either hormonal contraception or an intrauterine device started at least 1 month before the first research visit; individual affiliated to a health insurance system; previous failure of NRT for smoking cessation Exclusion criteria: any unstable disease condition within the last 3 months defined by the investigator as major change in symptoms or treatments, such as recent myocardial infarction, unstable or worsening angina, severe cardiac arrhythmia, unstable or uncontrolled arterial hypertension, recent stroke, cerebrovascular disease, obliterative peripheral arterial disease, cardiac insufficiency, diabetes, hyperthyroidism, pheochromocytoma, severe hepatic insufficiency, history of seizures, severe depression, COPD; any life‐threatening condition with life expectancy of < 3 months; alcohol use disorder defined as a score ≥ 10 on the Alcohol Use Disorders Identification Test (AUDIT)‐C questionnaire; abuse of or dependence on illegal drugs in the last 6 months, revealed by medical history; regular use of tobacco products other than cigarettes; current or previous (last 6 months) use of EC; pregnancy/breastfeeding; protected adults; current or past 3 months participation in another interventional research; current or past 3 months use of smoking cessation medication such as varenicline, bupropion, NRTs; known lactose intolerance (placebo tablets contain lactose); hypersensitivity to the active substance or to any of the excipients; known severe renal failure
Interventions	A) **EC without nicotine** (ECwoN) plus placebo tablets of varenicline (0.50 mg) administered by oral route: placebo condition B) **EC with nicotine** (ECwN) plus placebo tablets of varenicline: ECwN condition. C) Reference: ECwoN plus 0.5 mg varenicline tablets: **varenicline condition**. Varenicline administered according to the marketing authorization E‐cigarette details: EC device Mini iStick kit (20 W) Eleaf, clearomzser: GS Air M with resistance of 1.5 ohm. To keep the blinding, the clearomizer’s Pyrex window is of grey colour not allowing to distinguish the colouration of the e‐liquid containing nicotine. Liquid for EC is manufactured by GAIATREND SARL (www.gaiatrend.fr/fr/). All participants will be delivered a short manual and a video specifically developed for this study explaining the use of EC. At each visit, participants receive verbal counselling about the use of the EC device and answers to their questions about handling the EC device. Behavioural support: Brief behavioural smoking cessation counselling for all participants is administered at all visits by the investigators specialized in smoking cessation. It is based on the national guidelines for smoking cessation. Treatment duration: 1 week + 3 months
Outcomes	Week 2, 4, 8, 10, 12, 24 after target quit day Primary outcome: Continuous smoking abstinence rate (CAR) (abstinence from conventional/combustible cigarettes) during the last 4 weeks (weeks 9 to 12) of the treatment period of 3 months Secondary outcomes: Safety profile PPA rate CAR confirmed by urinary anabasine concentration Changes in cpd consumption Craving for tobacco and withdrawal symptoms with respect to baseline
Starting date	17 October 2018
Contact information	Ivan Berlin, ivan.berlin@aphp.fr
Notes

**Table:** Cox 2022

Study name	E‐cigarettes vs usual care for smoking cessation when offered at homeless centres
Methods	RCT. A multicentre, cluster‐randomized controlled trial Setting: 32 centres across 6 areas in Great Britain: Scotland; Wales; London; South‐East England; South‐West England and East England
Participants	Estimated enrolment: 480 participants Inclusion criteria: currently accessing homeless centre services and actively engaging with the service; > 18 years; self‐reported daily smoking, then biochemically verified Exclusion criteria: never‐ and ex‐smokers; currently using a smoking cessation aid; unable to provide written consent; not known to centre staff; allergic to any of the e‐liquid ingredients (EC arm only); pregnant
Interventions	EC: refillable Cluster rather than individual randomization will be used. Arm 1: Planned intervention Delivery of the EC intervention will be as per our feasibility study. Centre staff will provide EC arm participants with a tank‐style refillable EC starter kit (e.g. the PockeX as used in our feasibility study or similar model determined via our PPI work), a choice of nicotine strength e‐liquids (12 mg/mL and 18 mg/mL) and flavours (tobacco, menthol or fruit) and an EC fact‐sheet. E‐liquids (5 x 10 mL bottles per week) will be supplied for 4 weeks at weekly intervals by centre staff. Arm 2: Control/comparator group Control arm will be usual care (UC). This will include very brief advice (VBA) to quit (in the form of an ‘NHS choices’ leaflet adapted for this population as used in our feasibility study) and signposting to the local SSS. All participants (intervention and control) will be offered a GBP 15 Love2Shop gift card (which cannot be used for tobacco or alcohol purchases) for each follow‐up appointment attended.
Outcomes	Baseline, 4, 12, and 24 weeks Primary outcome measure Current primary outcome measure as of 27/07/2022: Sustained CO‐validated smoking cessation at 24 weeks using the Russell Standard for cessation trials and intention‐to‐treat analysis (i.e. no more than 5 cigarettes since 2 weeks from baseline, validated by expired CO < 8 ppm Secondary outcome measures Current secondary outcome measure as of 27/07/2022: Smoking reduction at 24 weeks 7‐day point prevalence quit rates at 4, 12, and 24 weeks self‐reported and validated by expired CO < 8 ppm Changes in the frequency of risky smoking practices (e.g. sharing cigarettes, smoking discarded cigarettes) Cost‐effectiveness of the intervention measured using a service use questionnaire and the EQ‐5D‐5L (4, 12, 24 weeks) Fidelity of intervention implementation Mechanisms of change measured quantitatively via questions (e.g. attitudes and perceptions of e‐cigs) Contextual influences and sustainability telephone interviews
Starting date	Start date 23 April 2021. Estimated study end date: 31 August 2024
Contact information	Lynne Dawkins, dawkinl3@lsbu.ac.uk
Notes	New to 2022 update

**Table:** El‐Khoury  2021

Study name	Preference‐based tools for smoking cessation among disadvantaged smokers, a pragmatic randomised controlled trial (STOP)
Methods	RCT France
Participants	Estimated enrolment: 528 Inclusion criteria: daily smokers (≥ 5 cigarettes/day); low socioeconomic position; available for at least 4 appointments over a 6‐month period; affiliation to or benefiting from social security or state medical support Exclusion criteria: individuals who do not speak French; major citizens protected by law, adults unable to express their consent; pregnant women; regular smokers who vape daily (at least once a day)
Interventions	EC: type not stated Arm 1: The STOP intervention Assisting smokers with low socioeconomic position in their smoking cessation attempt. Routine care and adapted advice supplemented with a free delivery of any or several type(s) of nicotine replacement therapy (NRT) (patches, inhalers, gum, tablets, etc.) and/or an e‐cigarette + e‐liquid, based on the smokers' preference and choice Arm 2: Standard care Participants randomised to the standard care group will be given standard care in assisting their smoking cessation attempt, but without free delivery of NRT or e‐cigarettes. Standard care includes motivational interviewing, advice to quit, and prescription for NRTs.
Outcomes	Smoking abstinence at 6 months after inclusion Total number of days of abstinence at 6 months Smoking abstinence at 1 and 3 months after inclusion Number of relapses; CPD; proportion of participants who have significantly reduced daily smoking
Starting date	
Contact information	
Notes	New to 2022 update

**Table:** Hameed 2024

Study name	Clinical study protocol on electronic cigarettes and nicotine pouches for smoking cessation in Pakistan: a randomized controlled trial
Methods	RCT Setting: Pakistan Recruitment centres in 2 metropolitan districts: Islamabad and Rawalpindi
Participants	Estimated enrolment: 600 EC + counselling: estimated 200 Nicotine pouches + counselling: estimated 200 Counselling: estimated 200 Inclusion criteria: > 18; > 10 CPD; CC use for > 1 year; willing to stop CC use; sign a written consent form; 1 applicant per household; phone Exclusion criteria: pregnant; childbearing mothers; using other nicotine‐ and non‐nicotine‐based cessation therapies; chest pain, or another cardiovascular event or procedure (e.g. heart attack, stroke, insertion of stent, bypass surgery) Motivated to quit
Interventions	Provision of EC or nicotine pouches for 48 weeks 3 groups: EC and liquid + 4 basic counselling sessions every 12 weeks over 48 weeks Nicotine pouches + 4 basic counselling sessions every 12 weeks over 48 weeks Counselling only: 4 basic counselling sessions every 12 weeks over 48 weeks
Outcomes	Baseline, 12, 24, 36, 48 weeks. 60 weeks FU. AEs CPD Self‐reported point‐prevalence abstinence from CC in the previous week with biochemical validation will be used (exhaled carbon monoxide less than 10 parts per million (PPM)) 7‐day point‐prevalence abstinence from CC (at all subsequent check‐ups) (biochemically validated at weeks 12, 24, 36, 48, and 60) Harm‐reduction effect of e‐cigarettes and nicotine pouches
Starting date	Estimated starting date: December 2023 Estimated completion date: April 2025
Contact information	Abdul Hameed, +923315813713, hameedleghari@gmail.com Daud Malik, +923028560310, daud31us@yahoo.com
Notes	New to 2024 Funding: Foundation for a Smoke Free World INC. Funded by Philip Morris International. ClinicalTrials.gov NCT05715164

**Table:** Holliday 2022

Study name	ENHANCE‐D trial: Enhancing dental health advice
Methods	Design: RCT A pragmatic, multicentre, definitive, open‐label, 3‐arm, parallel‐group, individually randomized, controlled, superiority trial, comparing the clinical‐ and cost‐effectiveness, and safety of enhanced smoking cessation interventions to usual care, and each other (with an internal pilot) Setting: 56 NHS primary dental care setting in England and Scotland. Newcastle Clinical Trials Unit, UK
Participants	Estimated enrolment: 1460 participants, 455 periodontitis subgroup Adult regular tobacco smokers attending an NHS dental setting. Dental patients with or without gum disease Inclusion criteria: a basic periodontal examination completed within the last 3 months; ≥ 18 years; current smoker Periodontitis subgroup: minimum of 16 natural teeth; diagnosis of periodontitis stage II (or greater) Exclusion criteria: pregnant or currently breastfeeding; enrolled in another interventional research trial; used quit‐smoking aid or reduce/quit alcohol; phaeocromocytoma, uncontrolled hyperthyroidism, extensive dermatitis/skin disorder; hypersensitivity to nicotine or any component of the study products; taking: clozapine, olanzapine, theophylline or aminophylline
Interventions	EC: EC starter kit. Stainless Steel Aspire PockeX e‐cigarette, coil replacement pack, 3‐pin plug, 10 x Halo standard 10ml e‐liquids (4 flavour options available) Condition: smoking cessation in dental patients with or without gum disease Arm 1: E‐cigarette (EC) starter kit with single‐visit behavioural support (same behavioural intervention as the NRT group). Participants will be expected to source their own supply of e‐liquid after the initial supply and advice will be given as to where to source suitable MHRA registered products. Duration will vary depending on use of EC. Arm 2. Nicotine Replacement Therapy (NRT): standard 12‐week course of combination NRT with single‐visit behavioural support including the offer of NRT. 12‐week course of combination NRT (patch plus faster‐acting form such as chewing gum or lozenge), in line with current recommendations. Duration will be 12 weeks if a participant wants to continue NRT after initial 4‐week supply. Nicotine transdermal patches. Option 1: NiQuitin 7 mg, 14 mg, 21 mg (24‐hour patch). Option 2: Nicorette invisi 10 mg, 15 mg, 25 mg (16‐hour patch) Nicorette gum 2 mg, 4 mg. Nicorette lozenge 2 mg, 4 mg Arm 3. VBA: usual care (control) 1. VBA is usual care for smokers in dental settings usually following the 3As: Ask, Advise, Act technique. This will signpost participants to a GP, pharmacy or stop‐smoking service (SSS). 2. Participants in the control group will be free to use NRT or ECs as they wish, but these will not be provided by the dental professional. 3. Conducted at baseline visit, only a 5‐minute intervention All patients will be followed up for up to 12 months from baseline.
Outcomes	6 months for periodontal health parameters and 12 months for smoking outcomes To compare smoking abstinence at 6 months of NRT and EC to usual care and to each other (all participants). Biochemically verified smoking abstinence at 6 months, carbon monoxide monitor. To compare the periodontal health at 6 months of NRT and EC interventions to usual care and to each other, for those with periodontitis at baseline. Percentage of periodontal sites at 6 months with PPD (Pocket Probing Depths) ≥ 5 mm AEs; oral health, and oral health QoL; to evaluate nicotine dependence, urges to smoke, withdrawal symptoms, and longer‐term smoking abstinence (12 months); cost benefit. SES inequalities Expired air carbon monoxide (eCO). Continuous biochemically verified smoking abstinence at 12 months. Fagerstrom Test for Nicotine Dependence (FTND). Cigarette withdrawal symptoms are measured using Mood and Physical Symptoms Scale (MPSS). Oral Health Quality of Life Assessment (OHQoL‐UK). Oral health is measured using number of teeth at baseline and 6 months. Health economic evaluation.
Starting date	Protocol 2022 Study start date February 2022. Estimated completion date March 2025.
Contact information	Dr Richard Holliday richard.holliday@newcastle.ac.uk Professor Elaine McColl, Newcastle University
Notes	New to 2023 update

**Table:** Howard 2022

Study name	Vaporized nicotine products (VNP) versus nicotine replacement therapy for tobacco smoking cessation among low‐socioeconomic status smokers: a randomised controlled trial
Methods	Design: RCT Recruitment: study advertisements across online and social media platforms such as Facebook advertisements. Participants in a recently completed clinical trial comparing cytisine versus varenicline for smoking cessation who consented to being contacted about future research and were receiving a government pension were also invited to take part. Setting: National Drug and Alcohol Research Centre at the University of New South Wales, Sydney, Australia
Participants	Target N: 1058 Inclusion criteria: participants can be included if they meet the following criteria: Willing to allow the research team and study clinician to access their data for quality assurance and to maintain the integrity of the trial 18 years of age or older Receiving a government pension or allowance (proxy for low SES) Are a current daily smoker Interested in quitting smoking and using the study products and willing to make a quit attempt in the next 2 weeks Have a mobile phone that can receive text messages Agree to use the allocated study product and refrain from using another quit‐smoking medication whilst using the study products Willing to receive daily quit‐support text messages during the treatment period (with the option to opt out during the study)
Interventions	Vaporized nicotine product (VNP) devices (1 tank device and 1 pod device) for 8 weeks plus 5‐week Text Message behavioural quit Support (TMS) with the option to opt out at any stage if desired. Participants will receive a mix of quit‐smoking support text messages with content including information on how to use the study products; coping with nicotine withdrawal symptoms; study progress updates; and motivational ‘feel good’ messages. A mix of text, emojis and links to resources such as videos, websites and Graphics Interchange Format (GIF) images, will be used throughout the TMS programme to promote engagement with the programme. Each device will be charged using the provided USB charger and wall adaptor. A replacement battery and replacement coils (5 pieces per pack) will also be provided. The VNP tank device used is the Innokin Endura T18 Personal Vaporizer, which has a refillable 2.5 mL tank for the e‐liquid (18 mg/mL nicotine). 3 e‐liquid flavours will be provided: tobacco, menthol and a fruit flavour. The study will have 3 e‐liquid suppliers to guarantee ongoing supply throughout the study: Lumo Liquid in 10 mL bottles; VAPO e‐liquid in 30 mL bottles; and DashVapes e‐liquid in 30 mL bottles. All e‐liquids are 18 mg/mL in strength. Lumo Liquid ingredients are as follows (w/w): tobacco flavouring (1.19%), nicotine (1.60%), vegetable glycerine (24.56%), propylene glycol (73.24%); menthol flavouring (4.83%), nicotine (1.60%), vegetable glycerine (22.99%), propylene glycol (71.18%); strawberry flavouring (0.63%), nicotine (1.60%), vegetable glycerine (33.00%), propylene glycol (71.00%). VAPO e‐liquid additional flavour ingredients are as follows (w/w): tobacco flavouring (25.88%), nicotine (17.25%), vegetable glycerine (36.53%), propylene glycol (20.34).
Outcomes	Primary outcome: CO‐verified 6‐month continuous abstinence at 7‐month follow‐up. Continuous 6‐month abstinence will be defined as having remained quit for 6 months (having smoked no more than 5 cigarettes in that time), and a CO level of ≤ 5 ppm. Depending on the participant's indicated preference, the CO breath test will be self‐administered using a hand‐held iCO™ Smokerlyzer® (using provided instructions), or administered by a trained researcher using a hand‐held iCO™ Micro+™ Smokerlyzer® with a disposable, one‐use mouthpiece. Both devices are non‐invasive and require the participant to blow air into the device for 15 seconds to measure their CO level. An exhaled CO level of ≤ 5 ppm will be considered abstinent. The final follow‐up interview will occur 7 months after the baseline interview completion date. Secondary outcome: Change in financial stress (assessed using Index of Financial Stress)
Starting date	
Contact information	
Notes

**Table:** ISRCTN14068059

Study name	E‐cigarettes for smoking cessation and reduction in people with a mental illness, ESCAPE
Methods	RCT, multicentre Setting: GP practice, hospital, England, UK Organisation: University of York Tees, Esk and Wear Valleys NHS Foundation Trust; Bradford District Care NHS Foundation Trust; Sheffield Clinical Commissioning Group Hq; Oxford NHS Foundation Trust; Greater Manchester Mental Health NHS Foundation Trust; South West Yorkshire Partnership NHS Foundation Trust; Nottinghamshire Healthcare NHS Foundation Trust; Lancashire and South Cumbria NHS Foundation Trust; Norfolk and Suffolk NHS Foundation Trust; CRN North East and North Cumbria; CRN East of England; The Burns Practice Bennetthorpe; Conisborough Medical Practice Conisbrough, Doncaster; Woodstock Bower Surgery, Rotherham
Participants	Target N = 616 Mental Health Trusts and GP practices mainly in Yorkshire (UK) Inclusion: adults (aged over 18 years) receiving treatment for a mental illness in primary or secondary care, who smoke regularly, willing to quit or reduce cigarette smoking Exclusion: inpatient admission in the last 3 months; currently using EC regularly (at least weekly); participating in other smoking cessation trials; receiving treatment for drug or alcohol use; Alzheimer’s disease or dementia; pregnant or breastfeeding
Interventions	Group 1 EC: e‐cigarette and e‐liquid to use for 4 weeks in addition to the usual care they are receiving. EC starter kit, a 20 mg/mL strength DOTPRO e‐cigarette starter kit (https://www.liberty‐flights.co.uk/DOT‐PRO/DOT‐PRO‐Vape‐Kit/) will be offered in a choice of flavours. The starter kit containing a pod‐based e‐cigarette, a 4‐week supply of refill pods and an information leaflet. Brief face‐to‐face consultation with a clinician, who will explain how to use the e‐cigarette and provide information to enable participants to make positive changes to their smoking behaviour. Encouraged to set a quit date. Participants will be provided with an e‐liquid supply for 4 weeks. Group 2, usual care: participants will receive care as usual but will receive an e‐cigarette and some e‐liquid at the end of the study at the 6‐month follow‐up.
Outcomes	Baseline, 1, 6 months Questionnaire; CO monitor Primary outcome measure Self‐reported 7‐day point prevalence abstinence measured using a questionnaire (question written in‐house) at 6 months Co‐verified quit (main outcome) measured using a CO monitor (smokylizer) at 6 months Secondary outcome measures General smoking‐related characteristics, abstinence, quit attempts and methods (including e‐cigarettes), measured using a questionnaire (questions written in‐house) at baseline, 1 month and 6 months Nicotine dependence measured using the Fagerstrom Test for Nicotine Dependence at baseline, 1 month and 6 months Strength of urges to smoke measured using the SUTS questionnaire at baseline, 1 month and 6 months Motivation to quit measured using the Motivation To Stop Scale (MTSS) at baseline, 1 month and 6 months Mental wellbeing measured using PHQ‐9 and GAD‐7 at baseline and 6 months Alcohol use measured using AUDIT‐C at baseline and 6 months Health‐related quality of life measured using EQ‐5D‐5L at baseline and 6 months Attrition measured using a questionnaire (questions written in‐house) at 1 month and 6 months Adherence rate measured using a questionnaire (questions written in‐house) at 1‐month follow‐up Cost‐effectiveness measured using a questionnaire (questions written in‐house) at baseline, 1 month and 6 months Adverse events measured using a questionnaire (questions written in‐house) at 1 month and 6 months
Starting date	Study start date: March 2023 Estimated study end date: April 2025
Contact information	Dr Anna‐Marie Marshall, a.marshall@york.ac.uk
Notes	New to 2024 Funding: Yorkshire Cancer Research (UK)

**Table:** ISRCTN17691451

Study name	ESCAPE: E‐cigarettes for smoking cessation and reduction in people with mental illness
Methods	RCT Setting: hospitals, UK West Park Hospital; Bradford District Care NHS Foundation Trust; Sheffield Clinical Commissioning Group
Participants	The target sample size for the randomized controlled feasibility trial is 72, with 36 participants allocated to each group. Inclusion criteria: receiving treatment for a mental illness; > 18 years; smoker; willing to attempt to quit Exclusion criteria: inpatient admission in the last 3 months according to their health care record; smokers using EC; participating in other smoking cessation trials; being treated for comorbid drug or alcohol problems; Alzheimer’s disease or dementia; pregnancy or breastfeeding
Interventions	EC: Aspire PockeX electronic cigarette The intervention consists of an e‐cigarette starter kit containing a third generation e‐cigarette (Aspire PockeX), a 4‐week supply of e‐liquid (a choice of flavours and concentrations will be offered) and an information leaflet. Intervention delivery will take place at a previously scheduled appointment with a clinician. The control condition is usual care.
Outcomes	Baseline, 2 to 4 weeks, 1 month Primary outcome measure: Feasibility and acceptability outcomes: the primary feasibility outcome measures in the feasibility trial will be consenting rate and recruitment frequency. Clinical (smoking‐related) outcomes: continuous abstinence assessed at 1 month will be defined as not having smoked in the 2 weeks prior to follow‐up, verified by a CO reading below 10 ppm, in keeping with the standard measure used in Stop‐Smoking Services. Secondary outcome measures: Feasibility and acceptability outcomes. Characteristics of ‘usual care’ in different locations will also be noted, recording 2 interactions of patients with CMHTs or GPs at each site at baseline and using short pro‐forma with control group participants at 1‐month follow‐up. Clinical (smoking‐related) outcomes: Self‐reported abstinence 2 to 4 weeks from enrolment or target quit date (whichever is later) will be recorded at 1‐month follow‐up. The change in cigarette consumption (and reduction in exhaled breath CO reading) from baseline to 1‐month follow‐up will be calculated in both intervention and control group participants. Clinical (mental health‐related) outcomes Cost‐effectiveness Serious adverse events (SAE) and adverse events (AE)
Starting date	Start date 29 September 2021. Trial end date: 30 November 2022
Contact information	Professor Lion Shahab, lion.shahab@ucl.ac.uk Dr Elena Ratschen, elena.ratschen@york.ac.uk Dr Anna‐Marie Marshall, a.marshall@york.ac.uk
Notes	New to 2022 update

**Table:** ISRCTN61193406

Study name	Do e‐cigarettes help smokers quit when not accompanied by intensive behavioural support? A multi‐center randomized controlled trial
Methods	RCT Setting: UK Multicentre. Participants will be recruited mainly from hospitals and GP practices across the UK by the Clinical Research Network. The study is being organized by Queen Mary University of London (QMUL). Researchers from QMUL will provide the study treatment and conduct follow‐up calls.
Participants	1170 people who smoke tobacco cigarettes Inclusion criteria: Adult daily smokers who are motivated to stop smoking Must own a mobile phone and be willing to try either an online or texting treatment package, or both, or an e‐cigarette with or without telephone support Be happy to receive follow‐up calls Be able to read/write/understand English Exclusion criteria: Women who are pregnant Currently using an e‐cigarette
Interventions	Control: NHS Quit Now programme (QN) E‐cigarette starter pack with no ongoing support (EC) EC starter pack with helpline support (EC+) The study will aim to use a refillable EC that is similar to the type used in a previous EC trial (One Kit ‐ Innokin, UK Ecig Store), and one that is compliant with UK regulations, and not produced by a tobacco company.
Outcomes	Follow‐up at 4 weeks, 6 months and 12 months. CO at 6 and 12 months Primary outcome measure: Sustained smoking cessation at 6 months post‐TQD. This is measured by asking participants if they have smoked since their TQD at the 6‐month follow‐up. To be counted as a 'quitter', participants must report smoking no more than 5 cigarettes since 2 weeks post‐TQD with no smoking in the previous week, validated by carbon monoxide (CO) reading of < 8 ppm. Participants lost to follow‐up will be counted as smokers. Secondary outcome measures: Validated sustained abstinence rates measured by asking smoking status and taking a carbon‐monoxide reading at 12 months post‐TQD Validated sustained abstinence rates between 6 and 12 months, measured by asking smoking status and taking a carbon‐monoxide reading at 6 and 12 months Self‐reported 7‐day point‐prevalence abstinence, measured by asking smoking status in last 7 days at 4 weeks, 6 months and 12 months post‐TQD Cigarette consumption in non‐abstainers by vaping status, measured by questionnaire at four weeks, 6 and 12 months Frequency and severity of urges to smoke and withdrawal symptoms, measured by questionnaire at 4 weeks post‐TQD Weight, measured by asking weight at 4 weeks, 6 months and 12 months post‐TQD Respiratory symptoms, measured by questionnaire, at 4 weeks, 6 months and 12 months post‐TQD Treatment adherence and ratings, measured by questionnaire at 4 weeks (and 6 and 12 months for EC arms) Adverse reactions to EC, measured by questionnaire at 4 weeks, 6 and 12 months post‐TQD Cost‐effectiveness of the interventions, measured by questionnaires at baseline, 6 and 12 months Smokers' and healthcare professionals' views and opinions of the helpline, measured by one‐off qualitative interviews separate to the main trial
Starting date	Overall trial start date: 1 September 2020 Estimated completion date January 2025
Contact information	Dr Katie Myers Smith, katie.smith@qmul.ac.uk
Notes

**Table:** ISRCTN82413824

Study name	Effectiveness of electronic cigarettes compared with combination nicotine replacement therapy for smoking cessation in patients with chronic obstructive pulmonary disease and effect on lung health (ECAL Trial)
Methods	Multicentre, two‐arm randomized controlled trial with embedded cost‐effectiveness and cohort analyses (Prevention, Efficacy) Setting: England, Scotland, UK Study to look at CC abstinence, COPD and respiratory outcomes
Participants	Target sample size: 1250 Inclusion criteria: COPD diagnosis previously confirmed by post‐bronchodilator spirometry (FEV1/FVC < 0.7), any GOLD stage; current smoker (= 5 cigarettes per day); motivated to stop smoking; aged 35 or over Exclusion criteria: unable to perform spirometry to a satisfactory standard (e.g. due to dementia, lack of teeth, lack of coordination or not having a good oral seal); unsuitable to participate in the trial (e.g. terminal illness, unable to give informed consent); unable to participate in behaviour support calls; severe angina or unstable cardiovascular disease; end stage kidney disease/cirrhosis of the liver; taking NRT, bupropion, varenicline or ECs to stop or reduce smoking; in another trial of smoking cessation or COPD treatment/management; COPD exacerbation or inpatient hospital stay within the last 8 weeks; contraindications to spirometry within the last 12 weeks – tuberculosis infection, cardiac infarction, retinal detachment or surgery on the chest, abdomen, brain, ears or eyes
Interventions	EC + telephone support vs NRT + telephone support Intervention arm: Electronic Cigarettes (EC) ‐ At the baseline visit, participants will be given an EC starter pack and an initial supply of e‐liquid (up to 20 mg nicotine/mL). Participants will be provided with instructions on how to continue sourcing further supplies themselves from reputable vendors in their preferred nicotine strength and flavours. Comparator arm: Combination Nicotine Replacement Therapy (NRT) ‐ Participants will receive up to a 12‐week supply of a nicotine patch plus a fast‐acting nicotine product to be used in combination. Participants who do not wish to use patches (e.g. due to previous experience with skin irritation) will be offered 2 types of fast‐acting products. Telephone behavioural support: All participants (intervention and comparator arm) will be advised at the baseline visit that they will receive 6 weekly behavioural support telephone calls from stop‐smoking advisors which will commence within a few days of the baseline visit. During these calls, the advisor will deliver behavioural support according to the National Centre for Smoking Cessation Training Standard Programme including setting a TQD and providing further support around medication use.
Outcomes	Baseline, 4, 26, 52 weeks Questionnaire, CO monitored. Study to look at CC abstinence, COPD and respiratory outcomes. Primary outcome Abstinence from smoking since target quit date (TQD) biochemically validated (exhaled CO < 8 ppm), defined in accordance with the Russell Standard. This will be measured with a questionnaire and exhaled carbon monoxide measurement at 52 weeks post‐TQD. Secondary outcomes Abstinence from smoking for at least 26 weeks biochemically validated (exhaled CO < 8 ppm) measured using a questionnaire at 52 weeks 7‐day point prevalence abstinence from smoking biochemically validated (exhaled CO < 8 ppm) measured using a questionnaire at 52 weeks Self‐reported abstinence from smoking for at least 26 weeks measured using a questionnaire at 52 weeks Self‐reported 7‐day point prevalence abstinence from smoking measured using a questionnaire at 4, 26, and 52 weeks Reduction in cigarettes smoked (self‐report of any and > 50% reduction) from baseline to 52 weeks measured using a questionnaire, confirmed by reductions in expired CO readings at 52 weeks Reduction in cigarettes smoked (self‐report of any and > 50% reduction) from baseline to 26/52 weeks, measured using a questionnaire Continued use of the allocated product measured using a questionnaire at 4, 26 and 52 weeks Withdrawal symptoms and urges to smoke (change from baseline to 1/2/3/4 week) measured using the mood and physical symptom scale (MPSS) COPD Symptoms (change from baseline to 4/26/52 weeks) measured using COPD Assessment Test (CAT) and the Clinical COPD Questionnaire (CCQ) Number of COPD exacerbations over the past 52 weeks (change from baseline to 52 weeks) measured using a questionnaire Number of self‐reported upper respiratory tract over the past 52 weeks (change from baseline to 52 weeks) measured using a questionnaire Post bronchodilator spirometry (FEV1, FVC and MMEF change from baseline to 52 weeks). Forced Expiratory Volume in 1 Second (FEV1): The maximal volume of air that can be expired in the first second of a forced expiration from a position of full inspiration (measured in Litres (L) and also expressed as the % predicted for age, sex, height and race). Forced Vital Capacity (FVC): The maximal volume of air that can be expired during a forced and complete expiration from a position of full inspiration (measured in L and % predicted). Mean Mid‐Expiratory Flow (MMEF): The average flow between 25% and 75% of the FVC manoeuvre (measured in L/sec and % predicted). Health economic outcomes: Health‐related quality of life (EQ‐5D‐5L) measured using a questionnaire change from baseline to 4/26/52 weeks Use of healthcare resources and costs measured using a questionnaire at 26 and 52 weeks Cost‐effectiveness based on cost per quitter and cost per Quality‐Adjusted Life‐Year (QALY) at 52 weeks, and modelled cost per QALY over a patient's lifetime
Starting date	Study registered: October 2023. First enrolment March 2024. Estimated study completion: September 2025
Contact information	Amanda Farley, a.c.farley@bham.ac.uk Institute of Applied Health Research, University of Birmingham, UK
Notes	New to 2024 update. Funding: National Institute for Health and Care Research

**Table:** Lin 2024

Study name	Efficacy of Electronic Cigarettes vs Varenicline and Nicotine Chewing Gum as an Aid to Stop Smoking: A Randomized Clinical Trial ChiCTR2100048156 (Chinese Clinical Trial Registry)
Methods	RCT 3 arm open label, multicentre Setting: 7 sites in China. China‐Japan Friendship Hospital (Beijing, China), Peking University Health Science Center (Beijing, China), Beijing Hospital (Beijing, China), Beijing Xiyuan Hospital (Beijing, China), Beijing Geriatric Hospital (Beijing, China), Beijing Dongzhimen Hospital (Beijing, China), and Wuhan Tongji Hospital (Wuhan, China) Recruitment: Participants were recruited via trial sites, local newspapers, community events, websites, and referrals from other medical institutions. Inclusion criteria: smoked at least 10 cigarettes per day for at least 5 years, had expired air carbon monoxide (CO) reading of 9 parts per million (ppm) or greater, were aged 25 to 45 years, and were motivated to stop smoking. Age group chosen: “Due to concerns about adverse events being more likely in older age groups, the sample was limited to adults aged 25 to 45 years. Caution is needed in generalizing the results to older smokers.” Exclusion criteria: pregnancy or breastfeeding, use of stop‐smoking medication during the previous 30 days, ever used ECs for 7 days or longer, history of severe psychiatric illness, unwillingness to use study products, and current diagnosis of cancer or in remission from cancer for less than 1 year.
Participants	Total N = 1068 EC = 409; varenicline = 409; NRT = 250 33.5% female; mean age 33.9 (SD 3.1); mean CPD 16.0 (SD5.3); mean FTND 4.1 (SD 2.1) EC use at baseline: no Motivation to quit: 50.4% had made previous quit attempt; 49.6% had not made previous quit attempt
Interventions	1. EC arm. A cartridge‐based EC product called RELX Wuxian (RELX Technology (30 mg/mL nicotine salt for 2 weeks and 50 mg/mL after that)). Leaflet with product use instructions. Supplied for 12 weeks free. Choice of 3 flavours: mung bean, watermelon, and ice cream. Participants were instructed to use 30 mg/mL cartridges of their preferred flavour for the first 2 weeks and 50 mg/mL cartridges after that, but were asked to continue using 30 mg/mL or reverse to it if they did not like the higher strength. One cartridge was expected to last for 3 days. At the baseline session, 10 cartridges were provided, with an option to request additional supplies at 1‐month and 2‐month follow‐ups (up to 30 cartridges altogether). Participants were instructed to start using their EC ad lib from the next day and stop smoking completely from their TQD onward. 2. Varenicline arm (0.5 mg, once a day for 3 days; 0.5 mg, twice a day for 4 days; and 1 mg, twice a day, after that). Supplied for 12 weeks free. Participants received a 12‐week supply of varenicline (Chantix; Pfizer) and a leaflet with product use instructions. Participants were instructed to take varenicline, 0.5 mg, once per day for the first 3 days, followed by 0.5 mg twice a day for the next 4 days and 1 mg twice a day from day 8, as per the China Clinical Guidelines for Tobacco Cessation. The product was purchased from the manufacturers. Participants were instructed to start using varenicline from the next day and stop smoking completely from their TQD onward. 3. NRT arm: nicotine chewing gum 2 mg (for smokers of 20 cigarettes per day) or 4 mg (> 20 cigarettes per day). Supplied for 12 weeks free Participants received a 12‐week supply of nicotine chewing gum (Johnson & Johnson) product use instruction leaflet. Nicotine gum was selected as the most widely used form of NRT in China. Three boxes containing 105 pieces of the gum each were provided at each monthly contact, with an option to request additional supplies if needed. Participants who smoked up to 20 cigarettes per day received 2 mg nicotine gum, those smoking 20 or more cigarettes per day received 4 mg nicotine gum. Both strengths were provided with the fresh mint flavour. Supplies were bought from the manufacturer. Participants were instructed to use 8 to 12 pieces per day during the first 6 weeks, 4 to 8 pieces per day during weeks 7 and 8, and 2 to 4 pieces per day during the final 4 weeks, asper China Clinical Guidelines for Tobacco Cessation. Participants were instructed to use their NRT from the next day and stop smoking completely from the TQD onward. All groups: At the 3‐month visit, participants were told that they could continue to use their products as needed, but would have to purchase them themselves. A leaflet was provided with information on where the products could be bought. At the last visit, participants received a $60 shopping voucher. All groups: accompanied by minimal behavioural support (an invitation to join a self‐help internet forum). WeChat group for motivational support All participants set up their target quit date (TQD), normally 2 weeks after the baseline visit. All participants received a $40 shopping voucher as compensation for their time and travel. The baseline visit took approximately 30 to 45 minutes. At the last visit, participants received a $60 shopping voucher.
Outcomes	Baseline, 1, 2, 3, 4, 5, 6 months CO measured at all time points Baseline and 6 months – blood pressure (BP) and heart rate
Starting date	Study start date May 2021
Contact information	Nicholas I. Goldenson, Juul Labs, Inc., 1000 F Street NW, Suite 800, Washington, DC 20004, United States. Email: Nicholas.Goldenson@juul.com Funding: This study was funded by Juul Labs, Inc.
Notes	New to 2024 update This study was published then withdrawn. We will monitor to see when this is re‐published. Authors stated: “109 participants, who should be in NRT group, were wrongly placed in the EC group, while another 109 participants, who should be in EC group, were wrongly placed in the NRT group*."*

**Table:** Murray 2020

Study name	Yorkshire Enhanced Stop Smoking (YESS) study: a protocol for a randomized controlled trial to evaluate the effect of adding a personalized smoking cessation intervention to a lung cancer screening programme
Methods	RCT Setting: Yorkshire, UK
Participants	Anticipated recruitment: 1040 people who smoke tobacco cigarettes Participants are aged 55 to 80, registered with a general practitioner (GP) in the Leeds Clinical Commissioning Group area and registered as a current or ex‐smoker in primary care databases Inclusion criteria: Attended a lung health check (LHC) and consent to participate in the Yorkshire Lung Screening Trial (YLST) Have smoked within the last month Have an exhaled carbon monoxide (CO) reading ≥ 6 ppm Have agreed to see an SCP on the mobile unit Exclusion criteria: Any individual who does not have an LDCT scan Unable to provide informed consent
Interventions	Arm 1: enhanced, personalized smoking cessation (SC) support package, including CT scan images. SC support over 4 weeks comprising behavioural support, pharmacotherapy and/or a commercially available e‐cigarette Arm 2: continued standard best practice
Outcomes	Follow‐up contact will be requested at 4 weeks, 3 months, and 12 months, with a 2‐week window to accommodate participant availability. The primary objective is to measure 7‐day point prevalent carbon monoxide (CO)‐validated SC after 3 months. Secondary outcomes include CO‐validated cessation at 4 weeks and 12 months, self‐reported continuous cessation at 4 weeks, 3 months and 12 months, attempts to quit smoking and changes in psychological variables, including perceived risk of lung cancer, motivation to quit smoking tobacco, confidence and efficacy beliefs (self and response) at all follow‐up points.
Starting date	January 2019 and December 2020 with follow‐up data collection ending December 2021. Study completion March 2022.
Contact information	Professor Rachael L Murray; rachael.murray@nottingham. ac.uk
Notes

**Table:** NCT01842828

Study name	Spain‐UK‐Czech E‐cigarette Study (SUKCES)
Methods	Randomized controlled trial, open‐label pilot study Setting: smoking cessation clinics in London, Madrid and Prague Recruitment: via smoking cessation clinics
Participants	220 people who smoke, seeking help to quit Inclusion criteria: 18 years or older Want help to quit Exclusion criteria: Pregnant or breastfeeding Enrolled in other research Currently using EC
Interventions	Standard care plus **4 weeks EC supply** **Standard care** only
Outcomes	CO‐validated continuous abstinence at 4 and 24 weeks post‐TQD Withdrawal symptoms at 1 and 4 weeks post‐TQD EC use EC taste and satisfaction compared to conventional cigarettes Adverse events
Starting date	December 2013
Contact information	Peter Hajek, p.hajek@qmul.ac.uk
Notes

**Table:** NCT02124187

Study name	Smoking cessation and reduction in depression (SCARID)
Methods	3‐arm prospective 12‐m randomized controlled trial investigating efficacy and safety of ECs
Participants	129 participants Inclusion criteria: Diagnosis of major depressive disorder (MDD) (according to DSM‐5 criteria) Smoke ≥ 10 cpd (for at least the past 5 years) Age 18 to 65 years In good general health Unwilling to quit smoking in the next 30 days Exclusion criteria: Use of smokeless tobacco or NRT or other smoking cessation therapies ​ Pregnancy or breastfeeding ​ Current or recent (< 1 yr) past history of alcohol or drug abuse or both ​ Active suicidal intention Other significant comorbidities according to the investigator's clinical assessment (e.g. cancer, acute myocardial infarction, unstable angina, severe cardiac arrhythmia, recent cerebrovascular incident, or severe atherosclerosis)
Interventions	12‐week supply of: **EC 24 mg** nicotine **EC 0 mg** nicotine **Nicotine‐free inhalator**
Outcomes	Follow‐up visits at 4, 8, 12, 24, and 52 weeks Outcome measures: Smoking cessation Smoking reduction (≥ 50% from baseline) Adverse events Quality of life Neurocognitive functioning Participant perceptions and satisfaction with products
Starting date	February 2015 Estimated completion June 2024
Contact information	Pasquale Caponnetto, p.caponnetto@unict.it
Notes

**Table:** NCT02398487

Study name	Head‐to‐head comparison of personal vaporizers versus cig‐a‐like: prospective 6‐month randomized control design study (VAPECIG 2)
Methods	Randomized, parallel‐assignment, open‐label trial Setting: Italy, community
Participants	Estimated enrolment: 200 Inclusion criteria: (People who smoke) in good general health Committed to follow trial procedures Exclude if: Recent vaping history (stopped vaping < 3 months ago) Use of any other form of non‐combustible nicotine‐containing products (chewable tobacco or nicotine replacement therapy) Symptomatic cardiovascular disease Clinical history of asthma and COPD Regular psychotropic medication use Current or past history of alcohol abuse Use of smokeless tobacco or nicotine replacement therapy Pregnancy or breastfeeding
Interventions	Comparison between 2 types of EC; 'personal vaporizers' and 'cig‐a‐like'
Outcomes	24 weeks: Smoking cessation Smoking reduction
Starting date	October 2014. Actual completion December 2015. Authors unable to provide date when study will be available.
Contact information	Riccardo Polosa
Notes

**Table:** NCT02590393

Study name	The role of nicotine and non‐nicotine alkaloids in e‐cigarette use and dependence
Methods	Randomized, parallel‐assignment, double‐blind trial Setting: smoking research clinic, USA Recruitment: volunteers
Participants	Estimated enrolment: 375 Inclusion criteria: Have no known serious medical conditions Are 18 to 65 years old Smoke an average of at least 10 cpd Have smoked at least 1 cumulative year Have an expired air CO reading of at least 10 ppm Are able to read and understand English Exclude if: multiple, related to baseline health status
Interventions	Switch to standard **nicotine EC** use for 8 weeks Switch to **ECs with same nicotine but very low non‐nicotine alkaloid levels** Switch to **ECs with very low nicotine** and non‐nicotine alkaloids
Outcomes	Primary: CO levels at 8 weeks Secondary: EC use EC solution use Cigarette use, at 8 weeks
Starting date	May 2016. Study completion 2022. Emailed author Feb and March 2024 no response.
Contact information	Jed Rose, jed.rose@duke.edu
Notes	"This is not a smoking cessation study; people who smoke will not be asked to quit smoking, and e‐cigarettes will not be used as a medical device or therapy."

**Table:** NCT03113136

Study name	Examination of low wattage and high wattage e‐cigarettes (SWITCH)
Methods	RCT, randomized interventional clinical trial
Participants	Estimated enrolment: 453 participants Inclusion criteria: no quit attempt in the prior 3 months and no plan to quit in the next 3 months; smoked ≥ 5 cigarettes per day for the past year; minimal interest in switching to an alternative product; never purchased or regularly used a tank system, mechanical mod, or advanced personal vaporizer EC, though previous use of cig‐a‐like devices will be allowed Exclusion criteria: unstable or significant medical condition such as respiratory, kidney, or liver disease; unstable or significant psychiatric conditions; history of cardiac event or distress within the past 3 months; and currently pregnant, planning to become pregnant, or breastfeeding; < 18 years
Interventions	Active comparator: low‐wattage e‐cigarette device. The low‐wattage e‐cigarette device will be provided to the participant and they will be instructed to vape ad libitum for 12 weeks and then be assessed at 6 months and 12 months for continued use of device. Active comparator: high‐wattage e‐cigarette device. The high‐wattage e‐cigarette device will be provided to the participant and they will be instructed to vape ad libitum for 12 weeks and then be assessed at 6 months and 12 months for continued use of device. Active comparator: usual brand cigarette. The usual brand of cigarettes will be provided to the participant and they will be instructed to smoke ad libitum for the duration of the study.
Outcomes	Baseline, week 1, week 4, week 8, week 12, week 26, and week 52 Complete change from conventional cigarettes Exhaled carbon monoxide of ≤ 10 ppm Secondary outcomes: EC dependence; EC preference; biomarkers of exposure NNAL; NNN; TNE; nicotine metabolite ratio; nickel and other relevant metals; cadmium and other relevant metals; lead and other relevant metals; 8‐iso‐PGF2a; PGEM; q‐PADDA
Starting date	29 June 2017. Estimated study completion date: 31 August 2022
Contact information	Theodore Wagener, PhD 405‐271‐8001 ext 44350, theodore‐wagener@ouhsc.edu
Notes	New to 2022 update

**Table:** NCT03277495

Study name	Predictors and consequences of combustible cigarette smokers' switch to standardized research e‐cigarettes
Methods	RCT. Randomized, parallel assignment Setting: USA
Participants	Estimated enrolment 120 participants Nicotine EC = 60; placebo EC = 60 Inclusion criteria: ≥ 21 years; ≥ 7 cpd ≥ 1 yr; breath CO ≥ 10 ppm; interested in reducing combustible cigarette use; willing to try EC; attend in‐person assessments for 5 months; English‐speaking; women who are of childbearing age cannot be pregnant and must agree to use an approved form of birth control during the study. Exclusion criteria: current use of any smoking cessation medication or participation in a smoking cessation programme or study; daily EC use; pregnancy; no 2 members of the same household may participate in this study.
Interventions	EC: Standardized Research E‐Cigarette (SREC) Participants will be stratified by sex and use of menthol cigarettes and randomly assigned with a 1:1 allocation ratio to one of two conditions: Active comparator: nicotine SREC. The liquid in the e‐cigarette refills contains nicotine and comes in the following flavours: tobacco, menthol, blueberry, and watermelon. Placebo comparator: placebo SREC The liquid in the e‐cigarette refills does not contain nicotine and comes in the following flavours: tobacco, menthol, blueberry, and watermelon.
Outcomes	3, 4, 5 to 13, 14, 18 weeks Combustible cigarette use Abstinence from combustible cigarettes (defined as no cigarette smoking in the past 7 days) The total number of cigarettes smoked in the 7 days prior to the last assessment CO level. BP. Heart rate. Weight. Self‐report of respiratory symptoms. Fagerstrom Test for Nicotine Dependence
Starting date	Estimated starting date June 2022. Estimated completion date: August 2024
Contact information	Kathleen Diviak, PhD 312‐996‐2327 kdiviak@uic.edu
Notes	New to 2022 update

**Table:** NCT03625986

Study name	Does switching to nicotine containing electronic cigarettes reduce health tisk markers
Methods	RCT. Prospective, parallel‐group, randomized, double‐blind, placebo‐controlled study Setting: Penn State Milton S. Hershey Medical Center, USA
Participants	Estimated enrolment: 240 Inclusion criteria: age 21 to 70 years; smoke regular, filtered cigarettes or machine‐rolled cigarettes with a filter ≥ 5 cpd for ≥ 12 months (CO ≥ 6 ppm at baseline visit); no serious quit attempt in prior month; willing to stop cigarette consumption and switch to an EC and to attend regular visits over a 7‐week period Exclusion criteria: unstable or significant medical condition such as COPD, kidney disease, or liver disease in the past 12 months or severe immune system disorders, uncontrolled mental illness or substance abuse or use of illicit drug/prescription, history of a seizure or seizure medication. Use of any non‐cigarette nicotine delivery product in the past 7 days (including EC); use of hand‐rolled, roll‐your‐own cigarettes; allergy to propylene glycol or vegetable glycerin; pregnancy or breastfeeding
Interventions	EC: Pod The electronic cigarette (e‐cig) used in this study will be the Standardized Research Electronic Cigarette (SREC). The SREC product is a pod‐based device and comprises a replaceable pre‐filled liquid reservoir ("pod") and a rechargeable power supply unit. Arm 1. Experimental: Nicotine‐containing electronic cigarette The experimental group will be provided with and encouraged to use a Standardized Research Electronic Cigarette (SREC) with liquid containing 58 mg/mL nicotine for the duration of 6 weeks. Arm 2. Placebo comparator: Non‐nicotine electronic cigarette The placebo group will be provided with and encouraged to use a Standardized Research Electronic Cigarette (SREC) with liquid containing 0 mg/mL nicotine for the duration of 6 weeks.
Outcomes	3 weeks, 6 weeks, 10 weeks (phone) 3 weeks and 6 weeks after switching NNAL, FEV1, CO, plasma cotinine concentration, Fagerstrom Test for Nicotine Dependence mean total score, cpd, abstinence from cigarettes and other tobacco (not including e‐cigs) CO < 6 ppm, total score on Minnesota Nicotine Withdrawal Scale, EC use days, self‐reported abstinence
Starting date	Actual start date 22 April 2022. Study completion date: 23 February 2024.
Contact information	Jessica Yingst, DrPH 7175314637, jyingst@phs.psu.edu Nicolle Krebs, MS 7175315673, nkrebs@pennstatehealth.psu.edu
Notes	New to 2022 update

**Table:** NCT03700112

Study name	An open‐label, randomized cross‐over study comparing nicotine pharmacokinetics of seven electronic cigarette products and one traditional cigarette across two delivery (10 puff and ad‐libitum) conditions, in healthy adult smokers
Methods	Open‐label, randomized, cross‐over trial Setting and recruitment not specified, New Zealand
Participants	Estimated enrolment: 24 Inclusion criteria: Male or female aged 18 to 60 years of age inclusive BMI between 18 to 35 kg/m^2^ inclusive Healthy based on medical history and screening assessments, in the opinion of the investigator Current smoker of at least 8 cigarettes per day on average Has been smoking for at least 12 months prior to screening. Brief periods of non‐smoking (e.g. up to ~7 consecutive days due to illness, trying to quit, participation in a study where smoking was prohibited) are permitted at the discretion of the investigator. Able to participate, and willing to give written informed consent and comply with study restrictions Exclusion criteria: Clinically relevant medical or psychiatric disorder, in the opinion of the investigator Clinically significant abnormality on screening ECG Sustained blood pressure recordings at screening of < 90 mmHg or > 150 mmHg for systolic blood pressure, or < 50 mmHg or > 90 mmHg for diastolic blood pressure Sustained resting heart rate of > 100 or < 40 beats per minute at screening Positive result for urine drugs of abuse test or alcohol breath test at screening. If a positive urine drug test is observed, and it is believed the positive urine test is due to prescription drugs, the PI should obtain documentation that a) confirms the person's use of the prescribed medication, and b) the prescribed medication will cause a false positive drug test. Clinically significant abnormality in laboratory test results at screening, in the opinion of the investigator Exposure to an investigational drug in a clinical trial within 1 month prior to assessment day 1 Blood or plasma donation of > 500 mL within 1 month prior to assessment day 1 Positive urine pregnancy test at screening or assessment day 1 in women Any clinically significant concomitant disease or condition that could interfere with, or for which the treatment of might interfere with, the conduct of the study, or that would, in the opinion of the investigator, pose an unacceptable risk to the participant in this study
Interventions	JUUL Virginia Tobacco flavoured 5.0% ENDS; consumed using 10 puffs delivery method, ad libitum PMI iQOS Heat sticks ‐ Regular consumed using 10 puffs delivery method, ad libitum Reynolds VUSE Solo ENDS ‐ Original consumed using 10 puffs delivery method, ad libitum Imperial MyBlu ENDS ‐ Original consumed using 10 puffs delivery method, ad libitum Altria MarkTen ENDS ‐ Bold Classic consuming using 10 puffs delivery method, ad libitum MLV PHIX ENDS ‐ Original Tobacco consumed using 10 puffs delivery method, ad libitum NJOY Daily EXTRA ENDS ‐ Rich Tobacco consumed using 10 puffs delivery method, ad libitum Altria Marlboro combustible cigarette ‐ Red consumed using 10 puffs delivery method, ad libitum
Outcomes	Day 48 Outcomes: Nicotine PK parameters calculated from the individual plasma concentrations Exhaled CO Level of user satisfaction measured by Modified Product Evaluation Scale Characterize consumption of 8 x e‐cigarettes/cigarettes products by collecting total number of puffs for each e‐cigarette
Starting date	7 December 2018. Study completion April 2019
Contact information	Study director: Concetta Carbonaro Responsible party: Juul Labs, Inc. Contact details not provided
Notes

**Table:** NCT03962660

Study name	Harm reduction for tobacco smoking with support of tobacco‐replacing electronic nicotine delivery systems (HaRTS‐TRENDS)
Methods	Parallel, randomized controlled trial Setting: USA Recruitment: from prominent Housing First programmes serving chronically homeless people who are often affected by multiple psychiatric, medical and substance‐use disorders. The proposed sample will be recruited from a highly vulnerable and marginalized population in a tight‐knit urban community.
Participants	Estimated enrolment: 94 Inclusion criteria: Having a history of chronic homelessness according to the widely‐accepted federal definition Being a current DESC client living in 1 of DESC's participating permanent supportive housing projects Being between 21 and 65 years of age Being a daily smoker (> 4 cigarettes/day in the past year with a breath CO ≥ 6 ppm or salivary cotinine test at level 1 if CO < 6 ppm) Having adequate English language skills to understand verbal information and communicate in the study Exclusion criteria: Use of other tobacco products besides cigarettes ≥ 9 days in the past month Refusal or inability to consent to participation in research Constituting a risk to the safety and security of other clients or staff
Interventions	Intervention: HaRTS‐TRENDS: 4 individual sessions delivered in the context of the interventionist's pragmatic harm‐reduction mind set paired with a compassionate, advocacy‐oriented 'heart‐set' or style. It comprises the delivery of 4 manualized components, including: a) participant‐led tracking of preferred smoking outcomes, b) elicitation of participants' harm‐reduction goals and their progress toward achieving them, c) discussion of the relative risks of various nicotine delivery systems, d) instruction in using ENDS. Additionally, HaRTS‐TRENDS entails provision of commercially available ENDS. Standard care: The 4‐session, individual standard care control condition entails the well‐documented and evidence‐based 5 As intervention (i.e. Ask about nicotine use, Assess use, Advice to quit smoking, Assist with exploring current smoking/planning smoking cessation, Arrange follow‐up). Part of arranging follow‐up is the recommendation to call the smoking quitline, which can supply additional counselling and nicotine replacement therapy.
Outcomes	Primary outcomes, measured across the 12‐month follow‐up: Biologically‐verified nonsmoking (i.e. self‐reported nonsmoking if corresponding CO measure is < 8) in the past 7 days Urinary concentration of a tobacco‐specific nitrosamine Secondary outcomes, measured across the 12‐month follow‐up: Self‐reported smoking intensity is the mean number of cigarettes participants report smoking per day in the 7 days prior to the assessment. Self‐reported smoking frequency is the number of days participants report smoking in the 7 days prior to the assessment CO level Urinary cotinine FEV1% 10‐item Clinical COPD Questionnaire EQ‐5D‐5L Other outcomes: Smoking craving Side effects of ENDS
Starting date	9 May 2019
Contact information	Tatiana M Ubay, tatiubay@uw.edu
Notes

**Table:** NCT04003805

Study name	Biomarkers of exposure and effect in standardized research e‐cigarette (SREC) users
Methods	Design: RCT Setting: USA
Participants	Estimated enrolment: 125 Inclusion criteria: 18 to 65 smokers willing to stop smoking and completely switch to EC or medicinal nicotine ≥ 5 cigarettes daily and not using any other nicotine or tobacco product; biochemically confirmed Smoking daily for at least 1 year and no serious quit attempts Exclusion criteria: Regular tobacco or nicotine product use other than cigarettes Currently using NRT or other tobacco cessation products Significant immune system disorders, respiratory diseases, kidney or liver diseases or any other medical disorders that may affect biomarker data; taking anti‐inflammatory medications; unstable health conditions; unstable mental health; excessive drinking; positive toxicology screen for illicit any drugs: pregnant or breastfeeding For a full list see NCT record.
Interventions	EC: Standardized Research E‐cigarette (SREC) Arm 1: Experimental: Switching from Smoking Cigarettes to E (SREC) The device operates at a single output voltage (3.30 ± 0.05 V) and uses sealed disposable 3 mL cartridges with tobacco‐flavoured e‐liquid (~350 puffs/cartridge). The concentration of nicotine in e‐liquid is 15 mg/mL, and the vehicle composition is 50:50 propylene glycol and glycerin. The device uses a battery that can be recharged via a micro USB port. Arm 2: Experimental: Switching from smoking cigarettes to nicotine mini‐lozenge We will use commercially available nicotine mini‐lozenges containing 2 or 4 mg nicotine/lozenge (Nicorette, manufactured by GlaxoSmithKline). Dose will be determined per instructions on the package (e.g. if smoking within 30 minutes upon awakening, then 4 mg dose will be prescribed).
Outcomes	1 year 4 and 8 weeks for formaldehyde‐DNA adducts and oxidative DNA adduct 8‐oxo‐dG in DNA Biomarkers: TNE, NNAL, NNN, PneT, mercapturic acids HMPMA, 2‐HPMA, 3‐HPMA, formaldehyde‐DNA adducts, oxidative DNA adduct 8‐oxo‐dG in DNA, NNN and nornicotine, HPB‐releasing DNA adducts cpd, product use (EC and nicotine lozenges), CC avoidance
Starting date	Actual start date: 11 May 2022. Estimated study completion date: January 2025
Contact information	Hanna Vanderloo, RN, MSN 612.624.4983, hannav@umn.edu
Notes	New to 2022 update

**Table:** NCT04058717

Study name	Low nicotine cigarettes plus electronic cigarettes
Methods	RCT: randomized, parallel‐group assignment, 2 x 2 factorial design Setting: USA
Participants	Estimated enrolment 240 participants Inclusion criteria: Meet lifetime diagnostic criteria for a current or lifetime unipolar or bipolar mood disorder Smoke > 5 cigarettes per day for at least the prior 12 months No serious cigarette smoking quit attempt or use of any FDA‐approved smoking cessation medication in the prior 30 days; no plans to quit smoking within the next 3 weeks Willing to both switch to a different type of cigarette that may contain a different amount of nicotine and to try an EC to substitute for some of their cigarettes Exclusion criteria: Unstable or significant medical condition in the past 3 months Uncontrolled mental illness or substance abuse, or inpatient treatment for these in the past 6 months or current suicide risk Use of any non‐cigarette nicotine delivery product or EC Use illegal drugs/prescription drugs Pregnancy or breastfeeding For a full list see NCT record.
Interventions	EC: type of EC not reported Arm 1 Experimental: NNC cigarettes + high nicotine‐containing e‐cigarette. Participants are provided with normal nicotine content (NNC) cigarettes (11.6 mg nicotine/cigarette) plus e‐cigarette with high nicotine e‐liquid. Arm 2 Experimental: NNC cigarettes + zero nicotine containing e‐cigarette. Participants are provided with normal nicotine content (NNC) cigarettes (11.6 mg nicotine/cigarette) plus e‐cigarette with zero nicotine e‐liquid. Arm 3 Experimental: VLNC cigarettes + high nicotine‐containing e‐cigarette. Participants are provided with very low nicotine content (VLNC) cigarettes (0.2 mg nicotine/cigarette) plus e‐cigarette with high nicotine e‐liquid. Arm 4 Experimental: VLNC cigarettes + zero nicotine‐containing e‐cigarette. Participants are provided with very low nicotine content (VLNC) cigarettes (0.2 mg nicotine/cigarette) plus e‐cigarette with zero nicotine e‐liquid.
Outcomes	4, 8, 12 and 16 weeks Urinary NNAL (4‐(methylnitrosamino)‐1‐(3‐pyridyl)‐1‐butanol) Exhaled carbon monoxide Kessler‐6 score measure of serious psychological distress Penn State Cigarette Dependence Index Penn State Electronic Cigarette Dependence Index Cigarette abstinence. No cigarette use in the past 7 days and exhaled carbon monoxide < 6 ppm
Starting date	Actual start date: 1 June 2021. Estimated completion date: May 2024.
Contact information	Nicolle Krebs, MS 717‐531‐5673, nkrebs@pennstatehealth.psu.edu Jonathan Foulds, PhD 717‐531‐3504, jfoulds@psu.edu
Notes	New to 2022 update

**Table:** NCT04063267

Study name	Electronic cigarettes as a harm reduction strategy in individuals with substance use disorder
Methods	Parallel‐group, randomized trial Recruitment/setting: Not specified
Participants	Estimated enrolment: 240 Inclusion criteria: Smokes at least 10 cpd Meet DSM‐V AUD and/or OUD within the past year, interested in reducing cpd Able to provide consent Use a cell phone, are willing/able to receive and respond to daily text messages about their cigarette use and e‐cigarette use on their cell phone Provide 1 additional contact, and are willing to use an e‐cigarette for 3 weeks Exclusion criteria: Pregnant and/or breastfeeding (self‐reported) Currently using smoking cessation medications (including other forms of NRT, bupropion, or varenicline) Enrolled in a smoking cessation programme or another cessation trial Have used an e‐cigarette in the past 14 days Have used any other tobacco products (pipe, cigar, cigarillos, snuff, chewing tobacco, rolling tobacco, or hookah/shisha) in the past 30 days Report having a history of asthma, other airways diseases, or heart disease
Interventions	**E‐cigarettes arm:** Participants will be encouraged to substitute e‐cigarettes for combustible cigarettes in order to reduce nicotine withdrawal symptoms. **Nicotine Replacement Therapy****arm:** Nicotine patches and gum to last them the first week based on their baseline recorded smoking. Participants will be advised to use both a 21 mg nicotine patch and 4 mg nicotine for cravings.
Outcomes	Proportion of participants who achieve 50% reduction in cpd at 3 weeks
Starting date	Start date: October 2020 Estimated completion date 30 June 2024
Contact information	NYU Langone Health, Scott.Sherman@nyulangone.org
Notes

**Table:** NCT04090879

Study name	Low nicotine content cigarettes in vulnerable populations: affective disorders
Methods	RCT Setting: USA
Participants	Estimated enrolment 232 participants Inclusion criteria Between 21 and 70 years old Must have current diagnosis of an affective disorder Exclusion criteria Being without an affective disorder
Interventions	EC: Not stated 'e‐cigarette...commercially available device' Either normal nicotine content cigarettes (15.8 mg/g) or reduced nicotine content cigarettes (0.4 mg/g) 1) Altering the nicotine content of the tobacco research cigarette E‐cigarettes 1) Altering the availability of e‐cigarettes 2) Altering option to personalize the e‐liquid in the e‐cig condition Use assigned product for 16 weeks Arm 1: RC 1 (Research Cigarettes) only. Either normal nicotine content cigarettes (15.8 mg/g) or reduced nicotine content cigarettes (0.4 mg/g) Arm 2: RC 2 only Arm 3: Research Cigarettes #2 plus e‐cigarettes #1 (participants receive tobacco flavour only) Arm 4: Research Cigarettes #2 plus e‐cigarettes #2 (participants can choose among varying flavours)
Outcomes	16 weeks Total cpd, cigarette demand assessed by behavioural economics‐based purchase tasks, craving, withdrawal, psychiatric symptoms, breath carbon monoxide (CO), biomarkers of tobacco toxicant exposure, brain function and structure, and airway inflammation (fractional nitric oxide concentration in exhaled breath (FeNO))
Starting date	Study start date: 18 September 2019. Estimated completion date: July 2023
Contact information	Shirley Plucinski 9788752361, shirley.plucinski@uvm.edu
Notes	New to 2022 update

**Table:** NCT04092101

Study name	Low nicotine content cigarettes in vulnerable populations: opioid use disorder
Methods	RCT Recruitment: Daily smokers who are receiving methadone or buprenorphine treatment will be recruited at the University of Vermont and Johns Hopkins University. Setting: University of Vermont and Johns Hopkins University, USA
Participants	Estimated enrolment: 310 Inclusion criteria: Maintained on opioid medication 21 to 70 years old
Interventions	EC: type not specified, 'normal nicotine content cigarette and ... a reduced nicotine content cigarette' Cigarettes with varying nicotine content E‐cigarettes Arm 1 Cigarettes with varying nicotine content. Altering the nicotine content of the tobacco research cigarette. Research Cigarettes #1 Arm 2: Cigarettes with varying nicotine content. Altering the nicotine content of the tobacco research cigarette. Research Cigarettes #2. Arm 3: Cigarettes with varying nicotine content. Altering the nicotine content of the tobacco research cigarette. E‐Cigarettes. 1) Altering the availability of e‐cigarettes; 2) Altering option to personalize the e‐liquid in the e‐cig condition Research Cigarettes #2 plus E‐cigarettes #1 Arm 4: Cigarettes with varying nicotine content. Altering the nicotine content of the tobacco research cigarette E‐Cigarettes. 1) Altering the availability of e‐cigarettes; 2) Altering option to personalize the e‐liquid in the e‐cig condition. Research Cigarettes #2 plus E‐cigarettes #2
Outcomes	16 weeks Outcome measures include total cpd, cigarette demand assessed by behavioural economics‐based purchase tasks, craving, withdrawal, psychiatric symptoms, breath carbon monoxide (CO), biomarkers of tobacco toxicant exposure, brain function and structure, and airway inflammation (fractional nitric oxide concentration in exhaled breath (FeNO))
Starting date	Start date: September 2019. Estimated completion date: Jan 2024
Contact information	Shirley Plucinski 9788752361, shirley.plucinski@uvm.edu
Notes	New to 2022 update

**Table:** NCT04092387

Study name	Low nicotine content cigarettes in vulnerable populations: women of reproductive age
Methods	RCT Setting: Johns Hopkins University and the University of Vermont, USA
Participants	Estimated enrolment 246 Inclusion criteria: Female 21 to 44 years old
Interventions	EC: type not specified, 'normal nicotine content cigarette and ... a reduced nicotine content cigarette' Cigarettes with varying nicotine content E‐Cigarettes Arm 1: Cigarettes with varying nicotine content. Altering the nicotine content of the tobacco research cigarette. Research Cigarettes #1 Arm 2: Cigarettes with varying nicotine content. Altering the nicotine content of the tobacco research cigarette. Research Cigarettes #2 Arm 3: Cigarettes with varying nicotine content. Altering the nicotine content of the tobacco research cigarette E‐Cigarettes. 1) Altering the availability of e‐cigarettes; 2) Altering option to personalize the e‐liquid in the e‐cig condition Research Cigarettes #2 plus E‐cigarettes #1 Arm 4: Cigarettes with varying nicotine content. Altering the nicotine content of the tobacco research cigarette E‐Cigarettes. 1) Altering the availability of e‐cigarettes; 2) Altering option to personalize the e‐liquid in the e‐cig condition. Research Cigarettes #2 plus E‐cigarettes #2
Outcomes	16 weeks Participants will be asked to use only their assigned study products for 16 weeks. Outcome measures include total cpd, cigarette demand assessed by behavioural economics‐based purchase tasks, craving, withdrawal, psychiatric symptoms, breath carbon monoxide (CO), biomarkers of tobacco toxicant exposure, brain function and structure, and airway inflammation (fractional nitric oxide concentration in exhaled breath (FeNO))
Starting date	Start date: 18 September 2019. Estimated study completion date: Jan 2024
Contact information	Catherine Markesich 8026569981, cmarkesi@uvm.edu
Notes	New to 2022 update

**Table:** NCT04218708

Study name	Electronic cigarettes as a harm reduction strategy among people living with HIV/AIDS
Methods	RCT Setting: NYU Langone Health, USA
Participants	Estimated enrolment 120 Inclusion criteria: Current Combustible Cigarette (CC) smokers (more than 5 packs in a lifetime; smokes 4 or more days/week), at least 10 cigarettes per day on days they smoke CC Motivated to quit smoking (at least a 5 on a 10‐point Likert scale) Be willing to use an e‐cigarette or NRT for 12 weeks Exclusion criteria: Pregnancy or breastfeeding Stated diagnosis of any medical condition (angina/heart disease) precluding use of nicotine patch or gum, or by self‐report in screening questionnaire. Reporting a history of severe or untreated cardiopulmonary disease such as asthma or emphysema Reporting using NRTs or e‐cigarettes within the last 30 days Have untreated/are undergoing current treatment for psychiatric illness or cognitive impairment
Interventions	EC: Pod. NIDA Standardized Research E‐cigarettes (SREC) (15 mg/mL nicotine in tobacco flavour) Arm 1: counselling + nicotine replacement therapies NRT A research assistant (RA) trained in motivational interviewing and qualitative methods will support the PI to deliver counselling sessions and conduct interviews. Briefly, during each visit, with help of the RA, participants will provide exhaled CO and saliva cotinine test, and complete surveys in REDCAP using a tablet, allowing programmed logic checks and skip patterns to minimize burden. The RA will also deliver brief motivational counselling tailored to the participant's readiness to quit and arm in the study (NRT). Participants will also receive their NRT to last them to the following visit based on their baseline smoking. Arm 2: Counselling + Standardized Research E‐cigarettes (SREC) Participants in the SREC arm to practice using the SREC and RA to give them instructions to return with their SREC and used refill tanks on every visit. A research assistant (RA) trained in motivational interviewing and qualitative methods will support the PI to deliver counselling sessions and conduct interviews. Briefly, during each visit, with help of the RA, participants will provide exhaled CO and saliva cotinine test, and complete surveys in REDCAP using a tablet, allowing programmed logic checks and skip patterns to minimize burden. The RA will also deliver brief motivational counselling tailored to the participant's readiness to quit and arm in the study (SREC). Participants will also receive their SREC to last them to the following visit based on their baseline smoking.
Outcomes	Week 1, 2, 4, 6, 8, 12 Change in cigarettes per day (cpd). Smoking reduction will be measured by a combination of self‐report, text message data and changes in CO and saliva cotinine between baseline and end of treatment. Assessing differences in nicotine withdrawal symptoms Assessing differences in e‐cigarette dependency Assessing differences in nicotine use Assessing differences in use of substance use Assessing differences in side effects associated with e‐cigarette use
Starting date	Study start date: 17 June 2021. Estimated study completion date: October 2024.
Contact information	Omar El Shahawy, MD 1‐646‐501‐2587, omar.elshahawy@nyulangone.org
Notes	New to 2022 update

**Table:** NCT04238832

Study name	Impact of non‐cigarette tobacco product formulation on reinforcement value and use in current smokers Short title: Salt‐based e‐cigarette
Methods	RCT Setting: USA, South Carolina
Participants	30 participants Inclusion criteria: Daily cigarette smoker Interested in using non‐cigarette tobacco product Have a smartphone that can receive text messages and has access to the internet or have an email account they check daily (necessary for daily diary completion) Exclusion criteria: Additional tobacco use criteria Additional medical criteria
Interventions	Salt‐base nicotine Free‐base nicotine
Outcomes	Most preferred product (time frame: Lab visit 2, occurring approximately 1 week after the initial screening/baseline visit) Participants complete a preference assessment in which they choose between the salt liquid, free‐base liquid, or a traditional cigarette in a series of trials. The outcome of this assessment is the product chosen most often by each participant. Cigarettes per day (time frame: Week 2 of study) The average number of cigarettes smoked per day during the 1‐week sampling period Biomarkers (i.e. expired CO, cotinine) will corroborate self‐reported indices of use.
Starting date	23 June 2020. Estimated completion: August 2021
Contact information	Tracy Smith, smithtra@musc.edu
Notes

**Table:** NCT04452175

Study name	Official title: Cigarette consumption after switchinG to high or low Nicotine strENght E‐cigaretteS In Smokers with schizophrenia spectrum disorders: a 12‐month randomized, double‐blind multicentre trial Brief title: Cigarette consumption after switchinG to high or low nicotine strENght E‐cigaretteS In Smokers with schizophrenia (GENESIS) NB: The GENESIS protocol (NCT04452175) now incorporates SCARIS protocol, NCT01979796. Antismoking effects of electronic cigarettes in subjects with schizophrenia and their potential influence on cognitive functioning: design of a randomized trial. Smoking Cessation And Reduction In Schizophrenia (The SCARIS Study). clinicaltrials.gov/show/NCT01979796
Methods	RCT Multicentre: Italy, Russia, Ukraine, UK Collaborators: Juul Labs, Inc. St. Petersburg State Pavlov Medical University Bashkir State Medical University Ukrainian Institute on Public Health Policy University of Surrey Eclat Srl
Participants	Estimated enrolment: 260 Inclusion criteria: Adult (> 18 yrs) Regular smoking (> 10 cigarettes a day; for at least 1 year) Exhaled breath CO (eCO) level > 7 ppm Not currently attempting to quit smoking or wishing to do so in the next 30 days; this will be verified at screening by the answer "NO" to the question "Do you intend to quit in the next 30 days?" Schizophrenia spectrum disorder diagnosis (schizophrenia, delusional disorder, schizoaffective disorder, personality disorder, schizoid personality disorder, etc) by DSM‐V criteria Understand and provide informed consent Able to comply with all study procedures Exclusion criteria: Institutionalized patients Acute decompensation of schizophrenia spectrum disorder symptoms within the past month Change in antipsychotic treatment within the past month No recent history of hospitalization for any serious medical condition within 3 months prior to screening, as determined by the investigator Myocardial infarction or angina pectoris within 3 months prior to screening, as determined by the investigator Current poorly‐controlled asthma or COPD Pregnancy, planned pregnancy, or breastfeeding. Any female participant who becomes pregnant during this study will be withdrawn. People who have a significant history of alcoholism or drug/chemical abuse within 12 months prior to screening, as determined by the investigator Accepting to take part in a smoking cessation programme People who regularly use any recreational nicotine (e.g. e‐cigarettes) or tobacco product (e.g. tobacco heated products, oral smokeless) other than their own cigarettes within 30 days of screening People who have used smoking cessation therapies (e.g. varenicline, bupropion, or NRT) within 30 days of screening People who are still participating in another clinical study (e.g. attending follow‐up visits) or who have recently participated in a clinical study involving administration of an investigational drug (new chemical entity) within the past 3 months People who have, or who have a history of, any clinically significant neurological, gastrointestinal, renal, hepatic, cardiovascular, psychiatric, respiratory, metabolic, endocrine, haematological or other major disorder that, in the opinion of the investigator or their appropriately qualified designee, would jeopardize the safety of the participant or impact on the validity of the study results
Interventions	Experimental: high 5%. Intervention: JUUL e‐cigarette Active comparator: low 1.7%. Intervention: JUUL e‐cigarette
Outcomes	Primary outcomes: Rates of participants with continuous smoking abstinence at 6 months; time frame: 24 weeks Self‐reported continuous smoking abstinence at 6 months from the previous visit, biochemically verified by exhaled CO measurements of ≤ 7 ppm Secondary outcomes Rates of participants with continuous smoking abstinence at 12 months (time frame: 52 weeks) Rates of participants with continuous smoking reduction at 6 months (time frame: 24 weeks) Rates of participants with continuous smoking reduction at 12 months (time frame: 52 weeks) Proportion of AEs (time frame: 24 weeks) Absolute change in PANSS (time frame: 24 weeks) Absolute change in mCEQ (time frame: 24 weeks) Absolute change in Chester Step Test‐derived values (time frame: 24 weeks) Change in App‐derived endpoints (self‐rated mental health SRMH) (time frame: 24 weeks)
Starting date	Actual start date 30 October 2021. Estimated study completion: February 2023 (NCT record update posted on 23 May 2022)
Contact information	Pasquale Caponnetto, p.caponnetto@unict.it
Notes

**Table:** NCT04465318

Study name	Electronic cigarettes as a harm reduction strategy among patients with COPD
Methods	RCT Setting: USA
Participants	Estimated enrolment 120 Inclusion criteria: An ambulatory ICD‐10 code for COPD in the last 12 months and a COPD Assessment Tool (CAT) score on the screening ≥ 10 Aged 21 to 75 Current CC smoker (more than 5 packs in a lifetime; smokes 4 or more days/week, smokes at least 10 cigarettes per day on days they smoke CC) Motivated to quit smoking (at least a 5 on a 10‐point Likert scale) Exclusion criteria: A CAT score > 30 representing severe COPD Pregnancy Diagnosis of any medical condition Reporting using NRTs or e‐cigarettes within the last 30 days
Interventions	EC: NJOY daily e‐cigarettes Arm 1: EC + Counselling E‐cigarette (EC). NJOY daily e‐cigarettes are self‐contained and non‐refillable. Each DAILY provides approximately 300 puffs, comparable to a full pack of cigarettes. Behavioural: Smoking harm reduction counselling sessions Counselling will cover health education, social support issues, and motivational enhancement to improve self‐efficacy while addressing other aspects know to contribute to smoking among people with COPD (e.g. tips on dealing with depression) Arm 2: Nicotine Replacement Therapy (NRT) Participants in the NRT arm will receive 21 mg nicotine patch (for those with cpd ≥ 20) or 14 mg nicotine patch (for those with cpd < 20) + 4 mg nicotine gum. Behavioural: Smoking harm reduction counselling sessions as for Arm 1
Outcomes	12 weeks Primary outcome measures: Number of participants who achieve 50% reduction in cigarettes per day (cpd) Average change in score on the mMRC Dyspnoea Scale Secondary outcome measures: Number of participants who reported satisfaction with use of ECs Number of participants who reported additional use of tobacco products and/or marijuana Change in score of COPD Assessment Test (CAT) Change in score of Clinical COPD Questionnaire (CCQ)
Starting date	Start date: 9 November 2020. Estimated study completion date: 31 March 2023
Contact information	Scott E. Sherman, MD, MPH NYU Langone Health Elizabeth Stevens, PhD, MPH NYU Langone Health
Notes	New to 2022 update

**Table:** NCT04521647

Study name	Effects of menthol in e‐cigarettes on smoking behaviors
Methods	Randomized cross‐over Setting: Connecticut Mental Health Center, USA
Participants	Estimated enrolment 85 Inclusion criteria: ≥ 21 years, use combustible cigarettes Exclusion criteria: none
Interventions	EC: type not stated Arm 1: menthol flavour. Participants will receive 5% nicotine in an EC. Participants will receive 2 nicotine concentrations via EC. Each exposure will be 10 3‐sec puffs and ad libitum use. Arm 2: tobacco flavour. Participants will receive 5% nicotine in an EC. Participants will receive 2 nicotine concentrations via EC. Each exposure will be 10 3‐sec puffs and ad libitum use.
Outcomes	Baseline and 2, 5, 15, 30, 45, 60, 90, 120, and 180 minutes after nicotine exposure (plasma nicotine levels), 2 weeks (CO), 3 weeks, 5 weeks (BP and heart rate) Primary outcomes: cigarette craving; plasma nicotine levels; carbon monoxide Secondary outcomes: EC craving; irritation/harshness; liking of EC; coolness; nicotine withdrawal; stimulation; EC use; cigarette use. Other outcomes measured: heart rate, blood pressure, pulse oximetry
Starting date	Study start date: 1 November 2020. Completion date: 25 July 20253
Contact information	Asti Jackson, PhD 4752414904, asti.jackson@yale.edu
Notes	New to 2022 update

**Table:** NCT04649645

Study name	International randomized controlled trial evaluating changes in oral health in smokers after switching to combustion‐free nicotine delivery systems (SMILE)
Methods	RCT Setting: multicentre: Italy, Moldova, Poland, UK, and Indonesia
Participants	Estimated enrolment 606 participants Inclusion criteria: Demonstrate understanding of the study and willingness to participate in the study by providing a signed written informed consent Healthy, not taking regular medications for chronic medical conditions Adults, age at least 18 years old Presence of at least 10 natural anterior teeth in total (cuspid to cuspid, lower and upper jaw) Presence of at least 18 'scorable' teeth with scorable facial and lingual surfaces. Teeth that are grossly carious, orthodontically banded, exhibiting general cervical abrasion and/or enamel abrasion, and third molars will not be included in the tooth count. Willingness and ability to comply with the requirements of the study, including installing an APP on their digital device, e.g. smartphone or tablet For Arms A and B, participants have to be: Regular smokers, defined as: smoked for at least 5 consecutive years prior to screening. Smoked > 10 and < 30 cigarettes per day (cpd) with an exhaled breath carbon monoxide (CO) level ≥ 7 ppm at screening Willing to regularly use any nicotine or tobacco product other than their own conventional cigarette brand within 14 days prior to screening Willing to change to use of study products or, if randomized to Arm A, continuing to use their own brand of conventional cigarettes for the whole duration of the study For Arm C, participants have to be: Never‐smokers, defined as: never smoked or who have smoked < 100 cigarettes in their lifetime and none in the 30 days prior to screening with an exhaled breath CO level < 7 ppm at screening Willing to not smoke or use any form of tobacco or nicotine‐containing products for the whole duration of the study Exclusion criteria: Pregnancy Presence of extensive crown or bridge work, dental implants, and/or rampant decay (per investigator/examiner discretion) Significant oral soft tissue pathology or any type of gingival overgrowth, other than plaque‐induced gingivitis and mild periodontitis (Stage I) Moderate‐to‐severe periodontitis (Stage II, III and IV) based on 2017 World Workshop on the Classification of Periodontal and Peri‐Implant Diseases and Conditions, which require: Detectable interdental Clinical Attachment Loss (CAL) ≥ 3 mm at ≥ 2 non‐adjacent teeth. Buccal or oral CAL ≥ 3 mm with pocketing ≥ 5 mm detectable at ≥ 2 teeth Removable dentures or fixed and removable orthodontic appliance (except fixed lingual wires) Significant history of alcoholism or drug abuse (other than tobacco/nicotine) within 24 months prior to screening, as determined by the investigator A course of treatment with any medications or substances (other than tobacco/nicotine) which: interfere with the cyclo‐oxygenase pathway (e.g. anti‐inflammatory drugs including aspirin and ibuprofen) within 3 days prior to each visit or are known to have antibacterial activity (e.g. antibiotics) within 7 days prior to each visit
Interventions	Standard arm (Arm A): own tobacco cigarette brand Intervention arm (Arm B): combustion‐free nicotine delivery system (C‐F NDS) Control arm (Arm C): no smoking or use of any nicotine/tobacco products
Outcomes	Oral health parameters and teeth appearance, comparing short‐ and long‐term impact on periodontal health between smokers continuing with conventional cigarette smoking, those switching to combustion‐free nicotine delivery systems (C‐F NDS), and never‐smokers over 18 months
Starting date	Not yet recruiting (last updated February 2021) Estimated study start date: Feb 2021. Estimated primary completion date: Feb 2023. Estimated completion April 2023.
Contact information	Principal investigator: Antonio Pacino, DDS, Addendo srl, Catania, Italy info@addendo.net
Notes

**Table:** NCT04708106

Study name	Characterization of product use in smokers switching from cigarettes to a RELX electronic nicotine delivery system Setting: USA
Methods	Design: RCT, multicentre, open‐label, parallel‐cohort study
Participants	Estimated 200 Inclusion criteria: Provides voluntary consent to participate in the study as documented on the signed informed consent form (ICF) Is 22 to 65 years of age, inclusive, at the time of consent Is willing to comply with the requirements of the study Reports typically smoking 5 or more combustible cpd at screening Has been a daily smoker for at least 12 months prior to screening. Brief periods of non‐smoking (e.g. up to ~7 consecutive days due to illness, trying to quit, participation in a study where smoking was prohibited) ≥ 56 days prior to screening will be permitted at the discretion of the investigator. Has a positive urine cotinine test (≥ 200 ng/mL) at screening and test visit 1 Has an eCO value > 10 ppm at screening and test visit 1 Has daily access to a cell phone for daily product use reporting If female, meets one of the following criteria: If of childbearing potential ‐ agrees to use one of the accepted contraceptive regimens from at least 30 days prior to the first product use and during the study. An acceptable method of contraception includes one of the following: Abstinence from heterosexual intercourse Hormonal contraceptives (birth control pills, injectable/implant/insertable hormonal birth control products, transdermal patch) Intrauterine device (with or without hormones) OR agrees to use a double barrier method (e.g. condom and spermicide) during the study If a female of non‐childbearing potential ‐ should be surgically sterile (i.e. has undergone complete hysterectomy, bilateral oophorectomy, or tubal ligation) or in a menopausal state (at least 1 year without menses), as confirmed by follicle stimulating hormone (FSH) levels. Exclusion criteria: Has a history or presence of clinically significant uncontrolled gastrointestinal, renal, hepatic, neurologic, haematologic, endocrine, oncologic, urologic, pulmonary, immunologic, psychiatric, or cardiovascular disease, or any other condition that, in the opinion of the investigator, would jeopardize the safety of the subject or impact the validity of the study results Has a clinically significant abnormal finding on the physical examination, medical history, vital signs, electrocardiogram (ECG), or clinical laboratory results, in the opinion of the investigator Has a positive test for human immunodeficiency virus (HIV), hepatitis B surface antigen (HBsAg), or hepatitis C virus (HCV) at screening Has a positive COVID‐19 test at screening or during the study Has had an acute illness (e.g. upper respiratory infection, viral infection) within 14 days prior to test visit 1 Has a fever (> 100.5°F) at screening or test visit 1 Has a body mass index (BMI) greater than 40.0 kg/m^2^ or less than 18.0 kg/m^2^ at screening Has a systolic blood pressure < 90 mmHg or > 150 mmHg, diastolic blood pressure < 40 mmHg or > 95 mmHg, or heart rate < 40 bpm or > 99 bpm at screening Has a post‐bronchodilator forced expiratory volume in 1 second:forced vital capacity (FEV1:FVC) ratio < 0.7 and FEV1 < 50% of predicted at screening Has a post‐bronchodilator FEV1 increase ≥ 12% and > 200 mL from pre‐ to post‐bronchodilator at screening Has used an ENDS product on > 7 days during each of the 3 months prior to screening or any use from screening to test visit 1 other than as may be required for this study Reports use of a very‐low‐nicotine content cigarette (e.g. Moonlight, Spectrum, VLN) as usual brand Has used nicotine‐containing products other than manufactured cigarettes (e.g. ENDS products (e‐cigarettes), roll‐your‐own cigarettes, bidis, snuff, nicotine inhaler, pipe, cigar, chewing tobacco, nicotine patch, nicotine spray, nicotine lozenge, or nicotine gum) within 14 days prior to test visit 1 Has used any products for the purpose of smoking cessation, including, but not limited to, nicotine replacement therapies, varenicline (Chantix), or bupropion (Zyban) from 30 days prior to screening through the duration of the study Is a self‐reported puffer (i.e. draws smoke from the cigarette into the mouth and throat but does not inhale) Is postponing a planned smoking quit attempt in order to participate in the study Has a history of drug or alcohol abuse within 12 months prior to screening, as determined by the investigator Is allergic to PG or glycerin Has a positive urine drug or alcohol breath test at screening or test visit 1. At the discretion of the investigator, a subject testing positive for tetrahydrocannabinol may be permitted to participate if the subject reports use by routes other than inhalation. If female, the subject is pregnant, breastfeeding, or intends to become pregnant from screening through the duration of the study Has been treated for depression, diabetes, asthma, emphysema, or chronic obstructive pulmonary disease within 12 months prior to test visit 1 Has previously been diagnosed with any form of cancer, except for basal cell or squamous epithelial carcinomas of the skin that have been resected at least 12 months prior to screening 1 Has a planned surgery that would occur during study participation Has participated in a previous clinical study for an investigational drug, device, biologic, or tobacco product within 30 days prior to test visit 1 Is or has a first‐degree relative (e.g. spouse, parent, sibling, or child) who is a current or former employee of a tobacco or ENDS manufacturer or is a named party or class representative in litigation with the tobacco or ENDS industry Is or has a first‐degree relative (e.g. spouse, parent, sibling, or child) who is a current employee of the clinic site Is or has a first‐degree relative (e.g. spouse, parent, sibling, or child) who is a current employee of the sponsor Has previously taken part in (from completion of any baseline measurements), has been withdrawn from, or has completed this study In the opinion of the investigator, the subject should not participate in this study.
Interventions	RELX ENDS tobacco flavour ad libitum use of the RELX ENDS tobacco flavour product RELX ENDS menthol flavour ad libitum use of the RELX ENDS menthol product Ad libitum use of the RELX ENDS tobacco and menthol flavour products
Outcomes	Primary outcomes: Weekly RELX ENDS product use; time frame: 56 days. Self‐reported number of RELX ENDS pods started each week Daily number of cigarettes smoked; time frame: 56 days. Self‐reported number of cigarettes smoked daily by study week Number of puffs from the RELX ENDS each day; time frame: 56 days. Self‐reported number of puffs from the RELX ENDS daily by study week (0, < 100, ≥ 100 per day) Secondary outcomes: Biomarkers of exposure measured in blood; time frame: baseline, day 28, and day 56; change in carbon monoxide concentration in the blood Biomarkers of tobacco exposure measured in urine; time frame: baseline, day 28, and day 56; change in creatinine‐adjusted NNAL, NNN, 3‐HPMA, CEMA, HMPMA, S‐PMA, HEMA, 1‐OHP, o‐toluidine, nicotine equivalents, and propylene glycol excreted in urine Subjective effects as measured by the Penn State (Electronic) Cigarette Dependence Index (PS(E)CDI); time frame: baseline, day 14, day 28, day 42, and day 56. Change in product dependence as measured by the PSCDI/PS(E)CDI total score. Total scores may range for 0 to 20, with higher levels of dependence associated with higher scores. Subjective effects as measured by the Cough Questionnaire; time frame: baseline, day 14, day 28, day 42, and day 56. Change in self‐reported cough symptoms as measured by responses to the Cough Questionnaire Subjective effects as measured by the Questionnaire of Smoking Urges‐Brief (QSU‐Brief); time frame: baseline, day 14, day 28, day 42, and day 56. Change in smoking urge as measured by the QSU‐Brief factor 1 and factor 2 scores. Questionnaire responses are measured on a Likert scale range of 1 (not at all) to 7 (extremely). Subjective effects as measured by the Minnesota Tobacco Withdrawal Scale‐Revised (MTWS‐R); time frame: baseline, day 14, day 28, day 42, and day 56. Change in withdrawal symptoms as measured by the MTWS‐R total score, which includes the DSM‐5 and craving items from the Minnesota Tobacco Withdrawal Scale. Questionnaire responses are measured on a Likert scale range of 0 (none) to 4 (severe)). Subjective effects as measured by the Modified Product Evaluation Scale (mPES); time frame: baseline, day 14, day 28, day 42, and day 56. Change in product assessments as measured by mPES satisfaction, psychological reward, aversion, and relief subscale scores. Questionnaire responses are measured on a Likert scale range of 1 (not at all) to 7 (extremely). Subjective effects as measured by the Future Intent to Use Questionnaire; time frame: baseline, day 14, day 28, day 42, and day 56. Change in future intent to use cigarettes and ENDS products as measured by responses to the Future Intent to Use Questionnaire Questionnaire responses are measured on a Likert scale range of 1 (extremely unlikely) to 7 (extremely likely) Subjective Effects as measured by the Health Effects Perceptions Questionnaire; time frame: baseline and day 56. Harmful and addictiveness perceptions as measured by responses to the Health Effects Perceptions Questionnaire Puff topography ‐ number of puffs; time frame: baseline, day 28, and day 56. Change in the number of puffs during a 1‐hour puff topography session Puff topography ‐ puff duration; time frame: baseline, day 28, and day 56. Change in puff duration during a 1‐hour puff topography session Puff topography ‐ puff volume; time frame: baseline, day 28, and day 56. Change in puff volume during a 1‐hour puff topography session Puff topography ‐ peak puff flow rate; time frame: baseline, day 28, and day 56; change in peak puff flow rate during a 1‐hour puff topography session Puff topography ‐ average flow rate; time frame: baseline, day 28, and day 56. Change in average flow rate during a 1‐hour puff topography session Puff topography ‐ inter‐puff interval; time frame: baseline, day 28, and day 56. Change in inter‐puff interval during a 1‐hour puff topography session RELX ENDS product use; time frame: day 28 and day 56; change in pod weight during a 1‐hour topography session Incidence of product‐use emergent adverse events (safety and tolerability); time frame: 56 days Incidence of product‐use emergent adverse events
Starting date	Study start date: 15 October 2020. Estimated completion date: April 2021
Contact information	Study Director: Donald Graff. Principal Investigator: Mark Adams, MD Cheerain HK Limited. AMR, USA. Pillar Clinical Research, USA. QPS, USA. No contact details provided.
Notes

**Table:** NCT04709471

Study name	E‐cigarette nicotine study
Methods	Design: RCT. Parallel‐group assignment Setting: USA Start date: 20 January 2021. Estimated completion date: September 2021
Participants	Estimated: 75 Eligibility criteria include at least 21 years old, use e‐cigarettes and tobacco cigarettes regularly, not planning to quit in the near future, and not pregnant, breastfeeding or planning to become pregnant or breastfeed in the next 2 months Additional criteria will be evaluated to assess for eligibility.
Interventions	Experimental: Switch to low‐nicotine e‐cigarettes: switch to e‐cigarettes containing 60% of baseline e‐cigarette nicotine content. Device: Juul e‐cigarette. Participants will switch to Juul pods containing less nicotine. Experimental: Reduce number of e‐cigarette pods: reduce e‐cigarette use to 60% of baseline number of pods per week Behavioural: Reduction: participants will reduce the number of Juul pods that they use No Intervention: Use e‐cigarettes as usual: continue using nicotine e‐cigarettes as usual
Outcomes	Primary outcome measure: Feasibility; time frame: Baseline and the 4‐week reduction period. The investigators will assess compliance with study e‐cigarettes and compare the percentage of non‐study e‐cigarette use between conditions to determine which behaviour‐changing strategy is more feasible. Combustible cigarette smoking; time frame: Baseline and the 4‐week reduction period. The investigators will compare change in number of cigarettes per day between conditions. Cigarette dependence; time frame: Baseline and the 4‐week reduction period. The investigators will compare change in cigarette dependence between conditions using the PATH dependence measure. E‐cigarette dependence: time frame: Baseline and the 4‐week reduction period. The investigators will compare change in e‐cigarette dependence between conditions using the PATH dependence measure. Secondary outcome measure: Cigarette demand; time frame: Baseline and the 4‐week reduction period. The investigators will compare change in cigarette demand using the Brief Assessment of Cigarette Demand task. E‐cigarette demand; time frame: Baseline and the 4‐week reduction period. The investigators will compare change in e‐cigarette demand using a version of the Brief Assessment of Cigarette Demand task adapted for e‐cigarettes.
Starting date	20 January 2021. Study completion date: June 2022.
Contact information	Elias M Klemperer, PhD802‐656‐1641, elias.klemperer@med.uvm.edu
Notes

**Table:** NCT04725656

Study name	Concentration Impact Nicotine Salt (CINS)
Methods	Design: RCT
Participants	Estimated enrolment: 312 Inclusion criteria: Adult (≥ 18 years old) smokers (at least 5 TC per day for at least 12 months) Motivated to quit smoking as evidenced by signing the informed consent form at trial enrolment specifying that a target quit date will be set Saliva cotinine of > 50 ng/mL at screening Willing to participate in the trial even if allocated to the control group Ability to communicate well with the investigator and to understand and comply with the requirements of the study Signed informed consent form Exclusion criteria: Known hypersensitivity/allergy to a content of the e‐liquid Pregnancy or breastfeeding Intention to become pregnant during the course of the study Regular use of EC or tobacco heating systems Use of NRT, varenicline, or bupropion in the month prior to the screening visit Smoke tobacco combined with marijuana and do not currently want to quit marijuana use Participation in an interventional trial within 30 days prior to the screening visit Legal incapacity or limited legal capacity at screening Any circumstances or conditions which, in the opinion of the investigator, may affect full participation in the study or compliance with the protocol
Interventions	Active comparator: Active arm, low concentration (18 mg/mL) nicotine salt e‐liquids. Procedure: Smoking cessation counselling: smoking cessation counselling at baseline, week 1, week 2 and week 4 Other: Open system vape device and nicotine salt e‐liquids; ad libitum use of nicotine salt e‐liquids during 3 months Active comparator: Active arm, high concentration (59 mg/mL) nicotine salt e‐liquids. Procedure: Smoking cessation counselling: smoking cessation counselling at baseline, week 1, week 2 and week 4 Other: Open system vape device and nicotine salt e‐liquids; ad libitum use of nicotine salt e‐liquids during 3 months Control group: Receive only smoking cessation counselling. Procedure: Smoking cessation counselling: smoking cessation counselling at baseline, week 1, week 2 and week 4
Outcomes	Primary outcome: 7‐day point prevalence tobacco abstinence (in terms of non‐inferiority); time frame: 1 month. Defined as no smoking, i.e. "not a puff", self‐reported and confirmed by exhaled carbon monoxide (< 10 ppm) and urinary anabasine levels (< 3 ng/mL) when using low vs. high nicotine salt concentration e‐liquids Volume of e‐liquid used (in terms of superiority); time frame: 1 month; volume of e‐liquid used when using low vs high nicotine salt concentration e‐liquids Secondary outcome: 7‐day point prevalence tobacco abstinence (in terms of non‐inferiority); time frame: 1 month. Defined as no smoking, i.e. "not a puff", self‐reported and confirmed by exhaled carbon monoxide (< 10 ppm) and urinary anabasine levels (< 3 ng/mL) when using low vs. high nicotine salt concentration e‐liquids Volume of e‐liquid used (in terms of superiority); time frame: 1 month; volume of e‐liquid used when using low vs high nicotine salt concentration e‐liquids Liking/rating of trial product (active arms); time frame: 1 and 3 months. Questions regarding helpfulness in refraining from smoking, how satisfying and how good the e‐cigarette tastes compared to the tobacco cigarettes, if they would recommend the assigned trial product to another smoker, and any potential practical problems they might have with the handling Respiratory symptoms; time frame: up to 12 months; checklist with specific questions regarding shortness of breath, wheezing, cough or phlegm Adverse events; time frame: up to 12 months; checklist with specific questions regarding presence or absence of nausea, sleep disturbance, throat/mouth irritation, or other Total nicotine amount vaped; time frame: 1 and 3 months Total volume of e‐liquid consumed; time frame: 1 and 3 months
Starting date	Start date: Jan 2024. Estimated study completion date: 31 Dec 2024
Contact information	Evangelia Liakoni, MD0041316325461, evangelia.liakoni@insel.ch
Notes

**Table:** NCT04946825

Study name	Quit smoking study for people who use e‐cigarettes. A randomized controlled trial of smoking cessation treatment for young adult dual users of combustible and electronic cigarettes
Methods	RCT. Randomized factorial assignment Setting: community; University of Vermont, USA
Participants	Estimated enrolment 390 Inclusion criteria: young adult; smokes tobacco cigarettes; uses EC; interested in quitting tobacco Exclusion criteria: pregnancy or breastfeeding; ≥ 1 contraindications for NRT
Interventions	EC: type not stated NRT: patch and lozenge A) NRT plus text messages to quit CCs only B) NRT plus text messages to quit CCs and ECs simultaneously C) text messages alone to quit CCs only D) text messages alone to quit CCs and ECs simultaneously
Outcomes	Baseline, 3 months, 6 months CO confirmed 7‐day point‐prevalence abstinence at the end of treatment (i.e. 3 months after randomization) CO biochemically confirmed prolonged 30‐day abstinence, 3‐month follow‐up (i.e. end of treatment) and 6‐month follow‐up (3 months after the end of treatment) Self‐reported abstinence, 7 days, 30 days Attempts to quit combustible cigarettes (CC), cpd, CC dependence
Starting date	Study start date: 27 June 2021. Estimated study completion: January 2024.
Contact information	Elias Klemperer, PhD 8026561641, elias.klemperer@med.uvm.edu Shaun Meyers, BA 8026568681, shaun.meyers@uvm.edu
Notes	New to 2022 update

**Table:** NCT05023096

Study name	Potential effects of electronic nicotine delivery system flavor regulations on African American menthol smokers (RVA Flavors)
Methods	RCT Virginia Commonwealth University, USA
Participants	Estimated enrolment: 210 Inclusion criteria: 21+ years; identify as Black/African‐American (single or multi‐race); used ≥ 5 cigarettes per day for ≥ 1 year (biochemically confirmed); regular cigarette brand menthol or mint flavoured; EC use in the past 30 days; no intent to quit smoking in the next 6 months; previous quit attempt using evidence‐based method; mobile phone, willing to receive calls/text Exclusion criteria: unwilling to use EC; report other tobacco use > 10 days in past 30 other than combustible cigarettes; unstable or significant medical condition in the past 12 months; > 15 days of marijuana or other illegal drug use in the past 30 days; pregnancy/breastfeeding
Interventions	EC: type not stated Arm 1: Menthol + tobacco. Both menthol and tobacco‐flavoured liquids for EC are available to choose from. Arm 2: Tobacco ‐ only tobacco‐flavoured liquid is available for EC. Arm 3: Unflavoured ‐ only unflavoured liquid is available for EC. Participants are instructed to smoke their usual brand of menthol cigarettes normally for 7 days and avoid using any other tobacco products. After this baseline week, participants are randomized to 1 of 3 EC flavour conditions; all contain 5% nicotine (menthol + tobacco, tobacco, unflavoured) with equal probability and provided with a supply of their condition‐specific EC and asked to use it in place of their usual menthol cigarettes for the next 6 weeks.
Outcomes	Week 1, week 6 Change in: average daily cigarette use; carbon monoxide exposure; urinary NNAL; urinary propylene glycol exposure; average daily ENDS use Willingness to substitute from cigarettes to EC (ENDS); measure of substitution for condition‐specific tobacco products will be assessed using drug purchase tasks. Choices made during this task are not reinforced. Willingness to pay for ENDS (week 6); willingness to pay for condition‐specific tobacco products will be assessed using drug purchase tasks. Choices made during this task are not reinforced.
Starting date	Study start date: 14 April 2022. Estimated primary completion date: June 2025
Contact information	Andrew J. Barnes, PhD 804‐827‐4361, abarnes3@vcu.edu Caroline O. Cobb, PhD, cobbco@vcu.edu
Notes	New to 2022 update

**Table:** NCT05144542

Study name	Risk and benefits of electronic cigarettes to older smokers at high risk for lung cancer
Methods	RCT Setting: M. D. Anderson Cancer Center, Texas, USA
Participants	Estimated enrolment: 330 Inclusion criteria: meeting National Comprehensive Cancer Network (NCCN) guideline for lung cancer screening; daily or non‐daily smoker; interested in trying ECs to change CC smoking behaviour; willing and able to complete two spirometry sessions Exclusion criteria: used ECs on more than 2 days in the past 30 days; meet criteria for current major depressive disorder (MDD) or suicidality; report more than once weekly of tobacco products other than CCs during the past 30 days; ever diagnosis of lung cancer, have uncontrolled or unstable medical condition; spirometry forced expiratory volume in 1 second (FEV1) percentage reading < 50; pregnancy/breastfeeding
Interventions	EC: type not specified GROUP A: Participants smoke their usual brand of cigarettes for 26 weeks. Participants use smartphone to answer questions about nicotine cravings and mood, and log daily smoking activity every day for up to 182 days. Participants complete questionnaires over 50 minutes and undergo collection of urine sample at 1, 6, 12, and 26 weeks, and collection of blood samples at 6, 12, and 26 weeks. Participants may also undergo measurement of CO levels at 1, 6, 12, and 26 weeks. GROUP B: Participants vape EC for 26 weeks. Participants use smartphone to answer questions about nicotine cravings and mood, and log daily smoking activity every day for up to 182 days. Participants complete questionnaires over 50 mins and undergo collection of urine sample at 1, 6, 12, and 26 weeks, and collection of blood samples at 6, 12, and 26 weeks. Participants may also undergo measurement of CO levels at 1, 6, 12, and 26 weeks.
Outcomes	1, 6, 12, and 26 weeks, and collection of blood samples at 6, 12, and 26 weeks Primary outcome measure: cigarettes per day, diary data of combustible cigarette use over last 24 hours Secondary outcome measures: high‐sensitivity C‐reactive protein (hs‐CRP); white blood cells (WBC); 8‐epi prostaglandin F2 alpha (8‐epi‐PGF2a). All from blood draws at weeks 0, 6, 12, and 26
Starting date	Start date: 7 March 2022. Estimated completion date: 30 April 2025
Contact information	Jason Robinson, PHD 713‐792‐0919, jdrobinson@mdanderson.org
Notes	New to 2022 update

**Table:** NCT05199480

Study name	Understanding the impact of cartridge‐based electronic cigarettes and generated aerosols on cardiopulmonary health
Methods	RCT Virginia Commonwealth University, USA
Participants	Estimated enrolment: 57 Inclusion criteria: E‐cigarette group: ≥ 21 yrs; used EC (≥ 3 times/week for ≥ 3 months) Non‐e‐cigarette group: ≥ 21 yrs Exclusion criteria: use of cigarettes for 15 days or more in the past 60 days; use of other tobacco products (cigars, hookah, smokeless) weekly or more frequently in the past 60 days; use of marijuana or any illicit or prescription drugs for non‐medical use weekly or more frequently in the past 60 days; allergy to propylene glycol or vegetable glycerin; evidence of cardiovascular, pulmonary, renal, hepatic, metabolic, or cerebral diseases; disorder or use of medication that affects cardiopulmonary health; pregnancy/breastfeeding
Interventions	EC: commercially available cartridge‐based EC device Arm 1: E‐cigarette liquid type 1 A commercially available cartridge‐based device with tobacco‐flavoured liquid. Participants will be instructed to use at least one study product daily in place of their own e‐cigarettes during the intervention period. Arm 2: E‐cigarettes liquid type 2 A commercially available cartridge‐based device with tobacco‐flavoured liquid. Participants will be instructed to use at least one study product daily in place of their own e‐cigarettes during the intervention period. Arm 3: No e‐cigarettes. No e‐cigarette use
Outcomes	Baseline, 2 weeks Change in peak oxygen consumption (VO2 peak) Change in expiratory volume Change in skeletal muscle O2 utilization Change in maximal microvascular dilation
Starting date	Study start date: 10 January 2022. Estimated completion date: Dec 2024
Contact information	Paula Rodriguez Miguelez, PhD804‐396‐4498, prodriguezmig@vcu.edu
Notes	New to 2022 update

**Table:** NCT05205811

Study name	A randomized controlled trial to determine the effects of combination zonisamide and bupropion on switching to an electronic cigarette
Methods	RCT Rose Research Center, USA
Participants	Estimated enrolment: 180 Inclusion criteria: 21 to 65 yrs; ≥ 10 commercially available cigarettes per day, for the last 12 months (CO reading ≥ 10 ppm); interested in switching to an EC; smartphone with text message and data capabilities Exclusion criteria: unhealthy or cannot participate in the study for any reason; PHQ‐9 score greater than 9, or a score greater than 0 on item #9; plans to use an FDA‐approved smoking cessation product; high blood pressure, coronary heart disease, structural cardiac disease; BMI ≤ 15.0 kg/m^2^ or > 40.0 kg/m^2^; depression, anxiety, or nicotine withdrawal within 30 days of screening, or during the study, taking antidepressants, psychoactive medications or medications that prolong QTc For full list see NCT record
Interventions	EC: JUUL Zonisamide Bupropion Arm 1: Combination zonisamide and bupropion with EC After the first week of EC use (JUUL), participants will be given bupropion (150 mg each morning for days 1 to 3, then 300 mg daily) with zonisamide (100 mg daily). The combination of zonisamide and bupropion use will continue for 7 weeks of treatment, and EC use will continue until the end of the study (an additional 4 weeks). EC for ad libitum use for 2 weeks prior to complete switch day and for an additional 10 weeks Arm 2: Bupropion with EC After the first week of EC use (JUUL), participants will be given bupropion (150 mg each morning for days 1 to 3, then 300 mg daily) with placebo zonisamide. The combination of placebo and bupropion use will continue for 7 weeks of treatment, and e‐cigarette use will continue until the end of the study (an additional 4 weeks). EC for ad libitum use for 2 weeks prior to complete switch day and for an additional 10 weeks Arm 3: Placebo with EC After the first week of EC use (JUUL), participants will be given placebo bupropion with placebo zonisamide. The combination of these placebos will continue for 7 weeks of treatment, and EC use will continue until the end of the study (an additional 4 weeks). EC for ad libitum use for 2 weeks prior to complete switch day and for an additional 10 weeks
Outcomes	Baseline, week 8, week 12, 6 months Complete switching from combustible cigarettes to JUUL EC as measured by: exhaled carbon monoxide (CO); change in total urinary 4‐(methylnitrosamino)‐1‐(3‐pyridyl)‐1‐butanol (NNAL); change in self‐report of daily cigarette and EC use Seven‐day point abstinence at 6 months post‐switch, assessed by self‐report and confirmed by exhaled CO < 5 ppm. Change in smoking withdrawal symptoms. Change in rewarding and aversive effects of smoking and EC use AEs; SAEs
Starting date	Start date: 14 December 2021. Estimated completion date: 30 June 2024
Contact information	Derek Mercedes 704‐350‐2999, derek.mercedes@roseresearchcenter.com
Notes	New to 2022 update

**Table:** NCT05206435

Study name	Methadone‐maintained smokers switching to e‐cigarettes (SHINE)
Methods	RCT Butler Hospital, Providence, Rhode Island, United States, 02906
Participants	Estimated enrolment: 240 Inclusion criteria: Moderate to heavy cigarette smokers (10 cigarettes/day for > 1 yr; breath CO > 10 ppm) Receiving methadone for ≥ 3 months and attend at least weekly to receive methadone dose Interested in switching to EC or NRT Exclusion criteria: Using ECs on > 2 of the past 30 days Currently use medications that may reduce smoking (e.g. bupropion, varenicline, NRT) Unstable psychiatric conditions Near‐daily or daily use of marijuana Pregnancy Cardiovascular event in the last month, daily medication for asthma or COPD
Interventions	EC: type not stated Arm 1: Electronic cigarettes. Participants in this arm are randomized to receive electronic cigarettes for the 6‐week study period. Electronic cigarettes are provided to replace tobacco cigarettes. Arm 2: Nicotine lozenges. Participants in this arm are randomized to receive nicotine lozenges for the 6‐week study period. Nicotine lozenges are provided to replace tobacco cigarettes.
Outcomes	Baseline, 6 weeks Nicotine exposure (urine) Lung functioning: FVC (changes in Forced Vital Capacity, spirometry); FEV1 (changes in Forced Expiratory Volume (FEV ‐ during the first second), spirometry) Smoking behaviour and experiences (self‐report) For complete switchers: nicotine exposure; lung functioning (FVC, FEV1, FEV1/FVC); smoking behaviour and experiences
Starting date	Study start date: 31 March 2022. Estimated study completion date: 30 June 2024.
Contact information	Michael Stein, MD 401‐455‐6200, michael_stein@brown.edu Ana Abrantes, PhD 401‐455‐6200, ana_abrantes@brown.edu
Notes	New to 2022 update

**Table:** NCT05257629

Study name	Aggressive smoking cessation therapy post‐acute coronary syndrome (ASAP) trial
Methods	RCT Setting: hospital Jewish General Hospital, USA
Participants	Estimated enrolment: 798 Inclusion criteria: Currently hospitalized (or at time of discharge) for ACS. Defined as follows: MI, defined by positive troponin T, troponin I, or CK‐MB levels (as defined by institution‐specific cut‐offs). For definition, see NCT record. CC user; motivated to quit smoking according to the Motivation To Stop Scale (MTSS) (≥ level 5); ≥ 18 years Exclusion criteria: Use of any of the following in the 30 days prior to ACS admission: i. Pharmacotherapy (e.g. NRTs, bupropion, or varenicline) for smoking cessation; ii. Nicotine or non‐nicotine e‐cigarettes; iii. Psychotropic medications (e.g. mood stabilizers, antipsychotics, prescribed opiates and sedatives); iv. Other anti‐craving medication (e.g. naltrexone, acamprosate) with the potential to alter substance‐seeking behaviours Pregnancy/breastfeeding For a full list, see NCT record.
Interventions	EC: participant's choice Arm 1: Combination therapy arm (varenicline and nicotine EC plus counselling) Patients in the combination therapy arm will be supplied funds and instructions for the purchase of EC and cartridges/pods upon hospital discharge and at the week 4 and 12 clinic visits. As with standard NRTs such as the gum, inhaler, and lozenge, we expect smokers will self‐regulate administration according to their withdrawal symptoms. Use will be monitored via self‐report for telephone follow‐ups. At clinic visits, patients will be asked to bring their EC, used and unused cartridges/pods, and purchasing receipts. Patients will be advised regarding the signs and symptoms of nicotine toxicity and of an allergic reaction. Arm 2: Varenicline plus counselling All patients will begin varenicline in‐hospital upon randomization. For the first 3 days, patients will take a 0.5 mg tablet once a day. They will then take a 0.5 mg tablet twice a day for the following 4 days, and one 1 mg tablet twice a day from day 8 onward for the remainder of the 12‐week treatment. Use will be monitored via self‐report for telephone follow‐ups and return of all unused tablets at the end of the treatment period. Should a patient experience severe side effects (such as headache, nausea, vomiting, dizziness, dyspepsia, fatigue, insomnia, abnormal dreams, constipation, or flatulence) on day 8 onward, the varenicline dose should be reduced from 1 mg twice daily to 0.5 mg twice daily prior to study medication discontinuation.
Outcomes	1, 2, 8, 18, 24 weeks Week 4, week 12, and week 52 Number of participants with: 7‐day point prevalence smoking abstinence (biochemically‐validated); continuous smoking abstinence; prolonged smoking abstinence; change in daily cigarette consumption; ≥ 50% reduction in daily cigarette consumption; point prevalent abstinence or ≥ 50% reduction in daily cigarette consumption at 24 weeks Frequency of adverse events (AEs) or SAEs Spirometry measurements (subset) at all other clinic visits (FVC, FEV1, and FEV1/FVC) O2 cost diagram and COPD Assessment Test (subset) at all other clinic visits Number of patients averaging ≥ 1 pill of varenicline/day
Starting date	Estimated start date: 1 June 2022. Estimated completion date: 7 March 2027
Contact information	Carole Bohbot 514‐340‐8222 ext 22790 ASAP.Trial@ladydavis.ca, carole.bohbot@ladydavis.ca
Notes	New to 2022 update

**Table:** NCT05278065

Study name	Complimentary electronic cigarettes for harm reduction among adult smokers with asthma (SWAP)
Methods	RCT Setting and recruitment: Participants will be adults from the local community with persistent asthma symptoms who are regular combustible cigarette smokers and do not also regularly use ENDS. The study will recruit 30 non‐treatment‐seeking participants using flyers, advertisements, a website triaging visitors to the Center for Alcohol and Addiction Studies, and through targeted recruitment at community immunology clinic partners at Rhode Island Hospital, USA.
Participants	Estimated enrolment 30 Inclusion criteria: 21 to 65 years Persistent asthma symptoms (i.e. episodic symptoms of airflow obstruction/airway hyper‐responsiveness (AHR) as documented in review of medical history) Currently prescribed SABA medication Past‐year smoking of ≥ 5 cigarettes/day (CO ≥ 6 ppm at baseline) Zero breath alcohol during informed consent for participation Exclusion criteria: Intention to quit smoking during the next 30 days or current engagement in any smoking cessation treatment Regular EC/ENDS user or using ENDS > 2 days/week Medical contraindication to nicotine Pregnancy (due to toxicity of nicotine and tobacco products) Current alcohol dependence (AUDIT > 15) Urine‐screened or past‐month self‐reported use of illicit substances (amphetamine, cocaine, methamphetamine, opioids, benzodiazepines) Current psychosis, mania, or suicidal ideation
Interventions	EC: 4th generation and disposable cartridges Arm 1: Electronic cigarette Participants in this experimental condition will be provided with a 4th generation EC device and disposable cartridges. Participants will be provided with EC and 5% nicotine e‐liquid cartridges for 8 weeks and encouraged at weekly assessments to use the EC any time they would normally smoke. Participants will be able to choose commercially available e‐liquid flavours (tobacco) at each weekly assessment. Arm 2: Smoking‐as‐usual Participants in this assessment‐only condition will continue smoking‐as‐usual.
Outcomes	Baseline, week 8, week 16. Eight weekly visits to complete follow‐up assessments cpd EC use Asthma symptoms Pulmonary functioning, FEV, FVC, FEF25‐75, PEF CO. Level of exhaled CO assessed with Smokerlyzer NNAL Cotinine Interleukin‐6 (IL‐6) Tumour necrosis factor alpha (TNF‐a) Chemokine ligand 9 (CXCL9) Matrix metallopeptidase 9 (MMP9)
Starting date	Start date: 1 May 2022. Estimated study completion date: May 2024
Contact information	Alexander W Sokolovsky, PhD 4018636629, alexander_sokolovsky@brown.edu Mary Ellen Fernandez, BA 4018635521, mary_fernandez@brown.edu
Notes	New to 2022 update

**Table:** NCT05311085

Study name	Cytisine and e‐cigarettes with supportive text‐messaging for smoking cessation (Cess@Tion)
Methods	RCT Setting: community University of Auckland, New Zealand
Participants	Estimated enrolment: 800 Inclusion criteria: daily smokers who live in New Zealand; motivated to quit smoking within the next 2 weeks and willing to use cytisine or an EC or both products; ≥ 18 years Exclusion criteria: another person in their household currently enrolled in the study; pregnancy/breastfeeding; using smoking cessation medication (including EC daily for the last month); hypersensitivity to cytisine or nicotine EC; health condition e.g. renal impairment; tuberculosis; myocardial infarction, stroke, or severe angina, high BP, seizures; strong preference to use or not to use cytisine and/or EC in their quit attempt For a full list, see NCT record.
Interventions	EC: pod device. Nicotine strength: 30 mg/mL (3%). Flavour: tobacco. Brand name: UpOx Cytisine Arm 1: Monotherapy (cytisine only) 12 weeks of cytisine: Participants allocated cytisine will be instructed to follow the manufacturer's 25‐day dosing regimen, then follow a maintenance dose of cytisine from day 26 to week 12. Participants will also receive 6 months of text‐based smoking cessation support. Cytisine. Brand name: Tabex. Standard dosing of: Days 1 to 3: 1 tablet (1.5 mg) every 2 hours through the waking day (6 tablets/day) Days 4 to 12: 1 tablet every 2.5 hours (5 tablets/day). Quit smoking date is day 5 Days 13 to 16: 1 tablet every three hours (4 tablets/day) Days 17 to 20: 1 tablet every 4 to 5 hours (3 tablets/day) Days 21 to 25: 1 tablet every six hours (2 tablets/day) Followed by a maintenance dose of cystine from day 26 to week 12 (1 tablet every 6 hours: 2 tablets/day) Arm 2: Monotherapy (nicotine EC only) 12 weeks of a nicotine EC. Participants will also receive 6 months of text‐based smoking cessation support. Arm 3: Combination therapy (cytisine plus a nicotine EC) 12 weeks of cytisine (as above) and 12 weeks of a nicotine EC. Participants will also receive 6 months of text‐based smoking cessation support.
Outcomes	Baseline, 3, 6, and 12 months post‐quit date Primary outcome: proportion of participants with verified continuous smoking abstinence CO confirmed Self‐reported continuous smoking abstinence; self‐reported 7‐day point prevalence smoking abstinence; change from baseline in the number of cigarettes smoked per day; health‐related quality of life; cystine compliance; use of allocated treatment by participants; frequency of EC use, number of pods used; treatment switching; dual use; AEs; number of text‐based behavioural support messages received by participants; marginal cost per quitter
Starting date	Study start date: 6 May 2022. Estimated primary completion date: February 2024
Contact information	Natalie Walker, PhD 64‐9‐923‐9884, n.walker@auckland.ac.nz Chris Bullen, PhD MBChB 64‐9‐923‐4730, c.bullen@auckland.ac.nz
Notes	New to 2022 update

**Table:** NCT05327439

Study name	Using alternative nicotine delivery systems (ANDS) to reduce harm for low SES cigarette smokers. (Tri‐PEC study)
Methods	RCT Center for Alcohol and Addiction Studies, Brown University School of Public Health, USA
Participants	Estimated enrolment: 45 participants Inclusion criteria: household income < 250% federal poverty level (FPL); past 6 months daily smoking of ≥ 5 cigarettes/day (exhaled CO ≥ 6 ppm at baseline); willingness to substitute combustible cigarettes for EC or NPs; aged 21+ years Exclusion criteria: intention to quit smoking during the next 30 days or current or past 30‐day engagement in smoking cessation; current use of EC or NP ≥ 4 days per month or self‐report of primarily using tobacco products that are not combustible cigarettes; hospitalization for a psychiatric issue in the past 30 days or visible instability; heart‐related event in the past 30 days; pregnancy Note: Cannabis use will be assessed but not excluded. For full list, see NCT record.
Interventions	EC: 4th generation electronic cigarette device and disposable cartridges (5% nicotine e‐liquid cartridges) Nicotine pouch, 4 mg Arm 1: Electronic cigarette Participants in this experimental condition will be provided with a 4th generation EC device and disposable cartridges. Participants will be provided with EC and 5% nicotine e‐liquid cartridges for 8 weeks and encouraged at in‐person and phone assessments to use the EC any time they would normally smoke. Participants will be able to choose one of two e‐liquid flavours (tobacco, menthol) at baseline. Arm 2: Nicotine pouch Participants in this experimental condition will be provided with nicotine pouches. Participants will be provided with 4 mg nicotine pouches for 8 weeks and encouraged at in‐person and phone assessments to use the nicotine pouches any time they would normally smoke. Participants will be able to choose one of two nicotine pouch flavours (tobacco, mint) at baseline. Arm 3: No intervention: smoking‐as‐usual Participants in this assessment‐only condition will continue smoking‐as‐usual.
Outcomes	Baseline, 8 weeks Change in cigarettes per day from baseline to week 8. Within and between‐group difference in past week average cigarettes per day assessed using timeline follow‐back (TLFB) Change in cigarette dependence from baseline to week 8 Cigarette abstinence at week 8. Past week any‐use of cigarettes assessed using timeline follow‐back (TLFB) Change in carbon monoxide; cotinine; NNAL; 8‐isoprostane from baseline to week 8 Feasibility and acceptability
Starting date	Start date: 23 March 2022. Estimated completion date: 30 December 2022
Contact information	Jasjit S Ahluwalia, MD 401‐863‐6654, jasjit_ahluwalia@brown.edu, Brown University, USA
Notes	New to 2022 update

**Table:** NCT05510154

Study name	Impact of e‐cigarette training on puff patterns, cigarette smoking, and health outcomes among smokers with COPD; COPD e‐cigarette topography training
Methods	Design: RCT. An open‐label, randomized clinical trial of e‐cigarette training and training dose amongst smokers with COPD Smokers with COPD (n = 45) stratified by e‐cigarette use history (naïve vs current use) will be randomized (1:1:1) to receive 1) brief advice to switch to e‐cigarettes, 2) single‐session e‐cigarette training, or 3) enhanced e‐cigarette training. Setting: University of Kansas Medical Center (KUMC) campus in Kansas City, Kansas (KS), USA Study start date March 2022. Study end June 2023.
Participants	N = 45 Smokers with COPD (n = 45) stratified by e‐cigarette use history (naïve vs current use) will be randomized (1:1:1) to receive 1) brief advice to switch to e‐cigarettes, 2) single‐session e‐cigarette training, or 3) enhanced e‐cigarette training. Inclusion criteria: smokers or dual users diagnosed with COPD; ≥ 21 years old; speak and understand English; smoke on > 25 of the last 30 days for the past 3 months; willing to switch from cigarettes to the study e‐cigarette for the duration of the study; have tried but failed to quit smoking in the last year; unwilling to make a pharmacotherapy‐assisted quit attempt in the next 30 days Exclusion criteria: smokers or dual users; use of tobacco products other than cigarettes, including e‐cigarettes in the past 30 days; current use of cessation medications; pregnant, planning to become pregnant, or breastfeeding; recent history of cardiovascular or pulmonary events in the past 3 months; household member current or previously enrolled in the study
Interventions	EC. The study product is an e‐cigarette device and is available for sale in the US. Brief advice to switch to e‐cigarettes Single‐session e‐cigarette training Enhanced e‐cigarette training (3 real‐time training sessions rather than 1)
Outcomes	12 weeks Changes in puff duration in seconds from pre‐ to post‐e‐cigarette training (time frame: 12 weeks) Complete switch to e‐cigarette (time frame: 12 weeks) Change in spirometry FVC, FEV1, FEV1/FVC ratio, systolic blood pressure, change in diastolic blood pressure, change in COPD Assessment Test (CAT) score, respiratory symptoms score, change in 6‐minute walk test distance
Starting date	Study start date March 2022
Contact information	Eleanor Leavens, Assistant Professor, University of Kansas Medical Center
Notes	New to 2023 update

**Table:** NCT05525078

Study name	E‐cigarettes as harm reduction tools in smokers who fail to quit with traditional methods; quit or switch: e‐cigarette study
Methods	Design: Randomized parallel assignment
Participants	N = 30 EC = 20 NRT = 10 Inclusion criteria: at least 21 years old; smoking at least 5 cigarettes per day for 1 year; a quit attempt using an FDA‐approved pharmacotherapy in the past year that resulted in abstinence of at least 24 hours; intention to quit smoking within the next month Exclusion criteria: use of non‐cigarette tobacco products in the past 30 days; current use of pharmacotherapy for smoking cessation; pregnant, breastfeeding, or trying to become pregnant
Interventions	EC vs nicotine patch/nicotine lozenge Provided 4 weeks of study product Behavioural: e‐cigarette. Participants will select a Target Switch on which they will stop smoking cigarettes and switch entirely to the e‐cigarette product. Other: nicotine patch, nicotine lozenge. Participants will select a Target Quit Date on which they will stop smoking, using the patches and lozenges provided.
Outcomes	Baseline to day 35 (5 weeks) Percentage of participants who report daily use of e‐cigarettes or NRT and no cigarette use (time frame: day 0 through day 35), daily use of e‐cigarette or NRT instead of cigarettes Percentage of participants who have biochemically confirmed abstinence from cigarette smoking (time frame: day 0 through day 35) CO measured for all participants. Self‐reported respiratory symptoms
Starting date	Study start date August 2022. Estimated study end date September 2023.
Contact information	Tracy Smith, Medical University of South Carolina, 843‐792‐5164, smithtra@musc.edu J'Neice Payne, 843‐876‐2291 paynejne@musc.edu
Notes	New to 2023 update

**Table:** NCT05555069

Study name	The impact of menthol flavoring on switching in adult menthol smokers
Methods	Design: Randomized parallel assignment Setting. USA; University of Kansas Medical Center
Participants	Estimated enrolment: 800 participants Inclusion criteria: ≥ 21 years of age; smoke ≥ 5 cigarettes per day (CPD); smoke menthol cigarettes for ≥ 6 months; verified smoker (CO > 5ppm); functioning telephone; interested in switching to e‐cigarettes Exclusion criteria: interested in quitting smoking; use of other tobacco products in past 30 days (i.e. cigarillos, cigars, hookah, smokeless tobacco, pipes); e‐cigarette use on ≥ 4 of the past 30 days; uncontrolled hypertension: BP ≥ 180 (systolic) or ≥ 105 (diastolic); use of smoking cessation pharmacotherapy in the month prior to enrolment; pregnant, contemplating getting pregnant, or breastfeeding Motivated to quit: no
Interventions	EC: 4th generation nicotine salt‐based pod‐system e‐cigarette in menthol versus tobacco‐flavoured e‐liquid Arm 1. Menthol flavour electronic cigarette. 400 adult cigarette smokers will receive 12 weeks of menthol‐flavoured electronic cigarettes. Arm 2. Tobacco flavour electronic cigarette. 400 adult cigarette smokers will receive 12 weeks of tobacco‐flavoured electronic cigarettes. Participants will receive 12 weeks of menthol OR tobacco‐flavoured electronic cigarettes to aid in switching from combustible cigarettes. Participants will be instructed on proper use of electronic cigarettes, educated about electronic cigarettes and participate in motivation enhancement and substituting electronic cigarettes for cigarettes.
Outcomes	Baseline, 12 weeks. Follow‐up will continue to 26 weeks. Outcomes at 12 weeks 1. Number of participants who switch from cigarettes to electronic cigarettes at week 12 Complete switching is defined as exclusive use of e‐cigarettes, confirmed with CO < 6 ppm and predominant switching; defined as use of the e‐cigarette with > 50% reduction in CPD. This will compare the effectiveness of menthol versus tobacco e‐cigarettes in facilitating switching at week 12. 2. Assessment of respiratory symptoms using spirometry Spirometry summarizing forced expiratory flow (FEF) 25‐75% and the American Thoracic Society Questionnaire will assess acute respiratory symptoms experienced by cigarette and electronic cigarette smokers. This will help assess the tobacco harm reduction of electronic cigarettes. 3. Amount of e‐liquid consumed
Starting date	8 November 2022. Estimated primary completion date 30 June 2025
Contact information	Tricia Snow, 816‐398‐8960 psnow@kumc.edu PI Nicole Nollen, PhD, University of Kanas Medical Center
Notes	New to 2023 update

**Table:** NCT05610514

Study name	Pulmonary and cardiac effects of e‐cigarette use in pulmonary patients who smoke cigarettes
Methods	Design: Randomized, cross‐over, open‐label Setting: Greater Burlington, VT, USA
Participants	N = 25 (estimated enrolment) Inclusion criteria: men and women 40 years of age or older; current, every‐day smoker (5 or more cigarettes per day for one year or longer) confirmed with intake CO of 8 ppm or greater; established pulmonary disease (chronic obstructive pulmonary disease (COPD), chronic bronchitis, emphysema, or asthma‐COPD overlap syndrome) confirmed by physician diagnosis and/or current prescription of medication for treatment (i.e. LABA, LAMA, ± ICS, or combination); no intention to quit smoking within the next month Exclusion criteria: patients who are medically unstable (unstable symptoms, changes in medications or hospitalizations within last 3 months); inability to conduct in‐home measurements Motivated to quit: no
Interventions	EC: JUUL/Vuse Alto and pods Experimental: e‐cigarette. Participants in this arm will smoke electronic cigarettes for 2 weeks. E‐cigarettes (either JUUL or Vuse Alto) and pods (JUUL: Virginia tobacco flavour at 3% or 5% nicotine concentration; Vuse Alto: golden tobacco flavour at 1.8%, 2.4%, or 5% nicotine concentration) will be provided. In the EC arm, availability of e‐cigarettes and altering the availability of financial incentives for abstaining from combustible cigarettes will be investigated. No intervention: combustible cigarette. Participants in this arm will smoke their usual brand of combustible cigarettes for 2 weeks.
Outcomes	Baseline, 2 weeks, 4 weeks Baseline and change from baseline: FEV1/FVC; lung reactance; oxygen saturation (SpO2); exhaled nitric oxide (FeNO); COPD; blood pressure; heart rate; tobacco use; Fagerstrom Test of Nicotine Dependence (FTND); Wisconsin Inventory of Smoking Dependence Motives‐Brief (WISDM‐Brief); Minnesota Tobacco Withdrawal Scale (MNWS); Questionnaire on Smoking Urges‐Brief (QSU‐Brief); health changes
Starting date	28 April 2022; estimated completion date April 2023
Contact information	Brian R Katz, PhD, 8025511798, Brian.Katz@uvm.edu Shannon O'connor, 8025511798, shannon.oconnor@uvm.edu
Notes	New to 2023 update

**Table:** NCT05703672

Study name	4th generation e‐cigarettes in African American smokers: reducing harm and quitting combustible cigarettes in dual users Brief title: Switching to e‐cigarettes in African‐American smokers
Methods	Design: Randomized, parallel‐assessment interventional study Setting: Missouri, USA. Swope Health Central, Kansas City, Missouri, United States, 64130. University of Kansas Medical Center, Kansas City, Missouri, United States, 64130
Participants	Estimated N = 500 Inclusion criteria: African‐American; ≥ 21 years of age; smoke > 5 cigarettes per day; smoked cigarettes for > 6 months; verified smoker (CO > 5 ppm); interested in switching to EC Exclusion criteria: interested in quitting smoking; use of smoking cessation pharmacotherapy in the month prior to enrolment; use of other tobacco products in past 30 days (i.e. cigarillos, cigars, hookah, smokeless tobacco, pipes); EC use on > 4 of the past 30 days; uncontrolled hypertension: BP > 180 (systolic) or > 105 (diastolic); heart‐related event in the past 30 days; medical contraindications to VAR: unstable cardiac condition (e.g. unstable angina or AMI) cardiac event, or stroke in the past 4 weeks; renal impairment; history of clinically significant allergic reactions; history of epilepsy or seizure disorder; hospitalized for psychiatric issue in past 30 days; active suicidal ideation; pregnant, contemplating getting pregnant, or breastfeeding Motivated to quit: no
Interventions	EC: Nicotine salt pod‐based e‐cigarette in 5% nicotine Arm 1: Experimental: varenicline and electronic cigarette At the end of the 6‐week open‐label phase, dual users of cigarettes and e‐cigarettes will receive 1 mg varenicline to take twice daily for 12 weeks. They will also receive an additional 12 weeks of the nicotine salt‐based pod system e‐cigarette. Drug: Varenicline Tartrate, 0.5 mg once daily for days 1 to 3, 0.5 mg twice daily for days 4 to 7 and 1.0 mg twice daily from day 8 through week 12. Electronic cigarette: nicotine salt pod‐based e‐cigarette in 5% nicotine Arm 2: Placebo comparator: placebo and electronic cigarette At the end of the 6‐week open‐label phase, dual users of cigarettes and e‐cigarettes will receive placebo pills to take twice daily for 12 weeks. They will also receive an additional 12 weeks of the nicotine salt‐based pod system e‐cigarette. Drug: placebo 1 pill (white) once daily for days 1 to 3, one pill (white) twice daily for days 4 to 7 and 1 pill (blue) twice daily from day 8 through week 12. Electronic cigarette: nicotine salt pod‐based e‐cigarette in 5% nicotine Arm 3: Open‐label electronic cigarette All participants will receive an initial 6‐week supply of the study electronic cigarette. Nicotine salt pod‐based e‐cigarette in 5% nicotine
Outcomes	Baseline, 6 weeks, 12 weeks. FU to 52 weeks Reduction in toxicant exposure as measured by NNAL excretion from baseline to week 6 CO verified 7‐day point prevalence abstinence from cigarettes at week 12 post‐randomization
Starting date	Estimated study start date 30 June 2023. Estimated completion date 30 November 2024.
Contact information	Tricia Snow, MPH, 816‐398‐8960, psnow@kumc.edu
Notes	New to 2023 update

**Table:** NCT05815199

Study name	Effectiveness and impact of counseling enhanced using electronic cigarettes for harm reduction in people with serious mental illness Brief title: E‐cigarettes for harm reduction among smokers with serious mental illness
Methods	Design: randomized, parallel‐assignment. RCT Setting: NYU Langone Health, USA
Participants	Estimated enrolment: 60 Inclusion criteria: currently smokes 5 or more CPD; age of at least 21 years; has SMI diagnosis (such as schizophrenia, schizoaffective disorders, bipolar disorder, depressive disorders, trauma and stressor‐related disorders etc.) as determined using the MINI tool; interested in reducing CC smoking but not necessarily trying to quit Exclusion criteria: pregnant /breastfeeding; used tobacco other than CC in the past 2 weeks (e.g. EC, cigarillo); currently engaged in an attempt to quit CC; change in dose of their psychotropic medication(s) in the last 30 days; meeting DSM‐V criteria for current alcohol or substance use disorder except for nicotine use disorder and active mild alcohol or substance use disorders; past month suicidal ideation/suicide attempt and/or psychiatric hospitalization in the last 30 days Population: people with serious mental illness (SMI) Motivated to quit: interested in reducing but not interested in quitting
Interventions	EC: NJOY Ace Electronic Cigarette Intervention period: 8 weeks Arm 1. Experimental: e‐cigarettes (EC) Interventions: E‐cigarette (EC) NJOY Ace; behavioural: harm‐reduction counselling; behavioural: Ecological Momentary Intervention (EMI) text messaging Arm 2. Active comparator: nicotine replacement therapy (NRT) Interventions: Other: nicotine replacement therapy (NRT) (patches, lozenges and gum); behavioural: harm‐reduction counselling; Behavioural: Ecological Momentary Intervention (EMI) text messaging Description of behavioural intervention for both groups Behavioural: harm‐reduction counselling. At baseline, after randomization, participants will receive their first telehealth session (20 to 25 minutes) from a counsellor trained in motivational interviewing, harm reduction, and smoking cessation. Up to 5 additional sessions will be delivered, 15 to 20 minutes each. Behavioural: Ecological Momentary Intervention (EMI) text messaging. EMI can be defined as delivering tailored interventions via electronic messages (i.e. regular text messages) that include personalized feedback based on real‐time assessment responses and other contextual factors. EMI will take place throughout the intervention period.
Outcomes	Baseline, wk 4, wk 8, wk 12 Abstinence from CC (wk 4, wk 8, wk 12). Self‐report (daily diary about smoking behaviour) and verified by exhaled carbon‐monoxide (eCO) level (< 6 ppm) Self‐reported percent change in CPD (baseline to wk 8, baseline to wk 12) Change in American Thoracic Questionnaire Score from baseline to wk 12. 8‐item questionnaire assessing general thoracic pain Change in Symptom Check Questionnaire Score from baseline to wk 12. 9‐item assessment of chronic obstructive pulmonary disease (COPD) symptoms
Starting date	Estimated start date: July 2023. Estimated completion date: March 2024.
Contact information	Omar El‐Shahawy, 646‐501‐3587, Omar.ElShahawy@nyulangone.org Adetayo Fawole, 646‐501‐3568, Adetayo.fawole@nyulangone.org
Notes	New to 2023 update

**Table:** NCT05825924

Study name	Randomized, two arm parallel, clinical trial to compare effectiveness of different tobacco harm reduction products in general adult population in low middle income countries
Methods	Design: randomized, parallel‐assignment, 2‐arm trial Setting: low‐middle‐income countries
Participants	Estimated enrolment: 258 Inclusion criteria: at least of legal age allowed for smoking in the country, of either gender, regular smokers (minimum 10 cigarettes/day for at least a year) and interested in stopping smoking Exclusion criteria: pregnant/breastfeeding; using other smoking cessation medications (including other forms of NRT other than patch, bupropion, clonidine, nortriptyline or varenicline); any contraindications to products such as cardiovascular history; major illness with prognosis of less than 1 year Motivated to quit: yes
Interventions	EC: EC 18 mg/mL designed to resemble tobacco cigarettes, aerosol generator, sensor, battery and storage area for liquid. Disposable or rechargeable Study Arm 1: 18 mg nicotine EC (ad libitum use) for 12 weeks after the nominated quit date Free EC and sufficient nicotine cartridges (18 mg/mL) supply to last till next in‐person visit. Participants will be instructed to use the device ad libitum 1 week before their quit day to familiarize themselves with its operation and on their designated quit day will stop smoking tobacco cigarettes and instead use the EC exclusively for the next 12 weeks. CC users often take 10 to 15 puffs over the course of 5 to 8 minutes, repeating this pattern with each cigarette. EC users may periodically use it throughout the day, and they may or may not take their puffs like those of traditional CCs. Study Arm 2: 21 mg nicotine patches (one daily) for 12 weeks after the nominated quit date 21 mg nicotine patches supply to last until the next‐person visit. Participants will use the nicotine patch daily for 1 week before their quit day to familiarize themselves with its use. On their designated quit day, they will stop smoking and use nicotine patches daily for the next 12 weeks. Usually, a full‐strength patch (15 to 22 mg of nicotine) daily for 4 weeks is suggested for use in the majority of smokers, followed by a lower‐strength patch (5 to 14 mg of nicotine) for an additional 4 weeks, depending on their body size and smoking habits. The nicotine patches are applied on the skin and nicotine is delivered at a steady rate. After administration, the peak blood levels are achieved within 6 to 10 hours. The levels remain constant, reducing by 25% to 40% with use of patches once daily. The patch is typically administered every 24 hours for no longer than 12 weeks. The dose of the patches is often determined by daily cigarette consumption and level of addiction. The duration of counselling will be at least 30 minutes on site. The duration of counselling through telephone will be at least 10 minutes. Participants will be scheduled for a screening visit and a baseline (BL) visit at the trial site. The participants will be scheduled for 8 study visits in total, including 5 treatment sessions and 3 follow‐up visits, using both face‐to‐face interaction at the trial site as well as follow‐up on telephone.
Outcomes	Weeks 1, 2, 4, 8, 12, 18, 24, and 52 7 day PP. Self‐report having smoked no cigarettes in the past 7 days Number of cigarettes smoked per day assessed using self‐reported diaries AEs (time frame 12 weeks). AEs evaluated using Naranjo Adverse Drug Reaction Probability Scale Physical signs and symptoms of withdrawal using Fagerstrom test for nicotine dependence Perception of the product
Starting date	Estimated study start date: September 2023. Estimated primary completion date: March 2025
Contact information	Ather Mehmood, FCPS +92518314299, athermehmood70@gmail.com
Notes	New to 2023 update Funded by: Foundation for a Smoke Free World INC

**Table:** NCT05881304

Study name	Switching individuals in treatment for opioid use disorder who smoke cigarettes to the SREC
Methods	Design: waiting‐list controlled RCT Setting: Massachusetts General Hospital, USA
Participants	Estimated enrolment: 40 Inclusion criteria: 18 +; report daily cigarette smoking (≥ 10 cigarettes per day in the past week); not ready to quit smoking (not planning to quit in the next 30 days); willing to try EC; in stable buprenorphine (BUP) treatment for opioid use disorder at a Massachusetts General Hospital‐affiliated primary care clinic (in treatment for ≥ 3 months without changes in BUP dose in the past 2 wks and planning to remain on current BUP treatment for ≥ 3 months) Exclusion criteria: pregnant/breastfeeding; using non‐cigarette nicotine or tobacco products (e.g. EC, cigarillos) recently (> 3 days in past 30 days); report past 30‐day use of behavioural or pharmacologic smoking cessation aids; have an unstable psychiatric or medical condition Motivated to quit: no EC use at baseline: no
Interventions	EC: NIDA standardized research e‐cigarette (SREC) Arm 1. Experimental: Immediate standardized research EC (SREC) provision (iSREC) Those randomized to the iSREC group will be provided a free 8‐week supply of standardized research e‐cigarettes (SRECs) and asked to try to switch completely to the SREC. Arm 2. Active Comparator: Delayed SREC provision waiting‐list control (WLC) Those in the WLC condition will receive SREC provision after an 8‐week delay.
Outcomes	Baseline, 2 wks, 8 wks SREC for 8 weeks, either immediately (iSREC), or after an 8‐week delay (waiting‐list control [WLC]). They will be followed for an additional 4 weeks after SREC provision ends (to 12 weeks in iSREC and 20 weeks in WLC). 1) tobacco use behaviour (CPD, SREC use), 2) biomarkers (e.g. carbon monoxide, anabasine), 3) cigarette dependence and withdrawal, and 4) short‐term health effects and tolerability (e.g. respiratory symptoms, substance use) Change in cigarettes smoked per day (CPD) between randomized groups. Change in mean number of CPD in the past 7 days from baseline 2 to week 8 comparing between randomized groups (iSREC group vs WLC) EC use during EC provision during the 8 wks of EC provision Change in expired air carbon monoxide (CO) during EC provision. Change in expired air CO (ppm) from baseline to week 8 between randomized groups Change in anabasine ‐ during EC provision. Change in urine anabasine level (ng/mL)from baseline 1 to week 8 between randomized groups
Starting date	Estimated study start date: August 2023. Estimated completion date: December 2024
Contact information	Joanna M Streck, PhD, 617‐643‐9977, jstreck@mgh.harvard.edu
Notes	New to 2023 update

**Table:** NCT05887947

Study name	Impact of e‐cigarette nicotine concentration on compensation, cigarette smoking, and biomarkers of exposure and harm in diverse smokers
Methods	Design: randomized cross‐over Setting: University of Kansas Medical Center, USA
Participants	Estimated enrolment: 48 Inclusion criteria: identify as non‐Hispanic white or non‐Hispanic African‐American/black; willing to switch from CC to EC for 6 wks; smoke greater than or equal to 25 of the last 30 days for the past 3 months; not previously used an EC for > 30 days; exhaled CO of ≥ 6ppm at screener visit; willing to abstain from marijuana for 12 hours prior to in‐person lab visits; willing to abstain from smoking and vaping for 12 hours prior to 3 in‐person lab visits Exclusion criteria: weekly use of EC over the last 6 months; use of tobacco products other than CC on ≥ 10 days in the past 30 days; use of EC on > 5 of the past 30 days; current use of cessation medications; pregnant/breastfeeding; past 30‐day hospitalization/ER visit for psychiatric issue, seizure, stroke, or new heart problem; recent history of cardiovascular or pulmonary events in the past 3 months; treatment for alcohol or drug dependence in the past yr; current enrolment in a programme aimed at changing smoking patterns Motivated to quit: not clear but excluded if using current cessation medication EC use at baseline: no
Interventions	EC: Pod. Electronic cigarette nicotine concentrations 1.8% and 5% Participants will complete 2 standardized, 10‐puff vaping bouts over 5 mins followed by a 60‐minute ad libitum vaping session, using 2 e‐liquids that differ only by nicotine concentration (5% vs 1.8%) to examine the effect of nicotine concentration on in‐lab compensatory puffing, nicotine exposure, and e‐liquid consumption. In Phase 2, the same participants will be randomized to 5% or 1.8% nicotine e‐liquid and instructed to switch completely for 6 weeks. African‐American EC Nicotine Concentration Order: 1.8%, 5%, 1.8% EC Nicotine Concentration Order: 1.8%, 5%, 5% EC Nicotine Concentration Order: 5%, 1.8%, 1.8% EC Nicotine Concentration Order: 5%, 1.8%, 5% White EC Nicotine Concentration Order: 1.8%, 5%, 1.8% EC Nicotine Concentration Order: 1.8%, 5%, 5% EC Nicotine Concentration Order: 5%, 1.8%, 1.8% EC Nicotine Concentration Order: 5%, 1.8%, 5%
Outcomes	Baseline lab visit 1, lab visit 2. Phase 2: 6 weeks home use Total inhaled volume. Time frame: 2 to 10 days between visits. Differences within participants in total inhaled volume in electronic cigarette puff topography during the pharmacokinetic portions of lab visit 1 and 2 Examine the impact of nicotine concentration on short‐term, real‐world EC use patterns and related biomarkers of exposure (e.g. exhaled carbon monoxide, 4‐(methylnitrosamino)‐1‐(3‐pyridyl)‐1‐butanol (NNAL), lung inflammatory markers)
Starting date	Starting date: 20 February 2023. Estimated primary completion date: 20 February 2025
Contact information	Leah Lambart, MPH, 913‐945‐7862, llambart@kumc.edu Eleanor Leavens, PhD, 913‐588‐3763, eleavens@kumc.edu
Notes	New to 2023 update

**Table:** NCT05960305

Study name	A multi‐site randomized actual use study of electronic nicotine delivery system (P12) products among current U.S. adult smokers to assess the relative impact of availability and use of different e‐liquid flavors on changes in cigarette consumption Brief title: CSD201204 An actual use study of P12 electronic nicotine delivery system among U.S. adult amokers
Methods	Multi‐site, open‐label, randomized, 3‐arm, 8‐week, prospective observational study Setting USA
Participants	1845 participants enrolled Inclusion criteria: 21 to 60 years of age, inclusive, who are regular smokers (≥ 5 cigarettes/day on at least 20 of the past 30 days), indicate "an intention to use" (6 or higher on a 10‐point Likert scale) for at least one Study IP flavour per arm across all 3 study arms. Exclusion criteria: currently quitting or intending to quit within the next 3 months all tobacco or nicotine product use ("currently" is defined as within [≤] 30 days prior to pre‐screening); regular ENDS user (using ENDS > 3 days per week, in the past 30 days), based on self‐report; pregnant or breastfeeding; "poor" physical health; "poor" mental health; employees of a company that manufactures tobacco or ENDS products.
Interventions	Active comparator: 1. Tobacco flavour 2. Menthol flavour 3. NTNM flavour For all can choose between 2 different flavour variants at 2 different nicotine levels (1.5% and 5%). 4 options per arm. Real‐life/naturalistic environments. Subjects will be able to choose freely among the Study IP available in 1 of the 3 study arms to which they are randomly assigned. The 3 study arms are organized by Study IP flavour categories: tobacco, menthol, and non‐tobacco‐non‐menthol (NTNM). Subjects will self‐report their ad libitum use of the Study IP as well as use of combustible cigarettes (CC) and any other tobacco‐ and nicotine‐containing product (TNP) on a daily basis using an electronic diary (eDiary).
Outcomes	Baseline to 6 weeks Number and proportion of subjects who reduce their cigarettes per day (CPD) (over 6 weeks). Number and proportion of subjects who reduce their cigarettes per day (CPD) consumption by at least 50% at Week 6 compared to baseline among all subjects who complete the study. CPD mean per cent reduction over 6 weeks. CPD mean per cent reduction at Week 6 compared to baseline among all subjects who complete the study.
Starting date	Study start date: September 2023 Study completion 2024
Contact information	RAI Services Company. Reynolds American. Tobacco Industry. Study Director: Kristen Jordan, PhD Contact information not provided in NCR record.
Notes	New to 2024 update

**Table:** NCT06063421

Study name	Comparison of nicotine replacement therapy and electronic cigarettes for smoking cessation in Pakistan
Methods	RCT Pragmatic, open‐label, parallel design RCT Setting: Pakistan
Participants	Inclusion criteria: 18 years or older; current smokers presenting to a cessation clinic expressing a desire to quit smoking; capacity to consent; can read and understand the instructions in Urdu and/or English and follow the study instructions and procedures. Exclusion criteria: pregnant or breastfeeding ; using EC or NRT products; enrolled in another similar study; not willing to quit; have had a recent cardiovascular event like unstable angina, stroke or myocardial infarction in the past 2 weeks. Motivated to quit
Interventions	EC vs NRT EC: The intervention consists of the use of an EC device, Vaporesso Gen Air 40 Vape Kit (includes EC device, integrated battery, refillable tank, charging cable, coil replacements and user manual). Three e‐liquid flavours will be provided; tobacco, menthol, and fruit flavour. All e‐liquids will have a nicotine concentration of 18 to 20 mg/mL. EC will be provided for a total of 12 weeks. NRT: NRT combination therapy with a transdermal nicotine patch (7, 14, or 21 mg) and an oral product: either gum or lozenge in 1, 2, or 4 mg strength. Usage will be in the form of a daily nicotine patch and ad libitum use of the faster‐acting lozenge to curb nicotine cravings. Participants will be provided with a 12‐week supply of NRT.
Outcomes	Baseline, 1, 4, 8, 24, and 52 weeks Carbon monoxide (CO) validated sustained abstinence at 24, 52 weeks Self‐reported 7‐day point prevalence abstinence at 4, 24, and 52 weeks AEs, CPD, and product satisfaction rating, 1, 4, 8, 24, and 52 weeks post TQD Intervention cost per participant (12 wks from TQD)
Starting date	Study posted October 2023. Updated March 2024.
Contact information	Fouad Aslam, MPH, 00447494700290, projectdirector@strategichealthresearch.org.uk Aftab Ahmad, MA
Notes	New to 2024 Funding: Foundation for a Smoke Free World INC

**Table:** NCT06077240

Study name	Effects of e‐cigs vs pouches on cigarette smoking and addiction Official title: Evaluating the effects of e‐cigarettes versus oral nicotine pouches and product constituents (menthol flavor, nicotine concentration) on adult cigarette smoking and addiction
Methods	Randomised parallel assignment Triple‐masked (participant, investigator, outcomes assessor) Setting: USA
Participants	Aim to recruit 256 adults who currently smoke CC and willing to switch Inclusion criteria: 21+ years old; English literate; currently smoking cigarettes, biochemically confirmed; not planning a smoking cessation attempt or to use smoking cessation pharmacotherapies (NRT, bupropion, varenicline) in the next month Exclusion criteria: currently using any smoking cessation services and/or pharmacotherapies; pregnant or breastfeeding; significant current medical or psychiatric condition; known hypersensitivity to propylene glycol
Interventions	EC 2.4% nicotine, menthol and tobacco flavours EC 2.4% nicotine with tobacco flavour only EC 5% nicotine with menthol and tobacco flavours EC 5% nicotine with tobacco flavour only Tobacco pouches 3 mg nicotine with menthol and tobacco flavours Tobacco pouches with 3 mg nicotine with tobacco flavour only Tobacco pouches with 6 mg nicotine with menthol and tobacco flavours Tobacco pouches with 6 mg nicotine with tobacco flavour only
Outcomes	Baseline, 4, 6 weeks; 5 visits Plan to share IPD Abstinence, biochemically verified 7‐day point‐prevalent abstinence from cigarettes (to week 4) CPD Cigarette dependence measured using the 4‐item PROMIS® Short Form v1.0 ‐ Smoking Nicotine Dependence for All Smokers 4a. Each item is scored from 1 to 5 with the range of scores from 4 to 20 with higher scores representing greater cigarette dependence. Use of non‐combustible product. Continued use of study product (to 6 weeks).
Starting date	First posted Oct 2023. Last update posted February 2024. Starting date not stated.
Contact information	Lisa M. Fucito, lisa.fucito@yale.edu Krysten W Bold, krysten.bold@yale.edu
Notes	New to 2024 Funding: National Institute on Drug Abuse (NIDA)

**Table:** NCT06088862

Study name	A randomized study to evaluate harm reduction products as a second line intervention for adult smokers who do not quit with nicotine replacement therapy (NRT)
Methods	Randomised parallel assignment Setting: USA
Participants	Estimated enrolment 325 Adults 22 to 65 Inclusion criteria: 22 to 65 years of age (inclusive) at screening; smokes an average of at least 10 commercially available CPD and has done so for the last 12 months; expired air CO reading of at least 10 ppm; interested in switching to an electronic cigarette or nicotine pouch; smartphone Exclusion criteria: medical or physical condition that may affect participant safety, the safety of others, or data validity; planned use of an FDA‐approved smoking cessation product during the study, not provided as part of this protocol; heart problems; taking psychoactive medications (e.g., antipsychotics, benzodiazepines, or mood stabilizers); frequent users (monthly) of smokeless tobacco (chewing tobacco, snuff), cigars (not cigarillos), pipes, hookahs or other non‐commercially available combustible or heated tobacco products; NRT or other smoking cessation treatments within 14 days of screening; pregnant or nursing; not using birth control; enrolled in a clinical trial within 30 days of screening Motivated to quit: participants are willing to switch
Interventions	Interventions: EC: BIDI e‐cigarette. BIDI e‐cigarette (flavours ‐ Classic, Arctic, and Solar); as needed for maximum of 5 weeks Nicoderm, 21 mg nicotine patch daily; maximum usage 9 weeks Nicorette 4 mg chewing gum. 4 mg Nicorette gum (flavours ‐ cinnamon surge, fruit chill, and mint); as needed maximum usage 9 weeks Nicorette lozenge product. 4 mg Nicorette mint lozenge; as needed maximum usage 9 weeks EC: BIDI e‐cigarette. BIDI e‐cigarette (flavours ‐ Classic, Arctic, and Solar); as needed for maximum of 5 weeks on! 4mg on! Nicotine Pouch (flavours ‐ Berry, Mint, and Original); as needed for maximum usage 5 weeks Study arms: Group I ‐ NRT Responder Participants that were successful in quitting smoking using NRT in the first 4 weeks of the study will have the option of remaining on their current NRT or switching to one of the other 2 options for the next 5 weeks. Interventions: Dr Nicoderm; Nicorette 4 mg chewing gum; Nicorette lozenge product. Group IIa NRT Non‐Responder ‐ Pouch Preferrers Participants that were unsuccessful in quitting smoking using NRT in the first 4 weeks and reported a preference to try a nicotine pouch. Randomized to be switched to nicotine pouch for the next 5 weeks. Interventions: Nicoderm; Nicorette 4 mg chewing gum; Nicorette lozenge product; on! (nicotine pouch). Group IIb NRT Non‐Responder ‐ Pouch Preferrers Control Participants that were unsuccessful in quitting smoking using NRT in the first 4 weeks and reported a preference to try a nicotine pouch. Randomized to remain on NRT for the next 5 weeks. Interventions: Nicoderm; Nicorette 4 mg chewing gum; Nicorette lozenge product Group IIIa NRT Non‐Responder ‐ ENDS Preferrers Participants that were unsuccessful in quitting smoking using NRT in the first 4 weeks and reported a preference to try ENDS. Randomized to be switched to ENDS for the next 5 weeks. Interventions: Nicoderm; Nicorette 4 mg chewing gum; Nicorette lozenge product; BIDI e‐cigarette Group IIIb NRT Non‐Responder ‐ ENDS Preferrers Control Participants that were unsuccessful in quitting smoking using NRT in the first 4 weeks and reported a preference to try ENDS. Randomized to remain on NRT for the next 5 weeks. Interventions: Nicoderm; Nicorette 4 mg chewing gum; Nicorette lozenge product
Outcomes	Baseline, 10 weeks Change in expired air CO at week 10 compared to baseline Smoking abstinence (expired air CO < 6 ppm), week 7 to 10 Do not plan to share IPD
Starting date	Study start date: December 2023 Estimated study completion date: December 2024
Contact information	Rose Research Center, North Carolina, USA Names and contact details not provided
Notes	New to 2024 update Collaborators: Foundation for a Smoke Free World INC

**Table:** NCT06111053

Study name	Trial for harm reduction with incentives and vaping e‐cigarettes Official title: Harm reduction in smokers with obesity: impact of contingent incentives and e‐cigarettes
Methods	Randomised, 2x2 factorial design Setting: USA
Participants	Estimated enrolment 36 Inclusion criteria: BMI ≥ 25 kg/m2; smoked ≥ 5 cigarettes/day during the past year; 21 or older; exhaled CO of > 6 ppm at baseline; willing to use ENDs for 6 weeks; daily access to a Bluetooth‐enabled smartphone/tablet Exclusion criteria: planning to set a smoking quit date in the next 30 days; receiving smoking cessation treatment of any kind in the past 30 days; using EC/ ENDS > 4 days per month; hospitalized for mental illness in past 30 days; heart‐related event (e.g. heart attack, severe angina) in past 30 days; resides with another person enrolled in the study; pregnant, nursing, or planning to become pregnant in the next 6 months
Interventions	Interventions: EC/ENDS: participants in active comparator groups that include ENDS will receive 6 weeks' worth of ENDS supplies. Contingent incentives: participants in active comparator groups will receive incentives that vary based on participant abstinence from smoking, measured by a carbon monoxide breath sample. No EC /ENDS: participants in active comparator groups labelled No ENDS will only receive information about the comparative risk of ENDS relative to combustible cigarettes. Non‐contingent incentives. Participants in active comparator groups labelled Non‐Contingent Incentives will receive compensation for each breath sample provided throughout the study, with no variation. Study arms: 1. Experimental: EC and Contingent Incentives Participants in this arm will receive information about the comparative risk of EC relative to smoking as well as 6 weeks' worth of provisions of EC and will receive 4 weeks of monetary incentives for complete abstinence from smoking (after a controlled ramp down of smoking). 2. Experimental: No EC and Contingent Incentives Participants in this arm will receive information about the comparative risk of EC relative to smoking and will receive 4 weeks of monetary incentives for complete abstinence from smoking (after a controlled ramp down of smoking). 3. Experimental: EC and Non‐Contingent Incentives Participants in this arm will receive information about the comparative risk of EC relative to smoking as well as 6 weeks' worth of provisions of EC and will receive monetary incentives for providing breath samples only (non‐contingent on smoking status). 3. Experimental: No EC and Non‐Contingent Incentives Participants in this arm will receive information about the comparative risk of EC relative to smoking and will receive monetary incentives for providing breath samples only (non‐contingent on smoking status).
Outcomes	Baseline 4, 6, and 12 weeks At 4, 6, and 12 weeks Use of EC and CC; abstinence; CO; CC and EC dependence; questionnaire comparing CC and EC; weight assessed; motivation to change; attitudes to EC using Comparing E‐Cigarette and Cigarettes (CEAC) Questionnaire. IPD: plan to share data
Starting date	October 2023 Estimated completion date: December 2024
Contact information	Cara M Murphy, 1 (401) 203‐5339, THRIVE@brown.edu
Notes	New to 2024

**Table:** NCT06118502

Study name	A clinical trial of adaptive treatment for early smoking cessation relapse (ADAPT)
Methods	Randomised sequential assignment, open‐label Setting: Alabama and South Carolina, USA.
Participants	Estimated enrolment 544 Treatment‐seeking people who smoke
Interventions	NRT (patches and lozenges); varenicline. At 8 weeks some non‐responders will be offered EC. Treatments free for 12 weeks Switching to a different medication: 4 weeks of the other FDA‐approved option, either varenicline or combination NRT, with instructions to try to quit again at week 4. Continued use of the same medication: 4 additional weeks of the same medication (varenicline or NRT) with instructions to try to quit again at week 4. Switching to a harm‐reduction tobacco product: 4 weeks of e‐cigarette products with instructions to switch completely at Week 8. Study arms 1. Adaptive Randomization 1 (switching to another medication): people who did not respond to 4 weeks of pharmacotherapy (either varenicline or combination NRT). After a 4‐week course of pharmacotherapy, participants that are not responding to medication will receive 4 weeks of the other FDA‐approved option, either varenicline or combination NRT, with instructions to try to quit again. 2. Non‐Adaptive Randomization 1 (continued use of the same medication) : people who did not respond to 4 weeks of pharmacotherapy (either varenicline or combination NRT). After a 4‐week course of pharmacotherapy, participants that are not responding to the medication will receive 4 additional weeks of the same medication with instructions to try to quit again. 3. Harm Reduction Randomization 2 (switching to a harm‐reduction tobacco product): people who did not respond to two 4‐week courses of pharmacotherapy (either varenicline or combination NRT or both). After two 4‐week courses of pharmacotherapy, participants who are not responding to medication will be randomly assigned to a harm‐reduction group (e‐cigarettes). Participants assigned to the harm‐reduction group will receive 4 weeks of e‐cigarette product with instructions to switch completely. 4. Non‐Adaptive Randomization 2 (continued use of the same medication): people who did not respond to two 4‐week courses of pharmacotherapy (either varenicline or combination NRT or both sequentially). After two 4‐week courses of pharmacotherapy, participants that are not responding to the medication will receive 4 additional weeks of the same medication with instructions to try to quit again.
Outcomes	Baseline, 4, 8, 12, 24 weeks. 8 surveys, all assessments remote. Abstinence from CC. 7‐day point prevalence abstinence.
Starting date	Posted November 2023
Contact information	Tracy T Smith, 8438725164, smithtra@musc.edu Matthew J Carpenter, 8438762436, carpente@musc.edu
Notes	New to 2024

**Table:** NCT06169813

Study name	E‐cigarette harm reduction among PLWHA in South Africa
Methods	RCT Open‐label, parallel assignment Setting: South Africa
Participants	Estimated N = 90 Inclusion criteria: adult PLWHA CC smokers; speaks Afrikaans, or Xhosa, or English; daily CC smoking (≥ 5 CPD); mobile phone; interested in reducing CC smoking but not necessarily trying to quit; receives HIV/AIDS care in one of the 8 selected clinics follow‐up rates Exclusion criteria: pregnant or breastfeeding; unable to provide consent; used tobacco products other than CC in the past 2 weeks (e.g. EC, cigarillo); currently engaged in an attempt to quit CC smoking; current major depressive or manic episode, current psychotic disorder, past‐year suicide attempt, or current suicidal ideation with plan or intent
Interventions	EC: EC VUSE "Solo" single‐use pods. Nicotine ‐ 48 mg/mL (4.8% nicotine) concentration + phone counselling + EMI texting NRT: NRT (daily patches and lozenges). NRT strength will be according to the established dosing guidelines for tobacco treatment. NRT is the standard of care in tobacco treatment and helped reduce CPD in prior trials + phone counselling + ecological momentary intervention (EMI) texting Quit Line: participants will receive referral to the existing South African Quitline. Participants will receive information to contact the Quitline if participants so choose. All groups receive counselling. Each participant will receive up to 5 motivational counselling sessions. The first session will also include orientation of EMA/EMI texting.
Outcomes	Baseline, 8 weeks, 3 months, 6 months 7‐day point prevalence abstinence at 3 months. Abstinence will be verified by exhaled carbon monoxide and defined as no combustible cigarette use in the last 7 days 50% reduction in cigarettes per day (CPD), compared with baseline at 6 months, CPD will be self‐reported Change in American Thoracic Society Questionnaire score Client Satisfaction Questionnaire (CSQ‐8) score at 8 weeks and 3 months Percent of patients who enrol in counselling at 6 months FU rate at 3 and 6 months
Starting date	Estimated start date: Feb 2024 Estimated completion date: June 2024
Contact information	Omar ElShahawy Omar.ElShahawy@nyulangone.org NYU Langone Health
Notes	New to 2024

LC:8‐iso‐PGF2a: an isoprostane 1‐OHP: 1‐hydroxypyrene ACS: acute coronary syndrome AE: adverse event AHR: airway hyperresponsiveness AMI: acute myocardial infarction AUD: alcohol use disorder AUDIT: AUDIT‐C checklist terminology for alcohol dependence BMI: body mass index BP: blood pressure BUP: buprenorphine CAL: clinical attachment loss CAR: continuous abstinence rate CAT: Computerized Adaptive Testing OR Computer‐Aided Tomography CC: combustible cigarette CCQ: Clinical COPD Questionnaire CEMA: 2‐cyanoethylmercapturic acid C‐F NDS: combustion‐free nicotine delivery systems CK‐MB: creatine kinase, heart specific isoenzyme CMHT: Community Mental Health Team CO: carbon monoxide COPD: chronic obstructive pulmonary disease COVID: COVID‐19, disease caused by SARS‐CoV‐2 cpd/CPD: cigarettes per day CRF: cardiovascular risk factors CT: computed tomography CVD: cardiovascular disease CXCL9: CSCL9 (chemokine ligand 9) DESC: DESC refers to a supportive housing project (see NCT03962660) DNA: deoxyribonucleic acid DSM‐IV/5: Diagnostic and Statistical Manual of Mental Disorders‐IV/5 EC: electronic cigarette eCO: expired carbon monoxide ECG: electrocardiogram ECwN: electronic cigarette with nicotine ECwoN: electronic cigarette without nicotine EMI: ecological momentary intervention ENDS: electronic nicotine delivery system EQ‐5D‐5L: EuroQol 5 Dimension 5 Level ER: emergency room FDA: Food and Drug Administration FEF: forced expiratory flow FeNO: fractional exhaled nitric oxide FEV1: forced expiratory volume FPL: federal poverty level FSH: follicle‐stimulating hormone FTND: Fagerström Test for Nicotine Dependence FU: follow‐up FVC: forced vital capacity GIF: graphics interchange format GP: General Practitioner (Dr) HaRTS‐TRENDS: (trial name) Harm reduction for tobacco smoking with HbA1c: haemoglobin A1C, glycosylated haemoglobin HBsAg: hepatitis B surface antigen HCV: hepatitis C HDL: high‐density lipoprotein HEMA: 2‐hydroxyethylmercapturic acid HIV: human immunodeficiency virus HMPMA: 3‐hydroxy‐1‐methyl propylmercapturic acid HPB: Health Promotion Board HPMA: hydroxypropylmercapturic acid hs‐CRP: high‐sensitivity C‐reactive protein HTP: hydroxytryptophan ICD‐10: International Classification of Diseases, Tenth Edition ICF: International Classification of Functioning IL‐6: Interleukin 6 iSREC: immediate standardized research e‐cigarette LDCT: low‐dose computed tomography LHC: lung health check mCEQ: modified Cigarette Evaluation Questionnaire MDD: major depressive disorder MetS: metabolic syndrome MHRA: Medicines and Healthcare products Regulatory Agency MI: myocardial infarction MINI: mini International Neuropsychiatric Interview MMP9: matrix metallopeptidase 9 mMRC: modified Medical Research Council MNWS: Minnesota Nicotine Withdrawal scale  mPES: multi‐Parameter Evidence Synthesis MPSS: mood and physical symptoms scale MTSS: Motivation To Stop Scale MTWS‐R: Minnesota Tobacco Withdrawal Scale‐R (15 items) NHS: National Health Service NIDA: National Institute on Drug Abuse NNC: non‐nicotine cigarette NCCN: National Comprehensive Cancer Network NNAL: carcinogen found in tobacco smoke (4‐(methylnitrosamino)‐1‐(3‐pyridyl)‐1‐butanol) NNN: N'‐nitrosonornicotine NRT: nicotine replacement therapy OHQoL‐UK: Oral Health Quality of Life assessment United Kingdom OUD: opioid use disorder PANSS: Mean Positive and Negative Syndrome Scale PATH: Population Assessment of Tobacco and Health PEF: peak expiratory flow PG: propylene glycol PGEM: a stable metabolite of prostaglandin E2 (biomarker of inflammation)  PHQ‐9: Patient Health Questionnaire 9 PI: principal investigator PK: pharmacokinetic PneT: PheT phenanthrene tetraol PP(A): point prevalence (abstinence) PPD: pocket probing depths PROMPT: Community‐Based Participatory Tobacco Dependence Strategy (PROMPT Project) PS[E]CDI: Penn State Electronic Cigarette Dependence Index (e‐cigarette dependence measure) QN: NHS quit now programme QoL: quality of life q‐PADDA: primer anchored DNA damage detection assay QSU‐Brief: Questionnaire of Smoking Urges QTC: QT interval (time it takes for the electrical system to fire an impulse through the ventricles and then recharge) RA: research assistant RC: research cigarettes RCT: randomised controlled trial REDCAP: Research Electronic Data Capture (web application for surveys) SABA: short‐acting β2‐agonists SAE: serious adverse event SC: e‐salivary cotinine SCP: smoking cessation programme SES: socioeconomic status SMI: serious mental illness S‐PMA: S‐phenylmercapturic acid SpO2: oxygen saturation SREC: standardized research e‐cigarette SRMH: self‐rated mental health SSS: stop‐smoking services T2DM: type 2 diabetes TC: tobacco cigarette THP: tobacco heating products TLFB: timeline follow back TMS: transcranial magnetic stimulation TNE: total nicotine equivalents TNF‐a: tumour necrosis factor alpha TQD: target quit date UC: usual care USB: universal serial bus V: volts VAR: varenicline VBA: very brief advice VLNC: very low nicotine content VNP: vaporized nicotine products VO2: oxygen consumption WBC: white blood cell WISDM‐Brief: Wisconsin Inventory of Smoking Dependence Motives‐Brief wk: week WLC: waiting‐list control YLST: Yorkshire Lung Screening Trial yr: year

## Differences between protocol and review

At the 2023 update we added a new outcome ‐ association between withdrawal and cessation measured at six months or longer ‐ and three new network meta‐analyses. These were prespecified in a published protocol that can be found at the following link: https://osf.io/gznsr.

The protocol did not specify a minimum follow‐up period for data on adverse events. As of the 2016 update, we have changed the Methods section to clarify that we will exclude follow‐up data at less than a week.

The original version of this review included reduction as a secondary outcome. The 2016 update removed reduction as an outcome, to bring the review into line with other reviews of cessation treatments produced by Cochrane Tobacco Addiction and to prevent substantial overlap with the update of the Group's review of interventions for harm reduction.

As prespecified in the 2016 update, in the 2020 update, we excluded non‐intervention studies. In the 2020 update, we also added an appendix with a protocol setting out our plans to convert this review into a living systematic review in the future.

As specified in an amendment in June 2021, we now include a new secondary outcome: number of people still using study product (EC or pharmacotherapy) at longest follow‐up (6+ months).

There were too few studies to conduct subgroup analyses for our pairwise analyses to investigate differences between studies, such as: intensity of behavioural support used; EC type (e.g. cartridge; refillable; pod); instructions for EC use (e.g. length of provision, choice); and type of participant (e.g. experience of EC use). For most analyses, there were also too few studies to conduct funnel plots.

For the next update of this review, we will change over from fixed‐effect to random‐effects meta‐analyses in accordance with evolving guidance in this space.
